# Genome-wide identification and evolutionary analyses of the *PP2C* gene family with their expression profiling in response to multiple stresses in *Brachypodium distachyon*

**DOI:** 10.1186/s12864-016-2526-4

**Published:** 2016-03-03

**Authors:** Jianmei Cao, Min Jiang, Peng Li, Zhaoqing Chu

**Affiliations:** Shanghai Key Laboratory of Plant Functional Genomics and Resources, Shanghai Chenshan Botanical Garden, Shanghai, 201602 China; Shanghai Chenshan Plant Science Research Center, Chinese Academy of Sciences, Shanghai, 201602 China

**Keywords:** *B. distachyon*, Protein phosphatases 2C (PP2C), Phylogenetic analysis, Gene expression profiling

## Abstract

**Background:**

The type-2C protein phosphatases (PP2Cs), negatively regulating ABA responses and MAPK cascade pathways, play important roles in stress signal transduction in plants. *Brachypodium distachyon* is a new model plant for exploring the functional genomics of temperate grasses, cereals and biofuel crops. To date, genome-wide identification and analysis of the *PP2C* gene family in *B. distachyon* have not been investigated.

**Results:**

In this study, 86 *PP2C* genes in *B. distachyon* were identified. Domain-based analyses of PP2C proteins showed that they all contained the phosphatase domains featured as 11 conserved signature motifs. Although not all phosphatase domains of BdPP2C members included all 11 motifs, tertiary structure analysis showed that four residues contributing to magnesium/manganese ions (Mg^2+^/Mn^2+^) coordination were conserved, except for two noncanonical members. The analysis of their chromosomal localizations showed that most of the *BdPP2C* genes were located within the low CpG density region. Phylogenetic tree and synteny blocks analyses among *B. distachyon, Arabidopsis thaliana* and *Oryza sativa* revealed that all PP2C members from the three species can be phylogenetically categorized into 13 subgroups (A–M) and *BdPP2Cs* were evolutionarily more closely related to *OsPP2Cs* than to *AtPP2Cs*. Segmental duplications contributed particularly to the expansion of the *BdPP2C* gene family and all duplicated *BdPP2C*s evolved mainly from purifying selection. Real-time quantitative reverse transcription PCR (qRT-PCR) analysis showed that *BdPP2Cs* were broadly expressed in disparate tissues. We also found that almost all members displayed up-regulation in response to abiotic stresses such as cold, heat, PEG and NaCl treatments, but down-regulation to biotic stresses such as Ph14, Guy11 and F0968 infection.

**Conclusions:**

In the present study, a comprehensive analysis of genome-wide identification and characterization of protein domains, phylogenetic relationship, gene and protein structure, chromosome location and expression pattern of the *PP2C* gene family was carried out for the first time in a new model monocot, i.e., *B. distachyon*. Our results provide a reference for genome-wide identification of the *PP2C* gene family of other species and also provide a foundation for future functional research on *PP2C* genes in *B. distachyon*.

**Electronic supplementary material:**

The online version of this article (doi:10.1186/s12864-016-2526-4) contains supplementary material, which is available to authorized users.

## Background

Wild plants are usually consistently exposed to various environmental challenges such as drought, high salinity, extreme temperature, heavy metals and pathogen infections, which affect their growth and development. To adapt to these unpredictable environmental stresses, plants have evolved signaling mechanisms to transmit stimuli to different cellular compartments and then respond to these stresses. Accumulating evidence indicates that reversible protein phosphorylation catalyzed by protein kinases and phosphatases plays important roles in cellular stresses signal transduction in plants [1]. In the past decades, several protein kinases have been extensively investigated and proved to be positive regulation factors responding to a diversity of abiotic and biotic stresses [2–5]. In contrast to the protein kinases, protein phosphatases are not well investigated.

Protein phosphatases modify protein function by removing the phosphate group from phosphorylated proteins. They are divided into three major classes: tyrosine phosphatases, serine/threonine phosphatases and dual-specificity phosphatases (DSPTPs). According to distinct amino acid sequences, different dependencies on metal ions and sensitivities to inhibitors such as cyclosporine A and okadaic acid, protein serine/threonine phosphatases can be classified into the phosphor-protein phosphatases (PPP), phosphoprotein metallophosphatases (PPM) and aspartate-based protein phosphatases [6]. The PPP family includes type 1 (PP1), type 2A (PP2A), type 4 (PP4), type 5 (PP5), type 6 (PP6), type 7 (PP7) and type 2B (PP2B), whereas the PPM family is represented by the Mg^2+^- or Mn^2+^- dependent type-2C protein phosphatases (PP2Cs) [7].

PP2Cs, evolutionarily conserved and found in archaea, bacteria, fungi, plants and animals, are implicated in regulating stress-signaling pathways and act as negative modulators of protein kinase cascades activated by diverse stresses. For instance, a PP2C phosphatase, RsbP, in *Bacillus subtilis* dephosphorylates RsbV (an anti-anti-σ factor) and mediates the regulation during energy stress [8]. In *Saccharomyces cerevisiae*, PP2Cs play a negative role in the signal-transduction process responding to osmotic stress by counteracting the mitogen-activated protein kinase (MAPK) pathway [9]. Furthermore, one of the human PP2Cs was shown to inactivate the AMP-activated protein kinase (AMPK), a central component of the protein kinase cascade activated by ATP depletion [10]. In higher plants such as *Arabidopsis*, *PP2C* genes were demonstrated to regulate signaling pathways negatively by opposing the action of particular protein kinases. At least eight PP2Cs from subgroup A in *Arabidopsis* have been characterized as key factors in ABA signaling transduction. Briefly, subgroup A PP2Cs inactivate SnRK2 via dephosphorylation, and this inactivation is inhibited by ABA receptors, PYR/PYL/RCRA, in an ABA-dependent manner [11]. The subgroup B PP2C, AP2C1, interacts with MPK4 or MPK6 and subsequently suppresses MAPK activities during wounding as well as pathogen stresses [12]. The subgroup C PP2C POL or PLL1 interacts with the receptor kinase CLV1, inducing and maintaining stem cell polarity [13, 14]. The subgroup E PP2C AtPP2C6-6 interacts with *Arabidopsis* histone acetyl transferase GCN5 and controls the activation of stress-responsive genes in the stomatal signaling network [15–17]. The subgroup F PP2C WIN2 may interact with the bacterial effector HopW1-1 and regulate HopW1-1-induced plant resistance [18]. The unclustered PP2C KAPP interacts with different receptor-like protein kinases (RLKs) and is predicted to control plant immunity responses or hormone signaling [19, 20]. To date, there are 80 and 90 genes coding for PP2C proteins identified using bioinformatics surveys in *Arabidopsis* and rice, respectively [21–24]. However, few *PP2C* genes in monocots have been functionally investigated.

*B. distachyon* is a new model monocot for exploring the functional genomics of temperate grasses, cereals and biofuel crops. In 2010, the complete genome of the *B. distachyon* Bd21 was sequenced and the sequence data can be easily obtained via the *Brachypodium* Genome Resource (http://www.brachypodium.org) [25]. To our knowledge, a genome-wide analysis of the *PP2C* gene family in *B. distachyon* has not been reported so far. Here, we first identified 86 PP2Cs based on phosphatase domains analysis. Then, we further examined whether all phosphatase domains of BdPP2C members harbored magnesium/manganese ions (Mg^2+^/Mn^2+^) coordination residues through protein structural analysis. We also investigated *BdPP2C* genes chromosomal localization, constructed the phylogenetic tree of all *BdPP2C* genes based on their PP2C domains and categorized them into 13 subgroups. Subsequently, we analyzed the duplication events contributing to the expansion and functional divergences of the *BdPP2C* gene family. In addition, we examined the expression profiles of *BdPP2C* genes in different tissues and their responses to different phytohormone treatments as well as various abiotic and biotic stresses. Our results provide a foundation for future functional analysis of the *PP2C* gene family in stress responses in *B. distachyon*.

## Results and discussion

### Genome-wide identification and characterization of *BdPP2C* genes

Protein phosphatase PP2Cs are evolutionarily conserved [26]. During the evolution from prokaryotes to multicellular eukaryotes, the number of *PP2C* genes increased from one member to as many as 130 members. In previous reports, one PP2C member in *Thermococcus*, three in *Synechocaccus*, four in *Bacillus*, seven in *Saccharomyces*, 34 in *Chlamydomonas*, 51 in *Physcomitrella*, 57 in *Selaginella*, 80 in *Arabidopsis*, 90 in *Oryza* and ~130 in *Zea mays* were characterized [26]. The increase and expansion of *PP2C* genes from Archaea to higher plants may correlate with adaptations to complex environmental conditions for plants. To identify *PP2C* candidate genes in *B. distachyon*, the InterPro PP2C domain “IPR001932” was used to search the Plaza2.5 database (http://bioinformatics.psb.ugent.be/plaza/versions/plaza_v2_5/) and 88 putative *PP2C* genes were found. By using Pfam and SMART domain identification tools, we found that 2 of the 88 putative PP2C genes lacked PP2C catalytic domains. Therefore, 86 genes in *B. distachyon* were identified as *PP2C* family members. The 86 *BdPP2C* genes identified in this study encode proteins varying from 281 to 1087 amino acids in length, with large variations in isoelectric point (pI) values from 4.24 to 9.31 and molecular weight from 30 kDa to 120 kDa. The subcellular localization prediction indicated that most of the BdPP2C proteins might be located in cytoplasm, chloroplast or nucleus, while only a few might be located in cytoskeleton or mitochondria (Table 1). A total of 86 *PP2C* genes were anchored to corresponding chromosomes and designated as *BdPP2C1*–*BdPP2C86* according to their order on the chromosomes.

**Table 1:** List of 86 *BdPP2C* genes and their basic characterizations

Gene identifier	Gene name	Size (aa)	Mass (kDa)	pI	Subcellular localization	Subgroup
Bradi1g02920	BdPP2C1	383	41.607	6.7	cyto	D
Bradi1g03690	BdPP2C2	662	71.157	5.62	chlo	C
Bradi1g04520	BdPP2C3	474	50.021	8.36	chlo	K
Bradi1g04540	BdPP2C4	566	61.298	6.36	chlo	M
Bradi1g07870	BdPP2C5	379	41.509	9.31	chlo	D
Bradi1g16630	BdPP2C6	381	40.055	8.83	chlo	B
Bradi1g16810	BdPP2C7	432	46.273	7.5	chlo	H
Bradi1g19620	BdPP2C8	476	49.814	5.29	cyto	L
Bradi1g24400	BdPP2C9	428	46.447	5.83	nucl	E
Bradi1g26690	BdPP2C10	290	31.918	6.01	cyto	F
Bradi1g30200	BdPP2C11	392	43.45	8.77	mito	D
Bradi1g31080	BdPP2C12	366	40.344	4.94	nucl	I
Bradi1g33900	BdPP2C13	281	30.172	5.01	cyto	F
Bradi1g36330	BdPP2C14	309	33.654	6.25	cyto	K
Bradi1g36690	BdPP2C15	309	33.423	5.51	cyto	K
Bradi1g36920	BdPP2C16	359	38.823	4.85	cyto	G
Bradi1g37500	BdPP2C17	312	33.409	6.33	chlo	K
Bradi1g37530	BdPP2C18	312	33.409	6.33	chlo	K
Bradi1g38670	BdPP2C19	362	38.975	8.9	chlo	F
Bradi1g47710	BdPP2C20	353	38.882	5.01	cyto	G
Bradi1g54080	BdPP2C21	263	29.067	7.75	extr	M
Bradi1g54110	BdPP2C22	690	75.85	7.81	chlo	M
Bradi1g60520	BdPP2C23	450	48.306	5.45	chlo	H
Bradi1g64780	BdPP2C24	433	46.283	7.02	chlo	H
Bradi1g65520	BdPP2C25	387	40.918	6.63	chlo	B
Bradi1g66650	BdPP2C26	625	68.08	6.37	nucl	C
Bradi1g66920	BdPP2C27	405	43.564	5.59	nucl	A
Bradi1g70680	BdPP2C28	403	43.323	8.57	chlo	D
Bradi1g71690	BdPP2C29	582	60.563	4.28	chlo	K
Bradi1g75940	BdPP2C30	399	44.021	9.12	nucl	D
Bradi2g03970	BdPP2C31	321	34.228	4.95	chlo	K
Bradi2g11350	BdPP2C32	386	42.481	4.81	cyto	G
Bradi2g13820	BdPP2C33	470	50.126	8.81	nucl	H
Bradi2g14420	BdPP2C34	424	44.985	5.35	nucl	A
Bradi2g14740	BdPP2C35	503	54.522	4.67	nucl	F
Bradi2g15840	BdPP2C36	385	41.206	5.66	nucl	A
Bradi2g18510	BdPP2C37	374	40.455	5.63	chlo	A
Bradi2g27970	BdPP2C38	340	37.233	7.5	chlo	E
Bradi2g27977	BdPP2C39	315	34.512	5.3	chlo	E
Bradi2g38640	BdPP2C40	385	41.564	4.69	cyto	G
Bradi2g38770	BdPP2C41	546	59.207	5.74	chlo	C
Bradi2g40550	BdPP2C42	656	73.068	5.96	cyto	\
Bradi2g40950	BdPP2C43	390	42.049	6.41	chlo	M
Bradi2g41950	BdPP2C44	480	49.799	4.87	cyto	A
Bradi2g43700	BdPP2C45	289	31.763	4.89	cysk	F
Bradi2g45470	BdPP2C46	392	42.104	5.47	chlo	A
Bradi2g54810	BdPP2C47	403	42.912	6.06	nucl	A
Bradi2g62650	BdPP2C48	384	42.197	8.56	mito	D
Bradi3g03480	BdPP2C49	380	39.678	5.3	chlo	K
Bradi3g03870	BdPP2C50	289	30.628	5.32	chlo	F
Bradi3g05990	BdPP2C51	372	40.923	5.14	nucl	I
Bradi3g08390	BdPP2C52	366	38.917	5.86	chlo	E
Bradi3g09560	BdPP2C53	361	38.717	6.68	chlo	F
Bradi3g10350	BdPP2C54	1087	120.827	5.05	chlo	L
Bradi3g18090	BdPP2C55	318	34.579	7.15	chlo	F
Bradi3g25590	BdPP2C56	282	29.87	4.59	cyto	K
Bradi3g32240	BdPP2C57	402	43.492	6.36	nucl	G
Bradi3g32480	BdPP2C58	395	43.639	8.25	chlo	D
Bradi3g39540	BdPP2C59	525	56.595	5.06	chlo	E
Bradi3g43360	BdPP2C60	521	55.581	7.6	chlo	E
Bradi3g43430	BdPP2C61	418	45.232	5.13	nucl	I
Bradi3g43440	BdPP2C62	290	31.948	5.66	nucl	I
Bradi3g46160	BdPP2C63	268	28.242	5.58	cyto	K
Bradi3g46430	BdPP2C64	452	48.558	5.87	chlo	H
Bradi3g48280	BdPP2C65	378	41.453	5.48	mito	I
Bradi3g49540	BdPP2C66	365	38.711	5.59	cyto	K
Bradi3g49550	BdPP2C67	324	36.604	5.33	cyto	K
Bradi3g52110	BdPP2C68	596	64.92	5.44	chlo	C
Bradi3g54290	BdPP2C69	360	40.2	5.1	cysk	G
Bradi4g03520	BdPP2C70	390	42.584	9.2	cyto	D
Bradi4g15117	BdPP2C71	1022	116.521	5.87	cyto	J
Bradi4g19660	BdPP2C72	438	47.799	6.24	cyto	E
Bradi4g21510	BdPP2C73	378	40.097	5.94	chlo	B
Bradi4g27880	BdPP2C74	360	39.49	7.14	nucl	\
Bradi4g28100	BdPP2C75	363	38.537	5.79	nucl	A
Bradi4g32230	BdPP2C76	441	48.086	5.77	chlo	H
Bradi4g37710	BdPP2C77	362	39.39	4.85	nucl	I
Bradi4g40490	BdPP2C78	392	41.132	8.02	chlo	B
Bradi4g44750	BdPP2C79	436	47.474	5.23	cyto	E
Bradi5g08830	BdPP2C80	519	57.198	4.94	chlo	C
Bradi5g11780	BdPP2C81	444	47.535	4.24	chlo	H
Bradi5g11980	BdPP2C82	282	30.706	6.26	cyto	F
Bradi5g14730	BdPP2C83	431	47.974	4.38	cyto	I
Bradi5g19410	BdPP2C84	393	43.381	6.33	cyto	D
Bradi5g21140	BdPP2C85	316	34.51	8.6	chlo	F
Bradi5g24530	BdPP2C86	284	30.813	4.87	nucl	F

cyto cytoplasm, chlo chloroplast, nucl nucleus, mito mitochondria, extr extracellular, cysk cytoskeleton

**Fig. 1:**
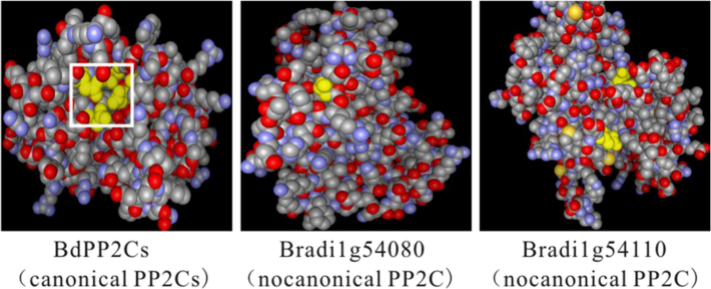
The predicted tertiary structure of BdPP2C domains showing their conserved Mg^2+^/Mn^2+^coordination sites in *B. distachyon*. The tertiary structures of BdPP2C domains were predicted using the Phyre2 tool. The yellow regions indicated by white boxes represent conserved residues that contribute to Mg^2+^/Mn^2+^ coordination

The PP2C phosphatase catalytic domain has been reported to harbor 11 conserved motifs, in which 4 conserved residues contribute to Mg^2+^/Mn^2+^ coordination [27, 28]. So we further checked if BdPP2Cs’ phosphatase catalytic domain harbored 11 conserved motifs. The result obtained from multiple alignments of the 86 BdPP2C domains indicated that not all of the BdPP2C members contained all of the 11 conserved motifs. For example, Bradi1g54110, Bradi3g03870, Bradi3g43440 and Bradi2g27977 were found to be partial deletions in the C-terminal of PP2C phosphatase catalytic domain, thus probably resulting in elimination of a few important motifs and loss of functions. Further analysis showed that scattered amino acid residues also existed among conserved motifs in several BdPP2C members, which suggests that the amino acid composition of plant PP2C domains is quite complex (Additional file 1: Figure S1). In addition, the 3-D structure prediction of 86 BdPP2C domains also showed the diversities of PP2C proteins (Additional file 2: Figure S2). However, as shown in Additional file 1: Figure S1, the residues contributing to Mg^2+^/Mn^2+^coordination in BdPP2Cs are absolutely conserved. The conserved amino acid residues [xxD], [DGxxG], [DG] and [GxxDN] (D, aspartic acid; G, glycine; N, asparagine) were found within motifs 1, 2, 8 and 11, respectively. The predicted protein tertiary structures further revealed that these 4 conserved residues were spatially clustered together in 84 BdPP2Cs (designated as canonical BdPP2Cs), except Bradi1g54080 and Bradi1g54110, which were designated as noncanonical members (Fig. 1, Additional file 3: Figure S3).

### Phylogenetic, gene structural and protein domain analyses of BdPP2C

**Fig. 2:**
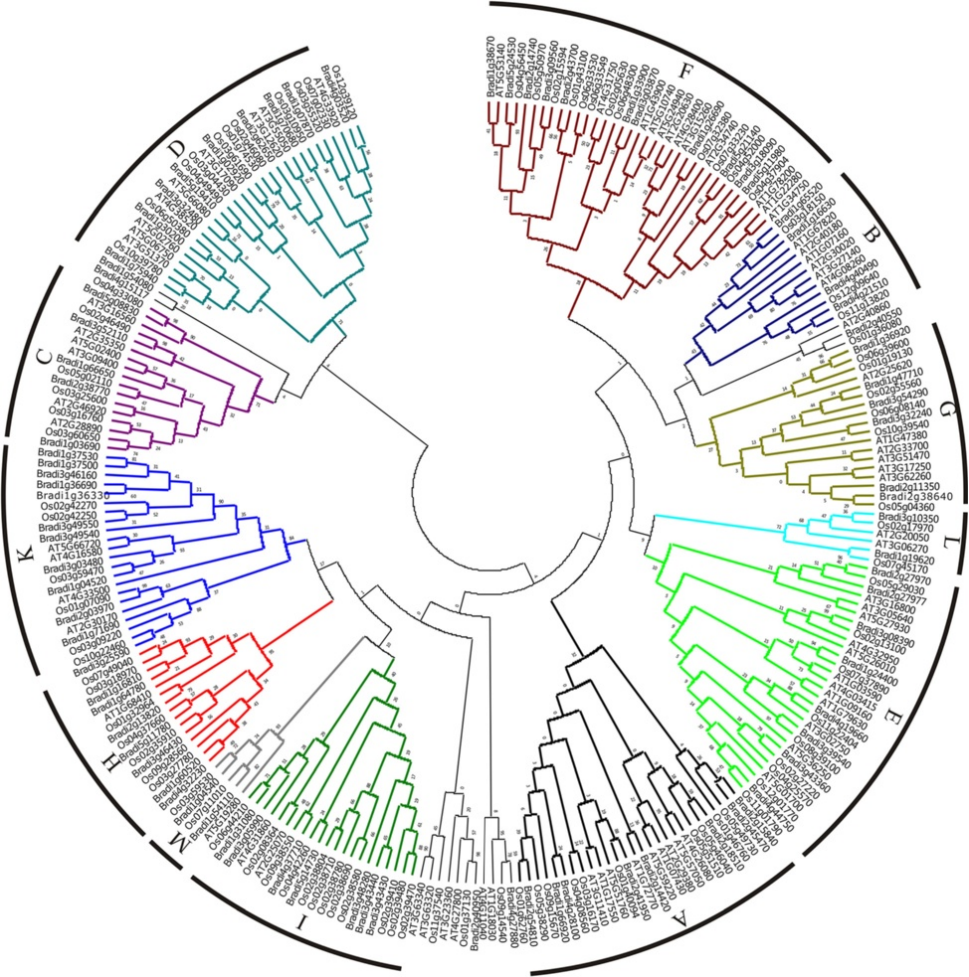
Phylogenetic analysis of PP2C proteins among *B. distachyon*, *Arabidopsis* and rice. Alignments of 256 PP2C domains from *B. distachyon*, *Arabidopsis* and rice were performed with ClustalW2, and the phylogenetic tree was constructed using the ML method with MEGA 6.0

To investigate the phylogenetic relationships of *PP2C* genes between *B. distachyon* and other plants, we constructed a phylogenetic tree based on the alignments of PP2C domains in *B. distachyon*, *Arabidopsis* and rice using the maximum likelihood (ML) method. The phylogenetic analyses indicated that the 80 BdPP2C proteins were divided into 12 subfamilies (A–L), which is consistent with the PP2C groups found in *Arabidopsis* and rice. Each subfamily tree includes PP2C protein from *B. distachyon*, *Arabidopsis* and rice (Fig. 2). Not surprisingly, the BdPP2C clustered together with those from rice because both *B. distachyon* and rice are monocots, while AtPP2C tended to form independent branches. Furthermore, the distributions of PP2Cs in each subfamily were similar among *B. distachyon*, *Arabidopsis* and rice except for the subgroups I and K (Table 2), which suggests that the *PP2C* gene family has evolved from one common ancestor in the three plants. The other three members, i.e., Bradi1g04540, Bradi1g54110 and Bradi1g54080, grouped into a new clade, M. In addition, the remaining three members, i.e., Bradi2g40550, Bradi4g27880 and Bradi2g40950, did not cluster with any other group, and each of them formed a single branch (Fig. 2).

**Table 2:** The distribution of *PP2C* genes in *Arabidopsis*, rice and *B. distachyon*

Subgroup of PP2C genes	Numbers of AtPP2Cs	Numbers of OsPP2Cs	Numbers of BdPP2Cs
A	10	10	8
B	6	3	4
C	7	6	5
D	9	11	9
E	12	12	8
F	13	11	11
G	6	8	6
H	3	7	7
I	2	12	7
J	2	1	1
K	3	6	12
L	2	0	2
M	0	0	3
Single branch 1	1	1	1
Single branch 2	1	1	1
Single branch 3	2	1	1

**Fig. 3:**
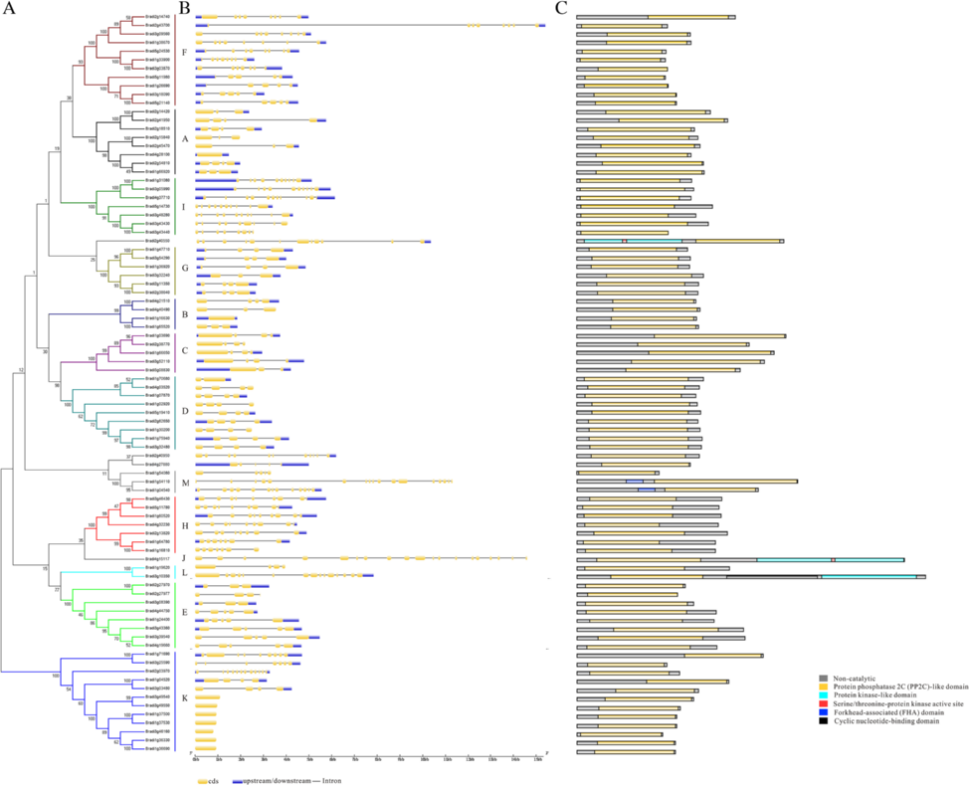
Phylogenetic relationship, gene structure and domain analysis of PP2Cs in *B. distachyon*. **a** The phylogenetic tree was constructed from alignments of the 86 PP2C domains in *B. distachyon* using the ML method with MEGA 6.0. 86 *BdPP2C* proteins were clustered into 13 groups (A–M), designated with different colors, except for three ungrouped members. **b** The exon/intron structure of each *BdPP2C* gene was proportionally displayed according to the scale at the bottom. Yellow boxes represent exons, gray lines represent introns and blue boxes represent untranslated regions. **c** The distribution of conserved domains within BdPP2C proteins. The relative positions of each domain are shown in color boxes, with the name indicated on the right

To determine the phylogenetic relationships among the BdPP2C proteins, we constructed a phylogenetic tree from the alignments of the 86 PP2C domains identified above. As shown in Fig. 2 and Fig. 3a, all of the BdPP2C proteins still remained in the same subfamily. Because the pattern diversity of exon/intron structure and protein domain often plays an important role in the evolution of gene families, the patterns of exon/intron structure of *BdPP2C* genes and conserved domains were examined according to their phylogenetic relationships. The investigation of exon/intron structures revealed that the majority of members in the same subfamily shared similar exon numbers and different exon and intron lengths. However, three members in the M group, consisting of 5, 13 and 17 exons, were largely distinct in exon/intron arrangements, although their phylogenetic relationship was supported by 100 % bootstrap value. More interestingly, almost all BdPP2Cs lacking introns were members of the K subgroup, and the number of PP2C members in the K subgroup in *B. distachyon* was four or two times more than that in *Arabidopsis* and rice, respectively (Fig. 3b, Table 2). Previous studies suggested that genes lacking introns would rapidly evolve via gene duplication events [29–31]. Therefore, the expansion of the BdPP2C K subfamily may be associated with gene duplication events.

When analyzing the phosphatase domains, we found that other domains were associated with the main PP2C domain, including S-TKc (ser/thr kinase catalytic domain), FHA (forkhead associated domain) and CNB (cyclic nucleotide-binding domain). As shown in Fig. 3c, S-TKc was found in Bradi3g10350, Bradi4g15117 and Bradi2g40550; FHA was identified in Bradi1g54110 and Bradi1g04540; CNB was only present in Bradi3g10350. However, the transmembrane region identified in AtPP2Cs and OsPP2Cs was not found in any BdPP2Cs. The S-TKc or FHA domains have been found in several *Arabidopsis* and rice *PP2C* genes, including AT2G40860, Os01g36080 and Os11g37540 or At5G19280, Os03g59530 and Os07g11010. PP2Cs with FHA domain known as the KAPP (kinase-associated protein phosphatase) were reported to play important roles in interacting with RLK and have been characterized as the first downstream negative regulator of RLK in plant development in *Arabidopsis* [18]. Hence, it would be interesting to investigate the different biological functions of *BdPP2C*s harboring these special domains.

### Chromosomal location and duplications of *BdPP2C* genes

**Fig. 4:**
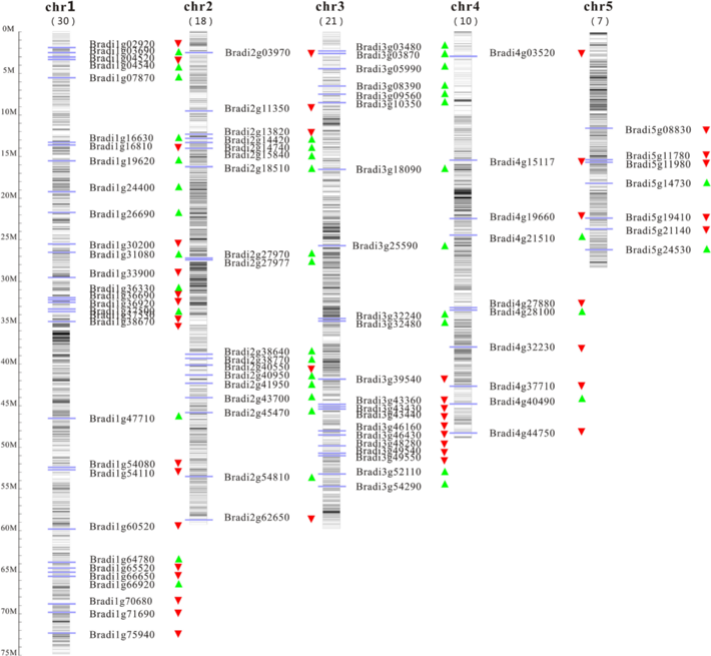
Genomic distribution of *BdPP2C* genes on each chromosome. 86 *BdPP2C* genes were mapped onto specific chromosomes, and the number of *BdPP2Cs* on each chromosome is indicated. The small green or red triangles show the direction of transcription for genes. The deeper color on the chromosomes’ region represents the higher density of CpG islands

To analyze the genomic distribution of *BdPP2C* genes, we marked their approximate position on each chromosome based on the information obtained from the *Brachypodium* genome database. The results showed that all of the 86 *BdPP2C* genes were localized on five chromosomes and their distribution appeared to be uneven. Further analysis indicated that the number of *BdPP2C* genes on each chromosome was proportional to the chromosome length. 30, 18, 21, 10 and 7 *BdPP2C* genes were mapped on chromosome 1, 2, 3, 4 and 5, respectively (Fig. 4). We also marked the densities of CpG islands on each chromosome. As shown in Fig. 4, the deeper color region on chromosomes represents the higher density of CpG islands; the gray or white region on chromosomes represents the lower density of CpG islands. We found that most of the *BdPP2C* genes were located on gray or white regions, indicating that they might be transcriptionally active.

**Fig. 5:**
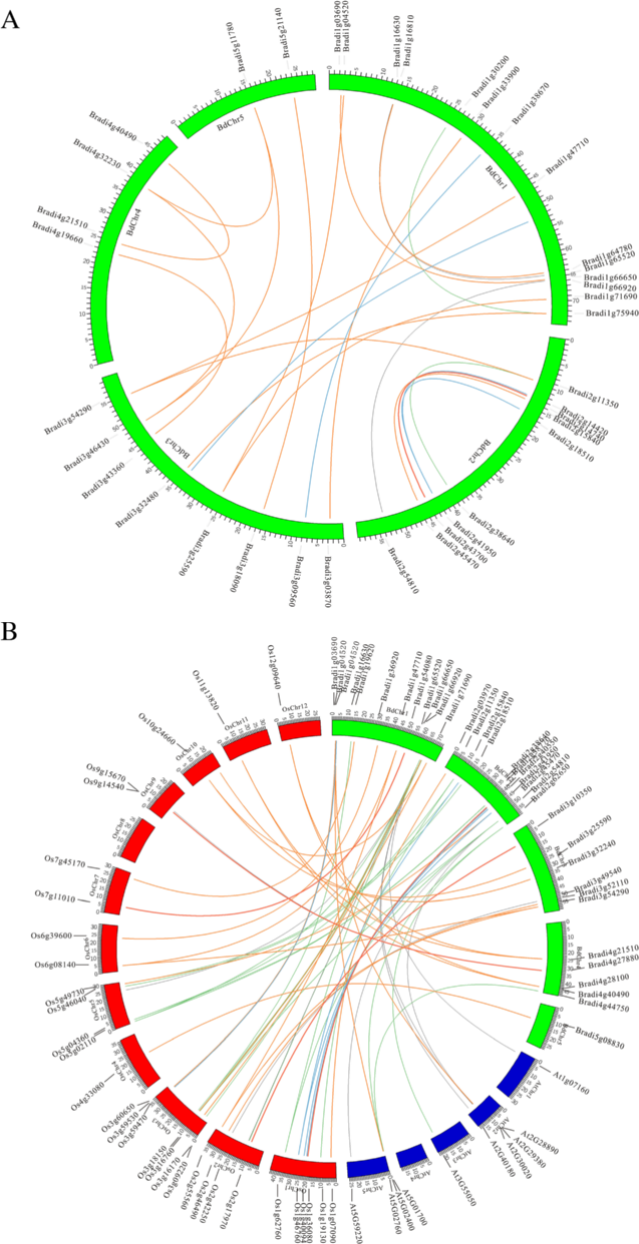
Duplication event analysis of *BdPP2C* genes and comparative synteny analysis among *B. distachyon*, *Arabidopsis* and rice. **a** The data were derived from the Plant Genome Duplication Database, and 22 couples of duplicated *BdPP2C* genes were anchored to corresponding positions on *B. distachyon* chromosomes using the CIRCOS program. **b** Synteny between *B. distachyon* and *Arabidopsis* or between *B. distachyon* and rice was anchored to the corresponding position on specific chromosomes using the CIRCOS program. *B. distachyon* chromosomes are depicted as green segments, and *Arabidopsis* and rice are shown in blue and red, respectively. The size of chromosomes was consistent with the actual pseudo-chromosome size. Positions are in Mb

During evolutionary processes, large segmental duplications and small-scale tandem duplications are two major mechanisms to generate new genes, which contribute to the genome complexities in the plant kingdom [32]. Indeed, previous studies have reported that *PP2C* gene families mainly expanded through whole-genome and chromosomal segment duplications, both in rice and *Arabidopsis* [23]. In *B. distachyon*, paralogous relationship analysis showed that six major chromosomal duplication events happened among the *B. distachyon* chromosomes, covering 92.1 % of the whole genome [25]. Closely related genes located within a distance of less than 200 kb on the same chromosome are defined as tandem duplications, otherwise they are segmental duplications [33]. According to this principle, 22 pairs of paralogous *BdPP2C* genes were found to be involved in segmental duplication events and no tandem duplication gene pairs were found in the *BdPP2C* family. Analysis of these 22 pairs of segmental duplication *BdPP2C* genes showed that 16 were located in 6 major duplicated chromosomal blocks (Fig. 5a). Amino acid alignment analysis indicated that two counterparts of each gene pair were from the same subgroup (Table 3). To determine the selection pressures for these duplicated *BdPP2C* genes, we calculated the substitution ratio of non-synonymous (Ka) to synonymous (Ks) mutations for these 22 pairs of duplicated *BdPP2C* genes. The ratio Ka/Ks can be used to measure the selection acting among the duplicated gene pairs. Commonly, if the value of Ka/Ks is less than 1, the duplicated gene pairs may evolve from purifying selection (also called negative selection); Ka/Ks = 1 means neutral selection; while Ka/Ks > 1 means positive selection [34]. The result showed that Ka/Ks for 22 pairs of duplicated *BdPP2C* genes was less than 1, suggesting that all duplicated *BdPP2C* genes have evolved mainly from purifying selection (Table 3). We also calculated the divergence time (as T = Ks/2λ) among 22 pairs of duplicated *BdPP2C* genes based on a clock-like rate of 6.5 × 10^–9^ mutations per synonymous site per year, as proposed previously [35]. The result in Table 3 showed that divergence events of duplicated *BdPP2C* genes were estimated to have occurred around 50.8–131.5 Mya (million years ago), and the majority of gene pairs diverged long before the divergence time of grass species (56–73 Mya) [25, 36]. This finding is consistent with a previous report that genes involved in conserved signal transduction regulation pathways are preferentially retained [37], and this could explain why many *BdPP2C* genes were retained during the long evolutionary history. In addition, we examined orthologous *PP2C* gene pairs between *B. distachyon* and *Arabidopsis* and between *B. distachyon* and rice. As shown in Fig. 5b, 34 *BdPP2C* genes have one or two putative orthologs in rice; however, only seven *BdPP2C* genes have orthologs in *Arabidopsis*, which suggests that the majority of *BdPP2C*s’ orthologs appeared after the divergence of monocots and dicots. Considering that gene orthologs often share similar functions [38], we infer that the functions of *B. distachyon PP2C* genes have more similarity with rice.

**Table 3:** *BdPP2Cs* present in segmental duplication in *B. distachyon* genome

Locus ID	Subgroup	Ka	Ks	Ka/Ks	Divergence time (Myr)
BdPP2C2/26	C	0.52	1.00	0.52	76.9
BdPP2C3/56	K	0.68	1.55	0.438709677	119.2
BdPP2C7/24	H	0.10	0.76	0.131578947	58.5
BdPP2C11/30	D	0.2	0.78	0.25641026	60
BdPP2C13/50	F	0.11	1.34	0.082089552	103.1
BdPP2C19/53	F	0.28	0.75	0.373333333	57.7
BdPP2C20/69	G	0.14	1.13	0.123893805	86.9
BdPP2C25/6	B	0.38	1.51	0.251655629	116.2
BdPP2C27/47	A	0.35	0.76	0.460526326	58.5
BdPP2C29/56	K	0.25	1.00	0.25	76.9
BdPP2C30/58	D	0.15	0.88	0.170454545	67.7
BdPP2C32/40	G	0.22	1.12	0.1964285	86.2
BdPP2C34/44	A	0.25	1.41	0.177304965	108.5
BdPP2C35/45	F	0.15	0.66	0.227272727	50.8
BdPP2C36/46	A	0.31	0.74	0.418918919	56.9
BdPP2C37/44	A	0.32	1.65	0.193939394	126.9
BdPP2C55/85	F	0.12	0.84	0.142857143	64.6
BdPP2C60/72	E	0.46	1.71	0.269005848	131.5
BdPP2C64/76	H	0.28	1.03	0.27184466	79.2
BdPP2C69/32	G	0.61	1.00	0.61	76.9
BdPP2C73/78	B	0.41	0.82	0.5	63.1
BdPP2C76/81	H	0.34	1.30	0.2615384	100

### Expression profiles analysis of *BdPP2C* genes in disparate tissues and under various stress conditions

**Fig. 6:**
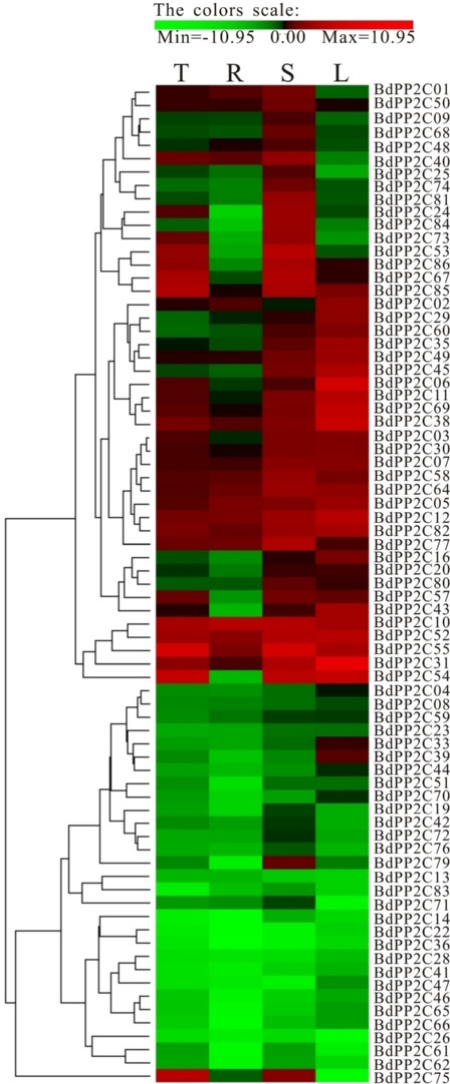
Expression profiles of *BdPP2C* genes in various tissues. The heat map shows the real-time qPCR analysis results of *BdPP2C* genes expression in roots, stems, leaves and seedlings. T, seedling; R, root; S, stem; L, leaf

To investigate the expression profile of the 86 *B. distachyon PP2C* genes under normal growth conditions, we used quantitative reverse transcription-PCR (qRT-PCR) analysis to examine their transcription levels in three different tissues: roots, stems and leaves. Most *BdPP2C* genes were observed to have a very broad expression range, and barely detectable or no expression was observed for only nine genes (*BdPP2C15*, *BdPP2C17*, *BdPP2C18*, *BdPP2C21*, *BdPP2C27*, *BdPP2C34*, *BdPP2C37*, *BdPP2C56* and *BdPP2C63*). The qRT-PCR data showed that 13 *BdPP2C* genes were highly expressed in all three tissues, while 26 displayed weak expression. Seven *BdPP2C* genes (*BdPP2C24, BdPP2C25*, *BdPP2C53*, *BdPP2C73*, *BdPP2C74*, *BdPP2C75* and *BdPP2C84*) were found to be highly expressed preferentially in stems; and two (*BdPP2C33* and *BdPP2C39*) were highly expressed in leaves. However, none of the *BdPP2C* genes was highly preferentially expressed in roots (Fig. 6). The expression profiles data of *BdPP2C* genes in various tissues are listed in Additional file 4: Table S3. We also compared the 22 duplicated *BdPP2C* gene pairs and found that the expression pattern was very similar for 6 gene pairs, i.e., *BdPP2C11/30*, *BdPP2C19/53*, *BdPP2C20/69*, *BdPP2C30/58*, *BdPP2C35/45* and *BdPP2C55/85*. On the other hand, the remaining 15 pairs showed differential expression patterns. Recent studies suggested that there was a positive correlation of duplicated gene pairs between the degree of differential expression and their divergence time, which meant that the longer the duplication, the more obvious the differentiation of the expression [39–41]. In our analysis, we found that the divergence times for these seven duplicated gene pairs with similar expression pattern were actually more recent (50.8–86.9Mya). These results were also consistent with the expression of *AtPP2C* and *OsPP2C* paralogous gene pairs, indicating the diversification of expression between duplicated *PP2C* gene pairs.

**Fig. 7:**
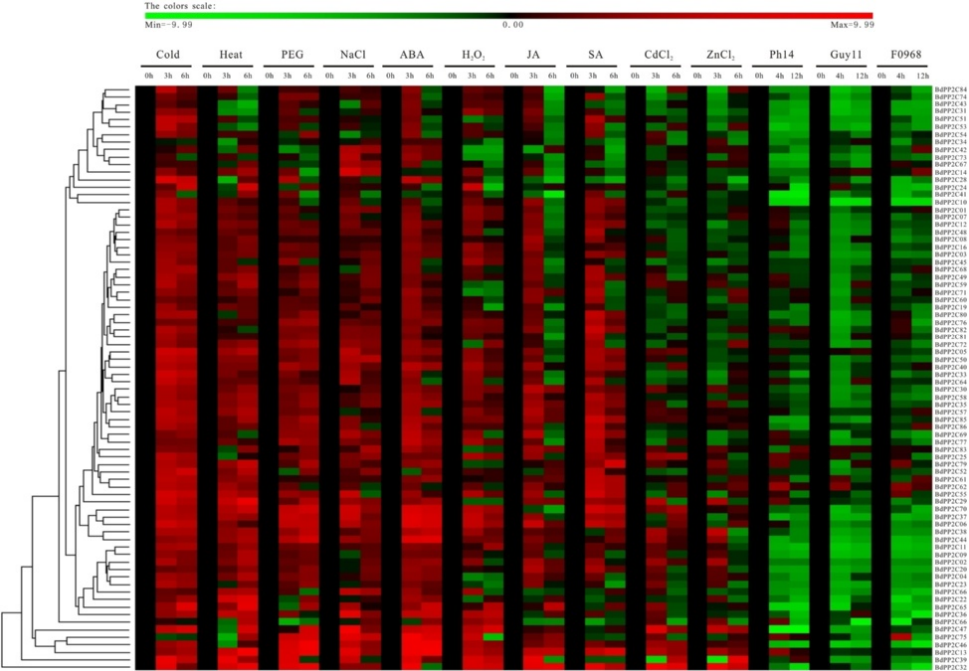
Expression profiles of *BdPP2C* genes under biotic/abiotic stresses and phytohormone treatments. Clustering of all *BdPP2C* genes according to their expression profiles under biotic/abiotic stresses and phytohormone treatments. The expression levels of genes are presented using fold-change values transformed to Log_2_ format compared with the control. The color bar represents fold-change values: green, representing down-regulation (Log_2_ ≤ -1); red, representing up-regulation (Log_2_ ≥ 1)

It has been demonstrated that subgroup A PP2Cs in *Arabidopsis*, foxtail millet and rice are transcriptionally up-regulated upon exogenous ABA treatment or stress conditions that stimulate ABA biosynthesis [23, 24, 42, 43]. Seven members of subgroup A AtPP2Cs have been characterized as negative regulators of ABA responses in ABA-mediated physiological processes [44–49]. In *B. distachyon*, subgroup A *BdPP2C*s includes eight members (*BdPP2C27*, *BdPP2C34*, *BdPP2C36*, *BdPP2C37*, *BdPP2C44*, *BdPP2C46*, *BdPP2C47* and *BdPP2C75*). The qRT-PCR result suggested that six of these genes were highly induced by exogenous ABA treatment, the exceptions being *BdPP2C27* and *BdPP2C34*. Consistently, *BdPP2C*s from subgroup A were also differentially up-regulated by drought, salt, cold, heat or H_2_O_2_ treatment. Among them, *BdPP2C36*, *BdPP2C37* and *BdPP2C44* were continuously up-regulated and remained at a high level upon all of these treatments. Both *BdPP2C46* and *BdPP2C47* were up-regulated by drought, salt, cold, or H_2_O_2_, whereas they were down-regulated by heat treatment. For *BdPP2C75*, the expression level increased under drought or salt treatment; however, cold, heat and H_2_O_2_ repressed its expression (Fig. 7). In addition, *BdPP2C70* from subgroup D, *BdPP2C13* from subgroup F and *BdPP2C32* from subgroup G also exhibited strongly increased expression levels in response to ABA and abiotic treatments (Fig. 7), suggesting that they may participate in the regulation of ABA signaling pathways. Similarly, the expression profiles of *BdPP2Cs* in other subgroups under exogenous hormone or abiotic treatments for 3 h or 6 h were analyzed as well using qRT-PCR. The other two exogenous hormones, i.e., MeJA and SA, were also tested. The heatmaps derived from expression profiles revealed that about 50–80 % of the *BdPP2C* genes were induced by various abiotic conditions or ABA treatment. On the other hand, most of the *BdPP2C* genes were up-regulated within 3 h of treatment by MeJA or SA, whereas only 15 and 26 *BdPP2C* genes maintained the increase until 6 h, respectively. In our analysis, the expression profiles of *BdPP2C* genes under abiotic stresses, including cold, heat, drought and high salinity, showed differential and overlapping expression patterns. Previous studies have suggested that different stresses can activate the same genes in distinct signaling pathways [50, 51], which might be the result of the production of some common signaling component such as ABA or calcium triggered by different stress stimuli. Increasing these signaling components leads to activation of protein kinases, including MAPK, CDPK, CCaMK and SnRK2, and then regulates the activities of transcription factors, leading to downstream responses. Moreover, such overlapping expression patterns might be the basis of crosstalk between different pathways, promoting the complex signal pathways for plants under abiotic stress conditions. Furthermore, we treated the *B. distachyon* seedlings with CdCl_2_ and ZnCl_2_ solutions, two heavy-metal stresses, and analyzed the changes in transcript abundances of *BdPP2C* genes. The expression analysis revealed that 60 % of *BdPP2C* genes shared a similar response pattern between CdCl_2_ and ZnCl_2_ treatments. For instance, the expression levels of *BdPP2C2*, *BdPP2C13*, *BdPP2C30*, *BdPP2C32*, *BdPP2C46* and *BdPP2C47* were up-regulated after 3 h and retained relatively high levels for 6 h when treated with CdCl_2_ and ZnCl_2_. However, for *BdPP2C37*, *BdPP2C39*, *BdPP2C40*, *BdPP2C43*, *BdPP2C55*, *BdPP2C69*, *BdPP2C77*, *BdPP2C82* and *BdPP2C84*, their expression was down-regulated after 3 h and then up-regulated or returned to basal levels after 6 h.

The previous study has reported that *AtPP2CG1* is induced by drought, salt or exogenous ABA treatment and thus positively regulates salt tolerance in *Arabidopsis* [52]. In this study, *BdPP2C32*, a homolog of *AtPP2CG1*, was also found to be strongly responsive to ABA and abiotic treatments (Fig. 7). Interestingly, CdCl_2_ and ZnCl_2_ treatments resulted in an obvious increase in its expression, which indicates that *PP2C* members in the G group may be related to heavy-metal-resistant pathways in plants. In addition, we also investigated the expression levels of *BdPP2C*s after phytopathogens’ infection. Bd21 seedling was infected with *F. graminearum* (F0968) and two *M. grisea*strains (Guy11, avirulent ACE1 genotype; PH14, virulent ACE1 genotype). As shown in Fig. 7, many *BdPP2C*s were significantly down-regulated at 4 h and 12 h after infection with all three phytopathogens. Only 20 *BdPP2C*s were up-regulated by a single phytopathogen within 4–12 h, such as *BdPP2C13*, *BdPP2C14*, *BdPP2C25*, *BdPP2C42* and *BdPP2C62*. It has been demonstrated that the PP2C WIN2 (At4g31750), a homolog in *Arabidopsis* with *BdPP2C13*, can enhance the resistance to phytopathogen *P. syringae* [18]. Consistent with this, the expression of *BdPP2C13* was remarkably increased after exogenous salicylic treatment and phytopathogen PH14 treatment. The expression profiles of *BdPP2C* genes under different treatments are listed in Additional file 4: Tables S4 and S5. These results indicate that PP2Cs may be involved not only in the regulation of abiotic stresses but also in biotic stresses.

As mentioned above, 22 pairs of paralogous *BdPP2C* genes involved in segmental duplication events have been found, which suggested that chromosome gene duplication may play an important role in the expansion and evolution of the *BdPP2C* gene family. It has been observed that segmentally duplicated genes displayed a greater degree of functional divergence, and they exhibited pseudo-functionalization, neo-functionalization and retention of gene functions [53]. We analyzed the expression pattern of 22 segmentally duplicated gene pairs under various stresses, which showed that 17 pairs of genes had very similar expression pattern, indicating the retention of their functions (Additional file 5: Figure S5). Most of the duplicated gene pairs were found to have a high amino acid sequence homology and share similar exon/intron structures. Although the diversification of expression occurred between duplicated *PP2C* gene pairs, their functions still were retained. This indicated that diversification of expression may occur after a very short period of time, while function diversification may need more time.

### The relevance analysis between *cis*-acting element and stress-induced *BdPP2C* gene expression

Cis-acting elements in promoter regions of genes often play key roles in stress responses in plants. For example, ABA-responsive elements (ABREs) are responsive to ABA, drought or salt signals [54]. LTR is involved in low-temperature response and regulation [55]. TCA-element and CGTCA-motif have good correlation with the expression levels after MeJA and SA treatment, respectively [56]. Analyses of cis-acting elements in the promoter regions of the 86 *BdPP2C* genes showed that every *B. distachyon PP2C* gene carried one or more ABRE, HSE, LTR, TCA-element, and CGTCA-motif in their promoter regions, suggesting the significant relationship between *BdPP2C*s and stress responses (Additional file 6: Figure S4). Among them, *BdPP2C* genes from subgroup A contained the greatest number of ABRE, which is consistent with *Arabidopsis*, foxtail millet and rice [23, 43]. Further analyses demonstrated that a good correlation existed between the number of the ABREs and the expression of *BdPP2C* in the majority of subgroups after 3 h or 6 h ABA treatment according to the statistical analysis. Similarly, this correlation was also found to exist between the number of ABREs and the expression levels after salt or drought treatment (Additional file 7: Figure S6A–F). This result indicates that *BdPP2C* genes may be involved in drought or salt stress through the ABA-dependent pathway.

## Conclusions

In the present study, beyond a comprehensive analysis of phylogenetic relationships, gene structures, conserved domains, chromosomal locations, gene duplications, *cis*-elements and expression patterns of the *PP2C* gene family was carried out for the first time in a new model monocot i.e., *B. distachyon*, one of the key features of our results was the multiple level approaches to identify BdPP2C members from sequence alignment, motifs and domain analysis to 3D protein structure confirmation (Fig. 1). Another key feature of our result was the expression patterns of the *BdPP2C* genes indicated that almost all members displayed up-regulation in response to abiotic stresses such as cold, heat, PEG and NaCl treatments, but down-regulation to biotic stresses such as Ph14, Guy11 and F0968 infection (Fig. 7).

## Methods

### Database searching and sequence retrieval

To identify *PP2C* candidates in *B. distachyon*, the InterPro domain “IPR001932” was used to search the Plaza2.5 database (http://bioinformatics.psb.ugent.be/plaza/versions/plaza_v2_5/). The resulting protein sequences were manually examined with Pfam (http://pfam.sanger.ac.uk/search) and SMART (http://smart.embl-heidelberg.de/) to confirm the presence of the PP2C domains [57]. *Arabidopsis* PP2C protein sequences were downloaded from the TAIR database (https://www.arabidopsis.org). PP2C protein sequences of rice were downloaded from the Rice Genome Annotation Project Database (https://rice.plantbiology.msu.edu/). The molecular weight (MW) and theoretical isoelectric point (pI) of the PP2C candidates were calculated using the Compute pI/MW tool in the ExPASy server (http://web.expasy.org/compute). The WoLF PSORT program (http://expasy.org/resources/search/keywords:subcellular%20location) was used to predict protein subcellular localization. The prediction of protein tertiary structure was done with the Phyre2 tool (http://www.sbg.bio.ic.ac.uk/phyre2/) [58].

### Phylogenetic analysis, exon/intron analysis and domain analysis

For phylogenetic tree construction, amino acid sequences of PP2C domains in *B. distachyon*, *Arabidopsis* and rice were aligned using the Clustal W2 program [59], and then phylogenetic trees were constructed using the ML method based on the JTT matrix-based model with MEGA 6.0 [60]. The data for the phylogenetic tree were deposited in Treebase Web (Accession URL: http://purl.org/phylo/treebase/phylows/study/TB2:S17847You). The exon/intron structures of the *PP2C* gene candidates were examined using the online Gene Structure Display Server (GSDS: http://gsds.cbi.pku.edu.ch) based on their corresponding genomic sequences [61]. The conserved domains were predicted using the InterPro database (http://www.ebi.ac.uk/interpro/).

### Chromosomal locations and synteny analysis

The chromosomal locations of PP2C genes were derived from the Plaza2.5 database. For synteny analysis, we investigated gene duplication events of *PP2C* genes in the *B. distachyon* genome and among *B. distachyon*, *Arabidopsis* and rice genomes from the Plant Genome Duplication Database (http://chibba.agtec.uga.edu/duplication/index/locus) [62]. Then the synteny blocks were illustrated with CIRCOS software (http://circos.ca/software/download/circos) [63].

### Plant materials and hormone, abiotic-and biotic-stress treatments

*B. distachyon* Bd21 was cultured in 1/4 Hogland’s solution at 25 °C/22 °C (day/night) with supplemental lighting for two weeks. Young roots, stems and leaves were harvested for tissue-specific expression analysis. Before the stress treatments, the 2-week-old seedlings were soaked in deionized water for two hours. For abiotic stress treatment, the seedlings were incubated in a solution containing 20 % PEG, 200 mM NaCl, 10 mM H_2_O_2_ or 100 μM ZnCl_2_/CdCl_2_. The cold or heat treatment was applied by incubating seedlings in a beaker containing water pre-treated to 4 °C or 42 °C, respectively. For phytohormone analysis, the seedlings were cultured in deionized water containing 100 μM MeJA, 100 μM ABA or 1 mM SA. Whole seedlings were collected from each stress treatment after 0 h, 3 h and 6 h exposure. Phytopathogen treatment was conducted by spraying 2-week-old seedlings with *Fusarium graminearum* (F0968) or two *Magnaporthe grisea* strains (Guy11, avirulent ACE1 genotype; PH14, virulent ACE1genotype) for 4 h and 12 h according to the previous report [55]. These tissues and seedlings were immediately frozen in liquid nitrogen and stored at −80 °C until used.

### Quantitative reverse transcription PCR (qRT-PCR) and cluster analysis

The total RNA was extracted from plant tissues and seedlings using RNAiso Plus (Takara, Dalian, China) according to the manufacturer’s instructions. About 1–2 μg total RNA was used to perform reverse transcription using the PrimeScript™ RT reagent kit with gDNA Eraser™ to remove genomic DNA contamination (Takara, Dalian, China). The resulting cDNA was diluted 20-fold with sterile distilled water. Quantitative PCR (qPCR) was performed in an ABI StepOne Real-Time Cycler (Applied Biosystems, USA) using 10 μL reaction volume containing 1 μL cDNA, 0.4 μL forward primer (10 μM), 0.4 μL reverse primer (10 μM), 0.2 μL ROX and 5 μL SYBR Premix Ex Taq ™II (Takara, Dalian, China). The following condition was used: pre-denaturation at 95 °C for 30 s, 40 cycles of amplification at 95 °C for 5 s and 60 °C for 30 s. Gene-specific primers was designed using the Primer5 program and their specificity was checked by blastN. The primers used for qPCR are listed in Additional file 4: Table S2 and *actin* was used as an internal control [56, 64, 65]. The data reported in this study were calculated based on three biological replicates. In addition, the expression profiles were calculated using the –ΔΔCT values [–ΔΔCT = (CTcontrol_gene_ – CTcontrol_actin_) –(CTtreated_gene_– CTtreated_actin_)], and obtained by PermutMatrixEN version 1.9.3 software, and then shown by a green-red gradient. The data were statistically analyzed using OriginPro 7.5. The differentially expressed genes were defined as those showing up-or down-regulation greater than twofold with *p-value* less than 0.05. All qPCR data were submitted to the NCBI GEO dataset with accession number GSE70366.

### Promoter analysis

The 1500 bp promoter sequences of *BdPP2C* genes were obtained from the Plaza2.5 database. The *cis*-acting regulatory elements in these promoter sequences were analyzed using the PLANT CARE program (http://bioinformatics.psb.ugent.be/webtools/plantcare/html/) [66].

### Availability of supporting data

The qRT-PCR data supporting the genes expression results of this study are available in the NCBI GEO database with accession number GSE70366. The supporting tables for phylogenetic tree were deposited in Treebase Web (URL: http://purl.org/phylo/treebase/phylows/study/TB2:S17847You).

## Additional files

Additional file 1: Figure S1. The amino acid alignment of 86 BdPP2C domains in *B. distachyon*. (TIF 9808 kb) Additional file 2: Figure S2. The predicted 3-D structure of 86 BdPP2C domains in *B. distachyon*. (TIF 8875 kb) Additional file 3: Figure S3. The predicted tertiary structure of 86 BdPP2C domains in *B. distachyon*. (TIF 7796 kb) Additional file 4: Table S1. The amino acid sequences of *PP2C* genes in *B. distachyon*, *Arabidopsis* and rice. **Table S2.** The primers used for real-time qPCR in this study. **Table S3.** The expression profiles of *BdPP2C*s in various tissues. **Table S4.** The expression profiles of *BdPP2C*s under abiotic or hormone treatment. **Table S5.** The expression profiles of *BdPP2C*s under biotic stress. **Table S6.** The orthologous *PP2C* gene pairs between *B. distachyon* and *Arabidopsis* or *B. distachyon*and rice. (DOCX 308 kb) Additional file 5: Figure S5. Expression pattern of duplication *BdPP2C* genes under biotic/abiotic stresses and phytohormone treatments. (TIF 9276 kb) Additional file 6: Figure S4.
*Cis*-acting elements analysis in promoter sequences of 86 *BdPP2C* genes. (TIF 9862 kb) Additional file 7: Figure S6. The correlation analysis between *cis*-acting elements and stress-induced expression of *BdPP2C* genes. (A) The correlation analysis between ABRE and ABA-induced expression of *BdPP2C* genes. The samples were treated with ABA for 3 h. (B) The correlation analysis between ABRE and ABA-induced expression of *BdPP2C* genes. The samples were treated with ABA for 6 h. (C) The correlation analysis between ABRE and NaCl-induced expression of *BdPP2C* genes. The samples were treated with NaCl for 3 h. (D) The correlation analysis between ABRE and NaCl-induced expression of *BdPP2C* genes. The samples were treated with NaCl for 6 h. (E) The correlation analysis between ABRE and PEG-induced expression of *BdPP2C* genes. The samples were treated with PEG for 3 h. (F) The correlation analysis between ABRE and PEG-induced expression of *BdPP2C* genes. The samples were treated with PEG for 6 h. (TIF 6349 kb)

## Abbreviations

ABRE: ABA-responsive element
AMPK: AMP-activated protein kinase
CCaMK: Ca^2+^/calmodulins-dependent protein kinases
CDPK: calcium dependentprotein kinases
CNB: cyclic nucleotide-binding domain
DSPTPs: dual-specificity phosphatases
FHA: forkhead associated domain
KAPP: kinase associated protein phosphatase
MAPK: mitogen-activated protein kinases
MW: molecular weight
Mya: million years ago
PP1: protein phosphatase 1
PP4: protein phosphatase 4
PP5: protein phosphatase 5
PP6: protein phosphatase 6
PP7: protein phosphatase 7
PP2As: protein phosphatases 2A
PP2Bs: protein phosphatases 2B
PP2Cs: protein phosphatases 2C
Pi: isoelectric point
PPM: phosphoprotein metallophosphatase
PPP: phosphor-protein phosphatase
PTPs: protein tyrosine phosphatases
qRT-PCR: quantitative reverse transcription-PCR
RLK: receptor like protein kinase
SnRK2: Nonfermenting1-related protein kinases2
S-TKc: Ser/thr kinase catalytic domain
