# Supplementary figures and images for: Genome-wide identification and evolutionary analyses of the PP2C gene family with their expression profiling in response to multiple stresses in Brachypodium distachyon
Source: BMC Genomics. 2016 Mar 3;17:175. doi: 10.1186/s12864-016-2526-4 (PMC4776448; doi:10.1186/s12864-016-2526-4)

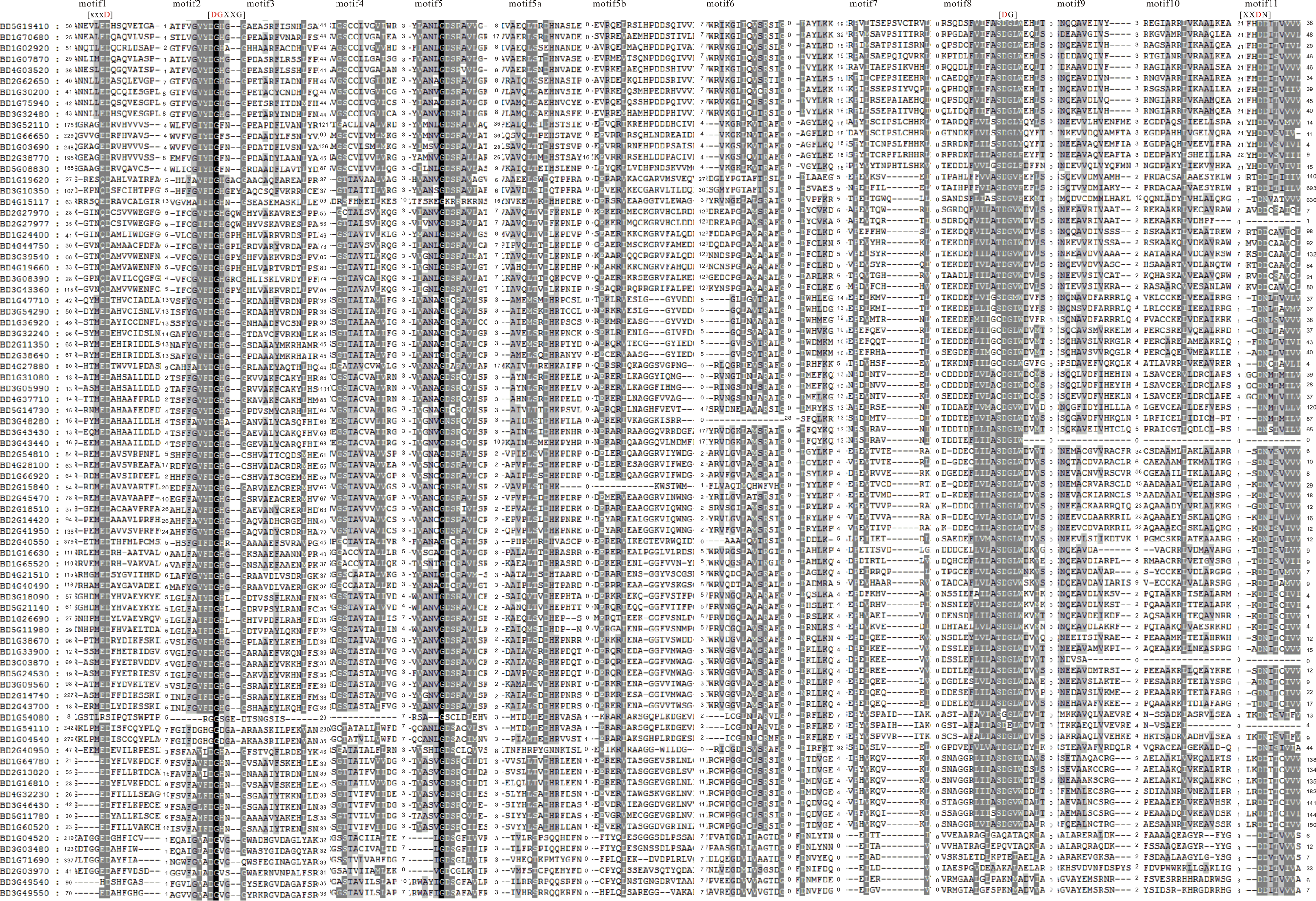

Supplement: Additional file 1: Figure S1. — The amino acid alignment of 86 BdPP2C domains in B. distachyon. (TIF 9808 kb) [file 12864_2016_2526_MOESM1_ESM.tif]

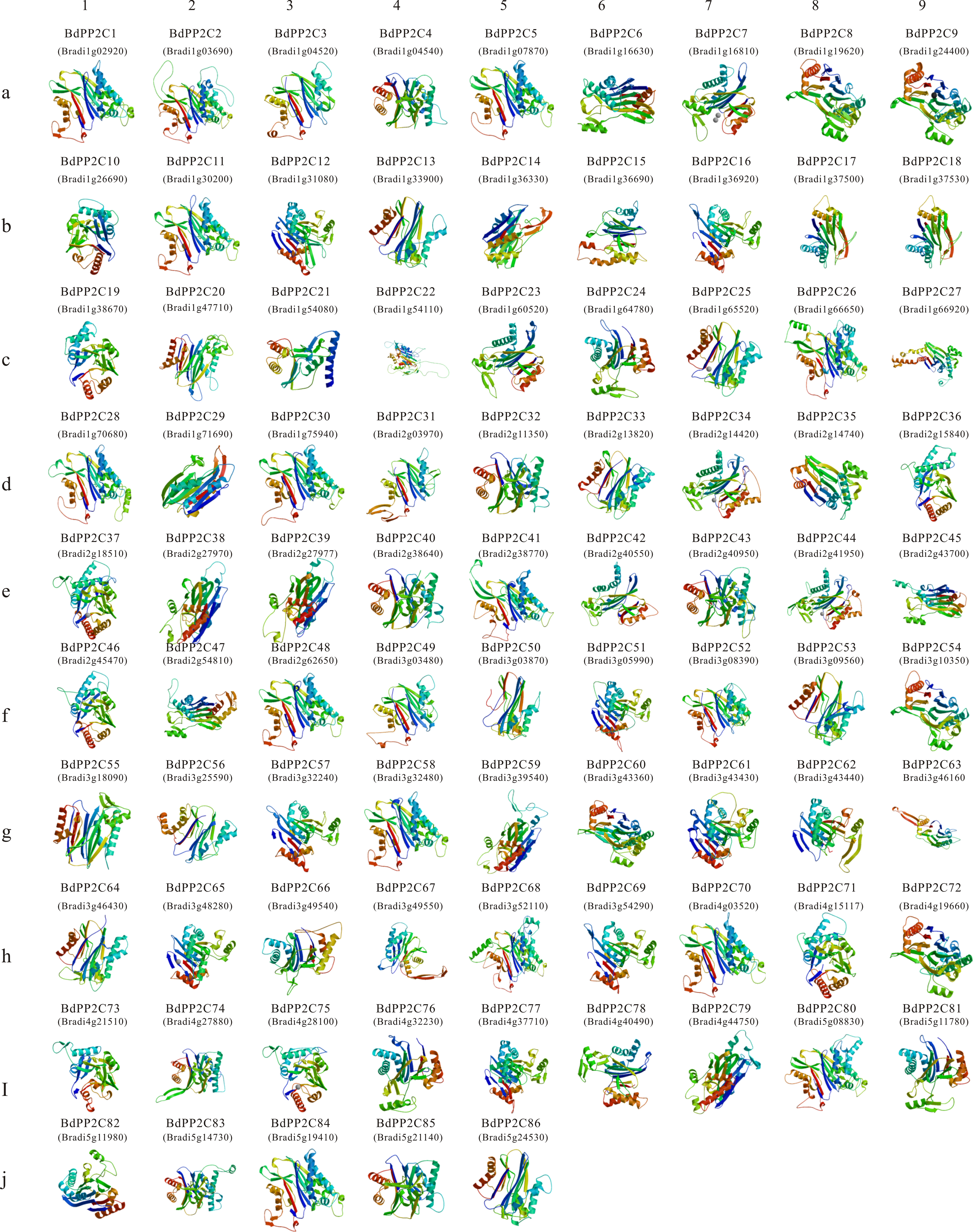

Supplement: Additional file 2: Figure S2. — The predicted 3-D structure of 86 BdPP2C domains in B. distachyon. (TIF 8875 kb) [file 12864_2016_2526_MOESM2_ESM.tif]

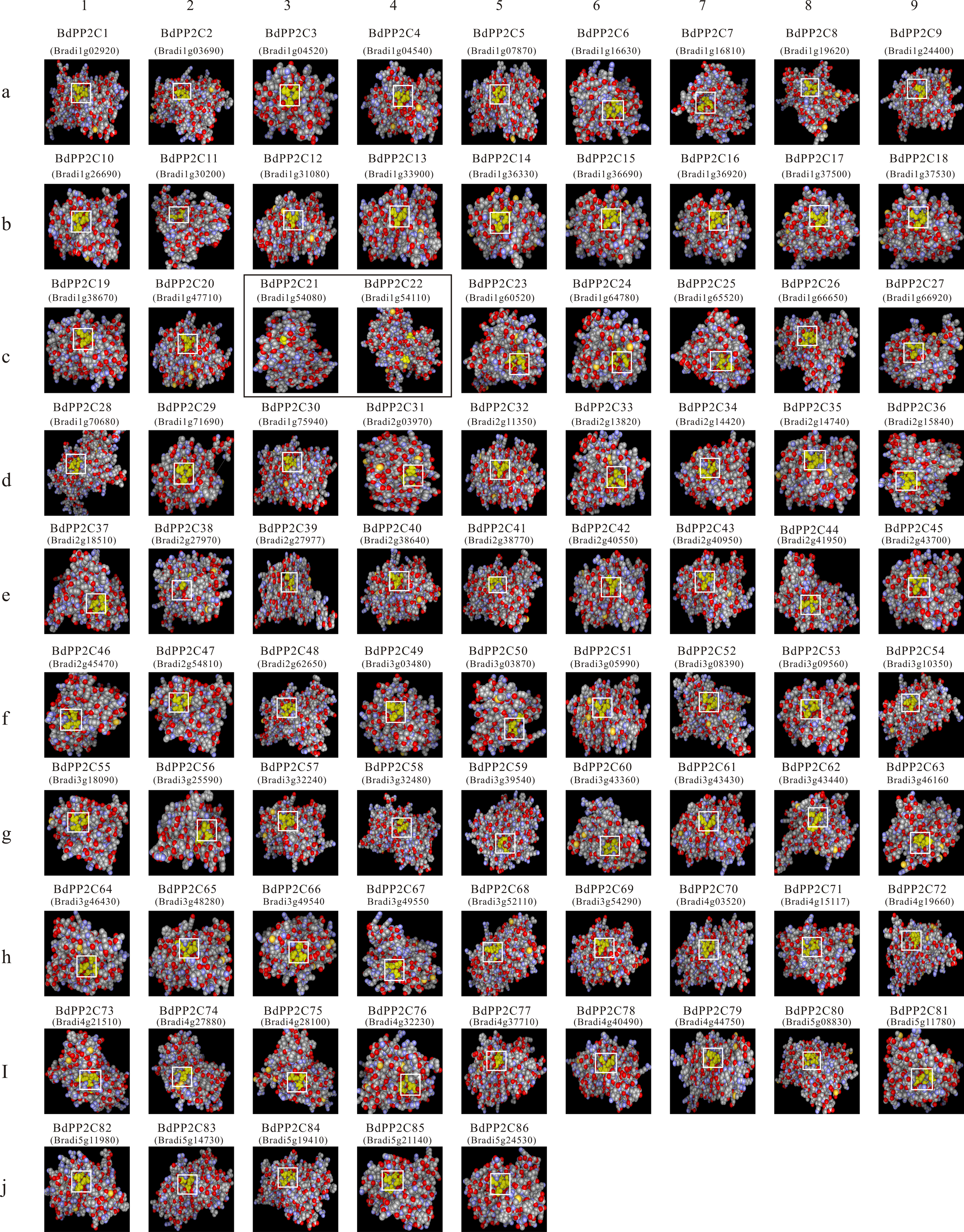

Supplement: Additional file 3: Figure S3. — The predicted tertiary structure of 86 BdPP2C domains in B. distachyon. (TIF 7796 kb) [file 12864_2016_2526_MOESM3_ESM.tif]

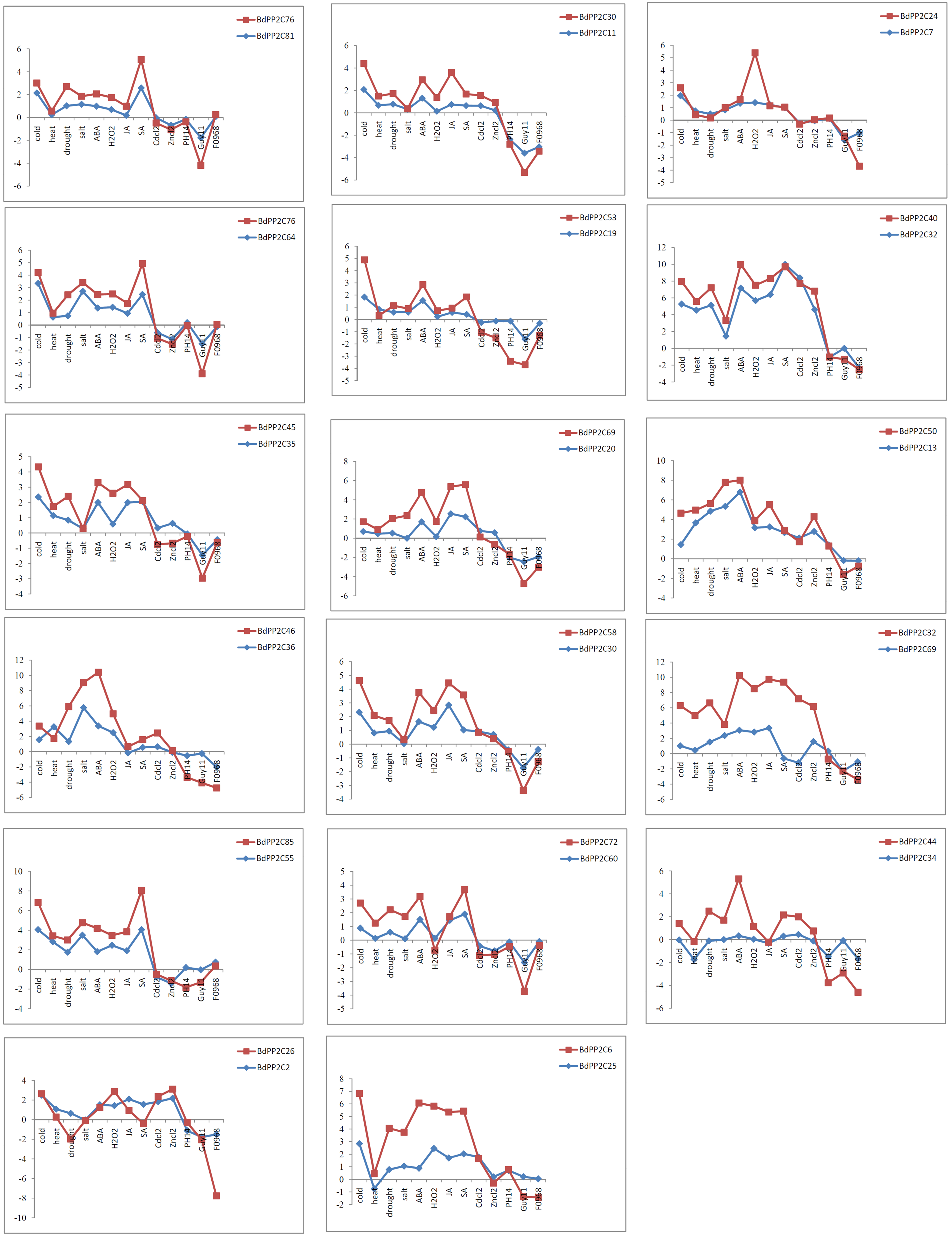

Supplement: Additional file 5: Figure S5. — Expression pattern of duplication BdPP2C genes under biotic/abiotic stresses and phytohormone treatments. (TIF 9276 kb) [file 12864_2016_2526_MOESM5_ESM.tif]
