# Supplementary material for: Genome-wide identification and evolutionary analyses of the PP2C gene family with their expression profiling in response to multiple stresses in Brachypodium distachyon
Source: BMC Genomics. 2016 Mar 3;17:175. doi: 10.1186/s12864-016-2526-4 (PMC4776448; doi:10.1186/s12864-016-2526-4)
Supplement: Additional file 4: Table S1. — The amino acid sequences of PP2C genes in B. distachyon, Arabidopsis and rice. Table S2. The primers used for real-time qPCR in this study. Table S3. The expression profiles of BdPP2Cs in various tissues. Table S4. The expression profiles of BdPP2Cs under abiotic or hormone treatment. Table S5. The expression profiles of BdPP2Cs under biotic stress. Table S6. The orthologous PP2C gene pairs between B. distachyon and Arabidopsis or B. distachyonand rice. (DOCX 308 kb) [file 12864_2016_2526_MOESM4_ESM.docx]

**Table S1: The amino acid sequences of *PP2C*genes in *B. distachyon*, *Arabidopsis* and rice**

| Gene name | Amino acid sequences |
| --- | --- |
| Bradi1g02920 | MLGPLLRFLSACGGANWQQTSPAAAGAAAPSSAAAGDPASEGRDGLLWWRDLAGCGAGELSVALVQANQTLEDQCRLDSAPPLGTFVGVFDGHAGHHAARFACDHIVPNLREAASGPRGVTADAIRDAFAATEEEFIALVSRLWETQPDVATAGSCCLVGVVHDRTLFIANLGDSRAVLGRKAGRTGQIVAEQLSSEHNANDEAVRQELMAQHPDDPQIVALKHGVWRVKGIIQVSRSLGDAYLKDTRYNTERIKQKFRVSEPFSRPIMSATPSIISRNLQPSDCFVIFASDGLWEHLSNQEAVEIVHSNQRAGSARRLIKAALQEAARKREMRYSDLIRIDKKVRRHFHDDITVIVLFIDHNLLVKGNAQGQPLSIRCALDH |
| Bradi1g03690 | MGNSLACFCCGGGGTGAKGPRRRHVAPAALPSDPAYDEGLGHSFCYVRPEKLAPLFPDDDAYDLVPDAKTAGEESAAVVAASTTFRAISGAALSANVSTPLSTSVLLLLPDESSATTASSGFESSGSFAAVPLQPVPRSSFPSSSGPISSSLSAAPFSGGFLSGPIERGFLSGPLDPAAHLLLSGPLPASGRVIGGGAGPVPALRRSLSHGGRRIRDFTRAILARSADKFHHGSSADLGSPDPAAAAMGGDSQGLQWAQGKAGEDRVHVVVSDECGWVFVGIYDGFNGPDATDFLVSNLYAAVHRELRGLLWEQQQDHQDPPASAPSTTASDHQDQCTRRRRTRRSRPPRSSSTDVDDEQRRWKCEWEQRDSSSLKPPTQQQHPPRSYGEHDHIAVLKALARALRKTEDAYLGIADKMVGEFPELALMGSCVLSMLMKGDDMYIMSVGDSRAVLATTDGDDDLEHVSEGSFGGLSAGDCSPCLSAVQLTTDHSTSVPEEVRRIRNEHPDDPSAISKDRVKGSLKVTRAFGAGFLKQPKWNDALLEMFRIDYVGSSPYITCNPSLFHHKLSRRDRFLILSSDGLYQYFTNEEAVAQVEMFIATTPEGDPAQHLVEEVLFRAANKAGMDFHELIEIPQGDRRRYHDDVSVIVISLEGRIWRSCV |
| Bradi1g04520 | MLLAGGGGSSRGGSSHLTSHKELLLGRGGRSFLFGNTWFMLSTYPARLLHTADRRAPAAAFVAAIHRTPCVRSHGTGQGLLQRGIVMAACGYVFGRAELGAAKRQLEKDSSVGNHTSRIVAMGSVGSAARPEVSFKYRGVEYYKKVGANLKCREQWGSARTFWTSAAGPGSKLSFSVEPWTRDLSTSCVAPYSAGATERQHTLDEAVQDKQMDTASDGKSPASKALKLLSGSCYLPHPAKEATGGEDGHFICVDEQAIGVADGVGGWADHGVDAGLYAKELMSKSIGAIKDEPEGAIDPSRVLEKAFISTKARGSSTACIIALTEQGIHAVNLGDSGFIVVRDGRTVLRSPSQQHDFNFTYQLESGGGSDLPSSAEVFRYPVATGDVIIAGTDGLFDNLYTNEITTIVVEAARAGLGAQATAQKIAALARERALDKNRQSPFAAAAQEAGYRFYGGKLDDITVVVSYVTSATTL |
| Bradi1g04540 | MAISPLAVSGAAVATLAVLGLAVFACRRWRRGSAPASPPPTSSQDDDMTRPLMSDNSDDYSGPSNNIRSSNAGESRVWTNRSSTSPRTHGLVSQGMIYPTEPHPIEGEIHVIDVTNGTMEEHNLASTLKRTAEVSGKIPEMKHTRRRSGENNNGGVPVKDITIGSHLALEVIAGPSHGINRYLQSGNTSMLSMTLGRVPQNDLILKDNEVSGKHARIDWNANTLKWQLVDMGSLNGTFLNSQSINHPDVGSRRWGEPAELADGDIITLGSSSKISVQISLQNKQVPVGVGMASDPMIARRTGKKLPMEDISCCQYPLAGVKQFGLFGIFDGHGGDGAAKAASRILPENVANILSQQDTIERVLTCGNASDVLECAFALTEAALDHQYEGCTATVLLVWFDQNKDCFAQCANLGDSACIMNVDGKPIAMTEDHRVVSTTERARIAKSGHPLRDGESRICGLNLCRMFGDKFLKEQDSRFSSEPYVSPVVRITKSCSAFALIASDGLWDVISAKRAAQLVVEHKERNKDHKTSADRVADHVLSEARNLRTKDNTSVIFVDFDLMRIAP |
| Bradi1g07870 | MLRAVARCCGHWPPGAAAADGMLWQTELRPHAAGEFSMAAAQANLIMEDQAQVLASPAATLVGVYDGHGGPDASRFLRSSLFPHVQRFAKEQGGMSTEVIRRAFGAAEDEFLQQVRQAWPKRPRMAAVGSCCLLGAISGDTLFVANLGDSRAVLGRRVVGGTVAVAERLSTDHNVASEEVRMEVTSQNPDDGQIVVHTRGAWRVKGIIQVSRSIGDVYLKKQEYSMDPLFRQIGPVIALKRPALSAEPQIQVRKLKPTDLFLIFASDGLWEHLSDDDAVQIVFKNPRTGIANRLVRAALKEATKKREVSLHDLKTIEKGVRRHFHDDISVVVVYLDRHRGRRHTRVVDSSSNCTSAPVDIYSSNSGKSAQSLQAYRDSG |
| Bradi1g16630 | MACTVAPPGSPVFSPSSRPAAPAHVSFKQQQQPAPPEGSPLRLIALRAAKLRIRPADPPLLVVPAASHAPSSSSSNAKRRRPPPLLVPATAPTPLDAVAAARGMEPEVAEDSGEGFAAYCRRGKGRNRLEMEDRHAATVALGGDPDAALFAVFDGHGGKSAAEFAANNMPRIIANELLHASKSSEAVEAAVRRAYLRTDKEFSSSAAAANGGGACCVTALLLRDRRQLVVSGAGDCRAVLGRGRSPALALTTDHRASRREERERIEALPGGLVLRDSRGTWRVRGSLAVTRGIGDAHLKPWVLPDPETTSVDLGDDDCELLVLASDGLWDKVGNQEAVDACSSGLPPSVACRRLVDMAVARGSTDDISVLVVHLKRLLLHP |
| Bradi1g16810 | MAGAEREKARLPPALPLATLIGRELRAGGSERPALRYGHAGFAKRGEDYFLVKPDCLRVPGDPSTAFSVFAVFDGHNGVSAAVFSKEHLLEHVMSALPPDIGGREEWLQALPRALVAGFVKADIEFQRKGEVSGTTATLVVVDGFTVTVASVGDSRCILDTQGGELQLLTVDHRLEENVEERERVTASGGEVGRLNLFGGQEVGPLRCWPGGLCLSRSIGDMDVGEYIVPVPHVKQVKLSNVGGRLIIASDGIWDTLSNEAAAKSCRGLPAELAAKLVVKQALKKCGLKDDTTCVVVDIIPSDRLVSPQLSPKRNQNKLKSLLFGRRSHSSVGKFGGKSASFGSVEELFEEGSAMLEERLGRNLSLKAASPPSRCAICQADQEPFEGLMAENGGSHCSSPYTPWGGPYLCLECRKKKDAMEGKRSSCSTACR |
| Bradi1g19620 | MGCAQGKCCGGGGEGVAEREGVGGVPGGGRGGGGATTLGRAAVPGAGLVLEYATLAVDGLYPDAPGRESQDAHLVATRFAGDPDLHLFAVFDGHGACGAACAQFAREALPRLLLLPRLAADPAGAFREAMTAANEEMHAAGGVDDSMSGTTAVAALVAGGALHVANVGDSRAVAGVWRDGRVAAEELSWDQTPFRADERARVKACGARVMSVEQVEGVRDPDAEGWLADEGDPPRVWARDGLYPGTAFTRSLGDLAAEGVGVIAEPEVKSVEITPAHLFFVVASDGVFEFLSSQEVVDMVAMHKDPRDACSAIAAESYKLWLEHENRTDDITIIIVHIRDAENSGPAGSDKANYSGTGAPIALHTVQSGLPTFVPSEVYHLNGGAATELPQSSSGSPSERRLSCVAPSPTHLLLGGDKASEASRPTQTERANDASPPLLLHLHTFCSEAVNHQKYSSRHKPRELHRKQWKLDFKVR |
| Bradi1g24400 | MGNCVPSGDTAVTASVAEDGKRRRRRWKAPREDQLGTVPGRIFANDGRSRTASVFTQQGRKGINQDAMLIWDGFGGEDDGVLCGVFDGHGPHGHLVARRVRDSLPLRLMSAARASAKNGQDMTDAAWRKAFARAFKAMDKDLRSHPSLDSFCSGSTAVTVLKLGSDLYMANIGDSRAVLGSRDAIAGGMVAVQLTVDLKPDVPSEAERIKKCKGRVFALQDEPEVPRVWLPFDDAPGLAMARAFGDFCLKDYGVISVPEFFHWSLTEKDQFVILASDGVWDVLSNQQAVDIVSSSPSRSKAAKTLVEAATREWKTKYPTSRTDDCAVICLYLDGKMDHERDSTASMDNISIDDCSVADPNEAPEQEPTLTRNFTVRTVPGSAHEKALAGVDTKLSGAADDHNWSGLDGVTRVNSLVQLPRFSEEKAIG |
| Bradi1g26690 | MAGKEIYHKMKDKVKDAFSSSGPETGKGKTKLSGKRVKHGYHLVKGKSNHPMEDYLVAEYRQVGEHDLGLFAIFDGHLGHTVPDFLRAHLFDNILKEPEFLSDTKSAIRNAYLLTDEKILERAAELGRGGSTAVTAILISSDDSVKLVVANVGDSRAVISENGKAEQLSVDHEPSMERQTIEEKGGFVSNLPGDVPRVDGQLAVARAFGDRSLKKHLSSEPHVAEIVIDENSDFLILASDGLWKVMTNQEAVDEIKDFKDAQSAAKHLTEQAVNRRSKDDISCIVVKFHC |
| Bradi1g30200 | MIVTLMNLLRACWRPSSNRHARTGSDATGRQDGLLWYKDTGQHVNGEFSMAVVQANNLLEDQCQIESGPLSFLDSGPYGTFVGVYDGHGGPETACYCNDHLFQNLKRFASEQNSMSADVLKKAYEATEDGFFSIVTKQWPVKPQIAAVGSCCLVGVICGGMLYVANVGDSRAVLGKHVKATGEVLAVQLSAEHNVSIEPVRKELQSMHPEDRHVVVLKHNVWRVKGLIQVCRSIGDAYLKKQEFNREPLYAKFRLREPFSKPILSSEPSIYVQPIQPHDQFLIFASDGLWEHLTNQEAVDIVHSSPRSGSARRLIKAALLEAAKKREMRYSDLKKIDRGVRRHFHDDITVIIVFLDSSLVSRAGTCRGPTVSLRGAGVSLRSNTLAPYGSQM |
| Bradi1g31080 | MGVYLSTPKTEKLSEDGENDKLKFGLSSMQGWRATMEDAHSALLDLDKDTSFFGVFDGHGGKVVAKFCAKYLHREVLKSEVYSVGDLGTSVHRAFFRMDEMMRGQRGWRELQALGDKINQFTGMIEGLIWSPKGSDSNDQHDDWAFEEGPHSDFSGPTCGSTACVAIVRNSQLVVANAGDSRCVISRKGQAYNLSRDHKPELEAERERILKAGGYIQMGRVNGTINLARAIGDMEFKQNKFLSPDKQMLTANPDINTVELCDDDDFLVLACDGIWDCMSSQQLVDFIHEHINTESSLSAVCERVLDRCLAPSTLGGEGCDNMTMILVQFKKPIDHEKNASASEQPSIEGKNDSAGEQLAAGNTEQP |
| Bradi1g33900 | MGHTGDGKAVCGGGFSENGKFSYGYASCLGKRSSMEDFHETRIDGVDGETVGLFGVFDGHGGARAAEFVKQNLFSNLIKHPKFFTDTKSAIAETFTRTDSELLKADTSHNRDAGSTASTAILVGDRLVVANVGDSRAVICRGGDAIAVSRDHKPDQTDERQRIEDAGGFVMWAGTWRVGGVLAVSRAFGDKLLKQYVVADPEIKEEVVDSSLEFLILASDGLWDVVTNEEAVAMVKPITDAQEAAKKLLNEASRRGSADNITCVVVRFLEQPEDTTTDKAS |
| Bradi1g36330 | METPPQVRKILEKIAASTPEPLRLAMRIHFGSLPASAAFARQDVRWYLAALTRIYEPEDLMEFEEIPLRMESAACYLLEHDEDAHFINAASNVIGVADGVGACREKGVDAAAFSRKIMENARAEVASCTPGTHLCPYGLLERAYLRAVAARTPAASTAVILSLEGRFLKWAYVGDSGFAVLRRGKIIQRSQPQQNYFNCPYQLSSNGVNKVSDAAVGEIRLKVGDVVLVGSDGLFDNVFDSALEQFVQMGAALKLSAHFLASVIAGFAYKKARSSQESPFSVDCRERTGVTINGGKKDDITVIVGYVVE |
| Bradi1g36690 | MDSLPQIRQTLEKIRARTPEPLRLAFRIHFGSLPASAAGARQDVARYLAALSNMYEPEDLMEFDEVPLKMEFASCYLPDHDEDAHFAHAEPGVIGVADGVGGCRGKGMDAAAFSRKIMENARAEVESCVPGTHICPCGLLERSYLRAVAARTPAASTAIILSLTGRFLKWAYVGDSGFAVFRRGKIIQRSQPQQNYFNCPYQLRSEGGNKISDAAVGEVRVKAGDVVVVGSDGLFDNVFDSGLERIVQMGAAVKLPPDLLANVIAEEAYVKARSSGDSPFSVSCREQTGTSCRGGKEDDITVVVAYILE |
| Bradi1g36920 | MEELRIGGGGGGRPPIPSSARRSVLSRHASFVRSPLDNTRSETGRIFENVDGEFIPVVRSGGWADIGSRSTMEDAYICCDNFLQDFGPENCEEGPSSFYGVFDGHGGNHAADFVCSNLPRFIVEDDGFPGEIEKAVSSAFLQIDAAFADACSANSSLESGTTALAALVIGRSLLVANAGDCRAVLCCRGKAIEMSRDHKPSCSREKMRIEASGGYVYDGYLNGLLNVARAIGDWHMEGVKACDGLGPLSAEPEVMTRNLTEEDEFMIIGCDGIWDVFRSQNAVDFARRRLQEHNDPVACCKELVDEAIKRKSGDNLSVVVVCFDSRPPPVLTAPRPRVQRSISAEGLRELQGFLDSLAD |
| Bradi1g37500 | MEVLPPAEILPTLAEMKARTPKPMRIAYAIRYGRLPASAAAGREDVARCVAALARTYEPDMDDLMEFPEVALKLQPGSCYLRDHDEDAHFIRADPGVIGVADGVGSWRAKGVDAAAFSRALMANARAQVDSAVPGTPVCPYKLLERAYEQTVAASTPGSSTAVIVSLSGRVLRWAYVGDSGFALFRRGRMVHRSQPQQASFNCPYQLGAWGNKVGEAAVGQIAVKDGDVLVVGSDGLFDNLFDSAIQQIVRMCGELKFSPKMVADILAGNAYCNARSNQDSPFSAASRQQQGTSFTGGKQDDITVVVAYIVS |
| Bradi1g37530 | MEVLPPAEILPTLAEMKARTPKPMRIAYAIRYGRLPASAAAGREDVARCVAALARTYEPDMDDLMEFPEVALKLQPGSCYLRDHDEDAHFIRADPGVIGVADGVGSWRAKGVDAAAFSRALMANARAQVDSAVPGTPVCPYKLLERAYEQTVAASTPGSSTAVIVSLSGRVLRWAYVGDSGFALFRRGRMVHRSQPQQASFNCPYQLGAWGNKVGEAAVGQIAVKDGDVLVVGSDGLFDNLFDSAIQQIVRMCGELKFSPKMVADILAGNAYCNARSNQDSPFSAASRQQQGTSFTGGKQDDITVVVAYIVS |
| Bradi1g38670 | MARAAASNLRGLVGIAAVDRRVVSASVQGRCAAPQASPGGRGFRAVASGSGGRTTPDSSSSSSSALRQLQPRRGAASARQSRRGVGRTGLRGLLSGGFESEDGKLSCGYSSFKGRRPTMEDRYDIKFSKIEGQTVSLFGVFDGHGGPLAAEYLKEHLLDNLMKHPQFLKDTKLAISATFLETDAVILQSVSSPYRDDGSTAIVAVLVGDHLYVANVGDSRAIVSKGGKAIPLSDDHKPNRRDERKRIENAGGTVSWDDTWRVDGVLAMSRAFGNRQLKNYVLAEPDIQEEKVNSDLEYLVLATDGLWDVVQNEEITSIVRAEDGPEAAAMKLTEIAHRWHSSDNITCIVVRFHHGKPSGINT |
| Bradi1g47710 | MCVDDAERLDFGDAGVEKPAEFPLPHMESVCENTTTADFKQSNFGNFVPIVRSGGWSDIGSRQYMEDTHVCIADLAKNFGYPEVDKEVVSFYGVFDGHGGKDAAHFVRDNLPRVIVEDADFPLELEKVVSRSFMQIDSQFADKCSHHRALSSGTTALTAMIFGRSLLVANAGDCRAVLSRCGIAMEMSMDHRPCSLTEKLRVESLGGYVDDDYLNGLLGVTRALGDWHLEGLKEVDRPGGGPLSAEPELKMVTLTKDDEFLVIGSDGMWDVFSNQNAVDFARRRLQEHNDVKLCCKEIVEEAIRRGATDNLTVVLVSFHLVAPPQIRVNRPGRVARSISVDGLNSLRILLGSQ |
| Bradi1g54080 | MPFFPVLTIAMIFVVETHVTDVRNQAYEEFHLGSTLRSIPQTSWPTPDQKHTRGGSGEDTSNGSISLKDNAHRKIFIKLDFIILLAHPCKGWHLYANFIQPFARSAGSCLDLEHVNGKMTDMTEDHRVASANKRARIARSGQPLKDGEVHLCGLNLARVLGDRFLKEQDSRFSSEPYVSPAIDIAKASTAFAVIASGGLWDVITMKKAVQLVAEVRENNGDNSSADKIASRVLSEARNLRTKDNTSVIFVGFDILRTDPCITT |
| Bradi1g54110 | MAAAAPAPLRGRLPAGRSLASRSTHTLIWNVQIAFGRFLIPAQHACTLPLQYEDLSGPLLPENLDDHSCQREIHVIDVRNETSEEIHLGSTLRSTRQTSRPSPDQKHKRGDSGEDTRNGSIPLKDNAHRSCLDLEVIAGPCQGISCSRQSTSPTLPITLGRVPPSELVLRDSEVSGKHAQINWNSKTMKWELVDMGSLNGTFLNSQAVHHPDDGSRHWGEPAELADGDIIALETSSKLSQWIQPNLLIGDLIPSLASRCGNKLPMEDISFCQYPLQGVEQFGLFGIFDGHGGDGAARAASKILPEKVANILSQHETKEKVLSCSDASDVLRYAFSLTEAAIDHQYELCHVTLAKVPDSSRWRMTAQGEFTSMFCYTALFNGSTCGDNWRPWPMGILAGQVPRPACAPGQVWHKVLSWCRSPVSLPQPANDIATWQASVVRFTLNSQKKGWFPSPCLQLGGFRNTNMAVPPMVSSHRSSDWYRPSRTMHVTGGRQERWVLGTSSRTRRSKTFLVLPSGERESAHSLPHAVHVRVMICKAQKGCTATALLIWFDQNKACFAQCANLGDSACVLSVNGKMTDMTEDHRVASANERARIARSGQPLKDGEVRLCGLNLARVLGDRFLKEQDSRFSSEPYVSPAIHIAKGSTAFAIIASDELWDVITTKKAEQLVVEVRENNCDNVSAEKIAVVY |
| Bradi1g60520 | MAVHAEADGSGVPLAVLLKRELCNQKVEKPDILFGEANKSKKGEDFTLLVAKCHRAPAEGPCDNAGSDDTISVFAIFDGHNGSAAAIYTRENLSNNVLAAIPPNLTSEEWTAALPRALVAGFVKTDKDFQTKAARSGTTVTFVIIDGWVVTVASVGDSRCILESAEGSVYYLSADHRLDANEEEVERVTASGGDVGRINIAGGAGIGPLRCWPGGLCLSRSIGDTDVGEYIVPVPHVKQVKLSNAGGRLVIASDGVWDALRFQEALNCTRGLPAESAANRIVKEAVSSKGLRDDTTCIVVDILPPEKLSPPLKRHGKGGIISLFRRRPSDELSEEQTDNGCFEPDVVEELYEEGSAMLSQRLNVNYPAGNMFKLHDCAVCQLEMKPGEGVSVHGNMPKLSRVDPWGGPFLCSSCQVKKILNLQSNLCPSSLPQRFCILKFIEANTTNKQC |
| Bradi1g64780 | MESASREERLPPALPLATLIGRELRGGGSERPLVRYGHSGFAKRGEDYFLVKPDCFRVPGDPSSAFSVFAVFDGHNGVSAAVFSKEHLLEDVMSAVPQGISREDWLQVLPRALVAGFVKTDIDFQRKGEMSGTTATLVVVDGFTVTVASVGDSRCILDTQGGVVSLLTVDHRLEENAEERERVTASGGEVSRLNLCGGQQVGPLRCWPGGLCLSRSIGDTDVGEFIVPIPHVKQVKLSNAGGRLIIASDGIWDAVSSETAAQACRGLPAELAAKLVVKQALKTSGLKDDTTCVVVDIIPSDHCSTPPPLSPNKNQNKLRSLLFGRRSHSSVGKLGNRKKSASFGFVEELFEEGSAKLEERLGRNSPSKAKFPPFRCAICQVDQVPFEDLMTDNGGGYCSAPSTPSAGPYLCSDCKKKKDAMEGKRSSPSTVCS |
| Bradi1g65520 | MSCTVAIPSSPVFSPSRRSLSCKAASASPETVSISSPAPPAPTAGSPLRPFGLLRAQIREEASPSPKTSSAAASAPAGSVLKRRRPAPLMVPVDGPAAAAAAAAAVAAVESDPRNEVEEEGDEFAVYCQRGKGRRRVEMEDRHVAKVALGGDPKVAFFAVFDGHGGNSAAEFAAENMPKFMAEEMKKVGGGDNGEIEGAVKKGYLKTDEQFLKREESGGACCVTALLQKGGLTVSNTGDCRAVLSRAGKAEALTTDHRASRDDEKERIENLGGFVVNYRGTWRVQGSLAVTRGIGDAHLKQWVVADPDTRTLLVDQHCEFLILASDGLWDKVENQEAVDIARPLCSNNEKASRMAACRRLVETGVSRGSTDDISVVIIQLHKFSASS |
| Bradi1g66650 | MGNGITKNPCFSGNPYAAAPAASDTAPEDSHGHSFTYLPMAAAFDRTPTAGSAMPSETSFFSLSGAAISANVATSASIPSFRLLNEQTWPPLSGGTFESSRSFASVPLLQAAPPRLSMSGPLLSVSSGRFSDTSAGGTGTASTTSDRFSDRPFVSGGTLDSSLSSSSFAGQHQPSVSRLIAERRAARSRRRDERSLFQYLASAASRLPGFRRPAGPKREMESLSEGGYRWPNNGNVQWAQGVVGEDRFHVAVSEEHGWVFVGIYDGFSGPDAADYLFSNLYVAVHRELKGVLWDDIQIGQPADILCSVDDGSAPEAVERKAKKGRTDNADANASASASFAGTAMATHRSVLQALARALRKTEDAFFEAAEENAEENPEVGLMGSCVLVMLMKGTDVYVMNVGDSRAVLATRREPDLENILGKASQDLKQFRQEIMRELQAQDRDGLQSVQLTPEHSTAVEEEVRRIRSQHLNDREAIDKGRVKGKLNVTRAFGAGFLKDPKWNARLIKRFQIRYVGTDAYISCIPSLCHHRIGTNDKFLVLSSDGLYQYFTNKEVVDQVAMFTAEHPEGDPAHHLVGELVQRAARKHGMDYCTLLGIPRGNRREYHDDVSVIVISFEGRIWRSSV |
| Bradi1g66920 | MAEICSEVVAPAVAEGKQGAECDAGGRAARRRRMEIRRLRLAAERGADEEASRKRRKLECRREEEEDVETGPARYGVTSVCGRRRDMEDAVSIRPEFLPGHHFFGVFDGHGCSHVATSCGEMMHEIVADEALSTGLLDGDGEERWKGVMERSFARMDAKAVGSRGSSDPAPTCRCELQLPKCDHVGSTAVVAVVGPRHLVVSNCGDSRAVLCRGGAAIPLSSDHKPDRPDELERIQAAGGRVIFWDGARVFGVLAMSRAIGDSYLKPFVISDPEVRVVERKDGEDEFLILASDGLWDVVSNEVACNVVRSCVRGNAKRRDDRGRSSPTSNLSPRQSSGSGSGSSSGDEAPNDDCGGAGSESDEESCEVDKACGEAAILLTKLALARQTSDNVSVVVVNLRRRPRP |
| Bradi1g70680 | MRWLRRCCCCCFGSGGGAGDGLVWDVALKAHASGDYSVAVAQANEALEDQAQVLVSPASTLVGVYDGHGGPEAARFVNARLFSLIQEFASENGGLSAEVIKKAFGATEEEFLGMVAKSWPSQPRLMSVGSCCLVGAIEAGTLYVANLGDSRAVLGRRATAPGKANKNHKKKRVVAERLSRDHNVADESVRREVAEMHPDDSTIVLNSHGVWRIKGIIQVSRSIGDAYLKKKPDYNNASNNSSGSSNPAGLVMMQYICPFPLPRPVMSAVPSITTRRLRPGDAFVIFASDGLWEQLSDEAAVGIVSRSPRKGVAMRLVRAAQLEAARKKDIKYESIAAIEKGRRRRFHDDITVVVLFLDDRGDSRSASTGGAAEGIDGTFAPVDVFSLGPDDHQSEDPTRPVLR |
| Bradi1g71690 | MAEAPAAVGVLGSRPREVVAPARAPTPPPPRAVRELCSPTDGDKEVTGGADAAEVPEDRSVSGGASEGSAVVDADKAAVLCSEGAAELESAEPGVLDVRLEAPVARLHEQKSDCGSSGSDEAGAINNISSLVAVSPSDTSPNSETTGEIRGSCLAEGSLAEASDSRGRQREAQEKPTGVQGMLVAAGSSDGHANSELGVEVKGDFDGRHGLMQGESELRVGGGGVEADTEMGGALCDEEVNREFEASDSSTARVQEGVDRMETSLDDSEGSDGSTTQDSDTDVETESSSSSIEEQDAGYGVHVPPMEQPICEVTRESNISEVKSSDRMVSVAVSTHVLASGAAMLPHPSKVLTGGEDAYFIACNGWFGVADGVGQWSFEGINAGLYARELMDSCKKYVMDSQGAPEMRTEEVLAMAADEAQSPGSSTVLVAHFDGQVLHVSNIGDSGLLVIRNGQVHEQTKPMTYGFNFPLQIEKDVDPLRLVQNYSIDLQEGDVIVAATDGVFDNVYEQEIADVVSKSLETDLKPTEIAELLAARAKEVGKSAWGSSPFSDAALAAGYLGYSGGKLDDVTVVVSIVRKSEI |
| Bradi1g75940 | MAAVMDYFRSCWGARSRAGHRGKKGSDTAGRQDGLLWYKDAGQVATGEFSMAVVQANNLLEDQSQVESGPLSMAEPGPHGTFVGVYDGHGGPETSRFITDNMFHHLKRFATEHKCMSADVIRKAFQATEDGFLSVVSKEWSIKPQIAAVGSCCLVGVICSGTLYIANAGDSRAVLGRLVKATGEIVAMQLSAEHNVCYEEVRQELQSSHPDDPQIVVLKHNVWRVKGLIQISRSIGDVYLKRPEYNRTPLHSKFRLRETFKKPILRSEPAIAEHQIQPSDQFVIFASDGLWEHLSNQEAVDLVQSNPRNGIARRLVKAAMQEAAKKREMRYSDLKKIERGVRRHFHDDITVVVVFLDANAASKAGWSKSPSVSVRGGGVSVRANSLAPFLAPTMLSSTY |
| Bradi2g03970 | MAASTAASRLSPPRPRTASPPSTPPRRSRFSPLRAAKMEAVLSVGAHVIPHPRKAETGGEDAFFVDSDTGGVFAIADGVSGWAERNVNPALFSRELMANSSAFLKDEEVRHDPQILLMKAHAATSSVGSATVIIAMLEKNGTLKIASVGDCGLKIIRKGQVMFSTCPQEHYFDCPYQISSEAVSQTYQDALVCTVNLVEGDMIVSGSDGFFDNIFDQEILDVIAESPGVDEAAKALAELARKHSVDVNFDSPYSMEARSRGFDVPWWKKLLGAKLIGGKMDDITVIVAQVKTVVIPDDEGSGVEQEQGSELGTGAAVVSAE |
| Bradi2g11350 | MVAGPEVMHQVVPMLEASFHRCPLKGGEEVVVARVAVSPPPPVASPEADAEVELEVPDLERKAPLETLQFVPNIRSGSFADMGPRRYMEDEHIRIDDLSGHLGSLLLCPAPNAFYGVFDGHGGSDAAAYMKRHAMRLFFEDSEFPEAVEEDELFFGSVENSIRKAFLNADLALADDSVISRSSGTTALTALIFGRQLLVANAGDCRAVLCRKGTAVEMSRDHRPTYDAERQRVTECGGYIEDGYLNGVLSVTRALGDWDMKMPQGSSSPLISEPEFQQTILTEDDEFLIIGCDGIWDVMTSQHAVSLVRKGLRRHDDPERCARELAMEAKRLQTFDNLTVIVICFASELSGCLPPSDQASSRRMRSCKSLSAEALSNLRRLLESDE |
| Bradi2g13820 | MSSSSAEPAAEQHHRHAGGNSGPVPLAALIKEEARTERRAGAASSISGQEDGGVGGGVGEAPTRRPLLRYGCAAQSKKGEDFFLLRTDCARPSTSYSSPTSSHHPTFAVFAVLDGHNGNAAAIYTRDNLLNHVLSAMPRGLSRNEWLHALPRALVSGFVKTDKEFQTKGQTSGTTATFVIIDGWTITVASVGDSRCILDAQGGSVSLLTVDHRLEENVEERERVTASGGEVGRLSVVGGAEIGPLRCWPGGLCLSRSIGDIDVGEFIVPVPYVKQVKLSNAGGRLIIASDGIWDSLSSEVAAKCCRGLPAELAAKQIVKEALRTRGLKDDTTCVVVDIIPPDQTMRLPSPPKKMNKIKSLIFRKKGKVMSNKLTKQLSSVGMVEEIFEEGSAMLSERCRLGNHSSGRRTSSLFTCAICQVDLDPSEGISVHAGSKFSSSSKPWQSPFLCSDCRDKKDAMEGKRPSGVKVL |
| Bradi2g14420 | MDDDSLGAAPPPLDSNGPDPRDDDDDASASACGSPCSVASDCSTVASADLDALLLSATAANLALSSPPSLLLLDDAPSPAAAEAAATSSGGRRTSVFALDAPPLWGLESVCGRRPEMEDAAAVLPRFHRLPLSMLSAPADPGADGLDRASFRLPAHFFAVYDGHGGAQVADHCRGELHNALVRELRAAELHDDHQAADPKKRWEKAFVDCFRRVDAEVAAKAADTVGSTAVVAVVCSSHVVVANCGDSRAVLCRGKEPVPLSLDHKPNREDEYARIEALGGKVIQWNGYRVLGVLAMSRSIGDRYLKPYIIPVPEVTVVARARDDECLVLASDGLWDVLSNEEVCDAARKRILLWHKKNATAAVARGSDGGSPDPAAQAAAEYLSKLALQKGSKDNITVLVVDLKAHRKFRSKPDNNNRQTDRQ |
| Bradi2g14740 | MVYDGAVKDQQESESSRPVLSSSAAAEAEAGAVTATVVAEGTAAGEQVGEREREGEEETVVGERPGSDKRLGVRHPLKYRRFRARGKTMVEPGGIIPPSLAMDGEGAGEVEEEEASPEREVQAGVVEVEVPLAPVEMEVDEVVESPAAAAVAVGEPELDGPSVEDEEDEEISSYLLAKEAARKKGIVAAVPVRSVEVPKDQDQEKERKEKERQRERERVDEVGYMSGGCKSDDGILSCGYSSFRGKRASMEDFFDIKSSKIDDQNINLFGIFDGHGGSRAAEYLKEHLFMNLMKHPEFMKDTKLAISETYKKTDSDFLDSESNTHRDDGSTASTAVLVGNHLYVGNVGDSRAVISKAGKAIALSDDHKPNRSDERKRIESAGGIVMWAGTWRVGGVLAMSRAFGNRLLKQYVVAEPEIQEQEIDDESEFLILASDGLWDVVPNEDAVSLVKMEEEPEAAARKLTETAFARGSGDNITCIVVKFQHDKTSCSGDCSSSPPGDKA |
| Bradi2g15840 | MRQEASAGHEEEAGLARKVAAAAAGSAARRRRRIELVRHRRGDDKAAKEEEEEDGAVGRGVRWAPACLSHGAVSVIGRRRDMEDAVAVARTFLSASPDGEVSMGDAAADEGEEEDFFAVYDGHGGARVAEACRERMHVVLAEELARRRLRADAGAIGDEEDVRVRCCWKEAMAASFARVDGEVVEAAAAGRDDADVDESGSRTVGSTAVVAVVGSRRIVVANCGDSRAVLSRAGVAVPLSTDHKKWSTWMFLVAQTMQHWFVHGDYYLKPYVSAEPEVTVCDRTEQDEFLVLASDGLWDVVSNEMACRVARSCLDGRAAAAFPESVSGRTAADAAALLVELAIARGSKDNVSVVVVELTRLKSRAARARASNGRRKGAPPQLCSS |
| Bradi2g18510 | MAAAEAICAEDEAACAGGIERLDLGDGRAALVAGGKRSVYLMECEPVWGCVATPGRGGEMEDACAAVPRFADVPVRLLARRQDLEGLGLDADALRLPAHLFAVFDGHGGAEVANYCRERLHDVLSKELRRPSKDLWEMSDVDMKEHWEELFTKCFQRVDDEVSGRASRLVDGFPEPRSEPIAAENVGSTAVVVVVCSSHVVVANCGDSRIVLSRGKEPVALSIDHKPDRKDERARIEAAGGKVIQWNGSRVSGILAMSRSIGDRYLKPFVISKPEVTVVPRAKDDDCLILASDGLWDVVSNEEACKAARRQIQQWHKNNSVATPLSHEGDGSTDPAAQAAADYLVRLALKKGSGDNISVIVVDLKPRRKAKNNS |
| Bradi2g27970 | MRQISSMLQGLARSLAVGKEKKGGGDEKGTVLRTSGTLWGEGSETFAAVCSRRGEKGINQDCSVVWEGFGCQEDGIFCGVFDGHGQWGHYVAKAVRESLPPSLLRRWQEAVALASLIDGEKKLSDYQLDLWRQSYLAAAAAVDEELRRSRRLDAVNSGCTALSVVKQGDLLVIANVGDSRAVLATTADDGGVAAVQLTVDFKPNLPKEKERIMECKGRVHCLRDEPGVHRVWLPDREAPGLAMSRAFGDYCVKDYGVISAPEVTQWRISGRDQFVILATDGVWDVLSNEEAVRIVAATPDREKAAKRLVECAVRAWRRKRRGIAVDDCSAICLFFHSPPS |
| Bradi2g27977 | MRQISSLLQGLARSMSLGREKKGADEEENGTVLRTSGALWGEGSQTLAAACSRRGDKGTNQDCSIVWEGFGCQEDTIFCGIFDGHGQWGHYVSKAVRESLPASLLCRWQEAMALASLIDGEKKLSDCHFDLWRESYLAAAAAVDEELRRNRRLDAVNSGTTALSVIKQGELLVIVNVGDSRAVLATTSDDGSVAAVQLTVDFKPNLPQEKERIMQCKGRVHCLDDEPGVHRVWLPDREAPGLAMSRAFGDYCVKAYGVISAPEVTQRTISDRDQFVILATDGVWDVISNEEAVRIVAATVDREKAAKRLVDHPFM |
| Bradi2g38640 | MVAEAEVMHQQPAQALEVQQYHRCVAKGVGMSAVAAPEVGVEVEVGVAVEVPLMGLASVDGATSVSAETIPFVPIIRSGSFADIGPRRYMEDEHIRIDDLSAHLGSLLVCPVPSAFYGVFDGHGGPDAAAYMKRHAIRFLFEDREFPQASQVDDIFLQAAENCIRSAFLQADLALADDLVISRSSGTTALTALVFGRQLLVANTGDCRAVLCRRGIAMELSQDHRANYVEECERVAASGGYIEDGYLNGVLSVTRALGDWDMKMPDCSTSPLIAEPEFRHATLSEGDEFLIMGCDGIWDVMTSQHAVSVVRQGLRQHDDPERCAQELVMEAKLLETADNLTVIVVCFASELGSPPPAPAAARPRSCKGLSAEALCNLRSWLETDR |
| Bradi2g38770 | MGNRATRLATPCFAAGGGSADDVDGGAINGGGIGHILSFDGGEDGPAALLGGATIHGVLLPSNQSTLGASSSSSVLNADHLSGGSSSSNSFSFRTLDYCPSPSSSSGTTMASSGVLSRQAARTDEQILADLYATRRRRRCQQLEESAPKNLLHRLRRALASLPLLPRVPSKKKTQQHAIADTTNGSGAIESNGCDEEDDRRAQWARGEAGEDRVHVVVSSSSETEKKEMFVGIYDGFNGPDAADYLAANLYAAIDEHTTSLMSEREVLDGMARALRRTEEAYFAAAEARAAECPELGMAGSCVLVVLVRGADVYAMNVGDSRALLARRDLPGAGAKEIRRRFDGAADGGGDLVAVQLTMDHSTSAYKVFDRSSNINCHSELYKEVRRIRSEHLDDPACIVNGRVKGSLQVTRAFGAGYLKEPRWNDALLEVFRVDYVGSSPYITCRPFLRHHRLRPRDKFLILASDGLFEYFTNEEAVAQVEAFTARYPDEDPAKYLSHEILLRAANQAGMEFNELLEVQHGDDRRRYHDDVSVIIISLDGKIWRS |
| Bradi2g40550 | MGLEVPPEESNRCVRGCCRSAAIPLHLPAASFSLLSPIARGSESTVYEARLGVERAAAKKPVLSTSDDLDKFHYQLQLLCELDHPGLAKLIAAHARPPNYLMFFEFFEPPNLADKIHVQEWSPSILQVVTIASYLAKTLQYLQILGIVHRDIKPANILLDKDLLPHLADFGLAMYKKDIKSVSVENWKSSGKPTGGFYKKNMVGTLIYMAPEILRKDLHTEKSDVYSFAISVNELLTGVVPYTDLRAEAQAHTVLEMTYTEQQLTSAVVSQGLRPALALPESGSPPSFLSLIQRCWDPDPEQRPSFEEIIEELNVIQKHLVTCSSPSSSRLVNKSQNGNTEVHHYQEALNWFNQGELLTKKENKLDSTVNPWSAYFGQSCISIYRPTLRWGSFATCGRRETMEDTHFMLPCMSEEKDVHSFGIFDGHRGAAAAEFSVRAVPGLLRQFAHTTSPTDALAEAFVRSDMAFREELILHQKSKRITQKDWHPGCTAVTALIVRNKLFVANAGDCRAILSRAGKPHPMTRDHVASCPEERERVIKEGTEVRWQIDTWRVGAAALQVTRSIGDDDLKPAVTALPEIIETDLSAEDEFLVMASDGLWDVVSNEEVLSIIKDTVKEPGMCSKRLATEAAARGSKDNITVIVVFLRPVSTAERIY |
| Bradi2g40950 | MALLSPRVPRLPLASHSAGAGALRCCGRGAASAARCHATAAGSVAAGSPSSSGLEAIRWGSAKLQGAREEMEDEVILRPESLLHGFSFAAVLDGHAGFSTVQFLRDELYKECAAALDGGAVLNTKNLEAITASIRRAFATVDANLSTWLEQMDKEDDSGATATALFLRNDVLVVSHIGDSCLQVVSRGGRPQSLTNFHRPYGNNKTSLEEIKRIRAAGGWILDGRICGDISVSRAFGDIRFKTRKNEMLVKGVKEGRWTEKFISRINFKGDLIISSPDVSLVELGPDVEFVLVATDGLWDYIKSTEAVAFVRDQLRQHGDVQRACEALGEKALDQRSQDNISIVIADLGRTNWKEMTVPRPNVFLELSQAVATVGAVSVGIWISSLLTLQ |
| Bradi2g41950 | MEDVAVAALAAAPAPVFSPATAGLTLIAAAAADPIAAVVVGAMEGVPVTLSVPPVRTTSAMEDEGLPAGGEEGEASAVGSPCSVTSDCSSVASADFEGVGLGFFGAGVEGGAVVFEDSAASAATVEAEARVAAGAKSVFAVECVPLWGFTSICGRRPEMEDAVVSVPRFFGLPLWMLTGNTIVDGLDPISFRLPAHFFGVYDGHGGAQVADYCRDRLHAALVEELNRIEGSVSGANLGAVEFKKQWEKAFVDCFSRVDDEIAGKTGRGAGGGVGTSGIAAAAVADPVAPETVGSTAVVAVICSSHIIVANCGDSRAVLCRGKQPVPLSVDHKPNREDEYARIEAEGGKVIQWNGYRVFGVLAMSRSIGDRYLKPWIIPVPEVTIVPRAKDDECLILASDGLWDVMSNEEVCDVARKRILLWHKKNGVNSSSAQRSGDDSDPAAQAAAECLSKLALQKGSKDNISVIVVDLKAQRKFKNKT |
| Bradi2g43700 | MAVPKESELPEETDYISGEWKSEDGSLLCGYSSFRGRRERMEDLYDIKSSKIDANKINLFGVFDGHGGSHAAEYLKQHLFGNLLKHPAFITDTKLAISETYKKTDLDLLDAETNINRQDGSTASTAIFVGNHLYVANVGDSRAVISKSGKAIALSDDHKPDRSDERERIENAGGVVTWSGTWRVGGVLAMSRAFGDRLLKQFVVAEPEIQEQEIDDELEYLILASDGLWDVVSNEHAVAFVKEEKGPQAAARKLTDIAFARGSTDNITCIVVEFHSDKTSSSPSSADQS |
| Bradi2g45470 | MSTETTSKRDHEASLRELLAGGDKELMTVARSARRRRLELRRLGRTASAAAEDEGAKRVRPAGPLDSCSSDSSDSAKVTPEPQPVCVSHGAVSVIGRRREMEDAVAVAAPFSAVVEGDGKEEGFFAVYDGHGGSRVAEACRERMHVVLAEEVQRLRGIQQQRGSGSGRDEEEDVIAGWKEAMAACFARVDGEVGVEDEAETGEQTVGSTAVVAVVGPRRIVVANCGDSRAVLSRAGVPVPLSDDHKPDRPDEMERVEAAGGRVINWNGYRILGVLATSRSIGDYYLKPYVIAEPEVTVMDRTDKDEFLILASDGLWDVVSNEVACKIARNCLSGRAASKYPESVSGSTAADAAALLVELAMSRGSKDNISVVVVELRRLRSRTAAVIKENRR |
| Bradi2g54810 | MAEICCQEAKSTPATAAAVATVSASAAAAVASSVMDRRRRRLEMRRFRLATDLEESAAEYARAGKRQRLSRTGSGPPCPRAPGPVPQRPDFGPRYGFSSVCGRRREMEDAVSVRPNFLPGSAESHFFGVFDGHGCSHVATTCQDSMHEAVADEHSKAAGSSSEEVPAWKGVMERSFARLDEKARNWATNRSGEEPKCRCEQQMPSRCDHVGSTAVVAVVSPTQLVVGNAGDSRAVLSRAGVPIELSVDHKPDRPDELERIQAAGGRVIYWDGARVLGVLAMSRAIGDGYLKPFVTAEPEVTVTERADDDECLILASDGLWDVVTNEMACGVVRACFRSNGPPEPFAEAEAGNDGASSSSGKGVSKAESDKACSDAAMLLAKLALARRSSDNVSVVVVDLRRGS |
| Bradi2g62650 | MVGQTMMRIVRPCFKPSLPDSQVAAAGGTKDGLLWYKDAGRHACGDFSMAVVQANNLLEDASQLEVGPFVPDGPCGTFVGVYDGHGGPETARFIADNLFHHLKKFATEQQTVSADVIQKSYAATEEGFLNLVRKQWLIKPQIASVGSCCLVGIINEGVLYVANAGDSRAVLGRVEAGVRDVRAIQLSSEHNASIPAVRDELKQLHPDDSRIVVLKHNVWRVKGIIQVSRTIGDAYLKSSEFNREPLLARFRLPEPFHKPILCPEPSIEEHRLCAEDQFVIFASDGLWEHLSNQEAVDIVNCSPRNGVARRLIKAALREAAKKREMRYSDLKKIERGVRRHFHDDITVVVLFMDPGLTGRRLYGGPLLSLRGGGGGGTSTLAQKS |
| Bradi3g03480 | MSPALALRRAAAWLLRAGAGAPRAGALPGLGGARAPVLGEAGRFLGIWGGPAAGGGGGAGSWWFRCAVSSVPRPGLLVEQLLVGGVRSFATGAAPEHVSFSAAVREEGDSQSEKPAVTSDKNMLGDRSLKLLSGSCYLPHPDKEDTGGEDAHFIWDEQAIGLADGVGGWASYGIDAGQYARDIMSHAVTAIEQEPKDSIDLSRVLEKAHRNTTVKGSSTACIVALTDQGIQAISLGDSGFILIRDGCTLFRSPIQQHDFNFTYQLESGNSSDLPSAAQVFMVPAASGDVLVAGTDGLFDNLYNNEITAVVVHATRAGLEPQVTAQKIAALARQRAQDKGRQTPFSSAAQEAGYRYYGGKLDDITVVVSYITAFGTSQPTD |
| Bradi3g03870 | MHERSAGIPPTLAAAASAAARFSFLLLLLLVVAGRSSSAWSWCREGEAEEVKGCSRIMGFGGDGSPATGGGISENGRFSYGFASCAGKRASMEDFYETRVDDVDGETVGLFGVFDGHGGARAAEYVKKHLFSNLIKHPQFIADTKSAIAETFTHTDSEFLKADSSHTRDAGSTASTAILVGGRLVVANVGDSRAVVCKGGKAIAVSRDHKPDQTDERQRIEEAGGFVMWAGTWRVGGVLAVSRAFGDKLLKQYVVADPEIKEEVVDSSLEFLILASDGLWDVVTNDVSA |
| Bradi3g05990 | MGVYLSTPKTDKLSEDGQNDKLKFGLSSMQGWRASMEDAHSALLDLDNETAFFGVFDGHGGRVVAKFCAKYLHSQVLKSEAYSSGDLGTAVHRAFFRMDGMMRGQRGWRELSALGDKINKFSGMLEGLIWSPKGSELKNGLDDWVLEEYSGYEAISQPTLIILYQGPHSDFDGPTCGSTACVALVRNNQLVVANAGDSRCVISRGGQAYNLSRDHKPELVAERERVLKAGGFIHMGRINGSLNLSRAIGDMEFKQNKSLPPEKQIVTANPDINVVELCDDDDFLVLACDGIWDCMSSQQLVDFIHEYIHTESSLSAVCERVLDRCLAPSTIGGYGCDNMTMILVQFKKPVSEKQKADIGEQSAKAIEESEIQ |
| Bradi3g08390 | MGICASSERLEQQGQEADENIVYVMDEQGGGGGGASSPRKVASLFSQKGKKGPNQDAVILCQGFGMEEGVFCGVFDGHGRCGHLISKLVRDYLPFMVLSHRNALLLADADDDPVFSDASPSSSSTGCSGGSSPQHPSQLLEEWREACTNAFNAMDNELKLQAGMDCSFSGTTAVCAIKQGKDLIIANLGDSRAVLATMSGTGFLKAVQLTTDQKPCVPQEAERIKRSEGRVFALKEEPGVMRVWLPGEDCPGLAMARALGDARLKRHGVISTPQVTGHRVTAADLFIILATDGVWDVLSNEEVVSIVCATPRKQHASKAVVEAAVQRWKTKYPSSRVDDCSAVCLFLQDPRWGAAAACRKAGGGLV |
| Bradi3g09560 | MVCISSLRALVLRAAAGASRRRGSRVLCGRAVDACVTPRTRGHGWRGFRAVAGARTKMMLDSSDSDPASAVGQLQLQRRAAAAGAQPQDGGAGGYASGGWQREDGKLKCGYSSFRGKRATMEDFYDVKLTEVDGQPVSLFGVFDGHGGSRAAEYLKEHLFENLMKHPKFLTDTKLAISETYQKTDSDFLESESNAFRDDGSTASTAVLVGGHLYVANVGDSRAVISKAGKAMALSVDHKPNRTDERKRIENAGGVVIWAGTWRVGGVLAMSRAFGNRLLKPFVVAEPEIQEELVDGDLESLVLASDGLWDAVENEEAVSLAKTEDVPESAARKLTEIAYSRGSADNITCIVVQFQHDKTGG |
| Bradi3g10350 | MGCSPSKDCSCSHFKGCLHSHGCFRQTPDSPRESREKSGRGRGKTDSSASDGSSDDLEGEDDGLNQMNITRESNVGINRLSRVSSQFLPPEGSRKVRVPLGNYDLRYSYLSQRGYYPESLDKPNQDSFCIHTPFGTSPDDHFFGVFDGHGEYGAQCSQFVKRRLCENLLRDSRFRTDAVQALHSAFLATNSQLHADSLDDSMSGTTAITILVRGKTLYVANSGDSRAVIAEKRGEDIVAVDLSIDQTPFRADEVERVKECGARVLTLDQIEGLKNPDLQCWGTEENDDGDPPRLWVESGMYPGTAFTRSIGDSVAESIGVVANPEIFILELTAIHPFFVIASDGVFEFLSSQTVVDMIAKYKDPRDACAAIVAESYRLWLQYETRTDDITIILVHINGLTDVGPTQTIMKVSLQPSQQVLELAGSESPSVVSMNPNKQRSTYDLSRTRLRALESSLGNGQLWAPPSPSHRKTWEEQAHIERILHDHFLFRKLTDSQCNVLLDCMQRLEAKPGDIVVQQGGEGDCFYVVGSGEFEVLAIQEEDGKEMTKVLHRYTADKQSSFGELALMYNKPLQSSVRAVTSGTLWALKREDFRGILMSEFSNISSLKLLRSVELFTKLTVVQLSQLAESLVEVSFADGQVIVEKDDEVSNLYIIQRGRVRLIVAADQLNSDAWDLLSAHTKQVQQSQENGNYVVEIDEGGHFGEWTLIGETIAFTAISVGDVICSTIAKEKFDLTVGSLPKLSQPDSKLKDSLIPKEHQHSAEEDFSFRRVHLSDLEWKTCIYAADCSEIGLVQVRGSDKIKSFKRFYIKRVKDLRKEAQVFQEKDIMKSLSQTACVPEVLCTCADQSYLGILLNCCLCCSLASILSTPLSESSARFYAASVVVSLEELHQRCILYRGVSADILMLDRSGHLQIVDFRFAKKLEGERTYTVCGIADSLAPEIVLGKGHGFAADWWALGVLIYFMLQSDMPFGSWRESELEPFAKIAKGHLIMPSTFSVEVVDLITKLLVVDENARLGTNGADAVKKHPWFDGIDWKQIADGTLTVPQEISDRIASYVETLQEDLTGSPSMMTEDPADLAVPEWVKDW |
| Bradi3g18090 | MAGGARAEIQPAAASSSSSSSAPFPSAGRRGRRRPDILTILRTLVCLDSSNPDTGRGRSKLSSNKVTHGFHLVEGRSGHDMEDYHVAEYKYENDHELGLFAIYDGHLGDTVSSFLKANLFNNILKEPLFWTNPQEAIKNAYSSTNKYILENSKQLGPGGSTAVTAIIVDGTDLWVANIGDSRAVICERGSAIQVTVDHEPHTADERKRIEKQGGFVSTFPGDVPRVNGQLAVARAGDQSLKAHLSSEPDFKHVAINSSIEFAILASDGLWKVIKNQEAVDLVKSVKDPQTAAKRLTSEALARMSKDDISCIVIRFRC |
| Bradi3g25590 | MAGEGLTWPDSTTLASVLPAAAEVADMTMGRHPGPNNHPDEVQLRVQFKVATGGEDAYLIAPNGWFGVADGVGQWSFEGVNAGLYASELMDGCKKFIAENEGDAELKPEQVLSKAADEARSPGSSTVLVAHFDGQFLHASNIGDSGFLVIRNGEVFRKSKPMVYGFNFPLQIEKGVDPITLVQNYTIDLEEGDVIVTATDGLFDNVYEQEVAAIILKTLQADLKPTEMAEHLAARHEVGRSGAGRSPFSDAALAAGYLGFSGGKLDDTAVVVSIVRKSEI |
| Bradi3g32240 | MSGGVEPETPPGSAGRGGSSSGSPMARKPPRHQLTSIRHCSSSARIAAASTEFVTTLTIPSSSLPSHFLKLLWCFSFFFFFQDLDSGALSLISPTDIRPGFLPVFRSGSCANIGPKSYMEDEHVCIDSLNEHLGLRTPGIPAPGAFYGVFDGHGGTDAVCFVRKNILKFIIEDGHFPNSMEKAIKSAFVKADHAIADSHSLDSNSGTTALTALIFGRTLLVANAGDCRAVLGKRGRAVELSRDHKPNCKSEKLRIENLGGIVFDGYLNGQLSVARAIGDWHVKGSKGSISPLTAEPEFQEVRLTEEDEFLIIGCDGLWDVMTSQCAVSMVRKELMAHNDPERCSRELVQEALRRDTCDNLTAVVVCFSADPPPQIEVPRFRVRRSISMEGLHMLKGALDGNA |
| Bradi3g32480 | MLSAAMEYLRSCWGPASSPDGRSRKGADAAGRQDGLLWYKDAGQLVAGEFSMAVVQANNLLEDHSQVESGPLSTTDPDLQGTFVGVYDGHGGPETARYINDHLFNHLRGFASEQKCMSVEVIRKAFRATEEGFLSVVSNQWSVRPQLAAVGSCCLVGVICAGTLYVANVGDSRAILGRLVKGTGEVLAMQLSAEHNASFEEVRREMHAMHPDDPHIVVLKHNVWRVKGIIQITRSIGDVYLKRPEFNREPLHSKFRLPETFRRPLLSSEPAITVHQIQLTDQFIIFASDGLWEHLSNQKAVELVHSSPRNGIARRLVKAAMQEAAKKREMRYSDLKKIDRGVRRHFHDDITVVVVFFDSNAVAADNWSRPTVSLRGGGVTLPANSLAPFSVPSSF |
| Bradi3g39540 | MGSCLSTQPGDGAAWPQRWRKRRGDEREGAAAAGGGGFGFFSSGGGGGGGKKLPGGGEMTEEELARVAGRTCANGASAVACLHTQQGRKGTNQDAMVVWENFNSSDSVFCGVFDGHGPYGHFVAKKVRDSLPVKLLTQWKTSGNGGTSPQLNGSISASLNSEETASAIDDEWGESVDVDGSDMLPEMFVPLKQSYLKAFKLMDKELKLHPTIDCFCSGSTAVTLVKQGWDLVVGNLGDSRAIMATRDASNHLTAVQLTVDLKPNLPKEAARIQQCRGRVFALQDEPEVSRVWLPNNDSPGLAMARAFGDFCLKDYGLISVPEISYRRLTEKDEFIILATDGVWDVLSNKEAVDVVAAAPSRATAARALVDCAVRSWRLKFPTSKSDDCAAVCLFLDHGNSPDSVEENEAKNGEEPAVEASIPDASVYENTADVNVHSSSQEQIPEPTLEHSSTLRNVDEIMPVDEPPVLKEPERCASARSLADCISTNEEEEWSALEGVTRVNSLLNLPRKLSGDKRSTSWRKRR |
| Bradi3g43360 | MVEAAAGRRSGTSRRRPSGGGEQQRLVAVALAAHVVMVTSAPAAGAAGEGGGGAGAGGRCLEDLLGCLLGVLRALGVPWAAPRPQRQPRPVPPRGGAVPAPADVRRFAAELRRIPGRIASNGACSVASLYTMQGKKGVNQDAMVVWENFCSRDDTIFCGVFDGHGPYGHLVAKRVRDLLPVKLGADLGTDEARETSTTNMKSNTNQAGLPVNPERTKTTSTTSTGAEQNGEHPEIFTTLRTSFLRAFHIMDRDLKLHKNIDCFFSGTTAVAVLKQGHNLIIGNLGDSRAVLGTRNEDHQLIAVQLTVDLKPNIPSEAQRIRQRRGRIFALPEEPEVARVWLPKYNSPGLAMARAFGDFCLKEYGLISMPDVFCHHVTEKDEFIVLATDGVWDVLSNTEVVSIVKRATSRASAARCVVESANLAWRTRYPTSKVDDCAVVCLFLNTHDANETSSSAANNLASDVEVSGDQHSTSVQLSTGVSDAVTALVKDKDELSVIDAVAKPATISDLTNDVSGAKESIM |
| Bradi3g43430 | MGAAASLPVTSKFSTAGENDSIKYGTSSMQGWREQMEDAHAAILDLDGSQSTSFFGVYDGHGGAEVALYCAKQFHVELVNDPDYVNNPAAAMEHVFFRVDEQLHQSDEWRVLANPRGYSYLMRCLRTSLCAAWPLKARYIGPQDEGSTACVAIIRGNQIIVGNVGDSRCVLSRNGQAINLSIDHKPNHRNERARIRAAGGQVRRDGFAKIQEGRVVATEWGVYRVDGKLAMSRAIGIVLSGFLFAGCTQSVSLLMGILAAYISGDFQYKQNKTLAPAEQMVTCNPSIRAVNITDDTDFLLIASDGIWDVMTSQKAVEIVHTCLQNGMTDPRAICGTLQDLCLRSEDNSTVILVGFKDGARIGSPPPPPGLEPEDAPSDIADTSGEARAGSNNEIVEAKAQPSLFFIAESSKQGFSEQE |
| Bradi3g43440 | MGAAGSLPSLPVTSKLSTAGENDSIKYATSSMQGWPEEMEDAHAAILDLDGSQSTSFFGVYDGHGGGEVALYCARQFHIELVNDPDYGNNPATAMEHVFFRIDEQLQQSDEWRELANPRGYCYLMRCLRTSLCAAWPVKARYIGPQYEGSTACVAIIRGNQIIVGNVGDSRCVLSRNGQSLYPFHPKAINLSMEHKPYHRNEKARIQAAGGQVLMDMFPKVLAGQVVGTELGIYRVDGKLAMSRAIGDFQYKQNKTLPRAEQMVTCNPSIRAVNITDDTEFLIIASDGIW |
| Bradi3g46160 | METSTHEQEDPMDCDEAPLRMVPASCYMPDHDEDAHFIHAASGVIGVADGVGGCRGLCADAAAFSRGLMAHAHALLASSSSSSPQPVCPYTLLDRAYHHTVDSLSRTPTLAASTAVILSLSGAVLRFAYVGDSGFAVFRGGRILHRSRPQQSYFNCPYQLSAHGTGGNRVRDAAVGQVPVAAGDVVVAGSDGLFDNLFDSGMERIVQLGAALRFPARTMADFMASHAYSKARSRTEDSPFSAACREQGVVGSVGGKMDDITVVVAYIE |
| Bradi3g46430 | MSTRSKSATGSVGGGSGGGSTAGAGGSTVPLAVLLRREVVSERTAAERPELQHGLFSQAKKGEDFTFLKPECERHPGVPSSSFSAFGLFDGHNGSGAAIYTKENLLNNILSALPADLNREDWLAALPRAMVAAFVKTDKDFQTKARSSGTTVTFVIIDGLFVTVASVGDSRCVLEAEGSIYHLSADHRFDASKEEVDRVIEAGGDVGKLNVVGGAEIGPLRCWPGGLCLSRSIGDQDVGQFIVPVPLVKQVKLSTAGGRLIIASDGVWDALTAEVALNCSRGLPPEAAAEKIVKEAVHSKGLRDDTTCIVVDLVPEKGNLAMAPPKRQPGMGVFKNMFRKKTCSDSSSHSDREYMDPDIVEEIFEDECAFLSRRLDSEYPVRNMFKLFICAICQVELKPNQGISVHEDSSQPGNLRRWDGPFLCQSCQEKKEAMEGKRRSRDSSSRNSGSSE |
| Bradi3g48280 | MGQSRSRSLPVTSKLSVRQENDRIKYAVSSMQGWRPYMEDAHAAILDLHDSKSTSFFAVYDGHAGANVALYCASQFHIELMHHEDYHNNLAHAVERTFFRIDEQLQQLDGWREAFKPPLVKAFNLLNCLKPPACDKGTPDTEGSTACVVLIRGNQIIVGNVGNSRCVLSRDGQAIDLSTDHKPTLAAERERIVKAGGKISRRSFQLKRNKDLTPEEQMVTCSPDIMTVDITDDTEFLVIASDGLWDYVSSQGAVDFVHKQLNSGIRDLRFICELLIDICMRTQDNMTMILVQFKHAPRVPPPASDVHVVPPPAGNVPVVDPAFIPSSSTNPIPAISAGGAGAKARADTCVTEITEVKEEEDKGSSGGATEEESLLPLS |
| Bradi3g49540 | MGTPSLLDRLLNGLDSASRRLYVAASRTIARRATTTTTTMETLEQIKPTMAEIDPRIPDALRVAFRLGGHRLSSPGANDNEGFASFVAAVLPAPPVPDCPAVVPAPRAARALRLDVGSCYLPHHDHDSHFGASDFGVLGVADGVGGYSERGVDAGAFSRGLMTSAFAAVVSAPPGAPVCPYTLLELAYEETAASAAPGASTAVILSLAPAADAEESPRLRWAYIGDSGFAVLRRGKILRRSRPQQSRFNCPYQLNSTGNGDRVTAAETGEVPVEEGDVVVAGTDGLFDNMFDEELERVVRMGAALGLPAKNMADVIAGVAYEMSRNRARDSPFSVESRRHHRADRWSGGKEDDITVVVAFVALSY |
| Bradi3g49550 | METLEPIQQTLREIDKRVPYSLRAAFGLAHRPVALPSDDGDIASFVASFFQPQDDGPGPAETRGNDEEEEPRARAGLRMDWACYARDHDEDAHFGHGEAGVVGVADGVGGYRKRGVDAGAFSRGLMTAAFAEVCAAEPGTPVCPHTLLERAYEDTAASGAPGGSTAVILSLAPGGTDNTLRWAFIGDSAFAVFRGGRIVHRSRRQQKRFNHPLQLSAREGGVAKADVGEMAVREGDVVVVGTDGLFDNVFDGEIGVVVRMGTALGFSPKNMADVVAGVAYEMSRSNERDSPYSIDSRKHRGDRRHGGKPDDITVVVAFIVSAYS |
| Bradi3g52110 | MGSGASRLLTACTCSRPAPASVDAEPCLDDALGHSFCYASAAAYSSSFRHGISGAALSANSSVPVPLYLSDSAAGAANMPPNYSSAFQTSSSFSSAPLQLSNLNSGPLFLSGPIDRGAQLSGPLDAAVPFSGPLPAKPAKHASSSSRALSRRFRKPLFGSLRRSVSEKHRPLTAPLPRDDGVQWAHGRAGEDRVHVVVSEDQRWLFVGIYDGFNGPEAPDFLVANLYRFLLRERGIFYEEAERDSKRLWQFLADGDDDDSELDFSGSGRFALSLARLKERRFSMWAQAAAVGDDEINREWGPKKLAAAPAVRDHGAVLGALTRALAATEAAYLDMTDQSMGSHPELAVTGACLLVALVRDDDVYVMNLGDSRAIVAQRVDDDHGCSLGTMRTDDAGLGLEIESRPVGFAMIGPEALQLSIDHSTSIEEEVQRIKREHPDDDHCIVNDRVKGRLKVTRAFGAGYLKQAKLNNGLLEMFRNEYIGDAPYISCIPSLCHHKLTARDQFLVLSSDGLYQYLSNEEVVLHVENFMERFPEGDPAQSLIEELLSRAAKKAGMDFHELLDIPQGDRRKYHDDVTVMVVSLEGRIWKSSGTYV |
| Bradi3g54290 | MCVEGFEDAERLDFGAAEIERPADFLMERVCENMVSLDFKQTKMVSGTGESWHNFVPAIRSGEWSDIGGRDYMEDAHVCISNLVKNFGCNKADDEIISFYGVFDGHGGKDAAHYVRDNLPRIIVEDADFPLELEKVVRRSFVQTDSQFAEKCSRHDALSSGTTALTAMIFGRSLLVANAGDCRAVLSRRGAAIEMSKDHRTCCLNERKRVESLGGYVDDGYLNGQLAVTRALGDWHLDGLKEMGEPGGPLSAEPELKMITLTKEDEFLLIGSDGIWDYFSNQNSVDFARRRLQEHNDLRLCCKEIIEEAIRRGATDNLTAVMVSFHQEAPPQIRVNRTGRVERTISAEGLHSLRVLLEGK |
| Bradi4g03520 | MLRWLARPAERCLGRSGCGCGGGGGAGGGGDGLLWNAELKTHASGQYSMAVAQANESLEDQGQVATSPAATFVGVYDGHGGPEASRFLSSHLFPHLHKFASEQGGVSNDAIKKAFHATEEEFLHLVKGSWLKRPKIAAAGSCCLVGAIANNVLYVANLGDSRVVLGHKGPNGRGVVAERLSNDHNVADEEVRKELAEQHPDDSHIVVYTKGVWRIKGIIQVSRSIGDVYLKKPEFARNPKFQHYVCPVPLKRAVITAEPSIKVHHLRQQDLFLIFASDGLWEQLTDKAAVDIVFKNPRAGIAKRLVRAALSEAARKREMKYADIQQIERGIRRHFHDDITVVVVYLDNHKRGAQPKFSNLNSFRFTNAPEDIFSGRSDQRGHQPLSGAVG |
| Bradi4g15117 | MSLTLAGHRILFLLLLLVELLAPTRCAGESATCLAVYREGGAPAVYQSAHCPRWSLHPGGEEDGEQRSSSSTPRTCHVAARRGRRRSQEDRAVCALGIRIPFIEGTRIKEVDVGVMAIFDGHNGSEASEMASKLLLEYFLLHVYFLLDGIYSIMFKKSTGKLTYKEVTILNNILNMYKEDQSIHRERSCWTSPAILDRSFHMEILKESLLRAVHDIDLTFSKEGKRRRKRNSSNHDDFALVNYDGPLYNVKELTKDHHPDREDERSRVEAAGGTVLEWAGVYRVNGELALSRAIGDVPFKRYGVISTPELTGWQLLSANDSFLIASSDGVFEKMTMQDVCDMMLHAKLGVNQGFETSVVAQQNLADYIVHLALQKGTTDNVATVVVPLVSASSSVATIENELHLEENSRKSVLPLHTIPYQHNSDDRVSSAVMDMEYFKHSSTKFQRFLVDAKLNSLGCFYLSESLDEDMDYIFRVPESYQHGGVRDFNHIPTENVLYSDGYLEKYKDRNFCWYLGHQDDELGRCNSPEGFANYFGLLDSVSHNGSNLNSSHSFGYNIADIRYKLKKRFDRGSYGEVWLAFRWNCSDDIDAHKNPSHFSTILTPDSYNCTSSNTTSSYEDNVSDIIDGDLFILKRIMVERGNAAYLSGLREKYFGELFSNASKTLEELSRMESSSTAFPVDMQFVQYTFPEQNMSAIEESLKHVARFIESFESESKEIWLVYRNEGRSLSKLIYAAEETKLVTGDDNERVRHIQVLQPSKWWYWLRTTKAGQKQMQNLLWQLLMGLKACHDRNITHRDIKPENMIICFEDLETAKCLREIPSEAKENKLNMRLIDFGSAIDDFTLKHLYGSGPTRSEQTFEYTPPEALLNSSWFQGSKTARLKYDIWSVGVVMLELIVGSPHVFQISDRARVLMDQRLEGWSEQTKELAYKLRSYMELCILVPGISSQHQGSGNSERGHAGLASWKCSEESFAHQVKIRDPLKMGFPNLWALRLARQLLVWHQEDRLTVDEALNHPYFQEPP |
| Bradi4g19660 | MGSCLPADQRPLDGTALLGKGGGCRRREEEAPGRIAGNGAGNAACLFTRQGKKGTNQDAMVAWENFNGRSDTVFCGVFDGHGPHGHLVARTVRDTLPSKLCDLIYHDYGESPTSNQDGSVIEEILSPYADADNKSPTAAGQKEEQRELFDSMKESFRKAFRVTDKELKLHRNIDSICSGTTAVTLIKQGQDLIVGNLGDSRAVLGTRDQNGRLVAHQLTVDLKPDHPREARRIKRCNGRVFAHQDEPDVARLWLPNCNSPGLAMARAFGDFCLKDFGLICVPEVSYRQITEKDEFIVLATDGVWDVLSNQEVVDVVASCSGHSAAARTVVDLANQTWKFKYPTSKTDDCAAVCLFLNKDAEAGELSGHSVANKGTGSGPRMPPRLKNPRYKSKKFIPEDAEDECDSNISGDERSLEGFTRLNTLLVLPKFGDTSPTKK |
| Bradi4g21510 | MASLLCSPTLSVPAASPLSLRRGNVSKVHPIQCFDQEQLLMVPTALSMDVFGACNVGNDIKVEQQQVEKTKKKAAWAAKRRPSMLVIPVSAPPEAGQAVVAGWGVAAAVAEKEAEAEVEGEGFWVASRRGARHGMEDSYGVITHKDGADSQLAFYGVYDGHGGRAAVDLVSDRLGKNVVSAVLAATEATHDAVTAAIRAAYVATDSEFLRQGVRGGSCAATALVKGGDLYVANLGDCRAVMSLDGAATALTSDHTAARDDERARIENSGGYVSCGSNGVWRVQDCLAVSRAFGDAGLKQWVISDPEIRRQPLTPGCEFLVLASDGLWNKVSNQEAVDAVARSRRSSSYCCKELVDLARGRGSRDDITVMVVDLERFLR |
| Bradi4g27880 | MAHQKREANSTDEYWTSKRLKGAATSIEKDYNVEAAASQETNAEKRETSQKESTMPVDPCMLDEKATMISKVSSQQDMIVTCVEADAAEDKGCRHTMEDTWVVLPDASMESPGNLRCAHFAIYDGHGGRLAAEYAQTHLHQNVIAAGLPRELMDVKVAKKAIIEGFRKTDESLLQESTRGNWQDGATAVCVWVLGQTVVVANAGDAKAVLARSTSTDGEGSLVDTKSLMKAIVLTREHKAIFPQERSRIQKAGGSVGPNGRLQGRIEVSRAFGDRHFKKVGLIATPDVHSFEVTKKDNFIILGCDGLWGVFGPSDAVEFVQKQLKETSSATLAVRRLVKEAVRERRCKDNCTAVLIVFKH |
| Bradi4g28100 | MSMAQVCCDTAVVVGAEAEARARARAGRRRRAAGDAATGRWKVAPEVPQGAEEAAAATRKRRAAGAEAGAAKRHGVASVAGRRREMEDAVSVREAFAAAPAEEEEEGKEPGKAGRDFYGVFDGHGCSHVADACRDRMHELVAEELPGAGASPDSWTTAMERSFSRMDAEVMAAGGRERDDSASCRCEAHKCDHVGSTAVVAVVEARRVVVSNCGDSRAVLCRDGAPVPLSSDHKPDRPDELERIESAGGRVIFWEGARVLGVLAMSRAIGDGYLKPFVSAVPEVTVTERLDGDECLILASDGLWDVVSNQTACDVARACLRRGRDRWCAEAAAMLTKMALTKGSSDNISVVVVDLRPRNPLPL |
| Bradi4g32230 | MAMAAEAGAAMTVPLGVLLRREVTSERMERPDVLCGEATRSRKGEDFTLLLSEAGQRVAGDPSTSFSVFALFDGHNGSGAATYTKKNLLDNVLRATPSGLSRDEWLAVLPRALVAAFVKTDKDLQALAETSGTTVTFVIIDEWVVTVASVGDSRCILESADGSLYHLSADHRFDSNRDEVERVTAWGSKVGKLNVIGGPEVGPLRCWPGGLCLSRSIGDMDVGECIIAVPHVKQVKLSNAGGRIIIASDGVWDDLTFEMALECSRGYPSDIAANRIVNEAILPRGLRDDTTCIVVDILPPEKLAPSPPTKWQGKFQGKVVLNNMFRRKHPNVSFKIDREYAEPDVVEEIFEDGSPMLSKRLTTGYALQNMFEPSSCAVCQLRLRAHQGISIHANPLQHEKLQGWQGPFLCQSCHEKKEAMEGKRRPIDSSTADVFGHVVQA |
| Bradi4g37710 | MGTHLSTPKTEKYCADGENDQLRYGLAAMQGWRTTMEDAHAAFPRLDDCTSFFGVYDGHGGKAVAKFCAKHLHMQVLRNEEYSSGDLATSVQKAFFRMDEMMKGQRGWRELAELGDKGQKFAGMLEGIIWSPKGGDSDKLGDDWAEEGPHSDFSGPTCGSTACVAIIRNDQLIVANAGDSRCVISRKGQAHNLSRDHKPELDTEKERILNAGGFVVAGRVNGSLNLARAIGDMELKGNENLPAEKQIVSAEPEVNTVKLSEDDEFIVLACDGIWDCMSSQEVVDFVHEKLNTEDSLSAVCEKLLDRCLAPESGGEGCDNMTVILVQLNKPRKSAATSSADQSAATTEEIRPNEPDDPKSPSQ |
| Bradi4g40490 | MLHSVKFLCSVARLVHALLMSLCSPRTPPAPLQQVQAIIKATKVLRPASMAVADHDEKPRPPPVLVLPPALFKQGEKGAKARGRPSRLVIPPPPVAGRDAGFDPFGEAAAADRVAAEVEEQGDGFCVASRRGVRHAMEDAYGAVADEIRGESRMAFYGVYDGHGGRAAVDLVAERLGKNVVAAAATASPGDELGVMAAIRQGYLTTDNEFLSQGLRGGCCAATALLKDGDLYVANAGDCRAVLGTRSGAAIPLTSDHTPARDDERRRIEAAGGYVSKGSGGVWRVQDTLAVSRALGDADMRASGVTGVPEVHAARRVTADCAFLVLASDGVWSKVSDQEAVDAVIARISSCTEKTTASVECCKALVALARSRGSRDDITAMVVDLQRFLLPR |
| Bradi4g44750 | MGICCSSRAGGGRREELEAAEGWFPWKHDDFLLQEPQFAGVSMHTKQGWKGVNQDAMAACPDFAGRKGQIFCGVFDGHGPLGRDVARYVRDALPAKLSSSLALPPKTEEDAPSSDADLDSFDKSDSTSFSDTSDENRLLSSWRSAIVKAFEDVDEELSQHSGIDCICSGTTAVSVVRQGDHLIIANLGDSRAVLCARDSKDRPIPVQLTTDLKPDLPGEAERIMSCKGRVFAMEDEPDVPRLWLPDQDAPGLAMARAFGDFCLKNHGLICTPEVYHRKLTEKDDFLVLATDGIWDVLSNKEVVKIVSSAADRSKAAKQLVDKAVRAWRRKFPTSMVDDCAAVCLFLKPIVSSDDNSNTIIKPPNASTLSFTGSFRKAMGGGEAEEGPAVWRALEGVARVNSVVRLPRIGAVLSWRRRSVSLDQEDDVGQESSSKIA |
| Bradi5g08830 | MVDELFYEGNNDHNISSEEEDTLVRSCSNLSVSFGYHCNSYQSFPLDNDEHDNSPQMRFESNTMMKSRNGSFTCLSGAGISANFTLANTNICKGLIGEEILPELDSPNSFRKIVSSPSMSRLDLLSTSQGSPASAESSIFEISKNIWRSSAPTTVSSNFLTNTEVKMAGGAAGEDRVQAVCSEKNGWLICGIYDGFNGRDAADFLAVTLYDNIVYYLYLLECRIKQQDGLYNSSEGSLNGVKSELTLAMRIAENEDVKLSENFRAGVLNCLTAAVEQAENDFLCMVEQDMDDRPDLVSVGSCVLVVLLQGTDLCILNLGDSRAVLASMPYAEMNTVKAIQLTEIHSLENPLEYQKLLVDHPNDSKVVMGNKVKGKLKVTRAFGVGYLKQKKFNDALMGILRVRNLCSPPYVYTNPHTLSHKVTEDDLFVVLGSDGLFDFFNNDEVVQLVYQFMNGNPNGDPAKYLIEKVVHKAAKEAALTAEQLMRIPVGSRRKYHDDVTVIVIILGNARRTVSASTSV |
| Bradi5g11780 | MAAAVAEATTPRRRRESVALGDLLQREASAERAEAAGGERPGVAAGQAGRARKGEDYALLKLSCERYPGASFSAFAMFDGHNGAAAAVYAKEHLLSNVLGCVPADLSRDEWLAALPRALVAGFVKTDKDFQTRAHSSGTTVTLVIIDGSVVTAASVGDSRCVLEAEGSIYYLSADHRFDASGEEVGRVMECGGEVGRLNVIGGAEIGPLRCWPGGLCLSRSIGDQDVGEYIIPVPFVKQIKLSSAGGRLIISSDGVWDALTAEMALSCSRGLPPEAAADQIVKEAVDSKGLRDDTTCIVIDIIPPRKPKSRLRSSKKARNGFSLLKNVFFRKTISDKLSNANKEHTSGPDLVEEVFEDGCPSLLRWLDSEYPVRNMFKLFICAICQVELQSGQGISIHEGLSKPGKLCPWDGPFLCHSCQEKKEAMEGKRPSRDSSSRNSGSSE |
| Bradi5g11980 | MAKFCCFAAGCSERHASATSGKGKGCQGEVKVSYGFSLVRGKTNHPMEDFHVAELTDAKGNELGLFAIFDGHLGDTVPAYLQKNLFPNILNEEEIWTHPDIAITKAYEKTDQSILSHTPDLGPGGSTAVTAILINGKKLWVANVGDSRAVLLKRGEAIQMSIDHDPNVERGAIENRGGFVSNMPGDVPRVCGQLAVSRAFGDRNLKSLLKSEPDVKVEDIDHTAELLVLASDGLWKVMNNQEAVDLAKRFKDPQTAAKQLVAESRKRDSKDDISCIVVRFKM |
| Bradi5g14730 | MADDNQPRQPVTTKTTQRGENDRLEYAVSSMQGYRRNMEDAHAAFEDFDVPTATSFFGVYDGHGGPDVSMYCARHLHLEIRKHPEFTNNLPTAVDGAFSRMDQMMTTDEGRRELTRYWDRKLTLKDMLLRCACFEDHPGPIEVGSTACVALIRGNQIIVGNAGDCRCVLSRNRQAIVLTTDHKPSVLAERQRILNAGHFVEVTQGVSRVDNEIAVARSIGDMRYKSNIALPPALQALTCAPEIRSENITDDAEFLVMACDGVWDVVDNQGFIDYIHLLLAAVPAMNLGEVCEALLDEFVERSRDNMTVLLVRFKHNAQAPDVPEDELPGVQQEDQSTDDELPGVQQEDQSTDDELPGVQQDQSTDDELPGVQQEDQSTDDELPGVQQEDQNTNDELPGAQQEDQSTDDELQSKKMKAPFELELSAGASCSR |
| Bradi5g19410 | MWPWLEKIASACWDRVRRYTLTRRDEEDGGGSGSGSGADAVDDDLLWSRDLARHAAGEFSFAVVQANEVLEDHSQVETGAAATFVGVYDGHGGAEASRFISNHLSAHLVRIAQQSGTISEDVVRNAFSATEEGFLSLVRRTHLIKPSIASIGSCCLVGVIWRKTLYLANLGDSRAVVGCLTGANKIVAEQLTRDHNASLEEVRQELRSLHPDDSQIVVLKNGVWRIKGIIQVSRSIGDAYLKKKEFAIDPSITRFHLSEPLRRPVLTSEPSVCTRVLRSQDSFVIFASDGLWEHLTNQQAVEIVYNNPREGIARRLVKAALKEAARKREMRYNDIAKLEKGVRRFFHDDITVVVVFIDHELLQEGNASAPELSVRGFVESGGPSSFSGLNSMS |
| Bradi5g21140 | MVGRMERQTASTSCSHSAAGSSSSSSCGGKKRPDILNMIRSATCLHSSSTDTGKGRSKLSSTKVAHGFHLVEGKSGHDMEDYHVAEYKYEKNHELGLFAIFDGHLGDRVPSYLRANLFCNILKEPLFWTNPQEAIKNAYGSTNKYILENAKQLGPGGSTAVTAIVVDGKDMWIANVGDSRAVLCERGAANQLTVDHEPHTTNERQRIEQQGGFVTTFPGDVPRVNGQLAVARAFGDHSLKTHLSSEPDVRHVPINSNIEFVILASDGLWKVMKNQEAVDLVKSTKDPQAAAKRLTTEALARKSKDDISCIVIRFRC |
| Bradi5g24530 | MGYLSTVIGHPTDGSPVSGGGLSQNGKFSYGYASSLGKRASMEDFYETRIESVDGQLIGLFGVFDGHGGAKVAEYVKHNLFSHLLRHPKFMSDTKVAIDDSYKSTDSEFLESDSSQNQCGSTASTAVLVGDRLFVANVGDSRAIICRGGDAVPVSKDHKPDQTDERQRIEEAGGFVMWAGTWRVGGVLAVSRAFGDKLLKQYVVVDPEIREEVVDDTLEFLILASDGLWDVVSNEEAVDMTRSIQDPEEAAKRLLQEAYKRESSDNITCVVVRFFHGQGSGGPA |
| AT1G03590 | MGGCISKTSWSNEEPMHRPCLGMGCCGSKMGKRGFSDRMVSLHNLVSIPNRIIGNGKSRSSCIFTQQGRKGINQDAMIVWEDFMSKDVTFCGVFDGHGPHGHLVARKVRDSLPVKLLSLLNSIKSKQNGPIGTRASKSDSLEAEKEESTEEDKLNFLWEEAFLKSFNAMDKELRSHPNLECFCSGCTAVTIIKQGSNLYMGNIGDSRAILGSKDSNDSMIAVQLTVDLKPDLPREAERIKQCKGRVFALQDEPEVSRVWLPFDNAPGLAMARAFGDFCLKDYGVISIPEFSHRVLTDRDQFIVLASDGVWDVLSNEEVVEVVASATSRASAARLVVDSAVREWKLKYPTSKMDDCAVVCLFLDGRMDSETSDNEEQCFSSATNAVESDESQGAEPCLQRNVTVRSLSTDQENNSYGKVIAEADNAEKEKTREGEQNWSGL EGVTRVNSLVQLPRFPGEEP KT |
| AT1G07160 | MSSSVAVCNSPVFSPSSSLFCNKPLNTSPAHETLTLSLSHLNPPVSSTSPSAASPTSPFCLRLLKPPAKLGFGSDSGPGSILKRKRPTTLDIPVAPVGIAAPISNADTPREESRAVEREGDGYSVYCKRGKREAMEDRFSAITNLQGDPKQAIFGVYDGHGGPTAAEFAAKNLCSNILGEIVGGRNESKIEEAVKRGYLATDSEFLKEKNVKGGSCCVTALISDGNLVVANAGDCRAVLSVGGFAEALTSDHRPSRDDERNRIESSGGYVDTFNSVWRIQGSLAVSRGIGDAHLKQWIISEPEINILRINPQHEFLILASDGLWDKVSNQEAVDIARPFCKGTDQKRKPLLACKKLVDLSVSRGSLDDISVMLIQLCHLF |
| AT1G07430 | MADICYEDETSACESRPLWSSRKWRIGVQRFRMSPSEMNPTASTTEEEDKSEGIYNKRNKQEEYDFMNCASSSPSQSSPEEESVSLEDSDVSISDGNSSVNDVAVIPSKKTVKETDLRPRYGVASVCGRRRDMEDAVALHPSFVRKQTEFSRTRWHYFGVYDGHGCSHVAARCKERLHELVQEEALSDKKEEWKKMMERSFTRMDKEVVRWGETVMSANCRCELQTPDCDAVGSTAVVSVITPEKIIVANCGDSRAVLCRNGKAVPLSTDHKPDRPDELDRIQEAGGRVIYWDGARVLGVLAMSRAIGDNYLKPYVTSEPEVTVTDRTEEDEFLILATDGLWDVVTNEAACTMVRMCLNRKSGRGRRRGETQTPGRRSEEEGKEEEEKVVGSRKNGKRGEITDKACTEASVLLTKLALAKHSSDNVSVVVIDLRRRRKRH VA |
| AT1G07630 | MGNGVTKLSICFTGGGGERLRPKDISVLLPDPLDEGLGHSFCYVRPDPTLISSSKVHSEEDTTTTTFRTISGASVSANTATPLSTSLYDPYGHIDRAAAFESTTSFSSIPLQPIPKSSGPIVLGSGPIERGFLSGPIERGFMSGPLDRVGLFSGPLDKPNSDHHHQFQRSFSHGLALRVGSRKRSLVRILRRAISKTMSRGQNSIVAPIKSVKDSDNWGIRSEKSRNLHNENLTVNSLNFSSEVSLDDDVSLENQNLQWAQGKAGEDRVHVVVSEEHGWLFVGIYDGFNGPDAPDYLLSHLYPVVHRELKGLLWDDSNVESKSQDLERSNGDESCSNQEKDETCERWWRCEWDRESQDLDRRLKEQISRRSGSDRLTNHSEVLEALSQALRKTEEAYLDTADKMLDENPELALMGSCVLVMLMKGEDIYVMNVGDSRAVLGQKSEPDYWLAKIRQDLERINEETMMNDLEGCEGDQSSLVPNLSAFQLTVDHSTNIEEEVERIRNEHPDDVTAVTNERVKGSLKVTRAFGAGFLKQPKWNNALLEMFQIDYVGKSPYINCLPSLYHHRLGSKDRFLILSSDGLYQYFTNEEAVSEVELFITLQPEGDPAQ HLVQELLFRA AKKAGMDFHELLEIPQGERRRYHDDVSIVVISLEGRMWKSCV |
| AT1G09160 | MSVSKASRTQHSLVPLATLIGRELRSEKVEKPFVKYGQAALAKKGEDYFLIKTDCERVPGDPSSAFSVFGIFDGHNGNSAAIYTKEHLLENVVSAIPQGASRDEWLQALPRALVAGFVKTDIEFQQKGETSGTTVTFVIIDGWTITVASVGDSRCILDTQGGVVSLLTVDHRLEENVEERERITASGGEVGRLNVFGGNEVGPLRCWPGGLCLSRSIGDTDVGEFIVPIPHVKQVKLPDAGGRLIIASDGIWDILSSDVAAKACRGLSADLAAKLVVKEALRTKGLKDDTTCVVVDIVPS GHLSLAPAPM KKQNPFTSFL SRKNHMDTNN KNGNKLSAVGVVEELFEEGSAVLADRLGKDLLSNTETGLLKCAVCQIDESPSEDLSSNGGSIISSASKRWEGPFLCTICKKKKDAMEGKRPSKGSVTT |
| AT1G16220 | MGLCHSKIDKTTRKETGATSTATTTVERQSSGRLRRPRDLYSGGEISEIQQVVGRLVGNGSSEIACLYTQQGKKGTNQDAMLVWENFCSRSDTVLCGVFDGHGPFGHMVSKRVRDMLPFTLSTQLKTTSGTEQSSSKNGLNSAPTCVDEEQWCELQLCEKDEKLFPEMYLPLKRALLKTCQQMDKELKMHPTINCFCSGTTSVTVIKQGKDLVVGNIGDSRAVLATRDQDNALVAVQLTIDLKPDLPSESARIHRCKGRVFALQDEPEVARVWLPNSDSPGLAMARAFGDFCLKDYGLISVPDINYHRLTERDQYIILATDGVWDVLSNKEAVDIVASAPSRDTAARAVVDTAVRAWRLKYPTSKNDDCAVVCLFLEDTSAGGTVEVSETVNHSHEESTESVTITSSKDADKKEEASTETNETVPVWEIKEEKTPESCRIESKKTTLAEC ISVKDDEEWSALEGLTRVNSLLSIPRFFSG ELRSSSWRKW L |
| AT1G17550 | MEEISPAVALTLGLANTMCDSGISSTFDISELENVTDAADMLCNQKRQRYSNGVVDCIMGSVSEEKTLSEVRSLSSDFSVTVQESEEDEPLVSDATIISEGLIVVDARSEISLPDTVETDNGRVLATAIILNETTIEQVPTAEVLIASLNHDVNMEVATSEVVIRLPEENPNVARGSRSVYELECIPLWGTISICGGRSEMEDAVRALPHFLKIPIKMLMGDHEGMSPSLPYLTSHFFGVYDGHGGAQVADYCHDRIHSALAEEIERIKEELCRRNTGEGRQVQWEKVFVDCYLKVDDEVKGKINRPVVGSSDRMVLEAVSPETVGSTAVVALVCSSHIIVSNCGDSRAVLLRGKDSMPLSVDHKPDREDEYARIEKAGGKVIQWQGARVSGVLAMSRSIGDQYLEPFVIPDPEVTFMPRAREDECLILASDGLWDVMSNQEACDFARRRILAWHKKNGALPLAERGVGEDQACQAAAEYLSKLAIQMGSKDNISIIVIDLKAQRKFKTRS |
| AT1G18030 | MLEKESDLTAMEKPNNKHAADSFSSEDLVSPVKKAKKSEEVSGGGEAVAAVGNREAEEDKPSFVSEEKKEFLVEADVAEDKGARHTMEDVWVVLPDASLDFPGTLRCAHFAIYDGHGGRLAAEFAKKHLHLNVLSAGLPRELLDVKVAKKAILEGFRKTDELLLQKSVSGGWQDGATAVCVWILDQKVFVANIGDAKAVLARSSTTNELGNHTEAGNPLKAIVLTREHKAIYPQERSRIQKSGGVISSNGRLQGRLEVSRAFGDRHFKKFGVSATPDIHAFELTERENFMILGCDGLWEVFGPSDAVGFVQKLLKEGLHVSTVSRRLVKEAVKERRCKDNCTAIVIVFKRV |
| AT1G22280 | MGKFCCFTSASEVVGGQSSSRSGKGRSDEGMIKYGFSLVKGKANHPMEDYHVANFINIQDHELGLFAIYDGHMGDSVPAYLQKRLFSNILKEVKTKKKGEFWVDPRRSIAKAYEKTDQAILSNSSDLGRGGSTAVTAILINGRKLWIANVGDSRAVLSHG GAITQMSTDHEPRTERSSIEDRGGFVSNLPGDVPRVNGQLAVSRAFGDKGLKTHLSSEPDIKEATVDSQTDVLLLASDGIWKVMTNEEAMEIARRVKDPQKAAKELTAEALRRESKDDISCVVVRFR |
| AT1G34750 | MAKLCCFGSSDYDLVVGRASTSSGKGRNNDGEIKFGYSLVKGKANHPMEDYHVSKFVKIDGNELGLFAIYDGHLGERVPAYLQKHLFSNILKEEQFRYDPQRSIIAAYEKTDQAILSHSSDLGRGGSTAVTAILMNGRRLWVANVGDSRA VLSQGGQAIQMTIDHEPHTERLSIEGKGGFVSNMPGDVPRVNGQLAVSRAFGDKSLKTHLRSDPDVKDSSIDDHTDVLVLASDGLWKVMANQEAIDIARRIKDPLKAAKELTTEALRRDSKDDISCIVVRLR |
| AT1G43900 | MKKTRNVASSPIECVHLQTKPTTTLVRSFFFFLFNSQTISSFIIFYLFLCSFFWFCQSPNLTNPSPPPLSVAPLRGDANSPPPESSSSPATKSSLMISSRDPNALFSGGGISFLAGVRTVKFSYGYSSLKGKRATMEDYFETRISDVNGQMVAFFGVFDGHGGARTAEYLKNNLFKNLVSHDDFISDTKKAIVEVFKQTDEEYLIEEAGQPKNAGSTAATAFLIGDKLIVANVGDSRVVASRNGSAVPLSDDHKPDRSDERQRIEDAGGFIIWAGTWRVGGILAVSRAFGDKQLKPYVIAEPEIQEEDISTLEFIVVASDGLWNVLSNKDAVAIVRDISDAETAARKLVQEGYARGSCDNITCIVVRFEVS |
| AT1G47380 | MSTKGEHHTVPLSVLLKRESANEKIDNPELIHGQHNQSKKGEDFTLVKTECQRVMGDGVTTFSVFGLFDGHNGSAAAIYTKENLLNNVLAAIPSDLNRDEWVAALPRALVAGFVKTDKDFQERARTSGTTVTFVIVEGWVVSVASVGDSRCILEPAEGGVYYLSADHRLEINEEERDRVTASGGEVGRLNTGGGTEIGPLRCWPGGLCLSRSIGDLDVGEYIVPVPYVKQVKLSSAGGRLIISSDGVWDAISAEEALDCCRGLPPESSAEHIVKEAVGKKGIRDDTTCIVVDILPLEKPAASVPPPKKQG KGMLKSMFKRKTSDSSSNIEKEYAEPDVVEELFEEGSAMLSERLDTKYPLCNMFKLFMCA VCQVEVKPGEGVSIHAGSDNCRKLRPWDGPFLCASCQDKKDAMEGKRSSG DRHSSESD |
| AT1G48040 | MILSQTMVAEAEIRVLDVKCHISAPKDQKNFQIDEVRVSESVRAEISGSAETPRFGSGMSCVTTTIGESASDFIPTIRSGSFADIRSRETMEDEHICIDDLSAHLGSYNFSVPSAFYGVFDGHGGPEAAIFMKENLTRLFFQDAVFPEMPSIVDAFFLEELENSHRKAFALADLAMADETIVSGSCGTTALTALIIGRHLLVANAGDCRAVLCRRGVAVDMSFDHRSTYEPERRRIEDLGGYFEDGYLNGVLAVTRAIGDWELKNPFTDSSSPLISDPEIGQIILTEDDEFLILACDGIWDVLSSQNAVS NVRQGLRRHG DPRQCAMELG KEAARLQSSD NMTVIVICFSSVPSSPKQPQ RRRLRFCVSD EARARLQAML AGE |
| AT1G67820 | MTNKLRSEETITSLSSFMASTLSIASPSPCSIPLSVTKVSPLKRKRPTHLNIPDLNPQQPISTDYFRFREGDAKVSPLKRKRPAHLNIPDLNPQQPIRTDYFSFTDFAHQNGTVSFGGNGFGVVSRNGKKKFMEDTHRIVPCLVGNSKKSFFGVYDGHGGAKAAEFVAENLHKYVVEMMENCKGKEEKVEAFKAAFLRTDRDFLEKGVVSGACCVTAVIQDQEMIVSNLGDCRAVLCRAGVAEALTDDHKPGRDDEKERIESQGGYVDNHQGAWRVQGILAVSRSIGDAHLKWVVAEPETRVLELEQDMEFLVLASDGLWDVVSNQEAVYTVLHVLAQRKTPKESEEENLVQGFVNMSPSSKLRRASLVKSPRCAKSQSYYYNSENESPSLNREIGSSPSKSPITPWKSLWAKAACKELANLAAKRGSMDDITVVIIDLNHYKG |
| AT1G68410 | MASREGKRRNHNHDDEKLVPLAALISRETKAAKMEKPIVRFGQAAQSRKGEDYVLIKTDSLRVPSNSSTAFSVFAVFDGHNGKAAAVYTRENLLNHVISALPSGLSRDEWLHALPRALVSGFVKTDKEFQSRGETSGTTATFVIVDGWTVTVACVGDSRCILDTKGGSVSNLTVDHRLEDNTEERERVTASGGEVGRLSIVGGVEIGPLRCWPGGLCLSRSIGDMDVGEFIVPVPFVKQVKLSNLGGRLIIASDGIWDALSSEVAAKTCRGLSAELAARQVVKEALRRRGLKDDTTCIVVDIIPPENFQE PPPSPPKKHN NFFKSLLFRK KSNSSNKLSK KLSTVGIVEELFEEGSAMLA ERLGSGDCSK ESTTGGGIFTCAICQLDLAPSEGISVHAGSIFSTSLKPWQGPFLCTDCRD KKDAMEGKRPSGVKVI |
| AT1G72770 | MEEMTPAVAMTLSLAANTMCESSPVEITQLKNVTDAADLLSDSENQSFCNGGTECTMEDVSELEEVGEQDLLKTLSDTRSGSSNVFDEDDVLSVVEDNSAVISEGLLVVDAGSELSLSNTAMEIDNGRVLATAIIVGESSIEQVPTAEVLIAGVNQDTNTSEVVIRLPDENSNHLVKGRSVYELDCIPLWGTVSIQGNRSEMEDAFAVSPHFLKLPIKMLMGDHEGMSPSLTHLTGHFFGVYDGHGGHKVADYCRDRLHFALAEEIERIKDELCKRNTGEGRQVQWDKVFTSCFLTVDGEIEGKIGRAVVGSSDKVLEAVASETVGSTAVVALVCSSHIVVSNCGDSRAVLFRGKEAMPLSVDHKPDREDEYARIENAGGKVIQWQGARVFGVLAMSRSIGDRYLKPYVIPEPEVTFMPRSREDECLILASDGLWDVMNNQEVCEIARRRILMWHKKNGAPPLAERGKGIDPACQAAADYLSMLALQKGSKDNISIIVIDLKAQRKFKTRT |
| AT1G78200 | MPKICCSRSATQVVVAQKSNSGKGRNGEGGIKYGFSLIKGKSNHSMEDYHVAKFTNFNGNELGLFAIFDGHKGDHVAAYLQKHLFSNILKDGEFLVDPRRAIAKAYENTDQKILADNRTDLESGGSTAVTAILINGKALWIANVGDSRAIVSSRGKAKQMSVDHDPDDDT ERSMIESKGGFVTNRPGDVPRVNGLLAVSRVFGDKNLKAYLNSEPEIKDVTIDSHTDFLILASDGISKVMSNQEAVDVAKKLKDPKEAARQVVAEALKRNSKDDISCIVVRFR |
| AT1G79630 | MGLCYSVDRTTGKEPGEASSTATTAETVEERSGSGRWRRPRDLKGGGDIEGIPQVLGRLVSNGSSKIACLYTQQGKKGTNQDAMLVFENFCSRDDTVFCGVFDGHGPFGHMVAKKVRDTLPFTLLTQLKMTSESDQSSLVGANGFQIKCTEEEEVQTTESEQVQKTESVTTMDEQWCELNPNVNNDELPEMYLPLKHAMLKSCQQIDKELKMHPTIDCFCSGTTSVTLIKQGEDLVVGNIGDSRAVLATRDEDNALLAVQLTIDLKPDLPGESARIQKCKGRVFALQDEPEVARVWLPNSDSPGLAMARAFGDFCLKDYGLISVPDINYRRLTERDQFIILASDGVWDVLSNKEAVDIVASAPSRSTAARALVDTAVRSWRIKYPTSKNDDCTVVCLFLQDSSVAMEVSTNVKKDSPKEESIESVTNSTSKEEDEIVPVKDEKIPESCGIESKMMTMTLA ECISVAQDDEEWSALEGLTRVNSLLSIPRFLSGELRSTSWRKWL |
| AT2G20050 | MGCAYSKTCIGQICATKENSIRQTHQQAPSRGGTRATAAAAAVEEDNPVFNFSSDAVDDVDNDEIHQLGLSRDQEWGITRLSRVSSQFLPPDGSRVVKVPSCNYELRCSFLSQRGYYPDALDKANQDSFAIHTPFGSNSDDHFFGVFDGHGEFGAQCSQFVKRRLCENLLRHGRFRVDPAEACNSAFLTTNSQLHADLVDDSMSGTTAITVMVRGRTIYVANAGDSRAVLAEKRDGDLVAVDLSIDQTPFRPDELERVKLCGARVLTLDQIEGLKNPDVQCWGTEEDDDGDPPRLWVPNGMYPGTAFTRSIGDSIAETIGVVANPEIAVVELTPDNPFFVVASDGVFEFISSQTVVDMVAKHKDPRDACAAIVAESYRLWLQYETRTDDITIIVVHIDGLKDDAPRQLSSTGTQLQPPIPQVVELTGSESPSTFGWNSKNQRVRHDLSRARIRAIENSLENGHAWVPPSPAHRKTWEEEAHIERVLRDHFLFRKLTDSQCQVLLDCMQRLEANPGDIVVKQGGEGDCFYVVGSGEFEVLATQDGKNGEVPRILQRYTAEKQSSFGELALMHNKPLQASVRAVDHGTLWL KREDFRGILMSEFSNLASLKLLRSVDLLSRLTILQLSHVAESLSEACFSDGQTIVTKDQKLQGLYVIQKGRVKISFCTEVLESQNVSSLTTGITNEYDNLEIGTEVSIEKHEGSYFGEWALLGELKDSLSVVAVGEVVCVVLTKENFESAVGPLTNISDDGPKTRHSSFELSKESAKVTDTTALAKATLADLEWTTCLSTTDCSEIGLVHLKDKENLLSLKRFSKQKVKKLGKEAQVLKERNLMKNVIKPSAIVPEILCTCVDQTFAAILLNTTLACPISSLLHSPLDESSVRFITGSLVSAIEDIHKNEILFRGSSPELLMLDQSGYLQIVDFRFAKKLSGERTFTICGNADYLAPEIVQGKGHGYAADWWALGVLIYYMLEGEMPFGSWRESELDTFQKIAKGQLTFPRVLSSEAEDLITKLLEVDEN LRFGSQGGPE SIKKHPWFNG LKWEAISNREFQVPQEIISRIHHHLENDNVLPLETSKSLDTTEDQDAQNWLEEW |
| AT2G20630 | MAGREILHKMKVGLCGSDTGRGKTKVWKNIAHGYDFVKGKAGHPMEDYVVSEFKKVDGHDLGLFAIFDGHLGHDVAKYLQTNLFDNILKEKDFWTDTKNAIRNAYISTDAVILEQSLKLGKGGSTAVTGILIDGKTLVIANVGDSRAVMSKNGVASQLSVDHEPSKEQKE IESRGGFVSN IPGDVPRVDGQLAVARAFGDKSLKIHLSSDPDIRDENIDHETEFILFASDGVWKVMSNQEAVDLIKSIKDPQAAAKELIEEAVSKQSTDDISCIVPCFLRREALSERYCR |
| AT2G25070 | MGTYLSSPKTEKLSEDGENDKLRFGLSSMQGWRATMEDAHAAILDLDDKTSFFGVYDGHGGKVVAKFCAKYLHQQVISNEAYKTGDVETSLRRAFFRMDDMMQGQRGWRELAVLGDKMNKFSGMIEGFIWSPRSGDTNNQPDSWPLEDGPHSDFTGPTSGCTACVALIKDKKLFVANAGDSRCVISRKSQAYNLSKDHKPDLEVEKERILKAGGFIHAGRINGSLNLTRAIGDMEFKQNKFLPSEKQMVTADPDINTIDLCDDDDFLVVACDGIWDCMSSQELVDFIHEQ LKSETKLSTVCEKVVDRCLAPDTATGEGCDNMTIILVQFKKPNPSETEPEDSKPEPSEDEPSSSS |
| AT2G25620 | MEETRGISDPENGSSSYGGKPPNPLSFSSSSAAAAVYRQTFDGERSLAPCNKRSLVRHSSLVKTMVSDISVENEFTIEKNKSEFVPATRSGAWSDIGSRSSMEDAYLCVDNFMDSFGLLNSEAGPSAFYGVFDGHGGKHAAEFACHHIPRYIVEDQEFPSEINKVLSSAFLQTDTAFLEACSLDGSLASGTTALAAILFGRSLVVANAGDCRAVLSRQGKAIEMSRDHKPMSSKERRRIEASGGHVFDGYLNGQLNVARALGDFHMEGMKKKKDGSDCGPLIAEPELMTTKLTEEDEFLIIGCDGVWDVFMSQNAVDFARRRLQEHNDPVMCSKELVEEALKRKSADNVTAVVVCLQPQPPPNLVAPRLRVHRSFSAEGLKDLQSYLDGLGN |
| AT2G28890 | MGNGIGKLSKCLTGGAGRNKKPELSILEPDPLDEGLGHSFCYVRPDPTRVSSSKVHSEEETTTFRTISGASVSANTATPLSTSLYDPYGHIDRAAAFESTTSFSSIPLQPIPRSSGPIVPGSGPLERGFLSGPIERGFMSGPLDGSSGPIDGKTGSDQFQRSFSHGLANLRVGSRKGSLVRVLRRAISKTITRGQNSIVAPIKPVKEPDWVFGSDKTRIHQIENNLTVNSLNFSSEGSLLDDDVSLESQNLQWAQGKAGEDRVHVVVSEEHGWLFVGIYDGFNGPDAPDYLLSHLYPAVHRELKGLLWDDPKTDAKSSDEADVENRDSSSEKKSKNWEESQRRWRCEWDRDLDRLLKDRSNGLDLDPDPNSSDVLKALSQALRKTEEAYLENADMMLDENPELALMGSCVLVMLMKGEDVYLMNVGDSRAVLGQKAESDYWIGKIKQDLERINEETMNDFDGCGDGEGASLVPTLSAFQLTVDHSTNVEEEVNRIRKEHPDDASAVSNERVKGSLKVTRAFGAGFLKQPKWNNALLEMFQIDYKGTSPYINCLPSLYHHRLGSKDQFLILSSDGLYQYFTNEEAVSEVELFITLQPEGDPAQHLVQELLFRAAKKAGMDFHELLEIPQGERRRYHDDVSIVVISLEGRMWKSCV |
| AT2G29380 | MAEICYEVVTDACPSSVYESTPAHSRRRPRFQTVMHEDWEKNCKRSKQEALATRYSSIPRSSREDFSDQNVDVSSPRYGVSSVCGRRREMEDAVAIHPSFSSPKNSEFPQHYFGVYDGHGCSHVAARCRERLHKLVQEELSSDMEDEEEWKTTMERSFTRMDKEVVSWGDSVVTANCKCDLQTPACDSVGSTAVVSVITPDKIVVANCGDSRAVLCRNGKPVPLSTDHKPDRPDELDRIEGAGGRVIYWDCPRVLGVLAMSRAIGDNYLKPYVSCEPEVTITDRRDDDCLILASDGLWDVVSNETACSVA RMCLRGGGRRQDNEDPAISDKACTEASVLLTKLALARNSSDNVSVVVIDLRR |
| AT2G30020 | MSCSVAVCNSPVFSPSSSLFCNKSSILSSPQESLSLTLSHRKPQTSSPSSPSTTVSSPKSPFRLRFQKPPSGFAPGPLSFGSESVSASSPPGGVLKRKRPTRLDIPIGVAGFVAPISSSAAVAATPREECREVEREGDGYSVYCKRGRREAMEDRFSAITNLHGDRKQAIFGVYDGHGGVKAAEFAAKNLDKNIVEEVVGKRDESEIAEAVKHGYLATDASFLKEEDVKGGSCCVTALVNEGNLVVSNAGDCRAVMSVGGVAKALSSDHRPSRDDERKRIETTGGYVDTFHGVWRIQGSLAVSRGIGDAQ LKKWVIAEPETKISRIEHDHEFLILASDGLWDKVSNQEAVDIARPLCLGTEKPLLLAACKKLVDLSASRGSSDDISVMLIPLRQFI |
| AT2G30170 | MAIPVTRMMVPHAIPSLRLSHPNPSRVDFLCRCAPSEIQPLRPELSLSVGIHAIPHPDKVEKGGEDAFFVSSYRGGVMAVADGVSGWAEQDVDPSLFSKELMANASRLVDDQEVRYDPGFLIDKAHTATTSRGSATIILAMLEEVGILKIGNVGDCGLKLLREGQIIFATAPQEHYFDCPYQLSSEGSAQTYLDASFSIVEVQKGDVIVMGSDGLFDNVFDHEIVSIVTKHTDVAESSRLLAEVASSHSRDTEFESPYALEARAKGFDVPLWKKVLGKKLTGGKLDDVTVIVAKVVS |
| AT2G33700 | MSMDFSPLLTVLEGDFNKDNTSSATEIDTLENLDDTRQISKGKPPRHLTSSATRLQLAANADVDVCNLVMKSLDDKSEFLPVYRSGSCAEQGAKQFMEDEHICIDDLVNHLGAAIQCSSLGAFYGVFDGHGGTDAAHFVRKNILRFIVEDSSFPLCVKKAIKSAFLKADYEFADDSSLDISSGTTALTAFIFGRRLIIANAGDCRAVLGRRGRAIELSKDHKPNCTAEKVRIEKLGGVVYDGYLNGQLSVARAIGDWHMKGPKGSACPLSPEPELQETDLSEDDEFLIMGCDGLWDVMSSQCAVTIARKE LMIHNDPERCSRELVREALK RNTCDNLTVIVVCFSPDPPQRIEIRMQSRVRRSISAEGLN LLKGVLDGYP |
| AT2G34740 | MKKTIKNSTHRPEDILVHAIKKRRSASGDVKNAVKLKEGEDYLRVEEEIPHDDNVVVIDDGCDHDHDVDDNDDDEEENGRYCRREFDHGYHLVKGQMGHGMEDFIVADTKTVKGHNLGLYAIFDGHSGSDVADYLQNHLFDNILSQPDFWRNPKKAIKRAYKSTDDYILQNVVGPRGGSTAVTAIVIDGKKIVVANVGDSRAILCRESDVVKQITVDHEPDKERDLVKSKGGFVSQKPGNVPRVDGQLAMTRAFGDGGLKEHISVIPNIEIAEIHDDTKFLILASDGLWKVMSNDEVWDQ IKKRGNAEEAAKMLIDKALARGSKDDISCVVVSFLQWID |
| AT2G35350 | MGSGFSSLLPCFNQGHRNRRRHSSAANPSHSDLIDSFREPLDETLGHSYCYVPSSSNRFISPFPSDRFVSPTASFRLSPPHEPGRIRGSGSSEQLHTGFRAISGASVSANTSNSKTVLQLEDIYDDATESSFGGGVRRSVVNANGFEGTSSFSALPLQPGPDRSGLFMSGPIERGATSGPLDPPAGEISRSNSAGVHFSAPLGGVYSKKRRKKKKKSLSWHPIFGGEKKQRPWVLPVSNFVVGAKKENIVRPDVEAMAASSGENDLQWALGKAGEDRVQLAVFEKQGWLFAGIYDGFNGPDAPEFLMANLYRAVHSELQGLFWELEEEDDNPTDISTRELEQQGEFEDHVNEMASSSCPATEKEEEEMGKRLTSSLEVVEVKERKRLWELLAEAQAEDALDLSGSDRFAFSVDDAIGAGNAVSVGSKRWLLLSKLKQGLSKQGISGRKLFPWKSGVEENETEEVDNVGVEEGVDKRRKRRKAGTVDHELVLKAMSNGLEATEQAFLEMTDKVLETNPELALMGSCLLVALMRDDDVYIMNIGDSRALVAQYQVEETGESVETAERVEERRNDLDRDDGNKEPLVVDSSDSTVNNEAPLPQTKLVALQLTTDHSTSIEDEVTRIKNEHPDDNHCIVNDRVKGRLKVTRAFGAGFLKQPKLNDALLEMFRNEYIGTDPYISCTPSLRHYRLTENDQFMVLSSDGLYQYLSNVEVVSLAMEKFPDGDPAQHVIQELLVRAAKK AGMDFHELLDIPQGDRRKYHDDCTVLVIALGGSRIWKSSGKYL |
| AT2G40180 | MQLSKNPIKQTRNREKNYTDDFTMKRSVIMAPESPVFFPPPLVFSPTSVKTPLSSPRSSPPKLTMVACPPRKPKETKTTGSDSETVLKRKRPPMLDLTAAPTVASWCSTTRETAEKGAEVVEAEEDGYYSVYCKRGRRGPMEDRYFAAVDRNDDGGYKNAFFGVFDGHGGSKAAEFAAMNLGNNIEAAMASARSGEDGCSMESAIREGYIKTDEDFLKEGSRGGACCVTALISKGELAVSNAGDCRAVMSRGGTAEALTSDHNPSQANELKRIEALGGYVDCCNGVWRIQGTLAVSRGIGDRYLKEWVIAEPETRTLRIKPEFEFLILASDGLWDKVTNQEAVDVVRPYCVGVENPMTLSACKKLAELSVKRGSLDDISLIIIQLQNFLP |
| AT2G40860 | MVMEIVKPNTCIRGCCTSESIPLHLPSSSFTLLSPIAKGSESVVYEAILDGRRVAAKKPILSTSDDLDKFHRNLQLSCNLNHPGVAKLLAAHAKPPNYMFFFDFYESGTLAEKLHVEEWSPSIDQVLLITLHLAKALQYLHNNGIVHRDVKPANVLLDEKFFPYLADFGLAEYKKNLREVNLQNWRSSGKPTGGFHKKNMVGTLIYMAPEILRKDMYTEKADIYSFGILINELLTGVVPYTDRRAEAQAHTVLEMNYTEQQLTVAIVSSGLRPALAEIGLHLPKSLLSLIQNCWESDPSKRPSSDNVVLELESIWEQVRGKQQGHLLEKTSNSQSDTDGADIIKNSGDYRDTVNWFSQGECLSKKSSVSTVFDVKLWSSSTDEPSRYVPVISCGSFATCGRRESMEDTHFIIPHMCNEESIHLFAIFDGHRGAAAAEFSAQVLPGLVQSLCSTSAGEALSQAFVRTDLAFRQELDSHRQSKRVSQKDWHPGCTAIASLLVENKLFVANVGDSRAILCRAGHPFALSKAHLATCIDERNRVIGEGGRIEWLVDTWRVAPAGLQVTRSIGDDDLKPAVTAEPEISETILSADDEFLVMASDGLWDVMNDEEVIGIIRDTVKEPSMCSKRLATEAAARGSGDNITVIVVFLRPVSTAERIY |
| AT2G46920 | MGNGTSRVVGCFVPSNDKNGVDLEFLEPLDEGLGHSFCYVRPSIFESPDITPSNSERFTIDSSTIDSETLTGSFRNDIVDDPSFLNRHNSKGLAETTFKAISGASVSANVSTARTGNQMALCSSDVLEPAASFESTSSFASIPLQPLPRGGSGPLNGFMSGPLERGFASGPLDRNNGFMSGPIEKGVMSGPLDVSDRSNFSAPLSFRRKKPRFQRFMRSVSGPMKSTLARTFSRRSGGLSWMHRFFLHPETRVSWAVGKDGKLHGEDPESCLESNRNLQWAHGKAGEDRVHVVLSEEQGWLFIGIYDGFSGPDAPDFVMSHLYKAIDKELEGLLWDYEEPSEDNQLQPDQEPPTEENMCDPESISEQHSKSVVAESEEVMIDDISSLGNTDTQIADGPPGDSAGPGKKSMRLYELLQLEQWEGEEIGLKRYGGNVALNNMTNQVENPSTSGGGAGNDPCTTDRSALDGIPNSGQRHGTKKSQISSKIRRMYQKQKSLRKKLFPWSYDWHREEGICVEEKIVESSGPIRRRWSGTVDHDAVLRAMARALESTEEAYMDMVEKSLDINPELALMGSCVLVMLMKDQDVYVMNVGDSRAILAQERLHDRHSNPGFGNDEGIGHKSRSRESLVRIELDRISEESPIHNQATPISVSNKNRDVTSYRLKMRAVQLSSDHSTSVEEEIWRIRSEHPEDDQSILKDRVKGQLKVTRAFGAGFLKKPNFNEALLEMFQVEYIGTDPYI TCEPCTVHHRLTSSDRFMVLSSDGLYEYFSNEEVVAHVTW FIENVPEGDPAQYLIAELLSRAATKNGMEFHDLLDIPQGDRRKYHDDVSVMVVSLEGRIWRSSGQYYPERKQKFNR |
| AT3G02750 | MGSCLSAESRSPRPGSPCSPAFSVRKRKNSKKRPGSRNSSFDYRREEPLNQVPGRMFLNGSTEVACIYTQQGKKGPNQDAMVVWENFGSRTDTIFCGVFDGHGPYGHMVAKRVRDNLPLKLSAYWEAKVPVEGVLKAITTDTVNNVTNINNPEDAAAAAAFVTAEEEPRTSADMEEENTETQPELFQTLKESFLKAFKVMDRELKFHGSVDCFCSGTTAVTLIKQGQYLVVGNVGDSRAVMGTRDSENTLVAVQLTVDLKPNLPGWIILCECMMLSCGCMMDPLIMFIGFFFIPSIELAAEAERIRKCRGRVFALRDEPEVCRVWLPNCDSPGLAMARAFGDFCLKDFGLISVPDVSFRQLTEKDEFIVLATDGIWDVLSNEDVVAIVASAPSRSSAARALVESAVRAWRYKYPTSKVDDCAAVCLYLDSSNTNAISTASSISKLEDGEEEELKATTEDDDASGPSGLGRSSTVRSGKEI ALDESETEKLIKEADNLDSEPGTEYSALEGVARVNTLLNLPRFVPGK |
| AT3G05640 | MGHFSSMFNGIARSFSIKKAKNINSSKSYAKEATDEMAREAKKKELILRSSGCINADGSNNLASVFSRRGEKGVNQDCAIVWEGYGCQEDMIFCGIFDGHGPWGHFVSKQVRNSMPISLLCNWKETLSQTTIAEPDKELQRFAIWKYSFLKTCEAVDLELEHHRKIDSFNSGTTALTIVRQGDVIYIANVGDSRAVLATVSDEGSLVAVQLTVDFKPNLPQEEERIIGCNGRVFCLQDEPGVHRVWQPVDESPGLAMSRAFGDYCIKDYGLVSVPEVTQRHISIRDQFIILATDGVWDVISNQEAIDIVSSTAERAKAAKRLVQQAVRAWNRKRRGIAMDDISAVCLFFHSSSSSPSL |
| AT3G06270 | MGCVQCKCCSRYPSSSSDGDSRGPLEANGVLKGKDQKPLGSIHVPSPNFDMVYSVLSQRGYYPDSPDKENQDTYCIKTELQGNPNVHFFGVFDGHGVLGTQCSNFVKERVVEMLSEDPTLLEDPEKAYKSAFLRVNEELHDSEIDDSMSGTTAITVLVVGDKIYVANVGDSRAVLAVKDRNRILAEDLSYDQTPFRKDECERVKACGARVLSVDQVEGLKDPNIQTWANEESEGGDPPRLWVQNGMYPGTAFTRSVGDFTAESIGVIAEPEVSMVHLSPNHLFFVVASDGIFEFLPSQAVVDMVGRYADPRDGCAAAAAESYKLWLEHENRTDDITIIIVQIKKLSNE |
| AT3G09400 | MGNGVASFSGCCAGTTAGEISGRYVTGVGLVQENLGHSFCYVRPVLTGSKSSFPPEPPLRPDPIPGTTTTFRSISGASVSANTSTALSTSLSTDTSGIASAFESSNRFASLPLQPVPRSPIKKSDHGSGLFERRFLSGPIESGLVSGKKTKEKAKLKKSGSKSFTKPKLKKSESKIFTFKNVFTNLSCSKKSVIKPINGFDSFDGSSDTDRYIPEINSLSTIVSSHEKPRIKEEEDKTESALEEPKIQWAQGKAGEDRVHVILSEENGWLFVGIYDGFSGPDPPDYLIKNLYTAVLRELKGLLWIDKGESYNRNGESNIEKQSTVEHASDSDQENCPVMNGNDVACGSRNITSDVKKLQWRCEWEHNSSNKSNNINHKDVLRALQQALEKTEESFDLMVNENPELALMGSCVLVTLMKGEDVYVMSVGDSRAVLARRPNVEKMKMQKELERVKEESPLETLFITERGLSLLVPVQLNKEHSTSVEEEVRRIKKEHPDDILAIENNRVKGYLKVTRAFGAGFLKQPKWNEALLEMFRIDYVGTSPYITCSPSLHHHRLSSRDKFLILSSDGLYEYFSNEEAIFEVDSFISAFPEGDPAQHLIQEVLLRAAKKYGMDFHELLEIPQGDRRRYHDDVSVIVISLEGRIWRSSM |
| AT3G11410 | MAGICCGVVGETEPAAPVDSTSRASLRRRLDLLPSIKIVADSAVAPPLENCRKRQKRETVVLSTLPGNLDLDSNVRSENKKARSAVTNSNSVTEAESFFSDVPKIGTTSVCGRRRDMEDAVSIHPSFLQRNSENHHFYGVFDGHGCSHVAEKCRERLHDIVKKEVEVMASDEWTETMVKSFQKMDKEVSQRECNLVVNGATRSMKNSCRCELQSPQCDAVGSTAVVSVVTPEKIIVSNCGDSRAVLCRNGVAIPLSVDHKPDRPDELIRIQQAGGRVIYWDGARVLGVLAMSRAIGDNYLKPYVIPDPEVTVTDRTDEDECLILASDGLWDVVPNETACGVARMCLRGAGAGDDSDAAHNACSDAALLLTKLALARQSSDNVSVVVVDLRKRRNNQASS |
| AT3G12620 | MVSSATILRMVAPCWRRPSVKGDHSTRDANGRCDGLLWYKDSGNHVAGEFSMSVIQANNLLEDHSKLESGPVSMFDSGPQATFVGVYDGHGGPEAARFVNKHLFDNIRKFTSENHGMSANVITKAFLATEEDFLSLVRRQWQIKPQIASVGACCLVGIICSGLLYIANAGDSRVVLGRLEKAFKIVKAVQLSSEHNASLESVREELRSLHPNDPQIVVLKHKVWRVKGIIQVSRSIGDAYLKKAEFNREPLLAKFRVPEVFHKPILRAEPAITVHKIHPEDQFLIFASDGLWEHLSNQEAVDIVNTCPRN GIARKLIKTALREAAKKREMRYSDLKKIDRGVRRHFHDDITVIVVFLDSHLVSRSTSRRPLLSISGGGDLAGPST |
| AT3G15260 | MTGREILHKMKESVKEKVGLGASASSADSGKGKSKMLKQITHGFHLVKGKAFHEMEDYVVAKFKEVDDNELGLFAIFDGHLSHEIPDYLCSHLFENILKEPNFWQEPEKAIKKAYYITDTTILDKADDLGKGGSTAVTAILINCQKLVVANVGDSRAVICQNGVAKPLSVDHEPNMEKDEIENRGGFVSNFPGDVPRVDGQLAVARAFGDKSLKMHLSSEPYVTVEIIDDDAEFLILASDGLWKVMSNQEAVDSIKGIKDAKAAAKHLAEEAVARKSSDDISVVVVKFQ |
| AT3G16560 | MQEGTDPYGEIEISFGYQCNNKKIGIPEDKIADGREVLGGFRLQKTSSFSCLSGAALSGNPTLANTNICNGVIGSEILPSLDSPKSFRKVPSSPALSKLDILSPSLHGSMVSLSCSSSTSPSPPEPESCYLTSMSSPSSVNEGFLLSAMEVQVAGGAAGEDRVQAVCSEENGWLFCAIYDGFNGRDAADFLACTLYESIVFHLQLLDRQMKQTKSDDDGEKLELLSNISNVDYSSTDLFRQGVLDCLNRALFQAETDFLRMVEQEMEERPDLVSVGSCVLVTLLVGKDLYVLNLGDSRAVLATYNGNKKLQAVQLTEDHTVDNEVEEARLLSEHLDDPKIVIGGKIKGKLKVTRALGVGYLKKEKLNDALMGILRVRNLLSPPYVSVEPSMRVHKITESDHFVIVASDGLFDFFSNEEAIGLVHSFVSSNPSGDPAKFLLERLVAKAAARAGFTLEELTNVPAGRRRRYH DDVTIMVITLGTDQRTSKASTFV |
| AT3G16800 | MVLLPAFLDGLARTVSTKKGKKLSEDEDGGREIAKSMIKDSKKNSTLLGTSGFVSSESSKRFTSICSNRGEKGINQDRAIVWEGFGCQEDITFCGMFDGHGPWGHVIAKRVKKSFPSSLLCQWQQTLASLSSSPECSSPFDLWKQACLKTFSIIDLDLKISPSIDSYCSGCTALTAVLQGDHLVIANAGDSRAVIATTSDDGNGLVPVQLSVDFKPNIPEEAERIKQSDGRLFCLDDEPGVYRVGMPNGGSLGLAVSRAFGDYCLKDFGLVSEPEVTYRKITDKDQFLILATDGMWDVMTNNEAVEIVRGVKERRKSAKRLVERAVTLWRRKRRSIAMDDISVLCLFFRPS |
| AT3G17090 | MSGSLMNLFSLCFKPFGHVCDNSEAGSGGGGGVSGGTGGEGKDGLLWFRDLGKYCGGDFSMAVIQANQVLEDQSQVESGNFGTFVGVYDGHGGPEAARYVCDHLFNHFREISAETQGVVTRETIERAFHATEEGFASIVSELWQEIPNLATVGTCCLVGVIYQNTLFVASLGDSRVVLGKKGNCGGLSAIQLSTEHNANNEDIRWELKDLHPDDPQIVVFRHGVWRVKGIIQVSRSIGDMYMKRPEFNKEPISQKFRIAEPMKRPLMSATPTILSHPLHPNDSFLIFASDGLWEHLTNEKAVEIVHNHPRAGSAKRLIKAALHEAARKREMRYSDLRKIDKKVRRHFHDDITVIVVFLNHDLISRGHINSTQDTTVSIRSALEH |
| AT3G17250 | MRTSKASVTQTWLLYTQLCLWKDLIIRYVRQIIRRAKSMLFSQNMVADSAEISVIDVKSHLSVAKDPSNFQIAEIRIHDSICIDIPSSEETPLLESIKSCSATTIEEHVTEFVPNISSGSYADKGDYREYMEDEHICIDDLSDHLGSSFYRFPVPMAFYGVFDGHGGSDASQYIKENAMSLFFEDAVFRQSPSVVDSLFLKELETSHREAYRLADLAMEDERIVSSSCGTTALTALVIGRHLMVANVGDCRAVLCRKGKAVDMSFDHKSTFEPERRRVEDLGGYFEGEYLYGDLAVTRALGDWSIKRFSP LGESLSPLISDPDIQQMILTEEDEFLIMGCDGVWDVMTSQYAVTFVRQGLRRHGDPRRCA MELGREALRLDSSDNVTVVVICFSSSPAPQ RRRIRFCVSD EARARLQTMLEG |
| AT3G23360 | MGFLDLPFMLKAFRFRRLVVEDGKRRKKKKPLWLTPVSHGYYTVDRLSYADNSSNDDSVFVQREQQSDELEIWLFGVSNAGTGKEIVKYMQNHLFDKLPNELGIMRKCKETMRRAYVEEERTGGSAASVMVVNGEKLAIASIGDHRVVVCKDGEAHQIRDRKASTKHWSQFIFPVCNQGEEEDESDPRNSELVVITEKINSDTEFIIIGSPGIWEVMKSQEAINLIRHIEDPKEAAKCLAKEALNRISKSSISCVVIRFG |
| AT3G27140 | MEDRFSTITNLHGDRKQAIFGVYVGHGGVKAAECPAKNLDKNIVEEVVGKRHELEIAEAGGSSCVTALVSEGSLVVSNAGDCRAVMSVGGVAKGSLVVPRGIGDAQLKKWVIAEPETKISRVEHDHEFLILASHGLWDKVSNQEAVDIARPFCLRTEKPLLLAACKKLVDLSASRGSFDD ISVMLIPLRPVRIEKRGILEDVSSSKANSIARDIAISVTRDGRFRSYLARGGPGWLLSRI EEDKR |
| AT3G51370 | MLSTLMKLLSACLWPSSSSGKSSDSTGKQDGLLWYKDFGQHLVGEFSMAVVQANNLLEDQSQVESGPLSTLDSGPYGTFIGIYDGHGGPETSRFVNDHLFQHLKRFAAEQASMSVDVIKKAYEATEEGFLGVVTKQWPTKPQIAAVGSCCLVGVICGGMLYIANVGDSRAVLGRAMKATGEVIALQLSAEHNVSIESVRQEMHSLHPDDSHIVMLKHNVWRVKGLIQISRSIGDVYLKKAEFNKEPLYTKYRIREPFKRPILSGEPTITEHEIQPQDKFLIFASDGLWEQMSNQEAVDIVQNHPRNGIARRLVKMALQEAAKKREMRYSDLKKIERGVRRHFHDDITVVIIFLDTNQVSSVKGPPLSIRGGGMTFPKKI |
| AT3G51470 | MAPVTEVSPMINTLEVADDKMTNLSSSGKPPRNISAMRHCNSTAWLTDYEGDERFGAKSPEGVNSTFQPVFRSGSWSDKGPKQSMEDEFICVDDLTEYIGSSTGAFYGVFDGHGGVDAASFTKKNIMKLVMEDKHFPTSTKKATRSAFVKTDHALADASSLDRSSGTTALTALILDKTMLIANAGDSRAVLGKRGRAIELSKDHKPNCTSERLRIEKLGGVIYDGYLNGQLSVARALGDWHIKGTKGSLCPLSCEPELEEIVLTEEDEYLIMGCDGLWDVMSSQCAVTMVRRELMQHNDPERCSQALVKEALQRNSCDNLTVVVVCFSPEAPPRIEIPKSHKRRSISAEGLDLLKGVLNEL |
| AT3G55050 | MVSTTFRRIVSPCWRPFGIGEDSSPGSDDTNGRLDGLLWYKDSGNHITGEFSMAVVQANNLLEDHSQLESGPISLHESGPEATFVGVYDGHGGPEAARFVNDRLFYNIKRYTSEQRGMSPDVITRGFVATEEEFLGLVQEQWKTKPQIASVGACCLVGIVCNGLLYVANAGDSRVVLGKVANPFKELKAVQLSTEHNASIESVREELRLLHPDDPNIVVLKHKVWRVKGIIQVSRSIGDAYLKRAEFNQEPLLPKFRVPERFEKPIMRAEPTITVHKIHPEDQFLIFASDGLWEHLSNQEAVDIVNSCPRNGVARKLVKA ALQEAAKKREMRYSDLEKIERGIRRHFHDDITVIVVFLHATNFATRTPISVKGGGLLSAHNPVL |
| AT3G62260 | MVAEAEVVFQQSLPAVLEIELFDGVSSAVKSPVSSPKLGFTQSTASVSGSLTTSPVQADIFPEGDCDPSVLDYIPTIRSGSFADIGPKRNMEDEHIRIDDLSSQVGSLFELPKPSAFYAVFDGHGGPEAAAYVRENAIRFFFEDEQFPQTSEVSSVYVEEVETSLRNAFLQADLALAEDCSISDSCGTTALTALICGRLLMVANAGDCRAVLCRKGRAIDMSEDHKPINLLERRRVEESGGFITNDGYLNEVLAVTRALGDWDLKLPHGSQSPLISEPEIKQITLTEDDEFLVIGCDGIWDVLTSQEAVSIVRRGLNRHNDPTRCARELVMEALGRNSFD NLTAVVVCFMTMDRGDKPVVPLEKRRCFSLSPEAFCSLRN LLDG |
| AT3G63320 | MVELRQFSDLPIALSGISRIADPSPPPPVVAIRRRFKGGGNTRRIVFSVPLIFAFPFPTGTPKDVLVGIAAVFDGHSGSEASEMASQLLLDYFALHIYFLLDATFSKELTGKLPNSLMHLYDLDSQRFQDSLPLNFHLDILKEALLRAIYDIDATFTKEASTRKLDSGSTATIALIADGQLLVASIGDSKALLCSERYETPEEAKATLIKLYRERKRNQDSSPSRFSDLKLEHRTGLMRFIAKELTKDHHPDREDEMLRVKAAGGYVTKWAGVPRVNGQLAVSRSIGDLTYRSYGVISAPEVMDWQPLVA NDSYLVVSSDGIFEKLEVQDACDRLWEVKNQTSFGAGVPSYCSISLADCLVNTAFEKGSMDNMAAVVVPLKSNLDWESQPKEQSVGPSGFKMKNTYALPCEFLSSQPNLFRMG |
| AT3G63340 | MTSSIKSSLLNLGLLIIFFVFFFLVINCRGESSTCLAVYKQGGAPAVFQSPKCPRWILQNWGSPTHSGAGRCHTAAIQGRRNYQEDRLLCALDLRIPFPGKTGTPKDVLVGIAAVFDGHNGAEASDMASKLLLDYFALHINFLLDATFSAMTRKLIGRFPTKGDHSVILHGVSRDEIMHLYNLDFQMQFRDSLPLHFDDSLPLDIMKEALLRAIHDIDVTFTKEASNRKLNSGSTATIALIADGQLMVASIGDSKALLCSEKFETLEEARGLATSVSKIKLPNKKPVLYLFINSLLQWCL FIAATLVKLYRERRRNRGSSPSRFSDFKLEHGNGLLRFIAKELTKDHHPNREDEKIRVEAAGGYVTEWAGVPRVNGQLTVSRAIGDLTYRSYGVISAPEVMDWQPLVANDSFLVVSSDGIFEKLEVQEVCDLLWEVNNQTSSGAGVPSYCSISLADCLVNTAFEKGSMDNMAAVVVPLKSNLVTQLQRKEQSMNDNKDKIASALPCSNCTLPPVPNDINLGPLQLKQAQPLGTMFNRLLVEVKNGSFCRFYMSENLIGASQGQMNNLNGYMGDLPQVLPASAEQFPGWCLPSGTATNENQDQCINPDSFATFLGLLESVPLHGFGAKNGTDEIPFPDSSYVLKKKFGRGAFGEVWLAFHWDCYQGNNATSSINEDENTSKNGVHNDTDGPNNSFILKRIMVERGPTVYLSGLREKHFGELFLNAYNVSESSSATQASSSQAASSELGLSEEGLKHIARYIEYFESRYNDIWLVFHHEGVSLSKLMYTVEEAEISSEKAEEASHGQILRPSKWWTWLKTTESGKEEMRRIIWQLLLGLKACHDRNITHRDIKPENMVICLEDIKSGRCLKGVPNGDQNFKTNMRIIDFGSALDEYTIKHLYGSTGPSRAEQTHDYAPPEAILNSSWHHGPTSLTLKYDMWSVGVVMLEMILGSPNVFEISSVTRALLDQHIRGWSENFKELAYKLRSLMEMCILIPGSSLKHGGASSKQGGISLASWKCSEEFFAEQIKSRDPLKIGFPNVWALRLVRGLLQWYPEDRVTVDEALQHPYFQPPPSS |
| AT4G03415 | MGGCVSTSSKSTCSSWSNGEKPVRRPYLGIGCCVSKRAKRTFSDHIVSLQNLTSIPNRITSSSKSRSSCIFTQQGRKGINQDAMIVWEDFMSEDVTFCGVFDGHGPYGHLVARKVRDTLPVKLQFFFQTLQSKQNCSKGTRFRRNSSKSAVQEAVKEGSDEDKLKGLWGEAFLKSFKAMDKELRSHPNLDCFCSGSTGVTILKQGSNLFMGNIGDSRAILGSKDSNDSMVATQLTVDLKPDLPREAERIKRCKGRVFAMEDEPEVPRVWLPYDDAPGLAMARAFGDFCLKEYGVISVPEFTHRVLTDRDQFIVLASDGVWDVLSNEEVVDIVASATSRASAARTLVNSAAREWKLKYPTSKMDDCAVVCLFLDGKMDSESDYDEQGFSSATNAVESDDGQRSEPCLQRNFTVRSSSDQENETYGNVNTETDAEDEKTVGD QNWLGLQGVTRVNSLVQLPRFSEEKSKT |
| AT4G08260 | MEDRFSAITNLHGDHKQAIFGVYVGHGGVKAAEFAAKNLDKNIVEEVVDATFLKEEGFKGGSSCVTALVSEGSLVVSNAGDCRAVMSVGEMMNGKELKPREDMLIRFTLWRIQGSLVVPRGIGDAQLKKWVIAEPETKISRVEHDHEFLILASHGLWDKVSNQEAVDIARPFCLRTEKPLLLAACKKLVDLSASRGSFDDISVMLIPLRQFV |
| AT4G11040 | MKTDTTLPIIAEDGDCGDSKRVRVADSGYTVGGQDRPVKLPKIENNGDVGTSEGTHVLVDALMAEVAIKDKDGKTNAGHGVVSVMGRQRAMTTAVSTVVDEIPSYDIFGIFDGLRLAKFFEDRLRRLVKEEVKACHGRGVAADWNKVMKSCFSEAVGTVGTTTSAVVTIVGKEEVIVLCRGGARVVLYSHDGVALPLCHIHHHKDGVEQILKIHKRKKIDDFIVLACDGLWDVVSDDDTYQLVKRCLYGKLPPDGCISESSSTKAAVILAELAIARGSKENINVIVIDLKSSTVS |
| AT4G16580 | MLPVRESLQKQVKILIGLGNLGFGGYRGLYTRFTNPNGFLEPASSDLLLINERRNLSVIGAVSRTFSVPSVSGPAFQVCGYHIDLLLSDPCKSMASLGSKSLFVDRHSASLVSKRFTGGMVSGDGPNRGRISMRLRGKDHNEKSTICAYFAYRGAKRWIYLNQQRRGMGFRGLHSSLSNRLSAGNAPDVSLDNSVTDEQVRDSSDSVAAKLCTKPLKLVSGSCYLPHPDKEATGGEDAHFICAEEQALGVADGVGGWAELGIDAGYYSRELMSNSVNAIQDEPKGSIDPARVLEKAHTCTKSQGSSTACIIALTNQGLHAINLGDSGFMVVREGHTVFRSPVQQHDFNFTYQLESGRNGDLPSSGQVFTVAVAPGDVIIAGTDGLFDNLYNNEITAIVVHAVRANIDPQVTAQKIAALARQRAQDKNRQTPFSTAAQDAGFRYYGGKLDDITVVVSYVAASKEEGKH |
| AT4G26080 | MEEVSPAIAGPFRPFSETQMDFTGIRLGKGYCNNQYSNQDSENGDLMVSLPETSSCSVSGSHGSESRKVLISRINSPNLNMKESAAADIVVVDISAGDEINGSDITSEKKMISRTESRSLFEFKSVPLYGFTSICGRRPEMEDAVSTIPRFLQSSSGSMLDGRFDPQSAAHFFGVYDGHGGSQVANYCRERMHLALAEEIAKEKPMLCDGDTWLEKWKKALFNSFLRVDSEIESVAPETVGSTSVVAVVFPSHIFVANCGDSRAVLCRGKTALPLSVDHKPDREDEAARIEAAGGKVIQW NGARVFGVLAMSRSIGDRYLKPSIIPDPEVTAVKRVKEDDCLILASDGVWDVMTDEEACEMARKRILLWHKKNAVAGDASLLADERRKEGKDPAAMSAAEYLSKLAIQRGSKDNISVVVVDLKPRRKLKSKPLN |
| AT4G27800 | MALLRPHLHRFHSNTLRHSAYPSADAGGGLVVYPTYGRHRCSAIAIDAPSSLTGVTPIRWGYTSVQGFRDEMEDDIVIRSDAVDSFSYAAVFDGHAGSSSVKFLREELYKECVGALQAGSLLNGGDFAAIKEALIKAFESVDRNLLKWLEANGDEEDESGSTATVMIIRNDVSFIAHIGDSCAVLSRSGQIEELTDYHRPYGSSRAAIQEVKRVKEAGGWIVNGRICGDIAVSRAFGDIRFKTKKNDMLKKGVDEGRWSEKFVSRIEFKGDMVVATPDIFQVPLTSDVEFIILASDGLWDYMKSSDVVSYVRDQLRKHGNVQLACESLAQVALDRRSQDNISIIIADLGRTEWKNLPAQRQNVVVELVQAATTIGLVTVGIWMSSHLS |
| AT4G28400 | MAGSNILHKIKLKAGFCGSAPDMGRGKSKMWKNITHGFHCVKGKSSHPMEDYVVSEFKKLEGHELGLFAIFDGHLGHDVAKYLQTNLFDNILKEKDFWTDTENAIRNAYRSTDAVILQQSLKLGKGGSTAVTGILIDGKKLVVANVGDSRAVMSKNGVAHQLSVDHEPSKEKKEIESRGGFVSNIPGDVPRVDGQLAVARAFGDKSLKLHLSSEPDITHQTIDDHTEFILFASDGIWKVLSNQEAVDAIKSIKDPHAAAKHLIEEAISRKSKDDISCIVVKFH |
| AT4G31750 | MGYLNSVLSSSSQVHSDDGPVSGGGLSQNGKFSYGYASSPGKRSSMEDFYETRIDGVEGEIVGLFGVFDGHGGARAAEYVKQNLFSNLIRHPKFISDTTAAIADAYNQTDSEFLKSENSQNRDAGSTASTAILVGDRLLVANVGDSRAVICRGGNAIAVSRDHKPDQSDERQRIEDAGGFVMWAGTWRVGGVLAVSRAFGDRLLKQYVVADPEIQEEKVDSSLEFLILASDGLWDVVSNEEAVGMIKAIEDPEEGAKRLMMEAYQRGSADNITCVVVRFFSDQAGGIGSSSTNIPIDHGIVPDRISGDSST |
| AT4G31860 | MGIYLSTPKTDKFSEDGENHKLRYGLSSMQGWRASMEDAHAAILDLDDNTSFLGVYDGHGGKVVSKFCAKYLHQQVLSDEAYAAGDVGTSLQKAFFRMDEMMQGQRGWRELAVLGDKINKFSGMIEGLIWSPRSGDSANKPDAWAFEEGPHSDFAGPNSGSTACVAVVRDKQLFVANAGDSRCVISRKNQAYNLSRDHKPDLEAEKERILKAGGFIHAGRVNGSLNLSRAIGDMEFKQNKFLPSEKQIVTASPDVNTVELCDDDDFLVLACDGIWDCMTSQQLVDFIHEQ LNSETKLSVVCEKVLDRCLAPNTSGGEGCDNMTMILVRFKNPTPSETELKPEASQAEGNHDEPSSSN |
| AT4G32950 | MGFCFCLSSGGSTDKSQIYEITDYGQENAVLYSDHHVVPQNLGSVSSLAGGKGLNQDAAILHLGYGTEEGALCGVFDGHGPRGAFVSKNVRNQLPSILLGHMNNHSVTRDWKLICETSCLEMDKRILKVKKIHDCSASGTTAVLAVKHGNQVMVANLGDSRAVMIGTSEDGETKVAQLTNDLKPSVPSEAERIRKRNGRVLALESEPHILRVWLPTENRPGLAMSRAFGDFLLKSYGVIATPQVSTHQITSSDQFLLLASDGVWDVLSNEEVATVVMKSASEAGAANEVAEAATNAWIQKFPTVKIDDISVVCLSLNKKHNPQPQI |
| AT4G33500 | MADHLILSLQAPPFLIFPCSLHRSWRFPGGIRYSVPEFRLSSQLQLANSISPSKSSASSSSPPENSAPEKFDLVSSTQLKDGSHVFRFGDASEIEKYLEAEEKARCVEVETQNAKIAEEASEVSRKQKKLVSSIIETSTEKEETAAPSDLSNVIKIKDRKRVRSPTKKKKETVNVSRSEDKIDAKSASVSNLSSIVSVAEAIPISSTEEEAVVEKEITAKSYNVEPLSSEAMKKVSVNKIGDCETNGYQENRMEVQARPSLSTQQEITPVSTIEIDDNLDVTEKPIEAEENLVAEPTATDDLSPDELLSTSEATHRSVDEIAQKPVIDTSEENLLNTFEAEENPVVEPTATAAVSSDELISTSEATRHSVDEIAQKPIIDTSEKNPMETFVEPEAVHSSVDESTEKLVVVTSDVENDGENVASTTEDEITVRDTITDSGSISNNDDTKVEDLQLPVPETASLEPIKAASGREELVSKAFYLDSGFASLQSPFKALAGREDAYFISHHNWIGIADGVSQWSFEGINKGMYAQELMSNCEKIISNETAKISDPVQVLHRSVNETKSSGSSTALIAHLDNNELHIANIGDSGFMVIRDGTVLQNSSPMFHHFC FPLHITQGCD VLKLAEVYHVNLEEGDVVIAATDGLFDNLYEKEIVSIVCGSLKQSLEPQKIAELVAAKAQEVGRSKTERTPFADAAKEEGYNGHKGGKLDAVTVIISFVKIVST |
| AT4G33920 | MLRALARPLERCLGSRASGDGLLWQSELRPHAGGDYSIAVVQANSRLEDQSQVFTSSSATYVGVYDGHGGPEASRFVNRHLFPYMHKFAREHGGLSVDVIKKAFKETEEEFCGMVKRSLPMKPQMATVGSCCLVGAISNDTLYVANLGDSRAVLGSVVSGVDSNKGAVAERLSTDHNVAVEEVRKEVKALNPDDSQIVLYTRGVWRIKGIIQVSRSIGDVYLKKPEYYRDPIFQRHGNPIPLRRPAMTAEPSIIVRKLKPQDLFLIFASDGLWEHLSDETAVEIVLKHPRTGIARRLVRA ALEEAAKKREMRYGDIKKIAKGIRRHFHDDISVIVVYLDQNKTSSSNSKLVKQGGITAPPDIYSLHSDEAEQRRLLNVLY |
| AT4G38520 | MLSGLMNFLNACLWPRSDQQARSASDSGGRQEGLLWFRDSGQHVFGDFSMAVVQANSLLEDQSQLESGSLSSHDSGPFGTFVGVYDGHGGPETSRFINDHMFHHLKRFTAEQQCMSSEVIKKAFQATEEGFLSIVTNQFQTRPQIATVGSCCLVSVICDGKLYVANAGDSRAVLGQVMRVTGEAHATQLSAEHNASIESVRRELQALHPDHPDIVVLKHNVWRVKGIIQVSRSIGDVYLKRSEFNREPLYAKFRLRSPFSKPLLSAEPAITVHTLEPHDQFIICASDGLWEHMSNQEAVD IVQNHPRNGIAKRLVKVALQEAAKKREMRYSDLKKIDRGVRRHFHDDITVIVVFFDTNLVSRGSMLRGPAVSVRGAGVNLPHNTLAPCTTPTQAAAAGAS |
| AT5G01700 | MGVCCSKGTGIIVEHGADDGNECGDGEAEVRDTNDGAVVRTRGSSKHVSMSIKQGKKGINQDAMTVWENFGGEEDTIFCGVFDGHGPMGHKISRHVCENLPSRVHSKIRSSKSAGDENIENNSSQSQEELFREFEDILVTFFKQIDSELGLDSPYDSFCSGTTAVTVFKQADCLVIANLGHSRAVLGTRSKNSFKAVQLTVDLKPCVQREAERIVSCKGRVFAMEEEPDVYRVWMPDDDCPGLAMSRAFGDFCLKDYGLVCIPDVFCRKVSREDEFVVLATDGIWDVLSNEEVVKVVGSCKDRSVAAEMLVQRAARTWRTKFPASKADDCAVVVLYLNHRPYPREGNVSRAISTISWRSNKSNNECYGAAPLSPLGLSQRVS |
| AT5G02400 | MGNGVTTLTGCCTGTLAGEISRRYDVSLVHDGLGHSFCYIRPDLPGVVLPSPESPLRSDHIQETTFRSISGASVSANPSTALSGALSSDSDCPYSSAVSASAFESSGNFASLPLQPVPRGSTWQSGPIVNESGLGSAPFERRFLSGPIESGLYSGPIESTKKTEKEKPKKIRKKPKSKKNFLTFKTLFANLISNNNKPRLKKSVIEPINGSDSSDSGRLHHEPVITSSRSNENPKSDLEEEDEKQSMNSVLDVQWAQGKAGEDRVHVVVSEDNGWVFVGIYDGFSGPDAPDYLLNNLYTAVQKELNGLLWNDEKLRSLGENGMTKTGKCSDEEDPESGKENCPVINNDDAVASGARNQAKSLKWRCEWEKKSNNKTKSDNRCDQKGSNSTTTNHKDVLKALLQALRKTEDAYLELADQMVKENPELALMGSCVLVTLMKGEDVYVMNVGDSRAVLGRKPNLATGRKRQKELERIREDSSLEDKEILMNGAMRNTLVPLQLNMEHSTRIEEEVRRIKKEHPDDDCAVENDRVKGYLKVTRAFGAGFLKQPKWNDALLEMFRIDYIGTSPYITCSPSLCHHKLTSRDKFLILSSDGLYEYFSNQEAIFEVES FISAFPEGDPAQHLIQEVLL RAANKFGMDFHELLEIPQGDRRRYHDDVSVIVISLEGRIWRSSM |
| AT5G02760 | MVKPCWRIGAGMERSKINPTKVDGLTWYKDLGLHTFGEFSMAMIQANSVMEDQCQIESGPLTFNNPTVQGTFVGVYDGHGGPEASRFIADNIFPKLKKFASEGREISEQVISKAFAETDKDFLKTVTKQWPTNPQMASVGSCCLAGVICNGLVYIANTGDSRAVLGRSERGGVRAVQLSVEHNANLESARQELWSLHPNDPTILVMKHRLWRVKGVIQVTRSIGDAYLKRAEFNREPLLPKFRLPEHFTKPILSADPSVTITRLSPQDEFIILASDGLWEHLSNQEAVDIVHNSPRQGIARRLLKAALKEAAKKREMRYSDLTEIHPGVRRHFHDDITVIVVYLNPHPVKTNSWASPLSIRGGYPMHSTS |
| AT5G06750 | MFSWLARMALFCLRPMRRYGRMNRDDDDDDDHDGDSSSSGDSLLWSRELERHSFGDFSIAVVQANEVIEDHSQVETGNGAVFVGVYDGHGGPEASRYISDHLFSHLMRVSRERSCISEEALRAAFSATEEGFLTLVRRTCGLKPLIAAVGSCCLVGVIWKGTLLIANVGDSRAVLGSMGSNNNRSNKIVAEQLTSDHNAALEEVRQELRSLHPDDSHIVVLKHGVWRIKGIIQVSRSIGDAYLKRPEFSLDPSFPRFHLAEELQRPVLSAEPCVYTRVLQTSDKFVIFASDGLWEQMTNQQAVEIVNKHPRPGIARRLVR RAITIAAKKREMNYDDLKKVERGVRRFFHD DITVVVIFIDNELLMVEKATVPELSIKGFSHTVGPSKFSIFLS |
| AT5G10740 | MGYLDLALSYSNQPQTVEAPASGGGLSQNGKFSYGYASSAGKRSSMEDFFETRIDGINGEIVGLFGVFDGHGGARAAEYVKRHLFSNLITHPKFISDTKSAITDAYNHTDSELLKSENSHNRDAGSTASTAILVGDRLVVANVGDSRAVISRGGKAIAVSRDHKPDQSDERERIENAGGFVMWAGTWRVGGVLAVSRAFGDRLLKQYVVADPEIQEEKIDDTLEFLILASDGLWDVFSNEAAVAMVKEVEDPEDSAKKLVGEAIKRGSADNITCVVVRFLEKKSASSSHISSSSSKEAKEMPPLGDLAISSNEAKQVQIGSGNKPENVTNRKPDTASRSTDTLTLERNSVTDKV |
| AT5G19280 | MGYLDLALSYSNQPQTVEAPASGGGLSQNGKFSYGYASSAGKRSSMEDFFETRIDGINGEIVGLFGVFDGHGGARAAEYVKRHLFSNLITHPKFISDTKSAITDAYNHTDSELLKSENSHNRDAGSTASTAILVGDRLVVANVGDSRAVISRGGKAIAVSRDHKPDQSDERERIENAGGFVMWAGTWRVGGVLAVSRAFGDRLLKQYVVADPEIQEEKIDDTLEFLILASDGLWDVFSNEAAVAMVKEVEDPEDSAKKLVGEAIKRGSADNITCVVVRFLEKKSASSSHISSSSSKEAKE MPPLGDLAISSNEAKQVQIGSGNKPENVTNRKPDTASRSTDTLTLERNSVTDKV |
| AT5G24940 | MGYMDLALSYSNQMRIVEAPASGGGLSQNGKFSYGYASSAGKRSSMEDFFETRIDGIDGEIVGLFGVFDGHGGSRAAEYVKRHLFSNLITHPKFISDTKSAIADAYTHTDSELLKSENSHTRDAGSTASTAILVGDRLLVANVGDSRAVICRGGNAFAVSRDHKPDQSDERERIENAGGFVMWAGTWRVGGVLAVSRAFGDRLLKQYVVADPEIQEEKIDDSLEFLILASDGLWDVFSNEEAVAVVKEVEDPEESTKKLVGEAIKRGSADNITCVVVRFLESKSANNNGSSSSEEANQVPTAVRNDSDHKISAKETNQDHTTVNKDLDRNTDSQSLNQKPIAARSADNSNQKPIATTATGHSVSSEQSGLTGEKSQMPIKIRSDSEPKSSAKVPNQTQSTVHNDLDSSTAKKPAATEQSGSTGERNRKPIKVHSDSAARKTTPSIFN |
| AT5G26010 | MGHCFSLPSSQSEIHEDNEHGDGNVVCYGEEFGLDQDLPVHRLGSVCSIQGTKVLNQDHAVLYQGYGTRDTELCGVFDGHGKNGHMVSKMVRNRLPSVLLALKEELNQESNVCEEEASKWEKACFTAFRLIDRELNLQVFNCSFSGSTGVVAITQGDDLVIANLGDSRAVLGTMTEDGEIKAVQLTSDLTPDVPSEAERIRMCKGRVFAMKTEPSSQRVWLPNQNIPGLAMSRAFGDFRLKDHGVIAVPEISQHRITSKDQFLVLATDGVWDMLSNDEVVSLIWSSGKKQASAAKMVAEA AEAAWKKRLKYTKVDDITVICLFLQNKEQPS |
| AT5G27930 | MGHFSSMFNGLARSFSIKKVKNNNGNCDAKEAADEMASEAKKKELILKSSGYVNVQGSNNLASLFSKRGEKGVNQDCALVWEGFGCQEDMIFCGIFDGHGPWGHYVAKQVRNSMPLSLLCNWQKILAQATLEPELDLEGSNKKISRFDIWKQSYLKTCATVDQELEHHRKIDSYYSGTTALTIVRQGEVIYVANVGDSRAVLAMESDEGSLVAVQLTLDFKPNLPQEKERIIGCKGRVFCLDDEPGVHRVWQPDAETPGLAMSRAFGDYCIKEYGLVSVPEVTQRHISTKDHFIILASDGIWDVISNQEAIEIVSSTAERPKAAKRLVEQAVRAWKKKRRGYSMDDMSVVCLFLHSSSSSSLSQHHHAMTILK |
| AT5G36250 | MGSCLSSSGGGGSRRSLHGSPHVPGPGRRKRPPKRRPGSCSSSFDNTEEPLLHRIPGRMFLNGSTDTVSLFSQQGKKGPNQDAMIVWENFGSMEDTVFCGVFDGHGPYGHIVAKRVRDLLPLKLGSHLESYVSPEEVLKEISLNTDDRKISEDLVHISANGESRVYNKDYVKDQDMIQMLIGSIVKAYRFMDKELKMQVDVDCFCSGTTAVTMVKQGQHLVIGNIGDSRAVLGVRNKDNKLVPFQLTEDLKPDVPAEAERIKRCRGRIFALRDEPGVARLWLPNHNSPGLAMARAFGDFCLKDFGLISVPDVSYRRLTEKDEFVVLATDGIWDALTNEEVVKIVAKAPTRSSAGRALVEAAVRNWRWKFPTSKVDDCAVVCLFLDSEPNRLSTASFSKEKHINNGVTEPEPDTASSSTPDSGTGSPELNGVNRIDTLVNLPVYVPTKE |
| AT5G51760 | MTEIYRTISTGRGDDVSPTKCRERRRRRIEMRRQAAVFGEPSSSRNRDRTDMEVYSSFDVPLRKQARRSEIGGLPADIGGFLAPPAASSCQKSEAPVWKGEETEDEPLYGIVSVMGRSRKMEDSVTVKPNLCKPEVNRQRPVHFFAVYDGHGGSQVSTLCSTTMHTFVKEELEQNLEEEEEGSENDVVERKWRGVMKRSFKRMDEMATSTCVCGTSVPLCNCDPREAAISGSTAVTAVLTHDHIIVANTGDSRAVLCRNGMAIPLSNDHKPDRPDERARIEAAGGRVLVVDGARVEGILATSRAIGDRYLKPMVAWEPEVTFMRRESGDECLVLASDGLWDVLSSQLACDIARFCLREETPSSLDLNRMAQEDDNDGEQNPSRSVLAATLLTRLALGRQSSDNISVVVIDLKNSSQ |
| AT5G53140 | MVCSSFIRSFIVQAGCRIGVLAQGRHQFIHIKKTLSVGFGFRTSVIGFRTTSGIGFRTSAKMMVDTSAGEKRISLVDMPPEKVDDGGYIGGGWKNDDGSLSCGYCSFRGKRSTMEDFYDIKASTIEGQAVCMFGIFDGHGGSRAAEYLKEHLFNNLMKHPQFLTDTKLALNETYKQTDVAFLESEKDTYRDDGSTASAAVLVGNHLYVANVGDSRTIVSKAGKAIALSDDHKPNRSDERKRIESAGGVIMWAGTWRVGGVLAMSRAFGNRMLKQFVVAEPEIQDLEIDHEAELLVLASDGLWDVVPNEDAVALAQSEEEPEAAARKLTDTAFSRGSADNITCIVVKFRHDKTESPKIETNAMAESEPELNPTTELEPESNPSTALETESIPKAELESEPDAIPDPKPETEPETKGEKAGE |
| AT5G57050 | MDEVSPAVAVPFRPFTDPHAGLRGYCNGESRVTLPESSCSGDGAMKDSSFEINTRQDSLTSSSSAMAGVDISAGDEINGSDEFDPRSMNQSEKKVLSRTESRSLFEFKCVPLYGVTSICGRRPEMEDSVSTIPRFLQVSSSSLLDGRVTNGFNPHLSAHFFGVYDGHGGSQVANYCRERMHLALTEEIVKEKPEFCDGDTWQEKWKKALFNSFMRVDSEIETVAHAPETVGSTSVVAVVFPTHIFVANCGDSRAVLCRGKTPLALSVDHKPDRDDEAARIEAAGGKVIRWNGARVFGVLAMSRSIGDRYLKPSVIPDPEVTSVRRVKEDD CLILASDGLWDVMTNEEVCDLARKRILLWHKKNAMAGEALLPAEKRGEGKDPAAMSAAEYLSKMALQKGSKDNISVVVVDLKGIRKFKSKSLN |
| AT5G59220 | MAEICYENETMMIETTATVVKKATTTTRRRERSSSQAARRRRMEIRRFKFVSGEQEPVFVDGDLQRRRRRESTVAASTSTVFYETAKEVVVLCESLSSTVVALPDPEAYPKYGVASVCGRRREMEDAVAVHPFFSRHQTEYSSTGFHYCGVYDGHGCSHVAMKCRERLHELVREEFEADADWEKSMARSFTRMDMEVVALNADGAAKCRCELQRPDCDAVGSTAVVSVLTPEKIIVANCGDSRAVLCRNGKAIALSSDHKPDRPDELDRIQAAGGRVIYWDGPRVLGVLAMSRAIGDNYLKPYVISRPEV TVTDRANGDDFLILASDGLWDVVSNETACSVVRMCLRGKVNGQVSSSPEREMTGVGAGNVVVGGGDLPDKACEEASLLLTRLALARQSSDNVSVVVVDLRDT |
| AT5G66080 | MLSLFFNFLTSCLWPSSSTTSHTYSDSKGKQDGLLWYKDSAHHLFGDFSMAVVQANNLLEDQSQVESGPLTTLSSSGPYGTFVGVYDGHGGPETSRFVNDHLFHHLKRFAAEQDSMSVDVIRKAYEATEEGFLGVVAKQWAVKPHIAAVGSCCLIGVVCDGKLYVANVGDSRAVLGKVIKATGEVNALQLSAEHNVSIESVRQEMHSLHPDDSHIVVLKHNVWRVKGIIQVSRSIGDVYLKKSEFNKEPLYTKYRLREPMKRPILSWEPSITVHDLQPDDQFLIFASDGLWEQLSNQEAVEIVQNHPRNGIARRLVKAALQEAAKKREMRYSDLNKIERGVRRHFHDDITVVVLFLDTNLLSRASSLKTPSVSIRGGGITLPKKL |
| AT5G66720 | MSATALSRLNPVSQFGFQRIVAGKSKSFFSNSGQRRLFSDSSRFRQAMAASGSLPVFGDACLDDLVTTCSNGLDFTKKRSSGGSFTINCPVASMRLGKRGGMMKNRLVCHYSVVDPLEKSRALFGTLSKSVHTSPMACFSVGPAHELSSLNGGSQESPPTTTTSLKSLRLVSGSCYLPHPEKEATGGEDAHFICDEEQAIGVADGVGGWAEVGVNAGLFSRELMSYSVSAIQEQHKGSSIDPLVVLEKAHSQTKAKGSSTACIIVLKDKGLHAINLGDSGFTVVREGTTVFQSPVQQHGFNFTYQLESGN SADVPSSGQV FTIDVQSGDVIVAGTDGVYDNLYNEEITGVVVSSVRAGLDPKGTAQKIAELARQRAVDKKRQSPFATAAQEAGYRYYGGKLDDITAVVSYVTSS |
| Os01g07090 | MAASSTATRLSPPRLHAPTTPSPHLPLRRSRFSPLRAAKLEAVLTIGTHLIPHPRKAETGGEDAFFVNGDDGGVFAVADGVSGWAEKDVNPALFSRELMAHTSTFLKDEEVNHDPQLLLMKAHAATTSVGSATVIIAMLEKTGILKIASVGDCGLKVIRKGQVMFSTCPQEHYFDCPYQLSSEAIGQTYLDALVCTVNLMEGDMIVSGSDGFFDNIFDQEIVSVISESPGVDEAAKALAELARKHSVDVTFDSPYSMEARSRGFDVPSWKKFIGGKLIGGKMDDITVIVAQVKAVMIPDDEGVDEEKGQGDEQGSAVAVASSEQKEDSITT |
| Os01g19130 | MVAGAEVMHQVVPLLEASFHRRCSVKGVDEVSPPVEEMSPEAASEAAIEVPELMVKAPVESLQFSPNIRSGSFADIGPRRYMEDEHIRIDDLSGHLGSLLMCPAPNAFYGVFDGHGGPDAAAYMKRHAIRLFFEDSEFPQALEEDESFYESVEKSIHNAFLSADLALADDLAISRSSGTTALAALIFGRQLLVANAGDCRAVLCRKGVAVEMSRDHRPTY DAEHERITECGGYIEDGYLNGVLSVTRALGDWDMKMPQGSRSPLIAEPEFQQTTLTEDDEFLIIGCDGIWDVMSSQHAVTIVRKGLRRHDDPERCARELAMEAKRLQTFDNLTVIVICFGSELGGGSPSSEQAPIRRVRCCKSLSSEALCNLKKWLEPNE |
| Os01g32964 | MSSPSPSSEAAAAHHHHHQRRQHAGAAGGSGLVPLAALIKEEARAERPMGSGSRICARDEEDGGGGGGAEGGRRWRRPLLRYGCAAQSKKGEDFFLLRTDCARPSTSSSSSSSLASSPPHTFAVFAVLDGHNGNAAAIYTRDNLLNHVLSAMPRGLSREEWLHALPRALVAGFVKTDKEFQHKGQTSGTTATFVIIDGWTITVASVGDSRCILDAQGGAVSLLTVDHRLEENVEERERVTASGGEVGRLSVVGGAEIGPLRCWPGGLCLSRSIGDIDVGEFIVPVPYVKQVKLSNAGGRLIIASDGIWDALSSEAAAKCCRGLPAELAAKQVVKEALRTRGLKDDTTCIVVDMIPPDQTIRHPSPPKKINKLKSLIFRKKTKDHPNKLTKQLSAAGMVEELFEEGSAMLSERLGNDSSGRRTSSSLFTCAICQVDLEPSEGISVHAGSIFSSSSSKPWEGPFLCSDCRDKKDAMEGKRPSGVKVL |
| Os01g36080 | MGVEVPPEESNRCVRGCCRSAAIPLHLPPSSFSLLSPIAKGSESTVYEARLGGERVAAKKPVLSTSDDLDKFHYQLQLLWWVLPIELDHPGLARLVAAHARPPNYLMFFDFFEPPNLADKIHVEEWNPSVQQVVTIATDLAKALQYLNILGIVHRDIKPANILIDKDFHPHLADFGLAMYQKDIKHVSVENWRSSGKPTGGFHKKNMVGTLIYMAPEILR KDIHTEKSDVYSFAISINELLTGVVPYTDLRAEAQAHTVLEMTYTEQQLTAAIVSQGLRPALALPESGAPPSLLSLIQRCWDSDPQQRPSFKDITEELKIIEKHIAVNSCSLASPANKSQNGNTEVHHYQEALSWLNQGELFAKGNKLDSTVDHWSDIFDQSSKYCPTLSWGSFATCGRRETMEDTHFMLPHMSEEKDLHAFGIFDGHRGSAAAEFSVRAVPGFLKQFNSNTSPTDALTEAFVRTDIAFREELILHQKSKRITQKNWHPGCTAVTALIVRNKLFVANAGDCRAILNRAGEPFPMTRDHVASCPKERERIVKEGTEVKWQIDTWRVGAAALQVTRSIGDDDLKPAVTAQPEVIETILSPDDEFLVMASDGLWDVMSNEDVLSIIKDTVKEPGMCSKRLATEAAARGSKDNITVIVVFLRPVSTAERIY |
| Os01g37130 | MALLSPRVPRLPLASASAAGAGLRCCVSGGRAGSAAWCHASAAGSVASSSSELEAIRWGTAKLQGARDEMEDEVVLRPGSLLDGFSFAAVFDGHAGFSAVEFLRDELYKECAAALDGGAVLSTKNLDAITDSIQRAFATVDANLSTWLEQMDKEDESGATATAMFLRNDVLVVSHIGDSCLQVVSRGGRPQAVTNFHRPYGNKKASLEEVKRIRAAGGWIVDGRICGEISVSRAFGDIRFKTRKNEMLVKGVKEGRWTEKFISRINFKGDLIVSSPDVSLVELGPDVEFVLLATDGLWDYIKSSEAVALVRDQLRQHGDVQVACEALGQIALDRRSQDNISIVIADLGRTNWKELPAQRPNLFLELTQAVATVGAVSLGIYISSLLALQ |
| Os01g40094 | MEDVAVAAALAPAPATAPVFSPAAAGLTLIAAAAADPIAAVVAGAMDGVVTVPPVRTASAVEDDAVAPGRGEEGGEASAVGSPCSVTSDCSSVASADFEGVGLGFFGAAADGGAAMVFEDSAASAATVEAEARVAAGARSVFAVECVPLWGHKSICGRRPEMEDAVVAVSRFFDIPLWMLTGNSVVDGLDPMSFRLPAHFFGVYDGHGGAQVANYCRERLHAALVEELSRIEGSVSGANLGSVEFKKKWEQAFVDCFSRVDEEVGGNASRGEAVAPETVGSTAVVAVICSSHIIVANCGDSRAVLCRGKQPVPLSVDHKPNREDEYARIEAEGGKVIQWNGYRVFGVLAMSRSIGDRYLKPWIIPVPEITIVPRAKDDECLVLASDGLWDVMSNEEVCDVARKRILLWHKKNGTNPASAPRSGDSSDPAAEAAAECLSKLALQKGSKDNISVIVVDLKAHRKFKSKS |
| Os01g43100 | MAAHGGGGVEEDQAGSSSLCPPAAEAEAAAAAAAIARAARPPRPGRDKRLGVRHPLKHRRFRAGGKAAVAAGAREVGEATTVAEATATGPPKGSDEDDEARYICGGWTSDDGRMSCGYSSFRGRRANMEDFYDIKSSKVDDNQINLFGIFDGHGGSHAAEHLKKHLFENLLKHPSFITDTKSAISETYRKTDSDFLDAETNINREDGSTASTAIFVGNHIYVANVGDSRTVMSKAGKAIALSSDHKPNRKDERKRIENAGGVVTWSGTWRVGGVLAMSRAFGNRFLKRFVVAEPEVQEQEIDDDLEFLILASDGLWDVVSNEHAVAFVKAEEGPEAAARKLAEIAFARGSTDNITCIVVKFLHAKMAVDAASSSERS |
| Os01g46760 | MSSDTSRRDHAAMAVREVLAGDRKVGTVSRSARRRRLELRRLGRTASAVAEDDAAKRVRPASDSSSDSSESAKVAPEPTAEVARWPACVSHGAVSVIGRRREMEDAIFVAAPFLAASKEAAVEGSGVAEEEGKEEDEGFFAVYDGHGGSRVAEACRERMHVVLAEEVRVRRLLQGGGGGADVEDEDRARWKEAMAACFTRVDGEVGGAEEADTGEQTVGSTAVVAVVGPRRIVVANCGDSRAVLSRGGVAVPLSSDHKPDRPDEMERVEAAGGRVINWNGYRILGVLATSRSIGDYYLKPYVIAEPEVTVMDRTDKDEFLILASDGLWDVVSNDVACKIARNCLSGRAASKYPESVSGSTAADAAALLVELAISRGSKDNISVVVVELRRLRSRTTASKENGR |
| Os01g62760 | MAEICCEEAMSPPATATAAVAAAVSASAAAAVSSAIDRRRRRMEMRRIRIASDLELQAGEDGRPGKRQRLARTASGAPRPDEDSASERPSCGRTEEFPRYGVTAVCGRRREMEDAVSIRPDFLPASGKFHFYGVFDGHGCSHVATTCQDRMHEIVAEEHNKGASGEVAPWRDVMEKSFARMDGEVGNRASTRSDDEPACPCEQQTPSRRDHAGSTAVVAVVSPTQVVVANAGDSRAVISRAGVPVALSVDHKPDRPDELERIEAAGGRVIYWDGARVLGVLAMSRAIGDGYLKPYVTSEPEVTVTERTDDDECLILASDGLWDVVTNEMACEVVRACFHNNGPPAPAARPSGVPSSAEAAETENGGAASVKGISKAESSDKACSDAAMLLTKLALARRSADNVSVVVVDLRRGL |
| Os01g74530 | MVGQTVMRIVRPCFKPDHQLAVGGTRDGLLWYKDTGRHACGDFSMALVQANNLLEDASQVEAAPLLLSHSSSTTFVGIYDGHGGPETAHFIAQHFFPNLKKFATEQQTVSVDVIRKSYAATEEGFLNLVRKQWLIKPQLASVGSCCLVGIINEGVLYVANTGDSRAVLGRLERGVIKAVQLSAEHNASIESVREELRQFHPDDPRIVVLKHNVWRVKGLIQVSRTLGDAYLKSTEFNREPLLARFRLSEPFHKPILSPEPSIEVHKLCTEDQFVIFASDGLWEHLTNQEAVDIVNCAPRNGIARRLIKAALREAAKKREMRYSDLKKIDRGVRRHFHDDITVVVLFLDSALVGKRFYGGPLLSLRGGDGASTLTQK |
| Os02g05630 | MHGRPSRPLASSSSSSSSRVFSFFLAPRVFLFLVVVVVVVFLPGRSSCWWLEGTEELEEEMGFAGDCSPVSGGGLSENGKFSYGYASAPGKRASMEDFYETRIDGVDGETIGLFGVFDGHGGARAAEYVKQHLFSNLIKHPKFISDIKSAIAETYNHTDSEFLKAESSHTRDAGSTASTAILVGDRLLVANVGDSRAVVCRGGDAIAVSRDHKPDQSDERQRIEDAGGFVMWAGTWRVGGVLAVSRAFGDKLLKQYVVADPEIKEEIVDSSLEFLILASDGLWDVVSNKEAVDMVRPIQDPEQAAKRLLQEAYQRGSADNITVVIVRFLEGTTTGGGPSREAASDQNS |
| Os02g08364 | MGIYLSTPKTDKFSEDGENDKLKLGLSSMQGWRANMEDAHSALLNLDNETSFFGVFDGHGGRVVAKFCAKYLHSQVLRSEAYSAGDLGTAVHRAFFRMDEMMRGQRGWRELSALGDKINKIGGMIEGLIWSPRGSDSNNGQDDWSFEEGPHSDFAGPTCGCTACVALIRNNQLVVANAGDSRCVISRAGQAYNLSRDHKPELEAERDRIVKAGGFIHMGRINGSLNLTRAIGDMEFKQNKFLPPEKQIVTANPDINVVELCDDDDFLVLACDGIWDCMSSQQLVDFIHEHIQKESSLSAVCERVLDRCLAPSTIGGEGCDNMTMVLVQFKKPITQNKKADVGEQSVKGVEEAEINAAEENGS |
| Os02g13100 | MGICASSEQLEHVHETDESIVYVKDEQGRGGRGVESGGARKVASLFSQRGKKGPNQDSVILCQGFGMEDGVFCGVFDGHGRCGQFISKLVRDYLPFMILSHRNALLLADAAADDDDDAAFSDDAAASSSADSSGNSSPQPSASASAQMLEEWRQACASAFAAMDGELKLQPNLDCAFSGTTAVCAIKQGRDLIIANLGDSRAVLATMSDTGYLQAVQLTVDHKPSVPEEAARIKRSGGRVFGLKDEPGVMRVWLPGENSPGLAMARSLGDMRLKRHGVIPAPEVTSRRVTGADLFMVLATDGVWDVLSNEEVVSIVCATPRKQHASKAVVEAAVQRWRAKFPTSRVDDCSAVCLFLHDHTLGTAAAASAAAAAAARKARRASTATPPAS |
| Os02g15594 | MVCFASLRRALPLLLRATTTTTPRFLLPRALSGGVGGGAAVDARALLRGHSGWRGLRVAARMMLDSSDSAAAAGQMQPQQRAAGAVACSAQDGGAAGYASGGWAREDGKLKCGYSSFRGKRATMEDFYDVKLTEIDGQAVSLFGVFDGHGGPRAAEYLKENLFENLLKHPEFLTDTKLAISETYQKTDTDFLESESNAFRDDGSTASTAVLVGGHLYVANVGDSRAVVSKAGKAMALSEDHKPNRSDERKRIENAGGVVIWAGTWRVGGVLAMSRAFGNRLLKPFVVAEPEIQEELVNEDLECLVLASDGLWDVVENEEAVSLAKTEDLPESVARKLTEIAYSRGSADNITCIVVQFHHDKTE |
| Os02g17970 | MGCSPSKVCSCPHYKGSLCFCDCGCFGQTPDSPRESRGKSNRVRGKTDSSASDASSDDLEEDDDGLHQMNITRDSNVGINRLSRVSSQFLPPEGSRKVRIPLGNYDLRYSYLSQRGYYPESLDKPNQDSFCIHTPFGTSPDDHFFGVFDGHGEYGAQCSQFVKRRLCENLLRDDRFRTDVVQALHSAFLATNSQLHADSLDDSMSGTTAVTVLVRGKTIYIANTGDSRAVIAEKRGEDVVAVDLSIDQTPYRTDELERVKECGARVMTLDQIEGLKNPDVQCWGTEESDDGDPPRLWVQNGMYPGTAFTRSIGDSVAESIGVVANPEIFILELNANHPFFVLASDGVFEFLSSQTVVDMIAKYKDPRDACAAIVAESYRLWLQYETRTDDITIIVVHINGLTDMECTQTVMKVSLQPSQQVVELVGSESPSTISLNPKNQRSRQDLSRARLRALESSLENGRLWVPPSPSHRKTWEEQAHIERILHDHFLFRKLTDSQCHVLLDCMQRVEVKAGDIVVQQGGEGECFYVVGSGEFEVLAIQQEEDGKEVTKVLHRYTADKLSSFGELALMYNKPLQASVRAVTTGTLWALKREDFRGILMSEFSNIPSLKLLRSVELFTRLTMLQLSQLADSLVEVTFGDGQMIVDKNDDASSLYIIQRGRVKLKLAADQVNSDAWDLLSSQTKVAQSSREDGNYVFEIDEGGHFGEWALFGETIAFTAMSVGDVTCSTIAKEKFDSIIGPLPKVSQSDSKLKDSLVPKGHGADDSSFRKAQLSDLEWKMCIYAADCSEIGLVQLRGSDKIKSLKRFYIKRVKDLHKEKHVFDEKDLMKSLSQSTCVPEVLCTCADQSYLGILLNCCLCCSLASILHAPLNESSARFYAASVVVALENLHQRSILYRGVSADILMVDRSGHLQLVDFRFAKKLQGERTYTICGIADSLAPEIVLGRGHGFSADWWALGVLIYFMLQSDMPFGSWRESELEPFAKIAKGHLVMPSTFSIEVVDLITKLLEVNENARLGAKGAESVKRHPWFDGIDWKQIADGTYTVPQEITDRVDSYVETLTEDLTASPSMPSEETADQAAPEWIQDW |
| Os02g27220 | MVEAAAGRRSGANRRRPSGGGERRRQQQQHQRLVAVAVAARVVMVAPAATPAPAAGGGGGCVEDILGCLLGVLRALGVTWAAAARPQRQQPRLAAQTPRGPAPGADGRRAAAELRGIPGRIAGNGACAVASLYTLQGKKGVNQDAMIVWENFCSREDTIFCGVFDGHGPNGHLVAKRVRDLLPIKLGADLGTDEGRQTSTSSIKSNGDETGSPGNMGRDAEQNGEYPEIFTALRTSFLRAFNVMDRDLKLHKSIDCFFSGTTAVAVLKQGRNLIIGNLGDSRAILGTRDKDNQLMAVQLTVDLKPNIPSEAQRIRQRRGRIFALPEEPEVARVWLPKYNSPGLAMARAFGDFCLKDYGLISMPEVSYHRITEKDEFVVLATDGVWDVLSNTEVVSIVNRATSRASAARLLVESAHRAWRARFPTSKIDDCAVVCLFLDTDELSETSSSMARDMTNAVEVSSGQHSNTIQLSTGVSSDVVTAVLTDGDDLSAVDAVAKLVTLTDLPNNASGATQSITTK |
| Os02g35910 | MSTRSKSVPVPAGGGAATVPLAVLLRREVVSEKTAAERPELQVGLFSQAKKGEDYTFLKPDCERLPGVPSSSFSAFGLFDGHNGNGAAIYTKENLLSNILTAIPADLNREDWLAALPRAMVAAFVKTDKDFQTKARSSGTTVTFVIIDGLFITVASVGDSRCVLEAEGSIYHLSADHRFDASKEEVDRVTESGGDVGRLNVVGGAEIGPLRCWPGGLCLSRSIGDQDVGQFIVPVPYVKQVKLSTAGGRLIISSDGVWDVLTAEVAFNCSRTLPPEAAAEQIVKEAVQQKGLRDDTTCIVVDILPDKANLTMPHTKKQPGMGVFKNMFRKKTPSDSSSHTDREYMDPDIVEEIFEDGCAFLSKRLDSEYPVRNMFKLFICAICQVELKPSQGISVHEDSSQPGNLRRWDGPFLCQGCQEKKEAMEGKRRSRDSSSRNSGSSE |
| Os02g38580 | MGNSLPVESKFTFEEENDRIKYVVSSMQGWGEKMEDAHAAILNLDDATSTSFFGVYDGHGGAEVALYCAKQFHIELCNHEDYHNDLINALDNVFLSMDENLQQSDAWRELVIPHDNGCMYFLKAGVCAKPFPQATYTGPAYEGSTACVVVIRGNQMIVGHVGDSRCVLSRQGGLAIDLSFDHKPCTRTESERERVQNAGGRSLGLRCEQVMGNYVVKEQWVLGDFGGGVTISRSIGDFAFKKNKDLDREKQMLVCDPDILADDITDDMEFLVIASQGLWSCVDSADVVSYIHDRLSVEGAELRVICEEVVEFGLASGENTTVILVQFKPGAFQYQLVDPAGFGTAVSNIASTSAAPAGASDTSDEGVMADSCATADTSGSARAESGELVPTPSANNTVTDEVDPTGTVAADDKVDPNSSANADADDGAPKPSLGAVIESDEVALDATATGHQVAVRQQEEFDPKKCWICGKGYKKILLEPSSARARNPLLAHAKTCESEDKKAKKKITKYMMKANVTNQYH |
| Os02g38690 | MNSFLFSALFFFWLLFCSCKASSAMGNSLPVESKFTFEEENDRIKYVVSSMQGWGEKMEDAHAAILNLDDTTSTSFFGVYDGHGGAEVALYCAKQFHIELCNHEDYHNDLINALDNVFLSMDENLQQSDAWRELVIPHDNGCMYFLKAGVCAKPFPQATYTGPAYEGSTACVVVIRGNQMIVGHVGDSRCVLSRQGGLAIDLSFDHKPCTRTESERERVQNAGGRSLGLRCEQVMGNYVVKEQWVLGDFGGGDFAFKKNKDLDREKQMLVCDPDILADDITDDMEFLVIASQGLWSCVDSADVVSYIHDRLSVEGAELRVICEEVVEFGLASGENTTVILVQFKPGAFQYQLVDPAGFGTAVSNIASTSAAPAGASDTSDEGVDDAATARPTVMGYDADSSTGSADATVDSDEVDPNATADSYNPRGHAEIVASHTDDEVYTSGSARVESGELAVPTPSANNTVADEVKVDAAVVAGGSTTAMAADEATVVSLLSTIVDNYYSINTSEEPARRLIPLPLSQPTTRFINININAENARMTYIVKRREYFLVKSSCRCGGRHQQREVTSGGAGVEHGSIGDVPPSIRRAGRWMEKGRRRGRRAGSKYAKKEEEEGASCHAGCRHFVPEIGPWSEATIPENPRLPNSDENDMDAGGEEAKQERHLVLAHELFLLSRPDLDDLANVALRSDALDAVKSDGMAPLFESLATAGVLLKPDDAAARRDARADRRGGPQARRE |
| Os02g38710 | MGPLLERWISREGRSDGGDASGPVLFWCVLIIFAVPDAIRSSSLRLGQVAPRLVLFSNFKAFRSPKFAKILPGSDLFSPRVLGFRTRVVLLLDVFEVGFALFCKASSTMGNSLPVESKVTVEEENDRIKYIVSSMQGLGHKMEDAHAAILSLDDTTSTSFFGVYDGHGGAEVASYCAKRFHIELCNHEDYHNDLTNALDNVFFSMDENLQQSDAWRELVIPRDNGWMYFLKAGVCANFWPFPQAYTGPAYEGSTACVVVIRGDQMIVGHAGDSRCVLSRQGGLAIDLSSDHKPRTSESERERVQNAGGISLGVDCEKVMENYVIKEQWILGYFGESVTISRSIGDFAFKQNKDLNREEQMLICDPDIHTHDITGDMEFLVIASQGLWSCMESADVVAYIHVRLLEGVELRVICEELVQSGLASGENTTVILVQFKPGAFQFQYELVDPAAFDTAASNVASTSAGPAGGSDSDTSATSDEGVDDTATAGTTTTGYEAGSSTGPGSGGGSANAAFDSGGDLAANLDIATNFGSDDLAANLDIATDIDTEDVFTFINSDDTFGINSDEVELDPNFRPKPQVRRAHDGPSPTPSEIEADLNASPTRYNMRDIFEAFDKVEAELGGFPLQGHDVSSTSTNPNTATDTGSGSRTGDDDVDGAIARAMAVASSVMTGAGYEVDSTTTNPSAAADTGSYTGDEIKVDDSTSGSARGDSGELVNNDTTVADNNASGVADSTTVGDEVDPTATVAADDSNTGDKVDPPAITKATADSNTSGEVDVDATATATASASAAVADDEGTAPDDSEGSP |
| Os02g38780 | MDAGGEEEKQERHRVDGASPSMVALRSDVLHAVLGGKSHNQDTSELVTLSEWSKNKFPLYAYAAAFMFLVMQEYDKRSHGIDFLNRDFVVRDLGMPSLNKHFPVKEFYCSYRRKSGHSTYTYGFFMLQKLILIQNAHIRREEEEEGACSMVTTSHRTGHSRSVGLAARDDPTAEMRLVQGEGSVGTTSHRMGALLERWISREGRTDGGDTSVQGERSVGTTSHRMGALLDVGSAVDDPAKQRSAMGNSLPVESKFTDEKENDRIKYVVSSMQGWGEKMEDAHAAILNLDDTMTSFFGVYDGHGGAEVASYCAKRFHIELCNHEDYDSNLSNAMRSAFYSMDEDLQLSDAWRELVIPRNNGWMYFLKAAACTSICKATYTEPAYEGSTACVVVIRGNQLIVGHAGDSRCVLSRNGQASALSVDHKPDRDFACKKNERLPPEDQMLTCNPDILTMDITDDMEFLVIATEGLWCNMTNQNVVDHTHDRLLEGAEARVICEELVQFGLPSGDNTTVILVLFKPGAYPAVPPVDTDTDTDSHTGDDVDNNDPANEVDPTANAGSDDSNTSDEVKVDATATAVGSSSTTAVAADEATVVSLSTATITIVDNYFFINTSEEVDPTANAGSDDSNTGDEVKVDATASSGLSDWELIYSEF |
| Os02g38804 | MWVMQGERRRARAPWGPPDTGGALLERWISRERRSDSRDASGSAKQRSAMGNSLPVESKFTDEKENDRIKYVVSSMQGWGEKMEDAHAAILNLDDTMTSFFGVYDGHGGAEVASYCAKRFHIELCNHEDYDSNLSNAMRSAFYSMDEDLQLSDAWRELVIPRNNGWMYFIKAGVCANLSPFPQATYTAPSYEGSTACVVVIRGDQLIVGHAGDSRCVLSRNGQASALSVDHKPDSESERERVQNAGGVAVGYSYRKIMGRWVTKKQWGFTDFKGRVSISRSIGDFACKKNERLPPEDQMLTCNPDILTMDITDDMEFLVIATEGLWCNMTNQNVVDHTHDRLLEGAEARVICEELVQFGLPSGDNTTVILVLFKPGAFPAVPPVDTDTDTDSHIDDDVDPTGSNNATASDNNDPANEVDPTANAGSDDSNTGDEVKVDATATAVGSSSTTAVAADEGTGNPPHGALVDTDDEDGLTYSQDMDLPPASTSPPTFPDEDDLPRSNPDKSPPHDDTYHRW |
| Os02g39410 | MGASPSRPLEQSPSSSEGENHRVKYASYTTQGFRPHMEDALAVELDLDATTSFFGVYDGHGGAEVAMYCAKRFHTMLLEDVDYINNLPNAITSVCFRLDDDLQRSNEWRESLNPCANRNCLTNICANLHHFTEDYVPPSYEGSTACVVIIRGNQIIVGNVGDSRCVLSKNGQAISLSFDHKPHHEAERERIQRAGGHVFLQRILGMLATSRAIGDFAYKQNRNMPPSQQMVTCVPDIRVENITDDTEFLVIASDGVWDGMRNNNVVQFVRQELRPGEENLRETCEKLVGHCLHSNDNATAILVKFKPIEEDPDEVASARDEHQHNPEGGDEKLDINNDND |
| Os02g39470 | MAAARLPSPAPVAHPPALRARPLAPHLYAALPRAPYPAWQWRGARAASPARREAVVRGAAVAEDEGVELADDDDGERGVGGGDGGSESFRTPARHPPAVAFAIGVGSHGIMVICWIGGGANEHPQPHGDLRFVSLSIFPFIDVTYLYNVVFTLVFFRKASLAMGASASSSVTSKLTNDGENQRVKYASSTMQGYCPTMQDALAVELDLDALRNTSFFGVYDGDGGAEVAMYCAKRFHAMLCEDENYLNNLPNAITSVCSRLDDDLQRSNEWKESLYPRGNGECFQFLKTGVCANLWHSEELGFMLPQQAYRAPLYEGSTACVVIIRGNQITVGNVGDSRCVVSHNGQAIDLSIDHKPTVGSERERILRAGGRVLVKRIPVMGSDGRLMRGWGYFELKKNQNIPASQQMVTCDPEFTIVDITADTEFLVIATDGIWGHMSSQDVVDFIRKELHSGEENLRAICEKLLDHCLTSRDNVTVILVRFKPGAAVIPILSDIDEEPVLSDVEEEPHEPQHNPGDGGGGQQDIGGESEELPLAHFPQEYLLPVYGDHPLLTGAVVQEPLPLPEGGDHPVPPKKKIKVAPLLPERADQPVVTSNSATTTRPQLCAPYDDEIEATLRAMETNPAERPSPYFLETTQGGRMTALVRASMIAFMDEFSRFHELADGTLQRAAYFLDRYLSVTPESDDALQLRLVGATAVFLAAKYEDQYTLRKIDASMVAARRGYTSETRHKMVSIMETEMLAALGFNLGGPTAYTFVEHFTRYYGDGEEEELLKEAAHRFADGSLLTYGFHRYLPSIVAASSIFLARLDVLGHEPWSQDLAELTGYKAIDLMGCGYYRSLSVSNSPSPCANPGNYIALVTLRVAEEMRPPLPPPARAERHGTGTVRVTRVKLLKPRDTLLLGQAYRLITVDEVTRVLQAKKEEKSRRAAAQHHLESKPAGAAGVGINSSGDDHTRPIRPAHLYIVSCRCSW |
| Os02g39480 | MVISVPLFSSVLLALVVAVPADFDVGGRLLGVGLCSVRGDPNEHYDPHGDLYLRPFLQLDSVHQFYSLVFARKASSAMGASTSTKRPLTSKVTNEGENDRVKYASSAMQGLRMSMQDALAVELDLDALKSTSFFGVYDGHGGAEVAMYCAKRFHVMLREEESFLNNLSYAITSVCSRLDDELEAPNVWRASLYPHRSSESSSESSDCFQFLSTGSCANVWRSSEAVSYKLPSYEGSTACVVIIRGNQITVGNVGDSRCVLSKNGQAIDLSTDHKPNVPLERQRILRVGGQVWREKFPAKDSGGEIREQWGPYCIEGKLSTSRALAGIFLTTISGDFAYKNIVYRPQYQMVTHFPDIRVAKITGDTEFLVIASDGICSIQILIVDLNTFFPFRDHMSSQDVVDFVHEKLNSRRQELCQSLINQGKKRECFTEDSQLATNKNIAPNTTTLGEETLHTTCEKLVENCLESRNNATAILVQFKPGADQPIPALPNIQEGSDEVAGGADQPIPVLPNIQQVSDEVAGGTGQPIPVLPDIQEGSDEVAGGAAVAEQHQHNPEGGGEQQLDLDDALDGEALALLFGQP |
| Os02g42250 | MEKRMETLEQIKETLRETSKLVPDIVRAAVGLEHHYQTVELPHDDGCVKSFAAAFLRPQAQEQAHGDGEVQQAVRMESASCYVPDHDEDAHFVHDAAGVVGGYRRRVGVDAGAFSRGLMTSAFAQLVTAEPGTPVCPYTLLERAYEETLESGAQGGSTAVILSLADGNVLRWAYIGDSAFAVLRDGRVVVRSVQQQRYFNAPYYLGGRRGDEGMTVGMVGEMKVRRGDVVVAGTDGLFDNMSDAELEKVVQIGTALGFSPKNMADIIGGTAYEMSRCLLKDSPFAVEWRKQHENEEGHFYGGKVDDITVVVACIVSSDS |
| Os02g42270 | MEALPQIRQTLSEIDRRIPDALRVAMGLRLRPTAGAALEEVTRIAASCLPRPCPEGGDDPMECDEAAPARALRMEAASCFLPDHDEDTHFVRPEAGVVALADGVGGYRAPGVDAAAFARALMYNAFEMVVATTPGGAGGICPYALLGWAYEQAVSARTQGASTAVILSLAGATLKYAYIGDSAFAVFRDGKLFFRSEAQVHSFNYPFQLSVKNGNSVTSAARGGVEVKEGDVVVAGTDGLFDNVTSEELQRIVAMGRALGLSPKQTADVVAGFAYEASTTMGRDTPFSLESRKKQGTIFRRGKRDDITVVVAYIV |
| Os02g46080 | MFSWLLRIASACLGPARRYARTRKDEDGGDNGGGVADGLLWSRDLGRHAAGEFSFAVVQANEALEDHSQVETGSAATFVGVYDGHGGADAARFISDHLFAHLIRLARESETVSEEVVRGAFSATEEGFLTLVRRTQFLKPMIAAVGSCCLVGIIWRGVLYVANLGDSRAVVGYLGRTNKITAEQITRDHNACKEEVRQELISRHPDDSQIVVLKHGVWRIKGIIQVSRTIGDAYLKRREFALDPSITRFRLSEPLRRPVLTAEPSICTRVLSLQDQFVIFASDGLWEHLTNQQAVDIVYKNPRAGIAKRLVNTALKEAARKREMRFVDLKKVEKGVRRFFHDDITVVVVYIDHELLQEKNVSVPELSVRGFVDSVGPSRISGFDAIS |
| Os02g46490 | MGSGASRLLTACTCSRPAPASVDAEPCLDDALGHSFCYAAAATATAHSSSFRHGISGAALSANSSVPVPLYNASAAAGGVAPGYSSAFHTSSSFSSAPLQLSNLSSGPLFLSGPIDRAGQLSGPLDPAVPFSGPLPAKPPKPASSSSRGFSRRFRKPSFGSLRRSVSEKNRPCAVPLRRDDGVQWAHGRAGEDRVHVVVSEDQRWLFVGIYDGFNGPEAPDFLVTNLYRFLLRELRGIFYKEADADNKKLWQFLVDGDDDDSELDFSGSGRFALSLDRLKESRFHMWAHAAADESGREWGSRRLAPAPAVRDHAAVLAALTRALASTEAAYLDMTDQSMGTHPELAVTGACLLVALVRDDNVYVMNLGDSRAIVAQRPDDGDDGCVFGTMRRMEDVGVGLEIETRPGGCAIIGLKPLQLSTDHSTSIEEEVHRIKREHPDDDQCIVNDRVKGRLKVTRAFGAGYLKQAKLNNGLLEMFRNDYIGDTPYISCTPSLCHHKLTARDQFLVLSSDGLYQYLSNEEVVLHVENFMERFPEGDPAQSLIEELLSRAAKKAGMDFYELLDIPQGDRRKYHDDVTVMVISLEGRIWKSSGTYV |
| Os02g55560 | MCVEELEGAERLDFGGVAELETTPADFEMEKVCENTVSLDFKQARSSSFVPVIRSGDWSDIGGRDYMEDAHVCISDLANNFGHNSVDDEIISFYGVFDGHGGKDAAHYVRDNLPRVIVEDADFPLELEKVVRRSFVQTDSQFAERCSHQNALSSGTTALTAMIFGRSLLVANAGDCRAVLSRRGTAIEMSKDHRTCCLNERKRIESLGGYVDDGYLNGQLAVTRALGDWHLEGLKEVGEPGGPLSAEPELKMITLTKEDEFLIIGSDGIWDFFSNQNAVDFTRKRLQEHNDLRLCCKQIVEEAIRRGASDNLTAVMVSFHQEAPPQLRVNRTGRVERSISAEGLHSLRVLLEGQ |
| Os03g04430 | MLAAVMDYFRSCWGPRSPAGHRVRGSDVAGRQDGLLWYKDAGQLVTGEFSMAVVQANNLLEDQSQVESGALSMAEPGPQGTFIGVYDGHGGPETARFINDHMFHHLRRFATEHKCMSTDVIRKAFQATEEGFLSLVSKQWSLKPQIAAVGSCCLVGVICSGTLYVANLGDSRAVLGRFVKSTGEVVATQLSSEHNACYEEVRQELQASHPDDPQIVVLKHNVWRVKGLIQISRSIGDVYLKRPEYNREPLHSKFRLRETFKRPILSSEPAIAVHQIQPNDHFVIFASDGLWEHLSNQEAVDLVQNNPRNGIARRLVKVAMQEAAKKREMRYSDLKKIDRGVRRHFHDDITVIVVFLDSNAISKANWSRGPSVSLRGGGVTLPANSLAPFSTPTVLSSTY |
| Os03g09220 | MKRRAPPDAPQRRPARRLLPVALPPSSASAPLQVWVRGIHACLISFGFGSRFDLVLVWFGVGLDAEGIEQVPGCPCFEDAGAVVVSGEAPEGPGVLCSEDGAELKLAEQGALDVRLGSPAVGIHEQQLLHRGTSGSDEAGAINEISPVEVSPSEASSNLDTAGAIGGSPLMLESLPETSDTRGCEQEVMPGVVVGSSNRDASSEVGVESERGSDADGRNGLGEGELVSSVDGGGAEKSSKVTGVLSEEGVDGMETALEPCVASVGSITQVEEGVDRMETSLDDSEASDGSTTQDFDTDVETESSGSSIEEQDMGYGVHIPHTEQAICEVARGNKSSEVKSSDRMSSVTLPTLILASGAAMLPHPSKVLTGGEDAYFIACDGWFGVADGVGQWSFEGINAGLYARELMDGCKKAVMESQGAPEMRTEEVLAKAADEARSPGSSTVLVAHFDGQVLHACNIGDSGFLVIRNGEIYQKSKPMTYGFNFPLQIEKGDDPFKLVQKYTIDLQEGDAIVTATDGLFDNVYEEEIAAVISKSLEAGLKPSEIAEFLVARAKEVGRSATCRSPFSDAALAVGYLGYSGGKLDDVTVVVSVVRKSEV |
| Os03g10950 | MGALRRWLPCCCCCCRGGGGGGGGGSVGDGLVWDVALKAHASGDYSVAVAQANEALEDQAQVFVSPAATLVGVYDGHGGPEAARFVNKRLFSLIQEFAAQSGGISAEVLEKAFGETEEEFVASVQRSWPSQPRILSVGSCCLVGAIEDGTLYVANLGDSRAVLGRRSAAGAAHGRKGKNRVVPERLSRDHNVADEDVRRELKELHPDDSHIVLNTHGVWRIKGIIQVSRSIGDVYLKKPEICKSNPMLQQTICPFPLRRPVMSAVPTIKTRKLRPGDQFVIFASDGLWEQLTDEAAVAIVAGSPRRGVAMRLVRAAQLEAARKKDVKYERIRTIEKGQRRHFHDDITVVVLFLDKCRGKAGRGDEIDGTDGPVDVFSLSPDDREDPTRPVLR |
| Os03g16170 | MAEICCEVVAGSSSEGKGPECDTGSRAARRRRMEIRRLRVVAERGAEEETSGKRRRLDGGGGEASTDEEDREVERARYGFTSVCGRRRDMEDSVSACPGFLPGHHFFGVFDGHGCSHVATSCGQRMHEIVVDEAGAAAGSAGLDEEARWRGVMERSFARMDAEAVASSRGSVAPAPTCRCEMQLPKCDHVGSTAVVAVLGPRHVVVANCGDSRAVLCRGGAAIPLSCDHKPDRPDELERIHAAGGRVIFWDGARVFGMLAMSRAIGDSYLKPYVICDPEVRVMERKDGEDEFLILASDGLWDVVSNEVACNVVRACLRSSGRRERNRSSPTSNLSPRQSSSSGDEAPNDGAPSAAAGSESDEESAAEEDKACAEAAVLLTKLALARQTSDNVSVVVVNLRRRKL |
| Os03g16760 | MGNGITKNPCFSGDPYAAAVASDPLPDDSHGHSFTYVPSSAAAFDHSPRSAAASSETSYFSLSGAAISANPATSASMPSFRLYNELTWPPSTACTFESSRSFAAAPLIQAAPPRLSMSGPLHATSGRFSEASGSASTASDRFSDHPFMDGMLDRASSASSTARLMPSFSHLMSEPRVAQSGLSNERSLIRSLVRVASKLRFGVPLSGRRSNGPAEPTTKSDGDYRSTPKGNVEWAQGMAGEDRFHVAVSEEHGWVFVGIYDGFNGPDATDYLFANLYVAVHRELKGVLWDDIQGVDVVTDNLPDPALANATHLCFLDAGGVGGGGDDDPDAERKAKRGRIERNADDDGASSVHRDVLKALARALARTEEAFFAAAEERAAQSPELGLVGSCVLVMLMKGKDVYLMNVGDSRAVLARRREPDFKDIFFRPDQDLQLLKAEVMRELEAHDRNGLQCVQLTPEHSAAAEEEVRRIRSQHLTDRQAVVNGRVKGKLSVTRAFGAGYLKQPKWNDRLLEAFKVDYIGAEPYISCTPSLRHHRISSNDRFLVLSSDGLYQYFTNKEVVDQVAMFTAEQPDGDPAKHLVGELVLRAARKAGMDCRRLLEIPHGDRRNYHDDVSIIVMSFEGRIWRSSV |
| Os03g18150 | MSCTVAIPSSPVFSPSRRPLSCKAASASASPESVSVAASSPAQAAPPAGSPLRPFALRAHLREEATPSPQPSAAAAAAVSAPAGSVLKRRRPAPLVVPVCGGAAAAAAAAAVAAVESDPRNEVEEDGEEFAVYCRRGKGRRRVEMEDRHVAKVALGGDPKVAFFGVFDGHGGKSAAEFVAENMPKFMAEEMCKVDGGDSGETEQAVKRCYLKTDEEFLKREESGGACCVTALLQKGGLVVSNAGDCRAVLSRAGKAEALTSDHRASREDERERIENLGGFVVNYRGTWRVQGSLAVSRGIGDAHLKQWVVSDPDTTTLGVDSQCEFLILASDGLWDKVENQEAVDIARPLYISNDKASRMTACRRLVETAVTRGSTDDISIVIIQLQQFSR |
| Os03g18970 | MESAAGEEGKAAPSLPLATLIGRELRGGGSERPLVRYGHFGFAKRGEDYFLVKPDCLRVPGDPSSAFSVFAVFDGHNGVSAAVFSKEHLLEHVMSAVPQGIGRDDWLQALPRALVAGFVKTDIDFQRKGEASGTTATLVVVDGFTVTVASVGDSRCILDTQGGVISLLTVDHRLEENVEERERVTASGGEVSRLNLCGGQEVGPLRCWPGGLCLSRSIGDTDVGEFIVPIPHVKQVKLSNAGGRLIIASDGIWDALSSEAAAQACRGLPAELAAKLVVKQALKTSGLKDDTTCVVVDIIPSDHSSTPPSLSPKKNQNKLRSLLFGRRSHSSVGKLGNKSASFDSVEELFEEGSAMLDERLGRNFPSKANSSPSRCAICQVDQAPFEDLVTDNGGGCCSAPSTPWVGPYLCSDCRKKKDAMEGKRSSRSTACR |
| Os03g25600 | MVLGLGVANQPAMGNSTSRVVGCFAPADKAAGGGVGLEFLQPLDEGLGHSFCYVRPGAITDSPAITPSNSERYTLDSSVLDSETRSGSFRQEVVVVDDLAAAAMAGLQRPSKSFSETTFRTISGASVSANPSSARTGNLCVSLAADVQEPAAAFESTASFAAVPLQPVPRGSGPLNTFLSGPLERGFASGPLDKGAGFMSGPLDKGVFMSGPIDSGNKSNFSAPLSYGRRKAGLGQLVRSISRPMRSALSRTFSRSSQGTGWVQRFLLHPMAQLSLSRDAKGTSEDSHNGLEAGLPELEYSVTRNLQWAHGKAGEDRVHVVLSEEQGWLFIGIYDGFSGPDAPDFLMSNLYKAIDKELEGLLWVYEDSPEGSAQVSTLGEGESVAVPQDLPDGGDILFQADSVESEQLVNSEEQDVSNVKISDGGASQVQMDLNTSGQRDLVLQASSNQKLNAGEIVEEKVGADMGNNLQSTESYNSGRDISNTDVNTSFGCTSDVNTSTCCNEDVKSPKEIRSRRLFELLEMELLEEYNRNVSKLSPEGMKGRSIFNMQAGTTEESSRDIAELSRSSMAATGECLDDFENDKHSRSGDGVLGVDPKECNECSISSSSSGHKQILRRYLFGSKLRKMYKKQKLLQKKFFPWNYDWHRDQPHVDESVIKPSEVTRRCKSGPVDHDAVLRAMSRALENTEEAYMDVVERELDKNPELALMGSCVLVMLMKDQDVYVMNLGDSRVVLAQDNEQYNNSSFLKGDLRHRNRSRESLVRVELDRISEESPMHNPNSHLSSNTKTKELTICKLKMRAVQLSTDHSTSVEEEVSRIRAEHPDDPQSVFNDRVKGQLKVTRAFGAGFLKKPKFNDILLEMFRIDYVGTSSYISCNPAVLHHRLCSNDRFLVLSSDGLYQYFSNDEVVSHVAWFMENVPEGDPAQYLVAELLCRAAKKNGMDFHELLDIPQGDRRKYHDDVSVMVISLEGRIWRSSG |
| Os03g27780 | MEVQGEVDGSGVPLAVLLKRELCNQKVERPDMLFGEASKSKKGEDFTFLLPKCSRRPGQAQADGEDAGGAGDDDTISVFAIFDGHNGSAAAIYTRENLLNNVLAAIPPNLTSEEWTTALPRALVAGFVKTDKEFQTKAARSGTTVTFVIIDGWVVTVASVGDSRCILESAEGSVYFLSADHRLDTNEEEVERVTASGGDVGRINIAGGAGIGPLRCWPGGLCLSRSIGDIDVGEFIVPVPHVKQVKLSNAGGRLVIASDGVWDALRFQEALNYTRGLPAEAAASRIVKESVSSKGLRDDTTCIVVDILPPEKLSPPLKKHGKGGIKALFRRRPSDELTEDQMDRGCLEPDVVEEIYEEGSAMLAQRLKINYPTGNMFKLHDCAVCQLVMKPGEGISVHGSIPRNSRVDPWGGPFLCSSCQLKKEAMEGKQHLTNSQPTVQPVLK |
| Os03g55320 | MLRAVARCCGHWPPGAAAADGMLWQTELRPHAAGEFSMAAAQANLAMEDQAQVLASPAATLVGVYDGHGGADASRFLRSRLFPHVQRFEKEQGGMSTEVIRRAFGAAEEEFLQQVRQAWRQRPKMAAVGSCCLLGAISGDTLYVANLGDSRAVLGRRVVGGGVAVAERLTDEHNAASEEVRRELTALNPDDAQIVVHARGAWRVKGIIQVSRTIGDVYLKKQEYSMDPVFRNVGPPIPLKRPALSAEPSIQVRKLKPNDLFLIFASDGLWEHLSDDAAVQIVFKNPRTGIANRLVKAALKEATRKREVSFRDLKTIEKGVRRHFHDDISVIVVYLDRHRGRRHTRVVDSSSNCTNAPVDIYSSNSGQSVETLQAHRGSGW |
| Os03g59470 | MLAGGGSRVSGGGGGFNLASYKYKDPLLGRGGRSFLFGNTWFLLSTYPARLLHTADRRAPAAFFAAINRTPCVRTHCTGQSLLQRGIVMAACGYAFRRAELGAAKRQPEKDSSVGTRISCVVAMGSAGSTPRPEVSFRHRGVEYCKKVGVSLKCREPWGPSRAFWTNAIGPSYKLSFSVEPWLRDFSTSCVAPYSAGATEHQLSLDEAVQDKQMDNSTVGPDGKPRAPGPLKLVSGSCYLPHPAKEATGGEDGHFICVDEQAIGVADGVGGWADHGVDAGLYAKELMSNSMSAIKDEPQGTIDPSRVLEKAYTCTKARGSSTACIVALKEQGIHAVNLGDSGFIIVRDGRTVLRSPVQQHDFNFTYQLESGGGSDLPSSAQTFHFPVAPGDVIIAGTDGLFDNLYSNEISAIVVEALRTGLEPEATAKKIAALAQQKAMDRNRQSPFAAAAQEAGYRYFGGKLDDITVIVSYVTSASAT |
| Os03g59530 | MAIPPLAVSGVAVATLAVLGLAVFACRRWRRGASPAPPPPASSQDDDINMPLISDNLDDYSVSSNSSTVDESGIRIDRIITSPKTHGIVGKGATYPTESHVIEGETHVIDVTNSKTEELYLGNTLKRPAVANGPTPDVKHIRRDSGESNHNGTIPDIIVGSNLALEVIAGPSHGINHYMQSGNKSMLPVTLGRVPPSHLVLKDSEVSGKHAQIDWNANKLKWEIVDMGSLNGTFLNSRSVNHPDVGSRRWGEPAELADGDIITLGSSSKVSVQIELQNQQPVGVGIASDPMTARRTGKKLHMEDVSCCQYPLIGVEKFGLFGIFDGHGGDGAAIAASRILPQNIANILSQQETKERVLSCHSASDVLRHAFALTEAALHHQYEGCTATILLIWFDQNEDCFAQCANLGDSACIMSVNGEIITMTEDHRVVSTTERARMANSGQPLKDGESRICGLNLGRMLGDKFLKEQDSRFSSEPYVSQAVRMTKACLASALIASDGLWDVISANRAAQLVLEGKQKYSEQKTSADKVAHHVLSEARKLRTKDNTSVIFVDLDTLRSDP |
| Os03g60650 | MGNSLACFCCGGGAGGRGGRHVAPAALPSDPAYDEGLGHSFCYVRPDKFVVPFSADDLVADAKAAAAAEGEATTFRAISGAALSANVSTPLSTSVLLLMPEESSASATASSGFESSESFAAVPLQPVPRFSSGPISAPFSGGFMSGPLERGFQSGPLDAALLSGPLPGTATSGRMGGAVPALRRSLSHGGRRLRNFTRALLARTEKFQDSADLGSPDAAAAAVAACGGDPCGLQWAQGKAGEDRVHVVVSEERGWVFVGIYDGFNGPDATDFLVSNLYAAVHRELRGLLWDQREQNVQHDQRPDQPGSAPSTTASDNQDQWGRRRRTRRSRPPRGADDDQRRWKCEWEQERDCSNLKPPTQQRLRCNSENDHVAVLKALTRALHRTEEAYLDIADKMVGEFPELALMGSCVLAMLMKGEDMYIMNVGDSRAVLATMDSVDLEQISQGSFDGSVGDCPPCLSAVQLTSDHSTSVEEEVIRIRNEHPDDPSAISKDRVKGSLKVTRAFGAGFLKQPKWNDALLEMFRIDYVGSSPYISCNPSLFHHKLSTRDRFLILSSDGLYQYFTNEEAVAQVEMFIATTPEGDPAQHLVEEVLFRAANKAGMDFHELIEIPHGDRRRYHDDVSVIVISLEGRIWRSCV |
| Os03g61690 | MLGALLRLLSACGGVWPTSPAPPARSSSSSSAAAAADQAAAEGRDGLLWWRDLARCHAGELSVAVVQGNHVLEDQCRVESGPPPLAATCIGVFDGHAGPDAARFACDHLLPNLREAASGPEGVTADAIRDAFLATEEGFLAVVSRMWEAQPDMATVGTCCLVGVVHQRTLFVANLGDSRAVLGKKVGRAGQITAEQLSSEHNANEEDVRQELMAQHPDDPQIVALKHGVWRVKGIIQVSRSLGDAYLKHSQYNTEQIKPKFRLPEPFSRPILSANPSIIARCLQPSDCFIIFASDGLWEHLSNQQAVEIVHNHQRAGSARRLIKAALHEAARKREMRYSDLMKIDKKVRRHFHDDITVIVLFINYDQLAKGHSQGQSLSIRCALDH |
| Os04g08560 | mvmasagvnmpggdgdhppaaaqechrlrrrryvpaaaaasedgdnssngggekrslpassaspspsptssaassdcssdrdddgcsstagaaarrlplpsgastaaavwpvafgsvslagrmrdmedavslrpsfctwldgspmhffavfdghggphvsalcreqmhvivaeemvaeaaalrqrqpaameeeeeeravaggavaelrpggraggggvrvrarhragvpcplsgqtgaiigstavvallvrdrlvvsncgdsravlcragdplplssdhkglnpslswrgtrvalargtwgdktgqsvgpaalllsggahpdrpdekarieavggrvvylngprvrgilamsralgdkylkpevicepdititvrtvddeclilasdgmwdvisnetasdvarqcledgsptsgrraarsgeaasssagapaaavgqeseprcyraaallarlalgressdnisvvvidlkgrg |
| Os04g25570 | mvavtggrppglqdapgapppapaaeavpsrplardatyggrvyggvggggcclefldcvlramgvatpaeimppadfrwaarpmrrrrrggsssssssprdreprdgriaangasaaaslytmrgnkgvnqdamlvwenfcskedtifcgvfdghgpyghlvskrvrdllpiklsanlgrdghketstnivtssmtegggtermdrdtetplgteengdypemfaalrtsllrafyvmdrdlkfhktidsvfsgttavtvikqghdllignlgdsravlgtrdeydqffavqltvdlkptipseaarirersgrifslpdepdvarvwlpkynmpglamarafgdfclkdyglismpdvsyhritekdefvvlatdgvwdvlsnsevvsivsqakseasaarfvvesaqrawrtrfptskiddcavvclflntdarnkppgsgikdlanaielgggnls |
| Os04g33080 | MVDEELFDKSSNDHSISSEEEDMLVRSYSNLNVSFGYHCNSYQCFSLDTDEYDISPNKRLETNTMMTSQNGSFTCLSGAAISANFTLANTNICKGLIGEEILPELDSPNSFRKIVSSPSMSRLDLLSTSQGSPVSTESSIFEISKNIWRSSAPTTVSSNFLTSTEIKMAGGAAGEDRVQAVCSEKNGWLICGIYDGFNGRDAADFLAVTLYDNIVYYLYLLECRIKQENGLYGSPEGSLNGVKSELTLAMRFAENEDVKFSETFRAGVLKCLTTAVEQAENDFLCMVEQEMDDRPDLVSVGSCVLVVLLHGTDLCILNLGDSRAVLASVPSSGMDKLKAVQLTEIHSLENPLEYQKLLADHPNEPSVVMGNKIKGKLKVTRAFGVGYLKQKKLNDALMGILRVRNLCSPPYVYTNPHTVSHKVTEDDLFVVLGSDGLFDFFSNDEVVQLVYQFMHDNPIGDPAKYLIEQLLLKAAKEAALTAEELMRIPVGSRRKYHDDVTIIVIILGNAQRTMTASTSL |
| Os04g37660 | MAAAAAAATVEAVGVAGGRRRRSGSVALGDLLRREASAERASASASAGAGGRERERRPSVAAGQACRAKKGEDFALLKPACERLPAGGAPFSAFALFDGHNGSGAAVYAKENILSNVMCCVPADLSGDEWLAALPRALVAGFVKTDKDFQTRAHSSGTTVTFVIIDGYVVTVASVGDSRCVLEAEGTIYHLSADHRFDASEEEVGRVTECGGEVGRLNVVGGAEIGPLRCWPGGLCLSRSIGDQDVGEFIIPVPYVKQIKLSSAGGRIIISSDGVWDALTVDTAFSCARGLPPEAAADQIVKEAIASKGLRDDTTCIVIDIIPPEKISPTVQPAKKAGKGLFKNIFYKKATSDSPCHADKDQCTQPDLVEEVFEDGCPSLSRRLDSEYPVRNMFKLFICAICQVELESGQGISIHEGLSKSGKLRPWDGPFLCHSCQEKKEAMEGKRHSRDSSSRNSGSSE |
| Os04g37904 | MSRFCCFGAGCSEFSGHASTSSGKGKGIQGQVKVSYGFYLVRGMTNHPMEDYHVAELAEEKGNELGLFAIFDGHLGDTVPAYLQKNLFANILNEEEFLTQPDRAIIKAYEKTDQAILSHTPDLGQGGSTAVTAILLNGRKLWVANVGDSRAVLLKGGRPIQMSTDHDPNVERSAIENRGGFVSNMPGDVPRVCGQLAVSRAFGDRNLKSLLKSEPDIKVEDIDYTAELLVLASDGLWKVMNNQEVVDVAKRFKDPQAAAKQLTAEALKRDSKDDISCVVVRFRM |
| Os04g42260 | MAQPQRPLQVPDITKSTHSGGNTVLAYASSAMQGYRSTMEDAHATIENLDALTNTSFFGVYDGHGGSAVARYCANHLHNKVLEQEDFSSNLANALRQSFFRMDEMLRNQAASKELTEYGSGNEYWRTAGRSWLRCAPCVLGPVYCGPLAEGCTACVVLIRNTQIVVGNAGDARCVISRNGQAIALSNDHKPNFPEETQRIVAAGGSVSFSRGSHRVNNGIAVSRAIGDLSYKNNKKLRPEQQLLTCSPEIRADQLTDDTEFLVIACDGVWDVLANQAVVDFVRLHLNNGVELSVICESLLQEAITRDPPSTDNMSVILVRFLHPEGNRGARAATSSTSTGTVPSRHSKSISL |
| Os04g49490 | MWPWLERIASACWDRVRRYALTRRDEEDGSGSGGDADDLLLWSRDLVRHAAGEFSFAVVQANDVLEDHSQVETGAAATFIGVYDGHGGAEASRFISNHLAAHLVRLAQERGTISEDIVRNAFSATEEGFLSLVRRTHLIKPSIASIGSCCLVGIIWKGTLYLANLGDSRAVVGCLTGSNKIVAEQLTRDHNASMEEVRQELRSLHPDDSQIVVLKNGVWRIKGIIQVSRSIGDAYLKKQEFALDPSMTRFHLSEPLRRPVLTSEPSIYTRVLHSQDSFFIFASDGLWEHLTNQQAVEIVHNNPREGIARRLVKAALKEAARKREMKYNDIKKLEKGVRRFFHDDITVVVVFIDHELLQDGDESTPEISVRGFVDSGGPSSFSGLNGIS |
| Os04g52000 | MVGRMERQSASSSASCSPSSSAAGTSSSSSACGGKKRPDILNMIRSATCLNSSSTDTGKGRSKQSSNKVTHGFHLVEGKSGHDMEDYHVAEYKYDKSHELGLFAIFDGHLGDSVPSYLKANLFCNILKEPIFWTNPQEAIKNAYRSTNKYILENAKQLGPGGSTAVTAIVVDGKDMWVANVGDSRAVVCERGAANQLTVDHEPHTTNERQRIEKQGGFVTTFPGDVPRVNGQLAVARAFGDQSLKAHLSSEPDVRHVPINSSIEFVILASDGLWKVMKNQEAVDLVKSIKDPQAAAKRLTTEALARKSKDDISCIVIRFRC |
| Os04g56450 | MGYLSSVIPTDGSPVSGGGLSQNGKFSYGYASSPGKRASMEDFYETRIDSVDGQIIGLFGVFDGHGGAKVAEYVKQNLFSHLLRHPKFISDTKVAIDDAYKSTDSEFLESDSSQNQCGSTASTAVLVGDRLFVANVGDSRAIICRGGNAIAVSKDHKPDQTDERQRIEDAGGFVMWAGTWRVGGVLAVSRAFGDKLLKQYVVVDPEIREEVIDHSLEFLILASDGLWDVVTNEEAVDMTRSIHDPEEAAKKLLQEAYKRESSDNITCVVVRFLHGQGSSGYA |
| Os05g02110 | MGNSLASLATPCFADAAAGGGRGRGHHAAGDDAVAFDDDDAAGGCNSIGHILSFDGRDAPAFAIHGVLLPSNPSTMASTGGGGGGGASVLNDGALSIGSSSFDSSNSFSFRTLQPRQYSGPLEYCTTSPSTSGASSSRQLGPRTDKQILNDIYANRQRRRCQGSKGPPLLGRLRKAVASLLRAGPCGFPEQEEPAAMINGVGVVRNGEESISRNVDAAAADDGAERVQWARGKAGEDRVHVVVSEEHGWMFVGIYDGFNGPDATDYLADNLYAAVCRELNGVLSEDEPDPPEAAAAAGRCNGCGGAARHREVLDAMARALRRTEEGYFAEAEARAAECPELAMMGSCVLVVLMKGADVYAMNVGDSRAVLAHQAEPDLSHVVLPRGSHHDGDGDLAGVKEAIKRQFDECEMGELAALQLTMDHSTNVYKEVRRIRSEHLDDPGCITNGRVKGCLKVTRAFGAGYLKEPRWNKALLEVFQVDYVGSSPYISCRPYIRHHRLGAQDKFLILSSDGLYDYFTKEEVVAQVEAFTAGYPDEDPAKYLSHQILLRAANQAGMGFHELLEIQQGDRRQYHDDVSIIIISLEGKIWRSSQ |
| Os05g04360 | MVAEAEVMHQPVPVLEVPYHRCVAKGVEEVAAAAAVAPPPVVEVEVAVQVPHMGLESAAGAPSISVDALQFVPSIRSGSFADIGPRRYMEDEHIRIDDLSAHLGSLLVCPLPSAFYGVFDGHGGLDAAAYMKRHAMRFLFEDSEFPQASQVDETYVQSVENSVRRAFLQADLALADDLDISRSSGTTALTALVFGRQLLVANAGDCRAVLCRRGVAMEMSRDHRANYAEECERVAASGGYIEDGYLNGVLSVTRALGDWDMKMPDGSISPLIAEPEFRQTMLTEDDEFLIMGCDGIWDVMTSQHAVSIVRRGLRQHDDPERCARELVMEAKRLETADNLTVIVVCFVSELGSPRQEQVGGQAGVARPRSCKSLSAEALCNLRSWLETDR |
| Os05g29030 | MRHISSLLQGLARSLSVGKERKGGDGDDGKAAAATATAVLRTSGTLWGEGSETFAAVCSRRGEKGINQDCSIVCEGFGCEEGSVLCGIFDGHGQWGHYVAKAVRESLPPALLRRWREAVTLAALIDGGEKRLCECRPDLWRQSYLAACAAVDAELRASRRLDAVHSGCTALSLVKHGDLLVVANVGDSRAVLATASPDDGGGARLAAVQLTVDFKPNLPQERERIMECNGRVQCLADEPGVHRVWRPDREGPGLAMSRAFGDYCVKDYGVISAPEVTHRRITAQDHFVILATDGVWDVVSNEEAVQIVASAPEREKAAKRLVEFAVRAWRRKRRGIAVDDCSAICLFFHSPPS |
| Os05g38290 | MAAEICREEAAKSMPAAAAGATAIARRRRRVEGFRFAAGSLEPPQEDADAGVARCGKRQRVAGARAGAGAATAGPCRPSAGAEFGSRWWPRYGVTSVFGRRREMEDAVSIRPDFLRGSTSSGKHHFFGVFDGHGCSHVARMCQDRMHELVVDAYKKAVSGKEAAAAAPAWKDVMEKGFARMDDEATIWAKSRTGGEPACRCELQTPARCDHVGSTAVVAVVGPNRVVVANSGDSRAVLCRAGVPVPLSVDHKPDRPDELERIKAAGGRVIYWDGARVLGVLAMSRAIGDGYLKPYVTSEPEVTVTERADDDECLILASDGLWDVVTNEMACEVVRACFRSNGPPSPPGCSRPKAVLPPPAGASGGGGGDAVVKGVDKAESDKACADAALLLAKLAIARRSADNVSVVVVDLRRPVP |
| Os05g46040 | MAAAAAAAAICGEDETAARVGCTGEWAGGIERVDLGERKEAVAAAGAGKRSVYLMDCAPVWGCASTRGRSAEMEDASAAVPRFADVPVRLLASRRDLDALGLDADALRLPAHLFGVFDGHGGAEVANYCRERIHVVLSEELKRLGKNLGEMGEVDMKEHWDDVFTKCFQRVDDEVSGRVTRVVNGGGEVRSEPVTAENVGSTAVVALVCSSHVVVANCGDSRIVLCRGKEPVALSIDHKPDRKDERARIEAQGGKVIQWNGYRVSGILAMSRSIGDRYLKPFVIPKPEVMVVPRAKDDDCLILASDGLWDVVSNEEACKVARRQILLWHKNNGAASPLSDEGEGSTDPAAQAAADYLMRLALKKGSEDNITVIVVDLKPRKKLKNIS |
| Os05g49730 | MRETGATDEGHACEVVVAGGDGKAAAARRRRRLELRRLGLAAEDDAAAKRIRSVKDGSSSDDSSTEVVPRSWPACVSHGSVSVIGRRREMEDAVAIERTFMASTGDGAGAIRGGGEGEEDFFAVYDGHGGSRVAEACRKRMHVVLAEEVSLRRLRGQSASGGDVRWKEAMLASFARMDGEVVGSVAAAAPRVDGTEPSGFRTVGSTAVVAVVGRRRIVVANCGDSRAVLSRGGVALPLSTDHKPDRPDELERVEAAGGRVINWNGYRVLGVLATSRSIGDYYLKPFVSAEPEVRVVERTDKDEFLILASDGLWDVVSNEVACKIARNCLNGRAASMFPESVSGSSAADAAALLAELAVSRGSRDNISVVVVELRRLKSRAA |
| Os05g50970 | MVYDGAVKDQESSANPASASAALSEASAAASEVTAAAAAGAGAGAAEEGAAVSGRPPRPPHDKRLGVRHPLKHRRFRAGGKVMVEPGDPPSAQEVADEEASEVEQEAAPVEREPPQEEGGDVEVSSAPAEMEVVEGDAMEVSPEPAVAVGESELEGRPGEEEEVSSPVVSQGERKQETAAAAPVPAVEEKKHKDQENKHKEREREKERERVDEVGYMSGGWKSEDGFLSCGYSSFRGKRASMEDFYDIKSSKIDDKQISLFGIFDGHGGSRAAEYLKEHLFENLMKHPEFMTNTKLAISETYKKTDSEFLDSESHTHRDDGSTASTAVLVGNHLYVANVGDSRAVISKAGKAIALSEDHKPNRSDERKRIESAGGVVMWAGTWRVGGVLAMSRAFGNRLLKQFVVADPEIQEQEIDDELEFLILASDGLWDVVPNEDAVSLVKIEEEPEAAARKLTETAFSRGSGDNITCIVVKFQHDKMDGDSSPTSDKS |
| Os05g51510 | MEDLALPAAPPAPTLSFTLLAAAAAVAEAMEEALGAALPPLTAPVPAPGDDSACGSPCSVASDCSSVASADFEGFAELGTSLLAGPAVLFDDLTAASVAVAEAAEPRAVGATARSVFAMDCVPLWGLESICGRRPEMEDDYAVVPRFFDLPLWMVAGDAAVDGLDRASFRLPAHFFAVYDGHGGVQVANYCRKRIHAVLTEELRRAEDDACGSDLSGLESKKLWEKAFVDCFSRVDAEVGGNAASGAPPVAPDTVGSTAVVAVVCSSHVIVANCGDSRAVLCRGKQPLPLSLDHKPNREDEYARIEALGGKVIQWNGYRVLGVLAMSRSIGDKYLKPYIIPVPEVTVVARAKDDDCLILASDGLWDVMSNEEVCDAARKRILLWHKKNAATASTSSAQISGDSSDPAAQAAADYLSKLALQKGSKDNITVVVIDLKAHRKFKSKA |
| Os06g08140 | MCVEESEGAERLDFGEPAAAAADAGKSKSKSPDELPSPRMERVCENTTAADFKQNKSGNFVPNIRSGDWSDIGGRQYMEDTHVCITDLAKNFGYQSVDNEAISFYGVFDGHGGKDAAHFVRDNLPRIIVEDADFPLELEKVVRRSFVHADNQFAKTTLSSGTTALTAMIFGRTLLIANAGDCRAVLSRCGTAIEMSVDHRPCSLSEKLRVESLGGYVDDGYLNGLLGVTRALGDWHLEGMKEAGNPGGPLSAEPELKMITLTKDDEFLIIGSDGIWDVFSNQNVVDFARRRLQEHNDVKSCCREIVEEAIKRGATDNLTAVLVSFHLEAPPQVRVSRPGRVARSISAEGLNSLRTLLRNQ |
| Os06g33530 | MRRHHLLGLLRRAAASSTSAASSRAGPHPSLHAPGPLRNGGSAPRFFSSRGGAGAASKGLGDDEVELYSLLLGVSIGDEGEASSRGPAASRGGRRGRNSKRQPPRSRFDGDGVGCSKDGKLSWGYSSFQGRRPSMEDRLSIKSTTVNGETVSLFGVFDGHGGPRAAEYLKKHLFKNLVKHPKFLKDTKLAINQTFLKTDADFLQSISSDRYRDDGSTAVAAILIGNRLYVANVGDSRAVALKAGKAVPLSEDHKPNKKDERKRIEDAGGIVVSDDIWRVDGILAVSRAFGNRLMKRYVKAEPNIQEKVVDEGLEYLVLATDGLWDVMRNEDAVSLLKAQDGPKAAAMKLTEVARSRLTLDNVTCIVLQFHHGKSTNSK |
| Os06g33549 | MARAAGLRALVGIEAAGRGRRVAASPSPGGTPAASRGLPGWPGFCGVGCGSSSSSSFAPPRMQARRAAGSAARTRSPSQSNGWITGGSASEDGRLSWDYSSFKGRRPSMEDRFSIKMTTINEQTVSLFGVFDGHGGSLAAEYLKEHLFENLVNHPELLRDTKLAISQTFLKTDADFLESVSSNPFRDDGSTAVTAILVGNHLYVGNVGDSRVVALKAGKAVPLSEDHKPNRKDEQKRIEDAGGIVVFDDTWRVNGLLAMSRAFGNRALKHYVKAEPDIQEKVVDESLEYLILATDGLWDVMRNEDAVSLLKAQDGPKAAAMKLTEVAHSRLTLDNITCIVLQFHHGKSTNSN |
| Os06g39600 | MEEHRLGGGGGGGGGGGRPPIPGAAGRKLPGLSRHASFVRSPANSTKSGTEKTFENMDAVAYMPVVRSGGWADIGSRHTMEDVFICSDNLMKEFGVESFEDGPSAFYGVFDGHGGKHAADFVCSNLARFIVEDEDFPREIEKALSSAFLQTDAAFADACSVNSSLASGTTALAALVVGRSLLVANAGDCRAVLCCRGKAIEMSRDHKPSCNREKVRIEASGGYVYDGYLNGQLNVARAIGDWHMEGMKACDGLGPLSAEPEVMIRNLTEEDEFLIIGCDGIWDVFRSQNAVDFARRKLQEHNDPVTCCKELVDEAIKRKSGDNLSVVVICFNSRPPPVLTTPRPRVQRSISAEGLRELQSFLDSLAD |
| Os06g44210 | MGVYLSTPKTEKLSEDGENDKLKFGLSSMQGWRATMEDAHSALLDIDNDTSFFGVFDGHGGRVVAKFCAKYLHREVLRSEAYSAGDLGNAAHKAFFRMDEMMRGQRGWRELQALGDKINQISGMIEGLIWSPRGSDSNDQHDDWAFEEGPHSDFAGPTCGSTACVAIVRNSQLVVANAGDSRCVISRNGQAYNLSRDHKPELEAERERILKAGGYIQMGRVNGTINLSRAIGDIEFKQNKFLSPDKQMLTANPDINTVELCDDDDFLVLACDGIWDCMSSQQLVDFIHEHINTESSLSAVCERVLDRCLAPSTLGGEGCDNMTMILVQFKKPISQNKNVSPAEQSAADKQPTGDTHWSEIHVTEESSS |
| Os06g48300 | MREVLLLGSLVVLALLSLFPCCSCLSQGAEEEEDDGEVRLMGLAGEAAGSPGSGGGFSANGKFSYGYASSPGKRSSMEDFYDTRIDGVDGETVGLFGVFDGHGGARAAEFVKQNLFTNLIKHPKLFSDTKSAIAETYTSTDSELLKAETSHNRDAGSTASTAILVGDRLLVANVGDSRAVICRGGDAIAVSRDHKPDQSDERQRIEDAGGFVMWAGTWRVGGVLAVSRAFGDKLLKQYVVADPEIKEEVVDSSLEFLILASDGLWDVVTNEEAVAMVKPILDSEQAAKKLLQEASQRGSADNITCLVVRFLEQENHLPERPTNDQAS |
| Os06g50380 | MIVTLMNLLRACWRPSSNQHARAGSDVAGRQDGLLWYKDTGQHVNGEFSMAVVQANNLLEDQCQIESGPLSFLDSGPYGTFVGVYDGHGGPETACYINDHLFHHLKRFASEQNSISADVLKKAYEATEDGFFSVVTKQWPVKPQIAAVGSCCLVGVICGGILYVANVGDSRVVLGRHVKATGEVLAVQLSAEHNVSIESVRKELQSMHPEDRHIVVLKHNVWRVKGLIQVCRSIGDAYLKRSEFNREPLYAKFRLREPFHKPILSSEPSISVQPLQPHDQFLIFASDGLWEHLTNQEAVDIVHSSPRNGSARRLIKAALQEAAKKREMRYSDLKKIDRGVRRHFHDDITVIVVFLDSSLVSRASTYRGPSVSLRGGGVNLRSNTLAPYASQM |
| Os07g02330 | MMARWCVGWPAAARGGRDELTWQAELTAHAAGEFSMAAAQANAVMEDQAQVMASPGATLVGVYDGHGGPDASRFLRSRLFPLIHEFAAERGGAVDADVIRKAFLAADEEYLQLLRWSLPNMSRAAASGSCCLLGAISGDTLYVANAGDSRAVLGRRAAAGQTVAERLSTEHNVASEEVRRELAALHPDDGEVVVHARGAWRVKGIIQVARAIGDVYLKTPEFKRDPAVQRLCSAAAAVELARPVVTAEPSIHARKLKAGVDLFVVFASDGLWEHLSDEAAVQLVSKSSTRRGVAARLVQAALGEAARKREVRRGDLRRIERGVRRHFHDDITAVVVFLDLDDDGGRRARRRGRVVDSSSSSCSNTPLDVYSLYNSTA |
| Os07g11010 | MAAPPLPLVLAAAAIASLVILVLVVFACRRWRRAVVAAAPQPPPRAAADVVAASPVRSQNEDLNKPLLEILDDHSSQSNTFPGNVVGESSKVQTSRSDTSPRSHGISDSGRTYPADSCTPQGETHVIDVTDDTSEEFHLGSTLKCTKQTSWSRPDKKHKRWGSGEDNKNGSISLKDNTYRSNLDVEVIAGPSHGISCSRQSTSPTIPITLGRVPPSDLVLKDSEVSGKHARINWNAKTLKWEIVDMGSLNGTFVNSRAVHHPNVGSRHWGEPAELADGDIITLGTSSKLSVQISLQNQRVPAGIGMASDPMVGRRSGKKLAMEDISFCQCPLQGVEQFGLFGIFDGHGGDGAARAVSKIFPENVATLLSHHETKEKVLSYSDASDVLRYAFTMTEAAIDHEYEGCTATVLLIWFDQKKDCFAQCANLGDSACVMSVNGKMIEMTEDHRVASVTERARIARAGQALKAGEVRINGLNLARMFGDKFLKEQDSRFSSEPYVSQAVHITKACTAFAVIASDGLWDVISTKRAVQLVVEGRERNSGDSASADKVANRILSEARNLRTKDNTSVIFVDFDILRTDHCIAK |
| Os07g32380 | MAGKEIYHKMKDKVKDAFSSSGPETGKGKTKLSGKRVKHGYHLVKGKSNHPMEDYLVAEYRQEGEHDLGLFAIFDGHLGHTVPDFLRSHLFDNILKQPEFLSNPQAAIRNAYQLTDAKILESAAELGRGGSTAVTAILISSENSVNLVVANVGDSRAVISKSGVAKQLSVDHEPNKERHSIEKKGGFVSNLPGDVPRVDGQLAVARAFGDRSLKKHLSSEPDVVEEPIDENTDFLILASDGLWKVMSNQEAVDEIKDFKDAQAAAKHLTEQAVNRKSKDDISCIVVKFLC |
| Os07g33230 | MASSQQAVRETGRGRASSSSAGGRKVTFGYHLVEGKTPHGMEDLHVAEFRRLDDGNEDGGDGGSTAVTAILINGETLAVANVGDSRAVAFDVRAGRAQQLSVDHEPLRERDAIEHCGGFVTEIHGDVPRVDAQLATSRAFGDRQIKEHISSDPNVTIEDVGGRRRRWWHGARRPRQRRGVEECFLQRLAEYAICIHGASMEHRNFHVHSSHVHYFEIPPFKEV |
| Os07g37890 | MGNCVARSGTAVDAGGDGGEDGKRRRRRWKAPREDQLGMVPGRIFSNDGRSRTATVYTQQGRKGINQDAMLVWDGFGGEDDGVLCGVFDGHGPHGHVVARRVRDSLPLRLMSAARDSGADMPAAAWRKAFARAYKAMDKDLRSHPSLDCFCSGSTAVTVLKLGSDLYMANIGDSRAVLGSREATGGGMVAVQLTVDLKPDVPSEAERIKKCRGRVFALQDEPEVPRVWLPFDDAPGLAMARAFGDFCLKDYGVISVPEFFHWSLTEKDQFVILASDGVWDVLSNQEAVDIVSASPSRSKAAKSLVEAATREWKTKYPTSKIDDCAVVCLYLDGKMDHERDSTASLDNISIEEGSVADPNEPQEQEPTLTRNFTVRTVAGSTQEKTLAGVDARIAGVANDQNWSGLDGVTRVNSLVQLPRFSEERAIG |
| Os07g45170 | MGCAQGKCCVPRRQRGRGGGGAVGGRGGATLGRVAVPGAGLVLEYATLAVAGLYPDSPGRESQDAHLVATRFAGHPDLHLFAVFDGHGACGAACAGFARDALPRLLAGVGVGAGEEGGGRMVVVEDPAAAFREALPAANAEMHAADEVDDSMSGTTAVAALVAGGALHVANVGDSRAVAGVWREGRVAAEELSWDQTPFRADERARVKACGARVMSVEQVEGVRDPEAESWVADEGDPPRVWARDGLYPGTAFTRSLGDQAAEAVGVIAEPEVKSVEITPAHLFFVVASDGVFEFLSSQDVVDMVAAYEDPREACSAIAAESYKLWLEHENRTDDITIIIVHIRDSENVDKFLYRKRIPSAGSCCCAMAVVLALQSVRHAPVANPLGAVARVFLRAPAD |
| Os07g49040 | MAGIAKERRLPPALPLATLIGRELRAGGSERPSLRYGHAGFAKRGEDYFLVKPDCLRVPGDTSTAFSVFAVFDGHNGVSAAVYSKEHLLEHVMSALPPDIGRDDWLQALPRALVAGFVKADIDFQRKGEVSGTTATLVVVDGFTVTVASVGDSRCILDTQGGEVQLLTVDHRLEENAEERERVTASGGEVGRLNLFGGQEVGPLRCWPGGLCLSRSIGDMDVGEFIVPIPHVKQVKLSNIGGRLIIASDGIWDALPSEAAAKACRGLPAELAAKLVVKQALKKSGLKDDTTCVVVDIIPSDYRLTSPQLSPKRNQSKFKSLLFGRRSHSSIGKLGGKSASFGSVEELFEEGSAMLEERLGRNLSLKATSAPLRCAICQVDQEPFESMMTEKGGSYCSSPCAPWGGPYLCLECRKKKDAMEGKRSSHSTACR |
| Os08g39100 | MGSCLSSDLPPRAGAGAGASPGWPQRWRRRRQRGVERGGAVSGGGGGVFSIGVGGKKLHHGGGGGGEMTEEELAKVEGRVCVNGASAAACLHTQQGRKGTNQDAMVVWENFNTSDSVFCGVFDGHGPYGHFVAKKVRDSLPVKIRTLWKTSANEDTSSHQNGSISGSVNSEESPVVDDEWGEYADDSEKLPEMFLPLKQSYFKAFKLMDKELKMHPTVDCFCSGSTAVTLVKQGLDLVVGNLGDSRAIMGTRDAANNLTAVQLTVDLKPNLPREAARIQQCRGRVFALQDEPEVARVWLPNNDSPGLAMARAFGDFCLKDYGLISVPQISYRRLTEKDEFIILATDGVWDVLSNKEAVDIVAAAPSRATAARALVDCAVRSWRLKFPTSKSDDCAVVCLFLDHAKSPDLIQENESEEETTEDVAIPDTVAKVDQDIAQGDAHISSEEQITEPALQHSYTLRDVDEIVPVEEPPVSKEPERCGSARSLADCISTNEEEEWSALEGVTRVNSLLNLPRILSGEKRSTSWRKRR |
| Os09g14540 | MAHQKREATSDNGGGDEEWASKRPKVVGAAAEKEHILTSDASHETNGDEAQGGDASRKENTVSTNPCVSDEKAATNSNVSSGHGVILTSVEADAAEDKGCRHTMEDAWVLLPDASMESPGNLRCAHFAIYDGHGGRLAAEYAQKHLHQNVIAAGLPRELMDVKAAKKAIIEGFRRTDECLLQESTKGNWQDGATAVCVWVLGQTVVVANAGDAKAVLARSTSADGEGAVDDAKSQLKAIVLTREHKAIFPQERARIQKAGGSVGPNGRLQGRIEVSRALGDRQFKKVGLIATPDVHSFEVTRKDHFIILGCDGLWGVFGPGDAVEFVQNQLKETSSATLAVRRLVKEAVRERRCKDNCTAVLIVFKH |
| Os09g15670 | MSMAEVCCDSAVVVGAEAEARARARAGRRRRAGVEGAGRWNATATAAGVAAEEAATRKRRASGGEAGLVVVAKRHGAASVAGRRREMEDAVSLREAFAAPANGEVAAARCDFYGVFDGHGCSHVADACRERMHELVAEEMGAGSPAAAAREPASWTETMERSFARMDAEVIAGCRAESGSCRCEGQKCDHVGSTAVVAVVEESRVVVANCGDSRAVLCRGGAPVQLSSDHKPDRPDELERIEAAGGRVIFWEGARVLGVLAMSRSIGDAYLKPYVTAVPEVTVTGRSDFDECLILASDGLWDVVSNEAACEVAQSCLRRGRQRWCAEAAAVLTKLALARRSSDNISVVVVDLRRGNAL |
| Os09g28560 | MAMTAAAVTVPLGVLLRREVTSERMERPDVLCGEAARSRKGEDFTLLLAEAGERVAGDPSTSFSVFALFDGHNGSGAAMYAKKNLLNNLLRAIPSGLSRDEWLAVLPRALVAAFVKTDKDFQAVAETSGTTVTFVVIDEWVVTVASVGDSRCILESADGSLYHLSADHRFDSNQDEVQRVTACGSKVGKLNLVGGPEVGPLRCWPGGLCLSRSIGDMDVGECIIPVPHVKQVKLSNAGGRIIIASDGVWDDLTFEMALECSRGFPSDIAANRIVNEAIHPRGLRDDTTCIVVDILPPEKLAPSPPTKRQGKIVFNNMFRRKHTDVSFILDREYAEPDEVEEIFDDGSAMLSKRLAAGYALQSMFEPFSCAVCQVQLKAGQGISVHSNPLQHEKLQGWQGPFLCQSCNEKKDAIEGKRPPRDS |
| Os09g38550 | MGVYLSTPKTEKYSGEGGNDRLRYGLASMQGWRTTMEDAHTALPRLDECTSFFGVYDGHGGKAVSKFCAKHLHLQVLKNEAYSSGDLATSVLKSFFRMDEMMKGQRGWRELAELGDKGQKFTGMLEGIIWSPKPGESDKPEDTWTEEGPHSHFPGPTSGSTACVAIIRNDELIVANAGDSRCVLSRKGRAYDLSKDHKPDLDAEKERILNAGGFIVAGRVNGSLNLARAIGDMELKQNEFLPAERQIVTAEPELNTVKLSEDDEFIVLACDGIWDCMSSQEVVDFVHKEMNTEDSLSAVCEKLLDHCLAPVSGGDGCDNMTVIIVKFKKPSKSAATSSTNQSVSSEEMRPNELDDGPSDPNK |
| Os10g22460 | MAELPLAAGLLDLRPCKLAPPPPPPLPVSPSPRHHRRPHSTATACRAAPDLHSSTELADGSIVFRFARPRDDDDEEQQQRRADAVAPEAAAVVESGLDGDAAAAAEPEARDGGGEGEVTATATGLDAEEVVASGGAEATATSGLEDAGEEASDGSTARDSDTDVDTESSASTAADDDQPAEFAVPPPPAEEVCNKVDWEKDTSEVKNTDRMVPVASSTLVLASGAAILPHPSKAATGGEDAYFIACDGWFGVADGVGQWSFEGINAGLYARELMDGCKKFIMENQGAADIKPEQVLSKAADEAHSPGSSTVLVAHFDGQFLNASNIGDSGFLVIRNGEVYQKSKPMVYGFNFPLQIEKGDNPLKLVQNYTIELEDGDVIVTASDGLFDNVYEQEVATMVSKSLQADLKPTEIAEHLAAKAQEVGRSAAGSTPFSDAALAVGYLGFSGGKLDDIAVVVSIVRKSEI |
| Os10g39540 | MSAGWWGPQDLGAGTLSLISPTDIRPGFLPVFRSGSCADIGTKSYMEDEHVCVDNLIEHLGVRTPVIPAPGAFYGVFDGHGGTDAACFVRKNLLRFIIEDGHFPSSIEKAIRSAFVRADHAIADSHSLDRNSGTTALTALIFGRTLLVANAGDCRAVLGKRGRAVELSRDHKPSCRSEKIRIENLGGTVFDGYLNGQLSVARAIGDWHVKGSKGSISPLTAEPEFQEVRLTEEDEFLIIGCDGLWDVMTSQCAVTMVRKELMTHNDPERCSQELVQEALRRNSCDNLTVVVVCFSSDPPPQIEVPRFRVRRSISMEGLHMLKGALDSNA |
| Os10g39780 | MLSAAMEYLRSCWGPASSPAGRPRKGSDAAGRQDGLLWYKDAGQLVAGEFSMAVVQANNLLEDHSQVESGPLSTTDPNLQGTLVGVYDGHGGPETARYINDHLFNHLRGFASEHKCMSADVIRKAFRATEEGFFSVVSSQWSMRPQLAAVGSCCLVGVICAGNLYIANLGDSRAVLGRLVKGTGEVLAMQLSAEHNASFEEVRRELQAAHPDDPHIVVLKHNVWRVKGIIQITRSIGDVYLKKPEFNREPLHSKFRLQETFRRPLLSSEPAIVVHQLQTTDQFIIFASDGLWEHISNQEAVDLVQHNPRNGIARRLVKAAMQQAAKKREMRYSDLKKIDRGVRRHFHDDITVVVVFFDSNAITTANWSRPSVSLRGGGVTLPANSLAPFSVPT |
| Os11g01790 | MGICCSKGKEELEEEGFPWKHDAFFHDQLWSAGVSMHTKQGWKGANQDAMTTCQDFAGHKGQIFCGVFDGHGPLGREVARHVRDVLPVKLSSSLALKTEQDPSSNTDKETLEKSDCTSLSDTSNEKQLLSTWKNIFVKTFEDVDEDLRQHSGIDCICSGTTAVTVVRQGDHLIIANLGDSRAVLCTRDSKDRPISVQLTTDLKPNLPSEAERILNSKGRVFAMDDEPDVPRMWLPDQDAPGLAMARAFGDFCLKSHGLICTPEVYYRKLSAKDDFLVLATDGIWDVLSNKEVIKIVSSATDHSKAAKQLVERAVRTWRRKFPTSMVDDCAVVCLFLKPSPSSSESTPGDAKPPQAVSFTGSFRKVLGGGGGEAEEGTNVWRALEGVARVNSVVRLPRMGAVLSWRRRSTSLEEDDEARID |
| Os11g13820 | MACMLCCSPLPALSSPATGGAAVVGFSSSSRRKAAHVHPAVAVKDHDLSSSSPAACGDEGGGAVVIEEEAHPSMMMSVAAPAMKKKAVAARWRPPRLVVPAVAGADEAMAAAAAVKAAKEKEEEEAMEVEGEGFWVASRRGLRHAMEDGYGVITHKIEGHSQMAFYGVYDGHGGRAAVDFVAGRLGNNVVAAAEKQRLSEKASSPAAADHVAAAIRAAYLATDSEFLSQGTRGGACAATALVIDGDLYVANLGDCRAVISRHGAAAALTSDHTPARDDERSRIESSGGYVSCGSNGVWRVQDCLAVTRSFGDGGLKRWVVAEPEVSRTPLAGAGCEFLVIASDGLWNKVSNQEAVDAVAAGHYSVDSCRRLVDMARRRGSRDDVTVMVVDLKRFLNC |
| Os11g22404 | MGTCLTTAEQRAMEVPAASVKGGGGRRSDEEAPGRIAGNGAGNVACLFTRQGKKGTNQDAMVAWENYNGRSDTVFCGVFDGHGPHGHLIARKVRDILPSRLCDLIYEDCGDSPTSNSDVSTLEENLSPYADAECRSPTLAGQKEHQEFFNAMKESFRKAFKNVDKELKLQRNIDSICSGTTAVTLIKQGHDLIVGNLGDSRAVLGTRDQNDKLVAHQLTVDLKPDHPREARRIRRCNGRVFAHQDEPDVARLWLPNCNSPGLAMARAFGDFCLKDFGLISVPDVTYRQITEKDEFIVLATDGVWDVLSNQEVVDVVASCSGRFAAARSVVDLANETWRFKYPTSKTDDCAVVCLFLNKYEVTGGLSGQPGYSPRMPALSGITRPNSKRVTPDDVDDGSDSNVSGDERSLDGFTRLNTLLALPKFGDTSPTKK |
| Os11g37540 | MRLGCSGRRRRLLRAALLRLVVLVLVAPPRRCAGESATCLAVYREGGAPAVFQSAHCPRWTLLAPSAGSGGEGDGDRRSSSSSPPPPPHPRGCHVAVDRGRRRSQEDRAVCALGIRIPFIGIYHKIKEVDVGVVAVFDGHNGAEASEMASKLLLEYFLLHVYFLLDGIYSIMFRKSTGKLTYKEVTILNNVINLYKEDQSSHSKGSCWALPAILDRSFHMEVLKESLLRAVHDVDLTFSKEALRNNFESGSTAAVILIVDGQIIAANVGDSKAFLCSESHDSSIDKKTSVVSGKRRRKRNSNNRDDFALANYDGPFYNVKELTKDHHPDREDERSRVEAAGGYVLEWAGVHRVNGELALSRAIGDVPYKRYGVIPTPELTEWQSLSANDTFLIASSDGVFEKMTMQDVCDLMLRVKLGVNQELGSFAVTQRNLADYVVDLALEKGTTDNVAAVIVPLGSHYSSKVTLEDWYMLEENSKTSISPLQTIPYQQKSGRFNVQ |
| Os12g01770 | MDGVPDAQRTTSPSMIKQQNYFNYPYAFNSILLSTPSFLPSFLPSYLYEVPAAEEAMGICCSKGKEELEEGFPWKHDAFFHDQLWSAGVSMHTKQGWKGANQDAMTTCQDFAGHKGQIFCGVFDGHGPLGREVARHVRDVLPMKLSSSLALKTEQDPSSNTDKEALEKSDCTSLSDTSNEKQLLSTWKNIFVKTFEDVDDDLRQNSGIDCICSGTTAVTVVRQGDHLIIANLGDSRAVLCTRDSKDRPIPVQLTTDLKPNLPSEAERILNCKGRVFAMDDEPDVSRMWLPDQDAPGLAMARAFGDFCLKSHGLICTPEVYYRKLSEKDEFLVLATDGIWDVLSNKEVIKIVSSATDHSKAAKQLVERAVRAWRRKFPTSMVDDCAVVCLFLKPSPSSEESTHVDAKAPQVVSFTGSFRKALGGGGGGEAEEGTNVWRALEGVARVNSVVRLPRMDAVLSWRRRSTSLEEDDDARID |
| Os12g09640 | MLCGVEILCSVVAVVARVLGRALMTICSPKFSTAGLIGSPTTAWGGGGAKKKVHMESEKQQLPPPPSPPALVLPAFKRNCTDQEQRAPATSTKAARGRPPRLVIPAAAPVAVARGGVDPFGGRETDVATETEVKGEGFCLASRRGVRHAMEDGYGVITRHKIEGGSQLAFYGVYDGHGGRAAVDFVADKLGKNVVTAAAAATTMSRHQAAGSSSPSQQRREEEDDVTAAIRAAYLTTDSEFLSQGVRGGACAATALVKDGELYVSNVGDCRAVLGSRGGVATALTSDHTPGREDERLRIESTGGYVSCGGSGVWRVQDSLAVSRAFGDAGVKQWVTCEPETARVSLAADGDCRFLVLASDGLWCKVSNQEAVDAVAAAAAAAAGVAGSTDPCKELAAMARSRGSRDDITVMVVDLQPFLPV |
| Os12g39120 | MLLRWLARPAERCLGRGGGGGGGGGGDGLLWHAELKPHASGEYSIAVAQANAELEDQGQVVTSPAATFVGVYDGHGGPEASRFISSRLFPHLHRFASEQGGMSTDAIKRAFHATEEEFLHMVKRSWLKQPQIASVGSCCLVGAITDNVLYVANLGDSRAVLGRRGPDGREVVAERLSNDHNVAEEEVRKELTEQHPDDSRIVIYTRGVWRIKGIIQVSRSIGDVYLKKPEFARDPIFRQYVCSIPLKRPVMTAEPSIKEHQLRQQDLFLIFASDGLWEQLTDKAAVDIVFKNPRAGIAKRLVRAALTEAARKREMRYTDIKHIERGSRRNFHDDITVVVVYLDHHKHGVRPNLGNRNSFRFTNAPVDIFSGSSEEVDHHPLRLNLAMDGAVG |

**Table S2: The primers used for real-time quantitative PCR in this study**

| **Gene name** | **Forward sequence** | **Reverse sequence** |
| --- | --- | --- |
| BdPP2C1 | GATGGCTTGTGGGAACACTTGA | ATGGAAATGCCTGCGAACCT |
| BdPP2C2 | GGGGATTGCTCGCCCTGCTT | CCGAACGCCCTCGTCACCTT |
| BdPP2C3 | GAGCAGGGTATTCACGCTGT | TAATGACATCGCCAGTCGCA |
| BdPP2C4 | GAGCACAATCTGGCAAGTAC | AGGTGGCTTCCAATCGTAAT |
| BdPP2C5 | GCCCTGTTATTGCGTTGAA | GCCCGTACTAATCGGTTTGC |
| BdPP2C6 | GACAAGGTGGGCAACCAGGAG | GTTTGAGGTGGACGACGAGGAC |
| BdPP2C7 | GATTGCCCGCAGAACTGG | GGATGGGATGATGTCAACTACC |
| BdPP2C8 | AAGTCGTTGACATGGTGGCTAT | CCCGTGCCGCTGTAGTTAG |
| BdPP2C9 | GGATTACGGTGTCATCTCGG | CGCCTTTGAACGGCTTGG |
| BdPP2C10 | AGGAATGCGTATCTACTAACCG | AACTGCTCGTGAATCTCCAA |
| BdPP2C11 | CTGTTGGCTCATGCTGTCTG | CAATACTTCTCCAGTGGCTTT |
| BdPP2C12 | CGATTTCATCCACGAGCACA | TCGCATCCTTCTCCACCTAA |
| BdPP2C13 | TGTCGGATTGTTCGGTGTTT | GCATCTCGGTTGTGGCTGGT |
| BdPP2C14 | AAGCTCTCTGCGCATTTCCT | AGCCGACGATGACAGTGATG |
| BdPP2C15 | GTGAAGCTCCCTCCGGATTT | ATGCCACGACGACTGTGATA |
| BdPP2C16 | AGGCGATTGAGATGTCCAGG | TGAGCAGCCCATTCAGGTAT |
| BdPP2C17 | GAGATGAAGGCCCGAACTCC | GGGAACTCCATGAGGTCGTC |
| BdPP2C18 | GAGATGAAGGCCCGAACTCC | GGGAACTCCATGAGGTCGTC |
| BdPP2C19 | CGGCAAGGCTATTCCACTCT | CCATCAACCCTCCAAGTATCA |
| BdPP2C20 | TTGCTGATAAATGCTCCCACC | TCGTCATCCACATAGCCACC |
| BdPP2C21 | GCAGACAAGTTGGCCTACAC | CTAGGCAGCTTCCTGCACTT |
| BdPP2C22 | TCAACTTGCGGCGATAACTG | GAAAGGACCTTGTGCCAGAC |
| BdPP2C23 | AGACGGATGGGTTGTCACTG | GTCACCACCACTTGCTGTTA |
| BdPP2C24 | GATGTCAGGGACGACGGCAACC | TGCTGTCCGCCGCAGAGGTT |
| BdPP2C25 | CGTGGTGAATTACCGAGGAACA | CCGTCAGAGGCCAGTATCAAGAA |
| BdPP2C26 | TGCTGATGAAGGGAACCGAC | TCCTGCCTGAACTGCTTGAG |
| BdPP2C27 | GAAGGGGGTCATGGAGAGGA | AGTTCGAGACGACGAGATGG |
| BdPP2C28 | CCCGGCAAAGCGAACAAAAA | ACACCTGGATGATGCCCTTG |
| BdPP2C29 | GTTTTGTGTTCCGAGGGTGC | GATTGCGCCAGCTTCATCAG |
| BdPP2C30 | ACAACGTTTGGCGTGTGAAG | TTTCCCGGAGTCGGAACTTG |
| BdPP2C31 | TGGTGCCAAGCTAATAGGCG | TCGTAGACGGCGAAGAAGTG |
| BdPP2C32 | TCAGGTCGTTCCGATGTTGG | TCCAAAGGTGCCTTACGCTC |
| BdPP2C33 | TCAGAGAGGTGCAGGTTAGGT | TCTTGTCGCGGCAATCAGAA |
| BdPP2C34 | ACAAGCCCAACAGGGAAGAC | ATCTGTCACCAATGGAGCGG |
| BdPP2C35 | CCGGAGTTCATGAAAGATACAAAAC | CAATAGCTTTGCCCGCCTTG |
| BdPP2C36 | ACGATGCAGCATTGGTTTGT | GGAACTCGTCCTGCTCTGTC |
| BdPP2C37 | GCGGATTATCTCGTGAGGCT | TTGGCTTTCCTCCGAGGTTT |
| BdPP2C38 | TTAGTGATCGCGAACGTGGG | GCAAGTTGGGCTTGAAGTCG |
| BdPP2C39 | GAGGTGACGCAGAGAACGAT | AATGGATGGTCCACAAGGCG |
| BdPP2C40 | GTGATTGTGGTCTGCTTCGC | CCAGCCAGCTCCTTAGGTTG |
| BdPP2C41 | AAGGTGTTCGACCGATCCAG | CAAACGCTCTTGTGACCTGC |
| BdPP2C42 | CTTCTCCCTTCTCTCCCCCA | GTGGAACTTGTCGAGGTCGT |
| BdPP2C43 | TTGCTTGCAGGTTGTATCGC | ACCTGCTGCTCTAATTCGCT |
| BdPP2C44 | GGTGCTAACTTGGGAGCTGT | CGTCTTACCCGCGATCTCAT |
| BdPP2C45 | CAGCCCGGAAGTTGACAGAT | GGGCGAACTGCTCGTCTTAT |
| BdPP2C46 | GCAGCTGGTGGCAGAGTTAT | CTCGTCTTTGTCTGTCCGGT |
| BdPP2C47 | GAGTCCGATAAGGCGTGCTC | GTCGTAGATCCACCACGACG |
| BdPP2C48 | TAATAAAAGCCGCCCTGCGA | CAGTGAAAGCAATGGGCCAC |
| BdPP2C49 | GCTGATGGTGTTGGTGGTTG | CCTTTCACGGTTGTGTTCCG |
| BdPP2C50 | GTGGCGTTCTTGCTGTTTCA | TGCACTTACATCATTAGTTACAACA |
| BdPP2C51 | TTGGTCTCCGAAAGGCAGTG | CAGAGTGGGGTCCCTGGTAA |
| BdPP2C52 | AACGAGGAGGTGGTGTCCAT | ACGACGGGTACTTGGTCTTC |
| BdPP2C53 | GTGGCAGAGAGAAGACGGTA | GGCTAACAGGCTGTCCATCA |
| BdPP2C54 | TTCCTCCAGAAGGTTCACGC | TGGTCATCAGGGCTTGTTCC |
| BdPP2C55 | GGTAAATGGCCAACTTGCGG | CTGCGGGTCCTTGACTGATT |
| BdPP2C56 | GAAGGATTGACGTGGCCTGA | CATCTTCACCACCTGTCGCT |
| BdPP2C57 | ATTCCCTCCAGCTCATTGCC | GAGATCAAGCTGAGTGCCCC |
| BdPP2C58 | CAAATGTTGGGGACTCCCGT | CCCGCCTAACCTCCTCAAAT |
| BdPP2C59 | TGTGGCGTCTTTGATGGTCA | CTTGCGGAAATGCTTCCGTT |
| BdPP2C60 | GTGAGCTCGATCATCCAGGG | TTGTCTGCGAGGTTTGGAGG |
| BdPP2C61 | TTGATGGGCATTTTGGCTGC | AGTTATGTTCACAGCGCGGA |
| BdPP2C62 | CGGGACAGAATTGGGCATCT | CCATTTGTTCTGCACGAGGC |
| BdPP2C63 | TACCACCACACGGTCGATTC | TTGAAGTAGGACTGCTGCGG |
| BdPP2C64 | GCGCATTTCTTTCCAGACGG | AAGGAATGGACCGTCCCAAC |
| BdPP2C65 | GGACCGACTCCTAAACGGAC | GAGATGAGAGTCTGTGGCCG |
| BdPP2C66 | TAAACGGACTGGATTCCGCC | GGGATTCGTGGGTCGATCTC |
| BdPP2C67 | TTACTCGGGCATTTGGTGCT | CAAAAACTGGTCCCTTGCCG |
| BdPP2C68 | CGGCAAGGGACCAGTTTTTG | TTGCTGCACGTGAAAGAAGC |
| BdPP2C69 | CCACGGACAACCTAACAGCA | CAGCTGATATGGTCCGCTCA |
| BdPP2C70 | ACAGCCGAGCCATCAATCAA | CAATATCCACCGCCGCTTTG |
| BdPP2C71 | GTGAGCTCGATCATCCAGGG | CATGGCCAAGCCGAAATCTG |
| BdPP2C72 | GAATGGTCGTTTGGTTGCCC | CAAAGGCGAGCAACATCTGG |
| BdPP2C73 | CTGCGGAGTATGCACAGACA | TTCGTGTCGACCAGTGATCC |
| BdPP2C74 | AGACTGCAAGGACGCATTGA | AAGCCATCGCACCCAAGAAT |
| BdPP2C75 | ACCTGAAGCCGTTCGTATCG | GATTCCTCGGCCGTAGATCG |
| BdPP2C76 | ACTTGCATCGTGGTGGACAT | AGGGGACCCGTCCTCAAATA |
| BdPP2C77 | GTGATGGCATTTGGGACTGC | GGGTCATCAGGCTCATTGGG |
| BdPP2C78 | AACGAGTTCTTGAGCCAGGG | GTGTGGTCGGAGGTTAAGGG |
| BdPP2C79 | CAGATCTTCTGCGGGGTGTT | CTTCAGTCTTCGGTGGCAGT |
| BdPP2C80 | TGCTATCCACCTCACAAGGC | GAGCACACAGCTTGGACTCT |
| BdPP2C81 | TAGTTGTTCACGGGGGCTTC | AAAGGCTAAAGCCGTTCCGT |
| BdPP2C82 | TGGACGCATCCAGACATAGC | CAGCTCGAGAATCCCCAACA |
| BdPP2C83 | AGACAGCCCGTGACAACAAA | GAAGGATGTGGCAGTGGGAA |
| BdPP2C84 | ACTCGATTCCACCTCTCCGA | CTTGCTGGTTTGTCAGGTGC |
| BdPP2C85 | TGGCAAGGGTCGAAGTAAGC | AGTAACTTGGCACACGGTCA |
| BdPP2C86 | TATGCAGGGGAGGAGATGCT | CCCACATCACGAAACCTCCA |

**Table S3: The expression profile date of *BdPP2C* genes in various tissues.**

|  | **Tissure** | **Root** | **Stem** | **Leaf** |
| --- | --- | --- | --- | --- |
| **BdPP2C** | **-ΔCT** | **-ΔCT** | **-ΔCT** | **-ΔCT** |
| BdPP2C1 | 0.366±0.245 | 0.833±0.002 | 1.52±0.314 | -1.145±0.405 |
| BdPP2C2 | 0.172±0.25 | 0.721±0.049 | -0.115±0.291 | 2.248±0.059 |
| BdPP2C3 | 0.723±0.235 | -0.214±0.011 | 1.726±0.189 | 1.82±1.024 |
| BdPP2C4 | -2.201±0.281 | -2.333±0.02 | -1.352±0.226 | -0.083±0.254 |
| BdPP2C5 | 0.895±0.274 | 1.646±0.026 | 1.763±0.21 | 2.669±0.071 |
| BdPP2C6 | 0.745±0.209 | -0.395±0.038 | 0.668±0.31 | 5.45±0.13 |
| BdPP2C7 | 0.579±0.296 | 0.487±0.063 | 2.13±0.264 | 2.008±0.145 |
| BdPP2C8 | -2.061±0.198 | -1.919±0.257 | -1.393±0.335 | -0.684±0.031 |
| BdPP2C9 | -0.541±0.26 | -0.59±0.002 | 0.645±0.241 | -1.202±0.561 |
| BdPP2C10 | 3.126±0.244 | 3.868±0.074 | 3.755±0.051 | 2.975±0.158 |
| BdPP2C11 | 0.742±0.253 | -0.089±0.051 | 1.696±0.291 | 3.831±0.046 |
| BdPP2C12 | 1.54±0.248 | 1.648±0.117 | 2.695±0.267 | 3.868±0.029 |
| BdPP2C13 | -3.858±0.24 | -3.731±0.032 | -4.768±0.217 | -5.286±2.017 |
| BdPP2C14 | -7.647±1.311 | -10.111±1.219 | -3.617±0.38 | -5.627±0.756 |
| BdPP2C16 | -0.718±0.186 | -2.176±0.062 | 0.254±0.218 | 1.611±0.03 |
| BdPP2C19 | -2.854±0.06 | -5.423±0.094 | -0.533±0.189 | -3.367±0.136 |
| BdPP2C20 | -0.328±0.127 | -1.636±0.132 | 0.397±0.26 | 0.478±0.434 |
| BdPP2C22 | -7.232±0.445 | -10.026±0.634 | -8.22±0.075 | -5.077±0.108 |
| BdPP2C23 | -3.25±0.553 | -3.117±0.037 | -1.482±0.258 | -1.429±0.083 |
| BdPP2C24 | 0.803±2.682 | -5.331±0.112 | 2.662±0.526 | -0.63±0.034 |
| BdPP2C25 | -0.533±0.252 | -1.436±0.054 | 0.801±0.208 | -3.239±0.028 |
| BdPP2C26 | -5.918±0.452 | -7.545±0.268 | -5.93±0.557 | -9.771±0.108 |
| BdPP2C28 | -6.162±0.604 | -6.691±0.451 | -6.121±0.549 | -4.114±0.088 |
| BdPP2C29 | -1.188±0.252 | -0.141±0.054 | 0.265±0.208 | 2.175±0.028 |
| BdPP2C30 | 0.571±0.252 | 0.049±0.054 | 1.699±0.208 | 1.808±0.028 |
| BdPP2C31 | 2.18±0.249 | 0.63±0.013 | 3.477±0.246 | 7.216±0.055 |
| BdPP2C32 | -1.811±0.636 | -3.524±0.299 | -3.937±0.127 | -1.346±0.502 |
| BdPP2C33 | -2.788±0.286 | -3.088±0.076 | -1.298±0.271 | 0.367±0.4 |
| BdPP2C34 | -0.615±0.337 | -5.531±0.086 | -1.83±0.069 | -4.226±0.465 |
| BdPP2C35 | -0.062±0.289 | -0.656±0.009 | 0.983±0.231 | 2.737±0.041 |
| BdPP2C36 | -6.684±0.445 | -10.952±0.109 | -8.18±0.075 | -5.73±0.41 |
| BdPP2C38 | 1.563±0.271 | 0.799±0.115 | 1.839±0.196 | 4.841±0.01 |
| BdPP2C39 | -2.181±1.33 | -4.42±0.356 | -2.051±0.381 | 0.864±0.029 |
| BdPP2C40 | 1.263±0.259 | 0.919±0.101 | 2.434±0.021 | -1.885±0.025 |
| BdPP2C41 | -6.405±0.431 | -7.534±0.149 | -5.233±0.212 | -3.674±0.08 |
| BdPP2C42 | -2.105±0.015 | -2.772±0.274 | -0.39±0.226 | -3.395±0.415 |
| BdPP2C43 | 0.207±0.346 | -3.807±0.037 | 0.496±0.123 | 3.016±0.107 |
| BdPP2C44 | -2.889±0.216 | -3.948±0.102 | -2.305±0.309 | -0.251±0.083 |
| BdPP2C45 | -0.563±0.304 | -1.079±0.115 | 1.549±0.215 | 3.408±0.01 |
| BdPP2C46 | -4.641±0.148 | -8.219±0.022 | -5.146±0.078 | -3.183±0.139 |
| BdPP2C47 | -6.245±0.493 | -7.102±0.113 | -8.141±0.049 | -2.372±0.059 |
| BdPP2C48 | -0.343±0.284 | 0.113±0.015 | 0.723±0.135 | -0.719±0.224 |
| BdPP2C49 | 0.176±0.391 | 0.257±0.09 | 1.43±0.171 | 2.637±0.078 |
| BdPP2C51 | -2.307±0.717 | -6.341±0.093 | -1.413±0.504 | -1.42±0.016 |
| BdPP2C52 | 2.778±0.594 | 2.109±0.102 | 3.576±0.031 | 3.901±0.131 |
| BdPP2C53 | 2.453±0.452 | -3.115±0.048 | 3.938±0.116 | -0.898±0.147 |
| BdPP2C54 | 4.625±0.491 | -3.993±0.114 | 4.101±0.073 | 4.584±0.013 |
| BdPP2C55 | 5.602±0.465 | 1.7±0.137 | 5.46±0.079 | 3.115±0.008 |
| BdPP2C57 | 1.214±0.368 | -2.517±0.027 | 1.452±0.051 | 1.099±0.046 |
| BdPP2C58 | 0.859±0.267 | 0.955±0.036 | 2.51±0.198 | 1.553±0.058 |
| BdPP2C59 | -2.173±0.254 | -1.539±0.027 | -0.434±0.156 | -0.42±0.182 |
| BdPP2C60 | -1.26±0.262 | -0.805±0.078 | 0.669±0.251 | 1.816±0.248 |
| BdPP2C61 | -2.901±0.092 | -9.493±0.139 | -2.92±0.453 | -7.788±0.108 |
| BdPP2C62 | -3.053±0.178 | -8.833±0.278 | -2.988±0.227 | -5.647±0.108 |
| BdPP2C64 | 0.73±0.319 | 1.28±0.023 | 2.604±0.237 | 2.309±0.201 |
| BdPP2C65 | -4.566±0.245 | -8.932±0.403 | -4.905±0.043 | -2.771±0.081 |
| BdPP2C66 | -4.956±0.535 | -9.654±0.023 | -4.233±0.122 | -2.794±0.032 |
| BdPP2C67 | 3.261±0.417 | -0.684±0.244 | 3.521±0.817 | 0.264±0.224 |
| BdPP2C68 | -0.676±0.322 | -0.845±0.059 | 1.223±0.12 | -0.614±0.34 |
| BdPP2C69 | 0.917±0.28 | 0.083±0.077 | 1.643±0.07 | 4.452±0.03 |
| BdPP2C70 | -2.669±0.296 | -5.336±0.25 | -2.903±0.299 | -0.29±0.109 |
| BdPP2C71 | -2.588±0.341 | -2.231±0.138 | -0.519±0.391 | -8.883±0.028 |
| BdPP2C72 | -3.153±0.248 | -3.106±0.004 | -0.267±0.344 | -3.121±0.074 |
| BdPP2C73 | 1.271±0.714 | -3.446±0.362 | 2.515±0.313 | -2.464±0.36 |
| BdPP2C74 | -1.313±0.24 | -1.873±0.117 | 1.345±0.173 | -0.802±0.111 |
| BdPP2C75 | 3.898±0.613 | -1.02±0.148 | 1.984±1.155 | -9.771±0.108 |
| BdPP2C76 | -3.236±0.297 | -3.773±0.117 | -0.872±0.198 | -3.72±0.028 |
| BdPP2C77 | 1.246±0.238 | 1.342±0.052 | 3.556±0.283 | 0.589±0.328 |
| BdPP2C79 | -2.065±0.249 | -7.81±0.198 | 1.16±0.243 | -1.744±0.082 |
| BdPP2C80 | -0.928±0.111 | -0.887±0.049 | 1.041±0.111 | 0.345±0.03 |
| BdPP2C81 | -0.647±0.206 | -1.786±0.129 | 2.487±0.066 | -0.887±0.053 |
| BdPP2C82 | 1.648±0.261 | 1.188±0.013 | 2.543±0.169 | 3.102±0.046 |
| BdPP2C83 | -7.805±0.504 | -4.285±0.129 | -2.644±0.122 | -5.259±0.215 |
| BdPP2C84 | -1.052±0.24 | -4.392±0.048 | 2.59±0.15 | -1.827±0.59 |
| BdPP2C85 | 3.565±0.415 | 0.079±0.114 | 3.271±0.073 | 1.667±0.1 |
| BdPP2C86 | 2.279±0.438 | -2.022±0.23 | 3.197±0.054 | 0.291±0.121 |

**Table S4: The expression profile date of *BdPP2C* genes under abiotic orhormone treatment**

|  | **Cold-3h** |  |  | **Cold-6h** |  |  |
| --- | --- | --- | --- | --- | --- | --- |
| **BdPP2C** | **average** | **fold-change** | **p-value** | **average** | **fold-change** | **p-value** |
| BdPP2C1 | -2.36495 | 5.15±0.02 | 0.015556 | -1.27967 | 2.43±0.26 | 0.0087 |
| BdPP2C2 | -2.50648 | 5.68±0.7 | 0.01137 | -1.11978 | 2.17±0.4 | 0.148489 |
| BdPP2C3 | -1.8225 | 3.54±0.25 | 0.055201 | -1.29527 | 2.45±0.52 | 0.004213 |
| BdPP2C4 | -1.95158 | 3.87±0.63 | 0.039109 | -1.58811 | 3.01±0.43 | 0.030615 |
| BdPP2C5 | -3.91362 | 15.07±1.06 | 0.004084 | -3.42971 | 10.78±2.61 | 0.000661 |
| BdPP2C6 | -4.00285 | 16.03±0.04 | 0.003125 | -3.02355 | 8.13±1.95 | 0.000278 |
| BdPP2C7 | -1.95663 | 3.88±0.02 | 0.04684 | -1.86888 | 3.65±0.35 | 0.002088 |
| BdPP2C8 | -1.3521 | 2.55±0.22 | 0.084421 | -1.06529 | 2.09±0.01 | 0.048152 |
| BdPP2C9 | -1.5482 | 2.92±0.28 | 0.043455 | -1.11923 | 2.17±0.07 | 0.089889 |
| BdPP2C10 | -2.38102 | 5.21±0.02 | 0.021743 | -1.39793 | 2.64±0.57 | 0.027877 |
| BdPP2C11 | -2.07989 | 4.23±0.61 | 0.028328 | -2.27548 | 4.84±1.72 | 0.0023 |
| BdPP2C12 | -2.64848 | 6.27±0.21 | 0.014687 | -1.48201 | 2.79±0.67 | 0.009904 |
| BdPP2C13 | -1.44539 | 2.72±0.41 | 0.061741 | -2.14168 | 4.41±0.74 | 0.003238 |
| BdPP2C14 | -1.39965 | 2.64±1.28 | 0.003223 | -0.42941 | 1.35±0.06 | 0.449324 |
| BdPP2C16 | -1.59804 | 3.03±0.2 | 0.048525 | -1.57084 | 2.97±0.34 | 0.000687 |
| BdPP2C19 | -1.83724 | 3.57±0.13 | 0.048233 | -0.7706 | 1.71±0.39 | 0.029633 |
| BdPP2C20 | -0.69746 | 1.62±0.23 | 0.322051 | -0.97026 | 1.96±0.59 | 0.331259 |
| BdPP2C22 | -0.78573 | 1.72±0.94 | 0.162852 | -1.93091 | 3.81±0.37 | 0.003964 |
| BdPP2C23 | -2.4122 | 5.32±0.84 | 0.0137 | -1.64823 | 3.13±0.91 | 0.07491 |
| BdPP2C24 | -0.63716 | 1.56±0.47 | 0.585086 | -4.0024 | 16.03±0.4 | 0.020016 |
| BdPP2C25 | -2.83897 | 7.16±0.06 | 0.006026 | -2.01176 | 4.03±0.6 | 0.000415 |
| BdPP2C26 | -0.13674 | 1.1±0.05 | 0.806836 | 0.036913 | 0.97±0.26 | 0.944013 |
| BdPP2C28 | -5.05992 | 33.36±0.22 | 0.00583 | -3.40003 | 10.56±0.75 | 0.000517 |
| BdPP2C29 | -3.85066 | 14.43±3.62 | 0.001251 | -2.51205 | 5.7±1.1 | 0.077173 |
| BdPP2C30 | -2.32214 | 5±0.83 | 0.028387 | -1.67318 | 3.19±0.16 | 0.017464 |
| BdPP2C31 | -1.5282 | 2.88±0.18 | 0.073629 | -1.24161 | 2.36±0.17 | 0.006385 |
| BdPP2C32 | -5.2655 | 38.47±0.05 | 0.053315 | -0.97003 | 1.96±0.11 | 0.736304 |
| BdPP2C33 | -3.96605 | 15.63±1.61 | 0.002924 | -2.94212 | 7.69±1.94 | 0.003222 |
| BdPP2C34 | 0.029924 | 0.98±0.43 | 0.928658 | 0.14227 | 0.91±0.35 | 0.692333 |
| BdPP2C35 | -2.36067 | 5.14±0.83 | 0.029782 | -2.11833 | 4.34±0.76 | 0.000864 |
| BdPP2C36 | -1.56561 | 2.96±0.24 | 0.048186 | -2.38728 | 5.23±0.57 | 0.04908 |
| BdPP2C37 | -2.76922 | 6.82±0.57 | 0.012766 | -2.36114 | 5.14±1.32 | 0.007937 |
| BdPP2C38 | -2.32157 | 5±0.28 | 0.034486 | -2.32031 | 4.99±0.08 | 0.023945 |
| BdPP2C39 | -6.0115 | 64.51±0.92 | 0.024793 | -3.19539 | 9.16±0.1 | 5.13E-05 |
| BdPP2C40 | -2.69613 | 6.48±0.41 | 0.010466 | -2.14912 | 4.44±1.1 | 0.001244 |
| BdPP2C41 | -1.30183 | 2.47±0.13 | 0.044862 | 0.681581 | 0.62±0.11 | 0.122138 |
| BdPP2C42 | -0.06566 | 1.05±0.26 | 0.714264 | -0.70492 | 1.63±0.33 | 0.088278 |
| BdPP2C43 | -0.64567 | 1.56±0.1 | 0.317141 | -0.23057 | 1.17±0.23 | 0.463005 |
| BdPP2C44 | -1.43916 | 2.71±0.52 | 0.043409 | -1.69188 | 3.23±0.21 | 0.056367 |
| BdPP2C45 | -1.97169 | 3.92±0.2 | 0.038302 | -1.08343 | 2.12±0.45 | 0.08543 |
| BdPP2C46 | -1.7855 | 3.45±0.39 | 0.004143 | -2.41677 | 5.34±1.31 | 0.001116 |
| BdPP2C47 | -3.04 | 8.22±0.71 | 0.00023 | -4.30046 | 19.7±0.55 | 0.049024 |
| BdPP2C48 | -1.01783 | 2.02±0.77 | 0.034618 | -0.62702 | 1.54±0.31 | 0.043265 |
| BdPP2C49 | -2.38956 | 5.24±0.33 | 0.024965 | -1.4115 | 2.66±0.54 | 0.003928 |
| BdPP2C50 | -3.19955 | 9.19±0.82 | 0.009086 | -2.63871 | 6.23±0.95 | 0.000234 |
| BdPP2C51 | -3.13413 | 8.78±2.23 | 0.005755 | -2.7023 | 6.51±1.22 | 1.42E-05 |
| BdPP2C52 | -2.79738 | 6.95±1 | 0.005781 | -4.07894 | 16.9±1.75 | 0.00131 |
| BdPP2C53 | -3.0452 | 8.25±0.41 | 0.000608 | -1.20275 | 2.3±0.07 | 0.026336 |
| BdPP2C54 | -1.73788 | 3.34±0.5 | 0.009838 | -1.01889 | 2.03±0.69 | 0.130616 |
| BdPP2C55 | -4.05823 | 16.66±0.09 | 0.002338 | -3.26599 | 9.62±0.09 | 0.006344 |
| BdPP2C57 | -2.98879 | 7.94±1.15 | 0.003493 | -2.03287 | 4.09±0.18 | 0.011221 |
| BdPP2C58 | -2.30027 | 4.93±0.32 | 0.01415 | -1.49143 | 2.81±0.62 | 0.000568 |
| BdPP2C59 | -2.59418 | 6.04±1 | 0.004772 | -1.17002 | 2.25±0.59 | 0.223096 |
| BdPP2C60 | -0.87548 | 1.83±0.23 | 0.022456 | -0.60392 | 1.52±0.12 | 0.09683 |
| BdPP2C61 | -3.03543 | 8.2±2.71 | 1.42E-05 | -2.51641 | 5.72±1.99 | 1.2E-06 |
| BdPP2C62 | -2.80774 | 7±0.77 | 0.001528 | -2.33963 | 5.06±0.26 | 0.000286 |
| BdPP2C64 | -3.33935 | 10.12±0.79 | 0.005872 | -2.27099 | 4.83±1.57 | 0.00079 |
| BdPP2C65 | -1.6448 | 3.13±1.69 | 0.001341 | -5.10303 | 34.37±0.88 | 0.004394 |
| BdPP2C66 | -0.69987 | 1.62±0.02 | 0.227923 | -0.48383 | 1.4±0.11 | 0.34275 |
| BdPP2C67 | -0.2544 | 1.19±0.28 | 0.443462 | -0.2835 | 1.22±0.03 | 0.008914 |
| BdPP2C68 | -2.01257 | 4.03±0.3 | 0.019917 | -2.27099 | 4.83±0.81 | 0.00079 |
| BdPP2C69 | -1.01771 | 2.02±0.24 | 0.154464 | -1.44842 | 2.73±0.22 | 0.010614 |
| BdPP2C70 | -2.84581 | 7.19±0.12 | 0.001612 | -2.14415 | 4.42±0.52 | 2.3E-06 |
| BdPP2C71 | -1.54848 | 2.93±0.06 | 0.041021 | -1.12995 | 2.19±0.15 | 0.011182 |
| BdPP2C72 | -1.82539 | 3.54±0.24 | 0.019048 | -1.00944 | 2.01±0.28 | 0.052197 |
| BdPP2C73 | -0.32861 | 1.26±0.15 | 0.265702 | 0.493108 | 0.71±0.11 | 0.19002 |
| BdPP2C74 | -0.96595 | 1.95±0.24 | 0.074542 | -0.43279 | 1.35±0.28 | 0.341414 |
| BdPP2C75 | 0.36832 | 0.77±0.08 | 0.668431 | 0.231742 | 0.85±0.11 | 0.202554 |
| BdPP2C76 | -0.87088 | 1.83±0.03 | 0.054985 | -0.91241 | 1.88±0.56 | 0.006506 |
| BdPP2C77 | -0.85055 | 1.8±0.41 | 0.238243 | -0.93359 | 1.91±0.59 | 0.138524 |
| BdPP2C79 | -2.57002 | 5.94±0.07 | 0.000214 | -1.81144 | 3.51±0.09 | 0.27644 |
| BdPP2C80 | -2.12419 | 4.36±0.17 | 0.018077 | -2.25247 | 4.76±1.47 | 5.5E-05 |
| BdPP2C81 | -2.13853 | 4.4±0.05 | 0.009674 | -1.209 | 2.31±0.75 | 0.003912 |
| BdPP2C82 | -2.57628 | 5.96±0.01 | 0.010998 | -1.69533 | 3.24±1.73 | 0.00202 |
| BdPP2C83 | -1.16245 | 2.24±0.15 | 0.025781 | -1.03391 | 2.05±0.28 | 0.238932 |
| BdPP2C84 | -3.09075 | 8.52±1.16 | 0.00507 | -0.65338 | 1.57±0.65 | 0.060163 |
| BdPP2C85 | -2.76765 | 6.81±0.02 | 0.001164 | -2.3436 | 5.08±1.94 | 0.021106 |
| BdPP2C86 | -2.4494 | 5.46±0.25 | 0.008378 | -1.75321 | 3.37±0.59 | 0.000419 |
|  | **Heat-3h** |  |  | **Heat-6h** |  |  |
| **BdPP2C** | **average** | **fold-change** | **p-value** | **average** | **fold-change** | **p-value** |
| BdPP2C1 | -0.90861 | 1.88±0.37 | 0.077434 | -0.59195 | 1.51±0.24 | 0.006991 |
| BdPP2C2 | -1.0719 | 2.1±0.18 | 0.012891 | -2.44049 | 5.43±1.28 | 0.012316 |
| BdPP2C3 | -0.67098 | 1.59±0.26 | 0.163349 | -0.993 | 1.99±0.17 | 0.007026 |
| BdPP2C4 | -0.91403 | 1.88±0.02 | 0.057359 | -2.52371 | 5.75±1.13 | 0.004922 |
| BdPP2C5 | -1.84112 | 3.58±0.52 | 0.00918 | -2.70714 | 6.53±2.34 | 0.002084 |
| BdPP2C6 | -1.19992 | 2.3±0.43 | 0.049115 | -0.28584 | 1.22±0.04 | 0.082248 |
| BdPP2C7 | -0.73872 | 1.67±0.34 | 0.192925 | -0.39865 | 1.32±0.42 | 0.000725 |
| BdPP2C8 | -0.54763 | 1.46±0.14 | 0.062896 | -1.19401 | 2.29±0.67 | 0.034902 |
| BdPP2C9 | -0.7968 | 1.74±0.31 | 0.011086 | -2.45501 | 5.48±1.69 | 0.004596 |
| BdPP2C10 | -0.63493 | 1.55±0.4 | 0.190387 | -0.23076 | 1.17±0.27 | 0.164325 |
| BdPP2C11 | -0.67484 | 1.6±0.23 | 0.037723 | -1.7977 | 3.48±0.3 | 0.004376 |
| BdPP2C12 | -1.10952 | 2.16±0.47 | 0.041554 | -0.94247 | 1.92±0.43 | 3E-07 |
| BdPP2C13 | -3.66328 | 12.67±1.33 | 3.1E-05 | -7.80727 | 223.99±8.39 | 5.21E-06 |
| BdPP2C14 | -0.92327 | 1.9±0.26 | 0.037289 | -2.00244 | 4.01±0.9 | 0.037432 |
| BdPP2C16 | -0.54447 | 1.46±0.22 | 0.102177 | -1.02087 | 2.03±0.33 | 0.002393 |
| BdPP2C19 | -0.8276 | 1.77±0.39 | 0.095576 | -0.62116 | 1.54±0.09 | 0.035682 |
| BdPP2C20 | -0.46048 | 1.38±0.07 | 0.043981 | -1.50842 | 2.84±0.32 | 0.055302 |
| BdPP2C22 | -4.05673 | 16.64±0.31 | 6.62E-05 | -5.14119 | 35.29±2.29 | 8.04E-05 |
| BdPP2C23 | 0.114832 | 0.92±0.05 | 0.761159 | -0.99961 | 2±0.76 | 0.19828 |
| BdPP2C24 | 0.307858 | 0.81±0.15 | 0.804684 | -4.36348 | 20.58±2.28 | 0.013071 |
| BdPP2C25 | 0.748569 | 0.6±0.13 | 0.085389 | -0.04879 | 1.03±0.06 | 0.801421 |
| BdPP2C26 | 0.810856 | 0.57±0.04 | 0.14381 | -2.51529 | 5.72±0.01 | 0.00671 |
| BdPP2C28 | 2.677618 | 0.16±0.02 | 0.048177 | -0.63288 | 1.55±0.32 | 0.260313 |
| BdPP2C29 | -3.41558 | 10.67±0.75 | 5.69E-05 | -6.33489 | 80.72±6.11 | 0.002067 |
| BdPP2C30 | -0.81582 | 1.76±0.33 | 0.024396 | -1.62613 | 3.09±0.3 | 0.009854 |
| BdPP2C31 | 1.404453 | 0.38±0.04 | 0.02751 | 1.707557 | 0.31±0.14 | 0.001062 |
| BdPP2C32 | -4.54191 | 23.29±0.01 | 0.079366 | -6.45382 | 87.66±0.14 | 0.051845 |
| BdPP2C33 | -1.05553 | 2.08±0.17 | 0.035559 | -0.45391 | 1.37±0.33 | 0.021007 |
| BdPP2C34 | 1.734411 | 0.3±0.01 | 0.005679 | -0.09023 | 1.06±0.16 | 0.842065 |
| BdPP2C35 | -1.13791 | 2.2±0.24 | 0.007184 | -1.91705 | 3.78±0.45 | 0.001374 |
| BdPP2C36 | -3.25949 | 9.58±0.26 | 0.001582 | -2.72644 | 6.62±0.36 | 0.039432 |
| BdPP2C37 | -2.17404 | 4.51±1.42 | 0.010512 | -1.70735 | 3.27±1.28 | 0.000133 |
| BdPP2C38 | -1.45087 | 2.73±0.81 | 0.090367 | -2.13929 | 4.41±0.62 | 0.020152 |
| BdPP2C39 | -6.23242 | 75.19±2.09 | 0.022132 | -1.85361 | 3.61±0.44 | 1.93E-05 |
| BdPP2C40 | -1.04305 | 2.06±0.41 | 0.022246 | -1.07358 | 2.1±0.18 | 0.012482 |
| BdPP2C41 | 0.203948 | 0.87±0.03 | 0.926178 | -1.49449 | 2.82±0.09 | 0.01481 |
| BdPP2C42 | 0.761324 | 0.59±0.1 | 0.055609 | -0.91351 | 1.88±0.63 | 0.083874 |
| BdPP2C43 | 1.219188 | 0.43±0.09 | 0.009105 | 2.499573 | 0.18±0.05 | 1.41E-06 |
| BdPP2C44 | -1.56403 | 2.96±0.003 | 0.000422 | -2.35787 | 5.13±0.22 | 0.022029 |
| BdPP2C45 | -0.5927 | 1.51±0.5 | 0.2434 | 0.147783 | 0.9±0.03 | 0.37001 |
| BdPP2C46 | 1.534338 | 0.35±0.07 | 0.004339 | -0.37209 | 1.29±0.6 | 0.10179 |
| BdPP2C47 | 0.9961 | 0.5±0.01 | 0.582979 | -4.36399 | 20.59±0.07 | 0.044003 |
| BdPP2C48 | -0.77134 | 1.71±0.37 | 0.070388 | -0.4868 | 1.4±0.07 | 0.043017 |
| BdPP2C49 | -1.41264 | 2.66±0.44 | 0.010298 | -2.00689 | 4.02±0.6 | 0.000275 |
| BdPP2C50 | -1.29534 | 2.45±0.14 | 0.046816 | -1.3265 | 2.51±1.04 | 0.000794 |
| BdPP2C51 | -0.45224 | 1.37±0.15 | 0.000257 | 0.367093 | 0.78±0.25 | 0.061159 |
| BdPP2C52 | -0.98853 | 1.98±0.17 | 0.05996 | -2.66885 | 6.36±2.17 | 0.018525 |
| BdPP2C53 | 0.490693 | 0.71±0.17 | 0.198308 | 1.370652 | 0.39±0.05 | 0.007963 |
| BdPP2C54 | -0.35945 | 1.28±0.04 | 0.025077 | -0.34188 | 1.27±0.11 | 0.381272 |
| BdPP2C55 | -2.8184 | 7.05±1.84 | 0.001302 | -1.9568 | 3.88±0.32 | 0.006346 |
| BdPP2C57 | -1.50837 | 2.84±0.2 | 0.000296 | -2.46628 | 5.53±0.43 | 0.004782 |
| BdPP2C58 | -1.26237 | 2.4±0.66 | 0.003502 | -1.9878 | 3.97±0.93 | 4.92E-05 |
| BdPP2C59 | -0.56162 | 1.48±0.12 | 0.000662 | -2.37077 | 5.17±1.1 | 0.013011 |
| BdPP2C60 | -0.12804 | 1.09±0.19 | 0.488666 | -0.61321 | 1.53±0.34 | 0.175697 |
| BdPP2C61 | -1.24003 | 2.36±0.37 | 0.00291 | -1.70235 | 3.25±0.15 | 6.83E-06 |
| BdPP2C62 | -2.03204 | 4.09±0.38 | 0.004392 | -1.18847 | 2.28±0.88 | 0.006622 |
| BdPP2C64 | -0.63062 | 1.55±0.38 | 0.079112 | -0.72409 | 1.65±0.11 | 0.044181 |
| BdPP2C65 | -1.40148 | 2.64±0.47 | 0.021768 | -5.64237 | 49.95±1.8 | 0.00267 |
| BdPP2C66 | -2.08645 | 4.25±0.2 | 0.007854 | -2.39997 | 5.28±0.07 | 0.006534 |
| BdPP2C67 | 0.152288 | 0.9±0.07 | 0.192621 | -0.95767 | 1.94±0.35 | 9.63E-06 |
| BdPP2C68 | -0.61003 | 1.53±0.24 | 0.080937 | -1.18477 | 2.27±0.35 | 0.002073 |
| BdPP2C69 | -0.43573 | 1.35±0.6 | 0.349964 | 0.499243 | 0.71±0.1 | 0.211001 |
| BdPP2C70 | -2.51435 | 5.71±1.57 | 0.000355 | -3.77492 | 13.69±3.89 | 0.000244 |
| BdPP2C71 | -0.6754 | 1.6±0.14 | 0.039908 | -1.05583 | 2.08±0.63 | 0.038826 |
| BdPP2C72 | -1.11578 | 2.17±0.65 | 0.001298 | -1.56567 | 2.96±0.34 | 0.022151 |
| BdPP2C73 | -0.32069 | 1.25±0.33 | 0.28248 | 0.240961 | 0.85±0.24 | 0.430945 |
| BdPP2C74 | -0.23451 | 1.18±0.34 | 0.567422 | 0.738898 | 0.6±0.05 | 0.140083 |
| BdPP2C75 | 2.501213 | 0.18±0.15 | 0.240551 | -1.92948 | 3.81±0.54 | 0.000244 |
| BdPP2C76 | -0.32307 | 1.25±0.67 | 0.379574 | -0.28899 | 1.22±0.17 | 0.29811 |
| BdPP2C77 | -0.80609 | 1.75±0.1 | 0.266881 | -0.17621 | 1.13±0.37 | 0.745681 |
| BdPP2C79 | -1.68106 | 3.21±0.46 | 2.75E-06 | -3.48292 | 11.18±0.33 | 0.035248 |
| BdPP2C80 | 0.199804 | 0.87±0.33 | 0.5572 | -0.66168 | 1.58±0.11 | 0.043609 |
| BdPP2C81 | -0.23465 | 1.18±0.04 | 0.290672 | -0.23114 | 1.17±0.55 | 0.387655 |
| BdPP2C82 | -0.08064 | 1.06±0.05 | 0.791934 | 0.344488 | 0.79±0.05 | 0.238229 |
| BdPP2C83 | 0.333309 | 0.79±0.33 | 0.294088 | -1.32326 | 2.5±0.68 | 0.164383 |
| BdPP2C84 | -0.9132 | 1.88±0.16 | 0.085949 | 1.323818 | 0.4±0.02 | 0.00599 |
| BdPP2C85 | -0.60266 | 1.52±0.05 | 0.009823 | -2.08703 | 4.25±0.52 | 0.00864 |
| BdPP2C86 | -0.59647 | 1.51±0.06 | 0.041489 | -0.87082 | 1.83±0.35 | 0.004418 |
|  | **PEG-3h** |  |  | **PEG-6h** |  |  |
| **BdPP2C** | **average** | **fold-change** | **p-value** | **average** | **fold-change** | **p-value** |
| BdPP2C1 | -0.6705 | 1.59±0.2 | 0.12049 | -0.08782 | 1.06±0.21 | 0.086867 |
| BdPP2C2 | -0.64568 | 1.56±0.07 | 0.00927 | -0.76992 | 1.71±0.11 | 0.244961 |
| BdPP2C3 | -0.61498 | 1.53±0.28 | 0.233071 | -0.64576 | 1.56±0.01 | 0.014401 |
| BdPP2C4 | -0.96955 | 1.96±0.06 | 0.01339 | -1.3252 | 2.51±1.14 | 0.041328 |
| BdPP2C5 | -0.94758 | 1.93±0.14 | 0.145862 | -1.19344 | 2.29±0.08 | 0.029112 |
| BdPP2C6 | -3.28706 | 9.76±1.06 | 0.004185 | -3.32049 | 9.99±0.03 | 5.81E-06 |
| BdPP2C7 | -0.49751 | 1.41±0.27 | 0.408064 | 0.094383 | 0.94±0.05 | 0.019616 |
| BdPP2C8 | -0.18835 | 1.14±0.29 | 0.508696 | -0.21282 | 1.16±0.35 | 0.600678 |
| BdPP2C9 | -0.50752 | 1.42±0.08 | 0.023503 | -0.81174 | 1.76±0.05 | 0.135545 |
| BdPP2C10 | -0.30583 | 1.24±0.13 | 0.446776 | 0.618111 | 0.65±0.17 | 0.002247 |
| BdPP2C11 | -0.767 | 1.7±0.14 | 0.024163 | -0.94139 | 1.92±0.43 | 0.03777 |
| BdPP2C12 | -0.80948 | 1.75±0.1 | 0.235523 | -0.58305 | 1.5±0.11 | 0.025632 |
| BdPP2C13 | -4.8546 | 28.93±0.39 | 0.000158 | -6.69694 | 103.75±15.12 | 9.6E-06 |
| BdPP2C14 | -0.82967 | 1.78±0.07 | 0.06393 | 1.979396 | 0.25±0.08 | 0.002745 |
| BdPP2C16 | -0.95856 | 1.94±0.15 | 0.089589 | -0.68343 | 1.61±0.14 | 0.009302 |
| BdPP2C19 | -0.59837 | 1.51±0.29 | 0.26316 | -0.55127 | 1.47±0.03 | 0.058187 |
| BdPP2C20 | -0.53435 | 1.45±0.02 | 0.273509 | -1.37472 | 2.59±0.63 | 0.073004 |
| BdPP2C22 | 0.356924 | 0.78±0.1 | 0.341226 | -1.19314 | 2.29±0.67 | 0.016199 |
| BdPP2C23 | -0.53819 | 1.45±0.02 | 0.015659 | -1.49321 | 2.82±0.71 | 0.102877 |
| BdPP2C24 | 0.319605 | 0.8±0.14 | 0.834826 | -0.86936 | 1.83±0.25 | 0.671771 |
| BdPP2C25 | -0.77652 | 1.71±0.04 | 0.113018 | -1.19298 | 2.29±0.5 | 0.01025 |
| BdPP2C26 | 2.615903 | 0.16±0.03 | 0.034876 | 0.967233 | 0.51±0.02 | 0.115393 |
| BdPP2C28 | -2.55636 | 5.88±0.41 | 0.053222 | 0.450407 | 0.73±0.05 | 0.299861 |
| BdPP2C29 | -0.6555 | 1.58±0.18 | 0.017458 | -2.54444 | 5.83±0.06 | 0.096307 |
| BdPP2C30 | -0.94991 | 1.93±0.05 | 0.01099 | -1.17968 | 2.27±0.23 | 0.034435 |
| BdPP2C31 | -0.66379 | 1.58±0.38 | 0.286472 | 0.3049 | 0.81±0.01 | 0.324513 |
| BdPP2C32 | -5.12341 | 34.86±0.1 | 0.057655 | -6.51795 | 91.64±0.04 | 0.04984 |
| BdPP2C33 | -1.07692 | 2.11±0.13 | 0.067404 | -0.21445 | 1.16±0.04 | 0.146643 |
| BdPP2C34 | 0.112218 | 0.93±0.09 | 0.156408 | -0.06357 | 1.05±0.05 | 0.834208 |
| BdPP2C35 | -0.8416 | 1.79±0.23 | 0.087915 | -1.65894 | 3.16±0.01 | 0.002948 |
| BdPP2C36 | -1.32558 | 2.51±0.15 | 0.046244 | 0.077367 | 0.95±0.04 | 0.937389 |
| BdPP2C37 | -3.57961 | 11.96±2.42 | 0.004148 | -3.10434 | 8.6±1.57 | 2.89E-05 |
| BdPP2C38 | -1.60625 | 3.04±1.12 | 0.069162 | -3.12177 | 8.7±3.03 | 0.004674 |
| BdPP2C39 | -4.83078 | 28.46±0.45 | 0.04822 | 0.238921 | 0.85±0.04 | 0.429979 |
| BdPP2C40 | -2.08483 | 4.24±0.82 | 0.00398 | -1.46646 | 2.76±0.48 | 0.004077 |
| BdPP2C41 | -0.92583 | 1.9±0.13 | 0.097682 | 1.805298 | 0.29±0.04 | 0.01763 |
| BdPP2C42 | 1.444408 | 0.37±0.09 | 0.007474 | 0.386189 | 0.77±0.16 | 0.278707 |
| BdPP2C43 | -0.56892 | 1.48±0.15 | 0.235612 | -0.13182 | 1.1±0.13 | 0.000584 |
| BdPP2C44 | -2.59804 | 6.05±0.09 | 0.000453 | -4.22622 | 18.72±2.69 | 0.001399 |
| BdPP2C45 | -1.55978 | 2.95±0.37 | 0.020475 | 0.297685 | 0.81±0.03 | 0.130145 |
| BdPP2C46 | -4.56698 | 23.7±2.19 | 0.000425 | -6.99332 | 127.41±32.73 | 7.29E-06 |
| BdPP2C47 | -0.05064 | 1.04±0.06 | 0.949385 | -4.24353 | 18.94±0.03 | 0.047519 |
| BdPP2C48 | -0.63017 | 1.55±0.3 | 0.179192 | -0.79379 | 1.73±0.09 | 0.053874 |
| BdPP2C49 | -0.43049 | 1.35±0.19 | 0.430819 | -1.16911 | 2.25±0.66 | 0.029569 |
| BdPP2C50 | -0.77403 | 1.71±0.24 | 0.087807 | -1.2187 | 2.33±0.06 | 0.012507 |
| BdPP2C51 | -0.4186 | 1.34±0.13 | 0.287899 | -0.37194 | 1.29±0.01 | 0.012937 |
| BdPP2C52 | 0.614414 | 0.65±0.07 | 0.181362 | -1.23278 | 2.35±0.29 | 0.056302 |
| BdPP2C53 | -0.52992 | 1.44±0.09 | 0.373993 | 0.295557 | 0.81±0.14 | 0.299554 |
| BdPP2C54 | 0.236873 | 0.85±0.02 | 0.338 | -1.58732 | 3±1 | 0.015235 |
| BdPP2C55 | -1.7527 | 3.37±0.61 | 0.019182 | -1.35004 | 2.55±0.7 | 0.025377 |
| BdPP2C57 | -0.76854 | 1.7±0.13 | 0.114044 | -1.01143 | 2.02±0.27 | 0.082521 |
| BdPP2C58 | -0.77126 | 1.71±0.01 | 0.188293 | -1.54136 | 2.91±0.8 | 0.005046 |
| BdPP2C59 | -0.58911 | 1.5±0.03 | 5.04E-05 | -1.45985 | 2.75±0.37 | 0.057654 |
| BdPP2C60 | -0.58115 | 1.5±0.09 | 0.011102 | -0.45793 | 1.37±0.05 | 0.221803 |
| BdPP2C61 | -0.09675 | 1.07±0.1 | 0.458429 | -0.16032 | 1.12±0.19 | 0.62453 |
| BdPP2C62 | -0.64725 | 1.57±0.36 | 0.141368 | 0.117877 | 0.92±0.13 | 0.772608 |
| BdPP2C64 | -0.73912 | 1.67±0.19 | 0.159908 | -0.37177 | 1.29±0.34 | 0.230877 |
| BdPP2C65 | 0.708318 | 0.61±0.02 | 0.403894 | -2.11844 | 4.34±0.13 | 0.078161 |
| BdPP2C66 | -0.41947 | 1.34±0.08 | 0.592983 | 0.896823 | 0.54±0.04 | 0.174925 |
| BdPP2C67 | 0.195172 | 0.87±0.01 | 0.695352 | 0.25516 | 0.84±0.2 | 0.433973 |
| BdPP2C68 | -0.26799 | 1.2±0.04 | 0.566173 | -0.72582 | 1.65±0.23 | 0.023021 |
| BdPP2C69 | -1.51911 | 2.87±0.28 | 0.052772 | -1.49752 | 2.82±0.23 | 0.00984 |
| BdPP2C70 | -3.30023 | 9.85±0.51 | 0.001028 | -4.04844 | 16.55±2.94 | 5.1E-05 |
| BdPP2C71 | -0.09424 | 1.07±0.08 | 0.844471 | -0.7573 | 1.69±0.09 | 0.130063 |
| BdPP2C72 | -1.63365 | 3.1±0.39 | 0.014577 | -2.10436 | 4.3±0.65 | 0.004761 |
| BdPP2C73 | -0.10227 | 1.07±0.13 | 0.803833 | 0.707104 | 0.61±0.17 | 0.065802 |
| BdPP2C74 | -1.09397 | 2.13±0.12 | 0.04472 | -1.0281 | 2.04±0.29 | 0.074614 |
| BdPP2C75 | -3.71832 | 13.16±0.6 | 0.000492 | -4.42853 | 21.53±0.08 | 1.22E-05 |
| BdPP2C76 | -1.68465 | 3.21±0.81 | 0.006205 | -1.98771 | 3.97±0.28 | 0.001056 |
| BdPP2C77 | -2.30742 | 4.95±0.71 | 0.020694 | -1.71428 | 3.28±0.82 | 0.027532 |
| BdPP2C79 | -0.39824 | 1.32±0.14 | 0.01916 | -2.35087 | 5.1±0.48 | 0.102315 |
| BdPP2C80 | -0.72712 | 1.66±0.07 | 0.093366 | -1.56834 | 2.97±0.43 | 0.00132 |
| BdPP2C81 | -1.01319 | 2.02±0.09 | 0.006678 | -1.2944 | 2.45±0.1 | 0.013618 |
| BdPP2C82 | -1.10341 | 2.15±0.08 | 0.022465 | -1.0928 | 2.13±0.97 | 0.009058 |
| BdPP2C83 | -0.06768 | 1.05±0.23 | 0.051794 | -1.22566 | 2.34±0.86 | 0.091497 |
| BdPP2C84 | -0.28471 | 1.22±0.11 | 0.560611 | -0.25631 | 1.19±0.06 | 0.368555 |
| BdPP2C85 | -1.24868 | 2.38±0.08 | 0.000321 | -2.24023 | 4.72±0.42 | 0.004514 |
| BdPP2C86 | -1.33991 | 2.53±0.28 | 0.003122 | -1.70299 | 3.26±0.71 | 0.001099 |
|  | **NaCl-3h** |  |  | **NaCl-6h** |  |  |
| **BdPP2C** | **average** | **fold-change** | **p-value** | **average** | **fold-change** | **p-value** |
| BdPP2C1 | -1.25179 | 2.38±0.33 | 0.015453 | -0.99984 | 2±0.03 | 1.01E-05 |
| BdPP2C2 | 0.028193 | 0.98±0.05 | 0.934953 | -0.78251 | 1.72±0.03 | 0.110803 |
| BdPP2C3 | -0.44846 | 1.36±0.05 | 0.305161 | -0.89684 | 1.86±0.04 | 0.000531 |
| BdPP2C4 | -0.08417 | 1.06±0.08 | 0.76069 | -0.81656 | 1.76±0.02 | 0.014965 |
| BdPP2C5 | -2.97306 | 7.85±0.14 | 0.000422 | -1.01407 | 2.02±0.15 | 0.002039 |
| BdPP2C6 | -2.69288 | 6.47±0.03 | 0.001346 | -1.185 | 2.27±0.25 | 0.018873 |
| BdPP2C7 | -0.82387 | 1.77±0.14 | 0.087968 | 0.008146 | 0.99±0.02 | 0.977662 |
| BdPP2C8 | -0.15366 | 1.11±0.16 | 0.497216 | -0.13639 | 1.1±0.24 | 0.637381 |
| BdPP2C9 | 0.331592 | 0.79±0.06 | 0.082634 | -0.58138 | 1.5±0.21 | 0.086487 |
| BdPP2C10 | -0.81314 | 1.76±0.07 | 0.075453 | 0.173224 | 0.89±0.02 | 0.594304 |
| BdPP2C11 | -0.32203 | 1.25±0.09 | 0.108728 | -0.54605 | 1.46±0.24 | 0.1071 |
| BdPP2C12 | -1.03665 | 2.05±0.04 | 0.026555 | -0.16059 | 1.12±0.03 | 0.62818 |
| BdPP2C13 | -5.34238 | 40.57±0.18 | 8.03E-07 | -1.46337 | 2.76±0.15 | 0.006654 |
| BdPP2C14 | -0.56689 | 1.48±0.06 | 0.194381 | 0.206806 | 0.87±0.02 | 0.605249 |
| BdPP2C16 | -0.16426 | 1.12±0.11 | 0.487833 | -0.39651 | 1.32±0.32 | 0.239391 |
| BdPP2C19 | -0.59874 | 1.51±0.02 | 0.127182 | -0.04835 | 1.03±0.07 | 0.871033 |
| BdPP2C20 | 0.011998 | 0.99±0.08 | 0.943038 | -0.95197 | 1.93±0.2 | 0.007479 |
| BdPP2C22 | 0.020296 | 0.99±0.18 | 0.940007 | -0.20907 | 1.16±0.02 | 0.87265 |
| BdPP2C23 | 0.05388 | 0.96±0.07 | 0.872966 | -0.29354 | 1.23±0.05 | 0.345855 |
| BdPP2C24 | -0.18756 | 1.14±0.04 | 0.555552 | -0.12624 | 1.09±0.29 | 0.267491 |
| BdPP2C25 | -1.05256 | 2.07±0.06 | 0.01807 | -1.60157 | 3.03±0.23 | 0.012974 |
| BdPP2C26 | 0.073243 | 0.95±0.04 | 0.670477 | -0.73352 | 1.66±0.03 | 0.080868 |
| BdPP2C28 | -0.33694 | 1.26±0.13 | 0.581649 | 0.260052 | 0.84±0.02 | 0.279425 |
| BdPP2C29 | -1.77398 | 3.42±0.11 | 0.000489 | -1.21106 | 2.32±0.22 | 0.002439 |
| BdPP2C30 | -0.03831 | 1.03±0.07 | 0.91743 | -0.57011 | 1.48±0.25 | 0.119495 |
| BdPP2C31 | 0.11656 | 0.92±0.03 | 0.781101 | -0.19614 | 1.15±0.02 | 0.019104 |
| BdPP2C32 | -1.44864 | 2.73±0.09 | 0.00107 | -1.341 | 2.53±0.17 | 0.004365 |
| BdPP2C33 | -2.31425 | 4.97±0.02 | 0.000346 | -0.30362 | 1.23±0.29 | 0.011342 |
| BdPP2C34 | 0.001698 | 1±0.01 | 0.99081 | -0.3267 | 1.25±0.09 | 0.336997 |
| BdPP2C35 | -0.27765 | 1.21±0.1 | 0.501712 | -1.05745 | 2.08±0.15 | 1.49E-05 |
| BdPP2C36 | -5.76829 | 54.5±0.11 | 4.46E-06 | -1.29182 | 2.45±0.67 | 0.005157 |
| BdPP2C37 | -3.67974 | 12.81±2.32 | 0.000939 | -0.85839 | 1.81±0.03 | 0.036293 |
| BdPP2C38 | -0.43593 | 1.35±0.13 | 0.594139 | -1.04663 | 2.07±0.31 | 4.02E-07 |
| BdPP2C39 | -3.60568 | 12.17±1.41 | 5.53E-05 | 0.119987 | 0.92±0.03 | 0.815288 |
| BdPP2C40 | -1.88249 | 3.69±0.21 | 0.00121 | -0.68085 | 1.6±0.18 | 1.27E-06 |
| BdPP2C41 | -0.34295 | 1.27±0.09 | 0.38661 | 1.089883 | 0.47±0.02 | 0.007764 |
| BdPP2C42 | -2.43528 | 5.41±0.49 | 0.002348 | -1.74443 | 3.35±0.25 | 0.001003 |
| BdPP2C43 | 1.292843 | 0.41±0.03 | 0.003891 | -0.62309 | 1.54±0 | 0.005494 |
| BdPP2C44 | -1.69886 | 3.25±0.35 | 9.77E-05 | -1.00777 | 2.01±0.24 | 0.019214 |
| BdPP2C45 | -0.00328 | 1±0.04 | 0.993901 | -0.77143 | 1.71±0.04 | 0.000789 |
| BdPP2C46 | -3.27471 | 9.68±0.22 | 0.000111 | -1.3942 | 2.63±0.08 | 0.011011 |
| BdPP2C47 | -7.53975 | 186.08±0.31 | 1.32E-05 | -1.04041 | 2.06±0.4 | 0.024274 |
| BdPP2C48 | -0.63967 | 1.56±0.01 | 0.067203 | -0.69079 | 1.61±0.11 | 8.86E-05 |
| BdPP2C49 | -0.28274 | 1.22±0.07 | 0.515883 | -1.07915 | 2.11±0.26 | 1.15E-07 |
| BdPP2C50 | -2.44523 | 5.45±0.58 | 8.18E-06 | -2.75476 | 6.75±0.39 | 1.52E-06 |
| BdPP2C51 | -0.11158 | 1.08±0.05 | 0.727885 | 0.029017 | 0.98±0.02 | 0.881377 |
| BdPP2C52 | -0.36761 | 1.29±0.08 | 0.604384 | -1.42757 | 2.69±0.21 | 0.000368 |
| BdPP2C53 | -0.28917 | 1.22±0.14 | 0.341526 | 0.071277 | 0.95±0.02 | 0.115813 |
| BdPP2C54 | 1.454966 | 0.36±0.18 | 0.000113 | 0.122228 | 0.92±0.31 | 0.659548 |
| BdPP2C55 | -3.49095 | 11.24±1.7 | 0.000404 | 0.613739 | 0.65±0.02 | 0.123709 |
| BdPP2C57 | 0.142575 | 0.91±0.02 | 0.599914 | -0.07002 | 1.05±0.22 | 0.832072 |
| BdPP2C58 | -0.28425 | 1.22±0.06 | 0.220221 | -0.9131 | 1.88±0.2 | 8.83E-08 |
| BdPP2C59 | -0.15299 | 1.11±0.06 | 0.333097 | -1.05951 | 2.08±0.29 | 4.51E-08 |
| BdPP2C60 | -0.1024 | 1.07±0.01 | 0.475659 | -1.00537 | 2.01±0.14 | 0.000121 |
| BdPP2C61 | -0.29047 | 1.22±0.05 | 0.379179 | -0.25836 | 1.2±0.02 | 0.105906 |
| BdPP2C62 | -0.53728 | 1.45±0.15 | 0.129285 | 0.232758 | 0.85±0.05 | 0.403095 |
| BdPP2C64 | -2.70374 | 6.51±0.37 | 0.000392 | -0.36635 | 1.29±0.12 | 2.11E-05 |
| BdPP2C65 | -2.10251 | 4.29±1.35 | 0.000765 | -0.03344 | 1.02±0.07 | 0.910566 |
| BdPP2C66 | -3.02498 | 8.14±0.96 | 1.09E-06 | -1.99263 | 3.98±0.22 | 7.56E-05 |
| BdPP2C67 | -2.21336 | 4.64±0.54 | 0.000841 | -1.54746 | 2.92±0.07 | 0.000487 |
| BdPP2C68 | 0.369955 | 0.77±0.03 | 0.354409 | -1.00838 | 2.01±0.08 | 3.89E-05 |
| BdPP2C69 | -2.37146 | 5.17±0.12 | 0.004148 | -1.09198 | 2.13±0.29 | 0.024638 |
| BdPP2C70 | -3.83828 | 14.3±0.25 | 4.01E-05 | -1.48281 | 2.79±0.11 | 0.012393 |
| BdPP2C71 | -0.16357 | 1.12±0.17 | 0.503888 | -0.54 | 1.45±0.04 | 0.120539 |
| BdPP2C72 | -1.62563 | 3.09±0.2 | 0.022918 | -1.29137 | 2.45±0.29 | 9.53E-06 |
| BdPP2C73 | -2.85314 | 7.23±0.6 | 7.9E-06 | -0.26243 | 1.2±0.17 | 0.471252 |
| BdPP2C74 | -0.19837 | 1.15±0.09 | 0.625395 | -0.27381 | 1.21±0.12 | 0.425467 |
| BdPP2C75 | -5.43026 | 43.12±0.92 | 0.012206 | -3.39113 | 10.49±0.2 | 0.004482 |
| BdPP2C76 | -0.70274 | 1.63±0.34 | 0.156155 | -0.50305 | 1.42±0.08 | 0.093379 |
| BdPP2C77 | -1.16682 | 2.25±0.07 | 0.131201 | -0.66464 | 1.59±0.24 | 3.65E-06 |
| BdPP2C79 | -0.92382 | 1.9±0.02 | 0.001939 | -0.32912 | 1.26±0.1 | 0.009403 |
| BdPP2C80 | -0.10781 | 1.08±0.04 | 0.775173 | -0.63123 | 1.55±0.04 | 0.106255 |
| BdPP2C81 | -1.15166 | 2.22±0.23 | 0.02657 | -1.02163 | 2.03±0.21 | 0.019888 |
| BdPP2C82 | -1.14489 | 2.21±0.26 | 0.028575 | -0.97948 | 1.97±0.23 | 0.027878 |
| BdPP2C83 | -0.20419 | 1.15±0.03 | 0.584612 | -2.29631 | 4.91±1.44 | 0.000144 |
| BdPP2C84 | -0.40936 | 1.33±0.54 | 0.382804 | -0.12038 | 1.09±0.18 | 0.682052 |
| BdPP2C85 | -1.27001 | 2.41±0.69 | 0.005622 | -0.13258 | 1.1±0.04 | 0.646249 |
| BdPP2C86 | -1.22079 | 2.33±0.1 | 0.015152 | 0.196065 | 0.87±0.03 | 0.541881 |
|  | **ABA-3h** |  |  | **ABA-6h** |  |  |
| **BdPP2C** | **average** | **fold-change** | **p-value** | **average** | **fold-change** | **p-value** |
| BdPP2C1 | -1.07161 | 2.1±0.39 | 0.046485 | 0.098953 | 0.93±0.08 | 0.65203 |
| BdPP2C2 | -1.52285 | 2.87±0.11 | 0.005289 | -1.70518 | 3.26±0.78 | 0.039879 |
| BdPP2C3 | -1.15017 | 2.22±0.35 | 0.039939 | -0.80138 | 1.74±0.05 | 0.043985 |
| BdPP2C4 | -1.5692 | 2.97±0.11 | 0.013951 | -1.15974 | 2.23±0.46 | 0.094178 |
| BdPP2C5 | -1.70339 | 3.26±0.21 | 0.018549 | -1.36889 | 2.58±0.43 | 0.041183 |
| BdPP2C6 | -5.18228 | 36.31±8.66 | 0.00017 | -4.37951 | 20.81±5.73 | 0.00011 |
| BdPP2C7 | -1.35094 | 2.55±0.54 | 0.040364 | 0.009372 | 0.99±0.13 | 0.97013 |
| BdPP2C8 | -1.12365 | 2.18±0.15 | 0.028791 | -0.43025 | 1.35±0.06 | 0.354474 |
| BdPP2C9 | -0.90302 | 1.87±0.13 | 0.023321 | -0.84859 | 1.8±0.09 | 0.133781 |
| BdPP2C10 | -0.59928 | 1.51±0.37 | 0.205897 | 0.542497 | 0.69±0.08 | 0.143578 |
| BdPP2C11 | -1.30639 | 2.47±0.21 | 0.00668 | -1.06955 | 2.1±0.4 | 0.034909 |
| BdPP2C12 | -1.60013 | 3.03±0.76 | 0.009562 | -0.6526 | 1.57±0.18 | 0.02318 |
| BdPP2C13 | -6.79887 | 111.34±4.75 | 6.34E-06 | -7.7174 | 210.46±49.74 | 7.16E-06 |
| BdPP2C14 | 0.052146 | 0.96±0.15 | 0.8341 | 0.099206 | 0.93±0.16 | 0.908053 |
| BdPP2C16 | -1.48547 | 2.8±0.49 | 0.004687 | -0.70075 | 1.63±0.35 | 0.033673 |
| BdPP2C19 | -1.55128 | 2.93±1.02 | 0.007715 | -0.3999 | 1.32±0.29 | 0.154022 |
| BdPP2C20 | -1.70329 | 3.26±0.31 | 0.002154 | -1.18343 | 2.27±0.48 | 0.114368 |
| BdPP2C22 | -2.06351 | 4.18±0.84 | 0.003389 | -1.26858 | 2.41±0.31 | 0.011994 |
| BdPP2C23 | -1.12682 | 2.18±0.1 | 0.006563 | -0.61253 | 1.53±0.17 | 0.407625 |
| BdPP2C24 | -0.28821 | 1.22±0.04 | 0.82604 | 0.980976 | 0.51±0.08 | 0.701428 |
| BdPP2C25 | -0.88507 | 1.85±0.04 | 0.049755 | -0.81951 | 1.76±0.27 | 0.009382 |
| BdPP2C26 | 0.282877 | 0.82±0.02 | 0.487678 | -1.37039 | 2.59±0.44 | 0.005601 |
| BdPP2C28 | -3.33735 | 10.11±0.32 | 0.026691 | -1.49479 | 2.82±0.03 | 0.012541 |
| BdPP2C29 | -1.3035 | 2.47±0.18 | 0.002045 | -2.0614 | 4.17±0.02 | 0.081281 |
| BdPP2C30 | -1.64016 | 3.12±0.21 | 0.006336 | -0.8402 | 1.79±0.08 | 0.096366 |
| BdPP2C31 | -1.15754 | 2.23±0.08 | 0.051337 | -0.0383 | 1.03±0.11 | 0.878811 |
| BdPP2C32 | -7.1611 | 143.12±0.46 | 0.028313 | 0.937731 | 0.52±0.09 | 0.779655 |
| BdPP2C33 | -1.79621 | 3.47±0.54 | 0.002293 | 0.107789 | 0.93±0.05 | 0.688152 |
| BdPP2C34 | -0.33163 | 1.26±0.19 | 0.536763 | -0.35637 | 1.28±0.05 | 0.009422 |
| BdPP2C35 | -2.00058 | 4±0.77 | 0.001276 | -1.80898 | 3.5±0.47 | 0.008191 |
| BdPP2C36 | -3.37267 | 10.36±0.06 | 0.001449 | -3.48984 | 11.23±0.36 | 0.015031 |
| BdPP2C37 | -5.25524 | 38.19±1.82 | 0.000366 | -4.36305 | 20.58±0.21 | 2.7E-05 |
| BdPP2C38 | -2.47388 | 5.56±0.77 | 0.02202 | -3.32864 | 10.05±1.52 | 0.005441 |
| BdPP2C39 | -5.01496 | 32.33±1.56 | 0.043597 | -0.90051 | 1.87±0.44 | 0.065827 |
| BdPP2C40 | -2.82683 | 7.1±1.21 | 0.000653 | -1.86209 | 3.64±1.12 | 0.003197 |
| BdPP2C41 | 0.358476 | 0.78±0.04 | 0.507085 | 0.106293 | 0.93±0.04 | 0.763546 |
| BdPP2C42 | -1.3358 | 2.52±0.05 | 0.000565 | -0.74841 | 1.68±0.22 | 0.413461 |
| BdPP2C43 | -1.22337 | 2.33±0.48 | 0.007196 | 0.223237 | 0.86±0.07 | 0.177393 |
| BdPP2C44 | -4.96941 | 31.33±0.54 | 4.22E-06 | -5.26593 | 38.48±10.09 | 0.000623 |
| BdPP2C45 | -1.29499 | 2.45±0.37 | 0.033252 | 0.289794 | 0.82±0.05 | 0.211482 |
| BdPP2C46 | -7.03861 | 131.47±24.41 | 1.17E-05 | -7.13064 | 140.13±7.92 | 8.16E-06 |
| BdPP2C47 | -3.57887 | 11.95±0.27 | 0.000215 | -4.22809 | 18.74±3.45 | 0.047384 |
| BdPP2C48 | -1.43458 | 2.7±0.58 | 0.009038 | -0.21981 | 1.16±0.2 | 0.261885 |
| BdPP2C49 | -1.0502 | 2.07±0.36 | 0.053958 | -0.43892 | 1.36±0.3 | 0.121283 |
| BdPP2C50 | -1.21142 | 2.32±0.52 | 0.016241 | -0.54039 | 1.45±0.07 | 0.057897 |
| BdPP2C51 | -1.00674 | 2.01±0.13 | 5.32E-05 | 0.618462 | 0.65±0.02 | 0.001153 |
| BdPP2C52 | 0.660096 | 0.63±0.05 | 0.208615 | -0.89964 | 1.87±0.08 | 0.120301 |
| BdPP2C53 | -1.30366 | 2.47±0.42 | 0.020022 | 0.27448 | 0.83±0.02 | 0.50433 |
| BdPP2C54 | -1.46398 | 2.76±0.19 | 0.000925 | 0.805525 | 0.57±0.12 | 0.055573 |
| BdPP2C55 | -1.81371 | 3.52±0.9 | 0.006068 | -0.27069 | 1.21±0.42 | 0.509505 |
| BdPP2C57 | -1.98918 | 3.97±0.22 | 8.81E-05 | 0.162863 | 0.89±0.27 | 0.682564 |
| BdPP2C58 | -2.11078 | 4.32±0.58 | 0.001513 | -1.17878 | 2.26±0.26 | 0.001071 |
| BdPP2C59 | -1.06488 | 2.09±0.08 | 0.003531 | -0.11581 | 1.08±0.5 | 0.839057 |
| BdPP2C60 | -1.50872 | 2.85±0.42 | 0.000902 | -0.15775 | 1.12±0.23 | 0.596023 |
| BdPP2C61 | -1.12118 | 2.18±0.3 | 0.004917 | -1.71928 | 3.29±0.86 | 0.039582 |
| BdPP2C62 | -0.81746 | 1.76±0.08 | 0.191937 | 0.043333 | 0.97±0.19 | 0.974523 |
| BdPP2C64 | -1.36627 | 2.58±0.6 | 0.006555 | 0.636272 | 0.64±0.11 | 0.061447 |
| BdPP2C65 | -1.45106 | 2.73±0.63 | 0.000645 | -3.70102 | 13.01±1.45 | 0.010819 |
| BdPP2C66 | -1.75679 | 3.38±0.15 | 0.020334 | -0.51684 | 1.43±0.11 | 0.33797 |
| BdPP2C67 | -1.1367 | 2.2±0.07 | 0.000194 | 0.397086 | 0.76±0.02 | 0.794961 |
| BdPP2C68 | -1.33111 | 2.52±0.2 | 0.009953 | 0.080181 | 0.95±0.01 | 0.095154 |
| BdPP2C69 | -3.07106 | 8.4±1.01 | 0.00203 | -1.41349 | 2.66±0.53 | 0.01575 |
| BdPP2C70 | -4.73179 | 26.57±5.15 | 1.13E-05 | -3.92617 | 15.2±0.62 | 7.93E-07 |
| BdPP2C71 | -1.83179 | 3.56±0.23 | 0.008094 | -0.51161 | 1.43±0.49 | 0.179994 |
| BdPP2C72 | -1.66534 | 3.17±0.64 | 0.000335 | -0.6726 | 1.59±0.37 | 0.131355 |
| BdPP2C73 | -1.73718 | 3.33±0.24 | 0.005785 | -0.74957 | 1.68±0.04 | 0.589499 |
| BdPP2C74 | -1.08277 | 2.12±0.67 | 0.056683 | 0.709526 | 0.61±0.18 | 0.167584 |
| BdPP2C75 | -7.5769 | 190.93±0.34 | 3.12E-05 | -4.8323 | 28.49±0.85 | 0.03349 |
| BdPP2C76 | -1.0717 | 2.1±0.03 | 0.117128 | -1.29242 | 2.45±0.37 | 0.00168 |
| BdPP2C77 | -3.25393 | 9.54±5.8 | 0.006448 | -1.8 | 3.48±0.61 | 0.044166 |
| BdPP2C79 | -1.36033 | 2.57±0.22 | 0.003526 | -1.77628 | 3.43±0.3 | 0.195939 |
| BdPP2C80 | -1.49534 | 2.82±0.76 | 0.011941 | -0.80333 | 1.75±0.17 | 0.004938 |
| BdPP2C81 | -0.98312 | 1.98±0.41 | 0.009893 | -0.02104 | 1.01±0.03 | 0.783522 |
| BdPP2C82 | -1.33656 | 2.53±0.52 | 0.018247 | -0.12835 | 1.09±0.19 | 0.628779 |
| BdPP2C83 | -2.18288 | 4.54±0.05 | 0.000262 | -1.13359 | 2.19±0.13 | 0.104131 |
| BdPP2C84 | -1.4515 | 2.73±0.7 | 0.037366 | 0.417014 | 0.75±0.24 | 0.173078 |
| BdPP2C85 | -2.37706 | 5.19±0.51 | 0.000195 | -1.78431 | 3.44±0.98 | 0.007336 |
| BdPP2C86 | -2.69423 | 6.47±0.1 | 0.009934 | -0.71961 | 1.65±0.02 | 0.003341 |
|  | **H_2_O_2_-3h** |  |  | **H_2_O_2_-6h** |  |  |
| **BdPP2C** | **average** | **fold-change** | **p-value** | **average** | **fold-change** | **p-value** |
| BdPP2C1 | -1.47102 | 2.77±0.06 | 0.007865 | -0.67387 | 1.6±0.11 | 0.023192 |
| BdPP2C2 | -1.42966 | 2.69±0.87 | 0.000447 | 0.359859 | 0.78±0.04 | 0.558966 |
| BdPP2C3 | -1.30769 | 2.48±0.29 | 0.012832 | -0.59698 | 1.51±0.13 | 0.09158 |
| BdPP2C4 | 1.261958 | 0.42±0.02 | 0.001118 | 0.77078 | 0.59±0.03 | 0.332068 |
| BdPP2C5 | -1.44053 | 2.71±0.25 | 0.008517 | -0.30618 | 1.24±0.05 | 0.514654 |
| BdPP2C6 | -3.36158 | 10.28±1.21 | 0.000509 | -1.84012 | 3.58±0.67 | 0.000853 |
| BdPP2C7 | -1.41275 | 2.66±0.32 | 0.01759 | -0.71821 | 1.65±0.18 | 0.028235 |
| BdPP2C8 | -0.15122 | 1.11±0.21 | 0.627824 | -0.03035 | 1.02±0.06 | 0.953053 |
| BdPP2C9 | -0.43615 | 1.35±0.19 | 0.169074 | 0.019474 | 0.99±0.04 | 0.967096 |
| BdPP2C10 | -0.87551 | 1.83±0.41 | 0.063084 | -0.08327 | 1.06±0.09 | 0.768434 |
| BdPP2C11 | -0.13608 | 1.1±0.07 | 0.443313 | -1.85679 | 3.62±0.78 | 0.114006 |
| BdPP2C12 | -0.90187 | 1.87±0.17 | 0.04335 | -0.02523 | 1.02±0.16 | 0.920796 |
| BdPP2C13 | -2.78278 | 6.88±0.09 | 0.001842 | -1.81303 | 3.51±1.4 | 0.007235 |
| BdPP2C14 | -1.25766 | 2.39±0.19 | 0.000176 | 0.31564 | 0.8±0.09 | 0.430341 |
| BdPP2C16 | -1.65826 | 3.16±0.2 | 0.006614 | -0.4625 | 1.38±0.1 | 0.349817 |
| BdPP2C19 | -0.22892 | 1.17±0.44 | 0.605055 | 2.043044 | 0.24±0.03 | 0.07685 |
| BdPP2C20 | -0.15146 | 1.11±0.16 | 0.692466 | -0.03794 | 1.03±0.07 | 0.953059 |
| BdPP2C22 | 0.81831 | 0.57±0.02 | 0.049321 | -0.19347 | 1.14±0.59 | 0.546333 |
| BdPP2C23 | 0.234947 | 0.85±0.09 | 0.575909 | 1.055773 | 0.48±0.05 | 0.228993 |
| BdPP2C24 | -3.98148 | 15.8±0.59 | 0.003732 | 3.146168 | 0.11±0.02 | 0.038195 |
| BdPP2C25 | -2.45916 | 5.5±0.4 | 0.003038 | -1.26695 | 2.41±0.3 | 0.001442 |
| BdPP2C26 | -1.42812 | 2.69±0.65 | 0.024196 | -2.24583 | 4.74±0.22 | 0.001264 |
| BdPP2C28 | -0.29676 | 1.23±0.26 | 0.77227 | 1.268814 | 0.42±0.01 | 0.099972 |
| BdPP2C29 | 0.603732 | 0.66±0.07 | 0.446393 | 0.66487 | 0.63±0.06 | 0.497698 |
| BdPP2C30 | -1.22331 | 2.33±0.16 | 0.011898 | -0.88908 | 1.85±0.02 | 0.125387 |
| BdPP2C31 | -1.69376 | 3.23±0.9 | 0.012331 | -0.72751 | 1.66±0.05 | 0.110112 |
| BdPP2C32 | -4.59746 | 24.21±0.09 | 0.078896 | -3.85955 | 14.52±0.15 | 0.174495 |
| BdPP2C33 | -1.49927 | 2.83±1.66 | 0.001892 | -0.42309 | 1.34±0.24 | 0.346211 |
| BdPP2C34 | 0.115529 | 0.92±0.34 | 0.21701 | 1.433442 | 0.37±0.05 | 0.000515 |
| BdPP2C35 | -0.58251 | 1.5±0.31 | 0.086226 | -0.34688 | 1.27±0.31 | 0.248329 |
| BdPP2C36 | -2.4937 | 5.63±0.06 | 0.028185 | -3.03088 | 8.17±0.68 | 0.024145 |
| BdPP2C37 | -3.35151 | 10.21±2.67 | 0.001302 | -1.29753 | 2.46±0 | 0.003867 |
| BdPP2C38 | -1.90005 | 3.73±1.74 | 0.112512 | -0.66412 | 1.58±0.1 | 0.371889 |
| BdPP2C39 | -4.64688 | 25.05±2.98 | 0.053602 | -3.06875 | 8.39±0.38 | 8.63E-06 |
| BdPP2C40 | -2.20699 | 4.62±0.6 | 0.002102 | -0.66706 | 1.59±0.56 | 0.109036 |
| BdPP2C41 | -0.44877 | 1.36±0.02 | 0.817901 | 0.708242 | 0.61±0.18 | 0.184587 |
| BdPP2C42 | 2.050529 | 0.24±0.02 | 3.38E-05 | 2.030223 | 0.24±0.06 | 0.00275 |
| BdPP2C43 | -0.51689 | 1.43±0.13 | 0.083912 | -0.5134 | 1.43±0.15 | 0.04324 |
| BdPP2C44 | -0.8676 | 1.82±0.26 | 0.026709 | -0.85622 | 1.81±0.07 | 0.190356 |
| BdPP2C45 | -2.02045 | 4.06±0.73 | 0.006564 | -0.78898 | 1.73±0.1 | 0.061926 |
| BdPP2C46 | -2.46004 | 5.5±0.94 | 0.014743 | -1.44646 | 2.73±0.49 | 0.015124 |
| BdPP2C47 | -2.7773 | 6.86±0.34 | 0.000363 | -4.59322 | 24.14±0.01 | 0.039495 |
| BdPP2C48 | -1.12094 | 2.17±0.78 | 0.013144 | 0.568721 | 0.67±0.07 | 0.047017 |
| BdPP2C49 | -0.51374 | 1.43±0.15 | 0.214474 | -0.13067 | 1.09±0.34 | 0.689331 |
| BdPP2C50 | -1.50258 | 2.83±0.28 | 0.009131 | -0.38487 | 1.31±0.13 | 0.343215 |
| BdPP2C51 | 1.160719 | 0.45±0.03 | 0.02429 | 0.340263 | 0.79±0.01 | 0.007516 |
| BdPP2C52 | -0.39702 | 1.32±0.06 | 0.498083 | -0.14988 | 1.11±0.09 | 0.91275 |
| BdPP2C53 | -0.49073 | 1.41±0.09 | 0.141813 | 0.183237 | 0.88±0.12 | 0.562024 |
| BdPP2C54 | -0.49357 | 1.41±0.07 | 0.263348 | -0.19238 | 1.14±0.05 | 0.626092 |
| BdPP2C55 | -2.45606 | 5.49±1.52 | 0.00125 | -0.70727 | 1.63±0.36 | 0.161551 |
| BdPP2C57 | -1.36778 | 2.58±0.94 | 0.004645 | -0.57849 | 1.49±0.47 | 0.19475 |
| BdPP2C58 | -1.23923 | 2.36±0.41 | 0.01921 | -0.19383 | 1.14±0.17 | 0.322244 |
| BdPP2C59 | 0.539452 | 0.69±0.04 | 0.029708 | 0.920059 | 0.53±0.03 | 0.183265 |
| BdPP2C60 | -0.13043 | 1.09±0.08 | 0.557356 | 0.401247 | 0.76±0.01 | 0.217383 |
| BdPP2C61 | -0.00017 | 1±0.002 | 0.99955 | -0.12814 | 1.09±0.07 | 0.597404 |
| BdPP2C62 | -0.60521 | 1.52±0.11 | 0.159434 | 0.131882 | 0.91±0.06 | 0.591653 |
| BdPP2C64 | -1.43206 | 2.7±0.19 | 0.003674 | 0.327655 | 0.8±0.19 | 0.225386 |
| BdPP2C65 | -0.1637 | 1.12±0.19 | 0.809846 | -2.62862 | 6.18±1.13 | 0.030969 |
| BdPP2C66 | -1.98992 | 3.97±0.07 | 0.013406 | 0.557314 | 0.68±0.05 | 0.37428 |
| BdPP2C67 | -0.01242 | 1.01±0.15 | 0.970118 | 1.052483 | 0.48±0.05 | 0.002028 |
| BdPP2C68 | -0.65278 | 1.57±0.06 | 0.119474 | 0.046525 | 0.97±0.28 | 0.90206 |
| BdPP2C69 | -1.58261 | 3±0.28 | 0.01701 | 0.669759 | 0.63±0.26 | 0.110894 |
| BdPP2C70 | -2.86415 | 7.28±0.08 | 0.001798 | -1.93624 | 3.83±0.84 | 0.019602 |
| BdPP2C71 | 1.477158 | 0.36±0.08 | 0.067631 | 1.012195 | 0.5±0.06 | 0.01067 |
| BdPP2C72 | 0.86431 | 0.55±0.07 | 0.332984 | -1.24305 | 2.37±0.23 | 0.081903 |
| BdPP2C73 | 1.58466 | 0.33±0.09 | 0.002301 | 1.578401 | 0.33±0.01 | 0.006835 |
| BdPP2C74 | -0.44288 | 1.36±0.05 | 0.38466 | -0.21166 | 1.16±0.09 | 0.691333 |
| BdPP2C75 | -3.45146 | 10.94±0.55 | 0.000791 | 3.197084 | 0.11±0.02 | 0.027648 |
| BdPP2C76 | -1.06085 | 2.09±0.01 | 0.183701 | -0.86565 | 1.82±0.07 | 0.074828 |
| BdPP2C77 | -2.15982 | 4.47±0.46 | 0.024798 | -0.7126 | 1.64±0.05 | 0.231549 |
| BdPP2C79 | 0.420139 | 0.75±0.04 | 0.579894 | 0.296715 | 0.81±0.24 | 0.830186 |
| BdPP2C80 | -0.46143 | 1.38±0.11 | 0.428256 | -0.15345 | 1.11±0.05 | 0.661593 |
| BdPP2C81 | -0.68931 | 1.61±0.13 | 0.306018 | -0.15451 | 1.11±0.2 | 0.734563 |
| BdPP2C82 | -0.84373 | 1.79±0.18 | 0.059035 | -0.76797 | 1.7±0.05 | 0.044216 |
| BdPP2C83 | 0.646311 | 0.64±0.09 | 0.389669 | 0.258331 | 0.84±0.28 | 0.663101 |
| BdPP2C84 | -0.68921 | 1.61±0.05 | 0.255601 | -0.45953 | 1.38±0.17 | 0.364503 |
| BdPP2C85 | -1.0118 | 2.02±0.03 | 0.116891 | -1.08343 | 2.12±0.03 | 0.072506 |
| BdPP2C86 | -0.40034 | 1.32±0.14 | 0.32657 | -0.37841 | 1.3±0.13 | 0.1251 |
|  | **JA-3h** |  |  | **JA-6h** |  |  |
| **BdPP2C** | **average** | **fold-change** | **p-value** | **average** | **fold-change** | **p-value** |
| BdPP2C1 | -0.84871 | 1.8±0.14 | 0.257989 | 1.195264 | 0.44±0.06 | 0.000261 |
| BdPP2C2 | -2.09931 | 4.29±1.04 | 0.01036 | -0.59546 | 1.51±0.1 | 0.358171 |
| BdPP2C3 | -0.93982 | 1.92±0.42 | 0.161387 | 0.255183 | 0.84±0.04 | 0.39197 |
| BdPP2C4 | -1.20678 | 2.31±0.88 | 0.011238 | -0.27599 | 1.21±0.08 | 0.61725 |
| BdPP2C5 | -1.18687 | 2.28±0.78 | 0.065014 | 0.234088 | 0.85±0.05 | 0.647856 |
| BdPP2C6 | -3.65018 | 12.55±2.08 | 0.003507 | -0.6938 | 1.62±0.37 | 0.066525 |
| BdPP2C7 | -1.24117 | 2.36±0.46 | 0.112348 | 0.93436 | 0.52±0.07 | 0.034507 |
| BdPP2C8 | -2.04224 | 4.12±1.79 | 0.021936 | 0.264425 | 0.83±0.38 | 0.550987 |
| BdPP2C9 | -0.59828 | 1.51±0.77 | 0.412247 | 0.318654 | 0.8±0.04 | 0.531252 |
| BdPP2C10 | -1.6302 | 3.1±1.36 | 0.13491 | 0.863021 | 0.55±0.05 | 0.014683 |
| BdPP2C11 | -0.74444 | 1.68±0.64 | 0.251293 | 0.085544 | 0.94±0.03 | 0.811678 |
| BdPP2C12 | -1.12101 | 2.17±0.07 | 0.244873 | 1.059188 | 0.48±0.02 | 0.00066 |
| BdPP2C13 | -3.15903 | 8.93±3.29 | 0.005793 | -3.27312 | 0.29±0.01 | 0.000247 |
| BdPP2C14 | -0.68495 | 1.61±0.15 | 0.051792 | 1.776919 | 0.93±0.25 | 0.006873 |
| BdPP2C16 | -1.63948 | 3.12±1.78 | 0.065215 | 0.110054 | 0.31±0.08 | 0.654729 |
| BdPP2C19 | -0.57718 | 1.49±0.45 | 0.525003 | 1.680122 | 1.57±0.06 | 0.004637 |
| BdPP2C20 | -2.54868 | 5.85±0.04 | 0.011859 | -0.65346 | 1.31±0.14 | 0.313781 |
| BdPP2C22 | 0.138383 | 0.91±0.14 | 0.698078 | -0.38606 | 0.98±0.04 | 0.3517 |
| BdPP2C23 | -1.2909 | 2.45±0.42 | 0.040907 | 0.024837 | 1.11±0.18 | 0.971499 |
| BdPP2C24 | 0.09143 | 0.94±0.27 | 0.86457 | -0.14772 | 0.85±0.25 | 0.895415 |
| BdPP2C25 | -1.70337 | 3.26±0.09 | 0.062295 | 0.232167 | 0.4±0.04 | 0.440643 |
| BdPP2C26 | 1.154005 | 0.45±0.02 | 0.012322 | 1.312632 | 0.12±0.05 | 0.023847 |
| BdPP2C28 | 0.620438 | 0.65±0.06 | 0.558418 | 3.063335 | 2.03±0.41 | 0.000996 |
| BdPP2C29 | -1.26231 | 2.4±0.42 | 0.01386 | -1.02393 | 2.54±0.06 | 0.319879 |
| BdPP2C30 | -2.84149 | 7.17±3.06 | 0.007413 | -1.34337 | 0.36±0.02 | 0.027833 |
| BdPP2C31 | -1.04572 | 2.06±0.61 | 0.322536 | 1.484986 | 99.92±0.08 | 0.002054 |
| BdPP2C32 | -5.67598 | 51.13±0.86 | 0.042923 | -6.64275 | 0.5±0.13 | 0.048133 |
| BdPP2C33 | -0.63258 | 1.55±0.21 | 0.46914 | 1.009322 | 0.48±0.05 | 0.001435 |
| BdPP2C34 | -0.03306 | 1.02±0.06 | 0.191521 | 1.045377 | 1.74±0.06 | 3.79E-05 |
| BdPP2C35 | -1.99867 | 4±1.06 | 0.031257 | -0.80165 | 1.48±0.17 | 0.026638 |
| BdPP2C36 | 0.162062 | 0.89±0.17 | 0.761191 | -0.56764 | 0.83±0.04 | 0.543637 |
| BdPP2C37 | -1.67791 | 3.2±0.94 | 0.13608 | 0.270838 | 1.27±0.07 | 0.108027 |
| BdPP2C38 | -0.55397 | 1.47±0.01 | 0.671519 | -0.3412 | 0.32±0.03 | 0.738432 |
| BdPP2C39 | -6.38729 | 83.71±0.09 | 0.028279 | 1.626695 | 1±0.01 | 0.004346 |
| BdPP2C40 | -1.83037 | 3.56±0.75 | 0.074882 | -0.00049 | 0.02±0.01 | 0.99856 |
| BdPP2C41 | 0.791767 | 0.58±0.04 | 0.221781 | 5.924688 | 1.27±0.09 | 0.011821 |
| BdPP2C42 | 2.121469 | 0.23±0.01 | 0.000744 | -0.34737 | 0.33±0.01 | 0.327199 |
| BdPP2C43 | -0.56144 | 1.48±0.01 | 0.482253 | 1.605695 | 1.37±0.49 | 6.85E-05 |
| BdPP2C44 | -1.12551 | 2.18±0.14 | 0.033724 | -0.45265 | 0.42±0.01 | 0.448534 |
| BdPP2C45 | -1.17834 | 2.26±0.49 | 0.239671 | 1.267036 | 2.75±0.73 | 0.001083 |
| BdPP2C46 | -0.80954 | 1.75±0.65 | 0.406858 | -1.46165 | 1.37±0.14 | 0.006005 |
| BdPP2C47 | -0.1422 | 1.1±0.15 | 0.612239 | -0.45491 | 0.65±0.03 | 0.775967 |
| BdPP2C48 | -1.40301 | 2.64±0.23 | 0.150467 | 0.629622 | 0.87±0.01 | 0.019528 |
| BdPP2C49 | -1.1502 | 2.22±0.29 | 0.13239 | 0.19694 | 0.61±0.1 | 0.269455 |
| BdPP2C50 | -0.71952 | 1.65±0.21 | 0.3744 | 0.719392 | 0.6±0.08 | 0.034135 |
| BdPP2C51 | -0.67982 | 1.6±0.15 | 0.239606 | 0.742758 | 1.39±0.38 | 0.000324 |
| BdPP2C52 | 0.312095 | 0.81±0.004 | 0.865698 | -0.47152 | 0.46±0.23 | 0.525319 |
| BdPP2C53 | -0.3563 | 1.28±0.09 | 0.674793 | 1.124824 | 0.42±0.03 | 0.010373 |
| BdPP2C54 | -0.0761 | 1.05±0.26 | 0.815201 | 1.259584 | 0.64±0.05 | 0.015482 |
| BdPP2C55 | -1.9068 | 3.75±0.88 | 0.092446 | 0.649077 | 1.52±0.24 | 0.158478 |
| BdPP2C57 | -2.49169 | 5.62±0.09 | 0.001699 | -0.60205 | 1.08±0.16 | 0.183717 |
| BdPP2C58 | -1.60918 | 3.05±1.11 | 0.035244 | -0.11507 | 0.86±0.02 | 0.256299 |
| BdPP2C59 | -0.73113 | 1.66±0.5 | 0.067095 | 0.225073 | 0.9±0.002 | 0.70378 |
| BdPP2C60 | -1.44253 | 2.72±0.68 | 0.036967 | 0.15511 | 1.56±0.05 | 0.623542 |
| BdPP2C61 | -0.44842 | 1.36±0.06 | 0.125132 | -0.64317 | 1.11±0.05 | 0.070733 |
| BdPP2C62 | -1.30121 | 2.46±0.67 | 0.021957 | -0.14582 | 0.36±0.02 | 0.496322 |
| BdPP2C64 | -0.93801 | 1.92±0.33 | 0.340335 | 1.464814 | 8.33±0 | 0.003745 |
| BdPP2C65 | -0.08283 | 1.06±0.12 | 0.375589 | -3.05849 | 0.84±0.08 | 0.025944 |
| BdPP2C66 | 0.530231 | 0.69±0.1 | 0.510037 | 0.255974 | 0.41±0.02 | 0.591215 |
| BdPP2C67 | -0.21764 | 1.16±0.12 | 0.422326 | 1.28429 | 0.56±0.09 | 5.71E-06 |
| BdPP2C68 | -1.05126 | 2.07±1.03 | 0.167101 | 0.834092 | 0.61±0.04 | 1.76E-05 |
| BdPP2C69 | -2.8243 | 7.08±0.14 | 0.040276 | 0.705876 | 0.25±0.02 | 0.092754 |
| BdPP2C70 | 0.319536 | 0.8±0.25 | 0.758021 | 1.998161 | 1.47±0.27 | 0.006588 |
| BdPP2C71 | -1.37611 | 2.6±0.33 | 0.02247 | -0.55206 | 0.72±0.01 | 0.087279 |
| BdPP2C72 | -0.27056 | 1.21±0.2 | 0.540103 | 0.467337 | 0.31±0.01 | 0.298356 |
| BdPP2C73 | 0.061723 | 0.96±0.14 | 0.802244 | 1.695147 | 0.2±0.01 | 0.005419 |
| BdPP2C74 | -0.30347 | 1.23±0.02 | 0.792308 | 2.306758 | 2.52±0.11 | 0.004564 |
| BdPP2C75 | -0.57008 | 1.48±0.14 | 0.707779 | -1.33155 | 0.52±0.03 | 0.000779 |
| BdPP2C76 | -0.80145 | 1.74±0.31 | 0.317628 | 0.94307 | 0.35±0.03 | 0.008513 |
| BdPP2C77 | -0.67494 | 1.6±0.23 | 0.502381 | 1.505888 | 1.25±0.16 | 0.044744 |
| BdPP2C79 | -0.07017 | 1.05±0.07 | 0.887722 | -0.32527 | 0.96±0.05 | 0.780993 |
| BdPP2C80 | -0.7392 | 1.67±0.36 | 0.37787 | 0.054085 | 0.28±0.08 | 0.79782 |
| BdPP2C81 | -0.17793 | 1.13±0.09 | 0.832066 | 1.829288 | 0.6±0.03 | 0.000183 |
| BdPP2C82 | -1.34882 | 2.55±0.69 | 0.112775 | 0.737512 | 0.29±0.01 | 0.033017 |
| BdPP2C83 | -1.88514 | 3.69±0.44 | 0.007274 | -1.04616 | 2.07±0.52 | 0.137384 |
| BdPP2C84 | -0.48458 | 1.4±0.01 | 0.632387 | 2.883744 | 0.14±0.09 | 0.000365 |
| BdPP2C85 | -1.93166 | 3.81±0.46 | 0.002654 | -0.60074 | 1.52±0.24 | 0.167859 |
| BdPP2C86 | -1.12413 | 2.18±0.4 | 0.191208 | 0.330467 | 0.8±0.02 | 0.04737 |
|  | **SA-3h** |  |  | **SA-6h** |  |  |
| **BdPP2C** | **average** | **fold-change** | **p-value** | **average** | **fold-change** | **p-value** |
| BdPP2C1 | -1.52045 | 2.87±0.18 | 0.020931 | -0.61746 | 1.53±0.28 | 0.062903 |
| BdPP2C2 | -1.56754 | 2.96±0.95 | 0.002125 | 0.001638 | 1±0.05 | 0.997818 |
| BdPP2C3 | -1.59946 | 3.03±0.5 | 0.007132 | -0.99163 | 1.99±0.45 | 0.005401 |
| BdPP2C4 | -0.7754 | 1.71±0.32 | 0.05002 | 0.64513 | 0.64±0.05 | 0.323328 |
| BdPP2C5 | -2.22203 | 4.67±1.02 | 0.002285 | -1.44843 | 2.73±0.97 | 0.015623 |
| BdPP2C6 | -3.39912 | 10.55±1.84 | 0.000934 | -2.4036 | 5.29±1.73 | 0.000991 |
| BdPP2C7 | -0.99354 | 1.99±0.24 | 0.080954 | -0.30919 | 1.24±0.29 | 0.333555 |
| BdPP2C8 | -1.4817 | 2.79±0.32 | 0.007749 | -0.0718 | 1.05±0.04 | 0.858948 |
| BdPP2C9 | -0.55988 | 1.47±0.38 | 0.056393 | 0.540659 | 0.69±0.03 | 0.275097 |
| BdPP2C10 | -1.53483 | 2.9±0.72 | 0.01419 | -1.02619 | 2.04±0.45 | 0.041615 |
| BdPP2C11 | -0.64345 | 1.56±0.22 | 0.085715 | -0.17876 | 1.13±0.35 | 0.594633 |
| BdPP2C12 | -1.57427 | 2.98±0.16 | 0.016229 | -1.20654 | 2.31±0.62 | 0.013854 |
| BdPP2C13 | -3.24858 | 9.5±0.48 | 0.001 | -4.7567 | 27.03±5.2 | 3.82E-05 |
| BdPP2C14 | -0.1201 | 1.09±0.09 | 0.714519 | 0.601092 | 0.66±0.11 | 0.143679 |
| BdPP2C16 | -0.1899 | 1.14±0.1 | 0.624135 | -0.41161 | 1.33±0.3 | 0.082105 |
| BdPP2C19 | -0.42216 | 1.34±0.54 | 0.376766 | 0.424463 | 0.75±0.02 | 0.124714 |
| BdPP2C20 | -2.2235 | 4.67±0.83 | 0.001898 | -0.61032 | 1.53±0.47 | 0.37113 |
| BdPP2C22 | 0.524085 | 0.7±0.02 | 0.157952 | 0.039647 | 0.97±0.09 | 0.898536 |
| BdPP2C23 | -0.15969 | 1.12±0.09 | 0.655381 | 1.770333 | 0.29±0.02 | 0.050986 |
| BdPP2C24 | -0.052 | 1.04±0.02 | 0.914424 | -0.27063 | 1.21±0.06 | 0.858415 |
| BdPP2C25 | -2.02738 | 4.08±0.7 | 0.009257 | -1.28833 | 2.44±0.39 | 0.001088 |
| BdPP2C26 | 1.971331 | 0.26±0.02 | 8.15E-05 | 0.316743 | 0.8±0.02 | 0.445007 |
| BdPP2C28 | 0.944941 | 0.52±0.05 | 0.373053 | 2.503103 | 0.18±0.02 | 0.001652 |
| BdPP2C29 | -1.82036 | 3.53±0.72 | 0.010096 | -1.46297 | 2.76±0.14 | 0.176352 |
| BdPP2C30 | -1.03127 | 2.04±0.28 | 0.032733 | -0.61851 | 1.54±0.7 | 0.173496 |
| BdPP2C31 | -0.3781 | 1.3±0.15 | 0.450824 | 0.721524 | 0.61±0.04 | 0.094862 |
| BdPP2C32 | -6.38431 | 83.54±0.08 | 0.030148 | -2.70405 | 6.52±0.16 | 0.385143 |
| BdPP2C33 | -0.96052 | 1.95±0.12 | 0.085563 | -0.25419 | 1.19±0.18 | 0.440681 |
| BdPP2C34 | 0.324042 | 0.8±0.05 | 0.256061 | 1.678971 | 0.31±0.03 | 0.000226 |
| BdPP2C35 | -2.04786 | 4.13±0.53 | 0.0027 | -1.20228 | 2.3±0.53 | 0.0067 |
| BdPP2C36 | -0.55991 | 1.47±0.03 | 0.316218 | 0.192443 | 0.88±0.03 | 0.878983 |
| BdPP2C37 | -2.10213 | 4.29±0.41 | 0.008383 | -1.82083 | 3.53±0.57 | 0.004679 |
| BdPP2C38 | 0.230492 | 0.85±0.04 | 0.741917 | -0.69821 | 1.62±0.09 | 0.260367 |
| BdPP2C39 | 0.441481 | 0.74±0.07 | 0.824771 | 0.475117 | 0.72±0.04 | 0.282621 |
| BdPP2C40 | -1.92307 | 3.79±0.25 | 0.003483 | -1.53403 | 2.9±0.92 | 0.004587 |
| BdPP2C41 | -0.94039 | 1.92±0.19 | 0.13915 | 0.774439 | 0.58±0.02 | 0.066216 |
| BdPP2C42 | -0.1349 | 1.1±0.1 | 0.608739 | 2.221629 | 0.21±0.04 | 0.002263 |
| BdPP2C43 | 0.445715 | 0.73±0.07 | 0.30122 | 1.017677 | 0.49±0.12 | 0.015074 |
| BdPP2C44 | -0.10158 | 1.07±0.05 | 0.631233 | -0.87336 | 1.83±0.18 | 0.247528 |
| BdPP2C45 | -0.07657 | 1.05±0.07 | 0.862505 | 0.346098 | 0.79±0.1 | 0.339267 |
| BdPP2C46 | -1.01315 | 2.02±0.001 | 0.094073 | -0.27944 | 1.21±0.21 | 0.17505 |
| BdPP2C47 | 0.106043 | 0.93±0.08 | 0.686507 | -0.16655 | 1.12±0.07 | 0.93465 |
| BdPP2C48 | -2.38705 | 5.23±0.7 | 0.004985 | 0.004637 | 1±0.01 | 0.985245 |
| BdPP2C49 | -2.2508 | 4.76±0.06 | 0.016695 | -0.6702 | 1.59±0.52 | 0.059315 |
| BdPP2C50 | -2.26472 | 4.81±1.24 | 0.003228 | -0.91951 | 1.89±0.18 | 0.003261 |
| BdPP2C51 | -2.44162 | 5.43±1.51 | 0.003304 | -0.3567 | 1.28±0.12 | 0.008769 |
| BdPP2C52 | -3.03666 | 8.21±1.47 | 0.005992 | -0.13586 | 1.1±0.33 | 0.786419 |
| BdPP2C53 | -1.42462 | 2.68±0.16 | 0.044827 | 1.210259 | 0.43±0.01 | 0.018905 |
| BdPP2C54 | -1.20781 | 2.31±0.87 | 0.043812 | 0.553067 | 0.68±0.03 | 0.217288 |
| BdPP2C55 | -4.05347 | 16.6±2.56 | 0.001053 | -2.27942 | 4.85±0.25 | 0.008977 |
| BdPP2C57 | -2.2206 | 4.66±1.12 | 0.006526 | -0.52771 | 1.44±0.78 | 0.276334 |
| BdPP2C58 | -2.54643 | 5.84±0.14 | 0.00376 | -1.01945 | 2.03±0.4 | 0.003067 |
| BdPP2C59 | -1.71865 | 3.29±0.65 | 0.006858 | 0.804169 | 0.57±0.03 | 0.209479 |
| BdPP2C60 | -1.89736 | 3.73±0.1 | 0.004584 | 0.374856 | 0.77±0.04 | 0.358344 |
| BdPP2C61 | -3.26375 | 9.6±0.09 | 1.24E-05 | -2.28624 | 4.88±2.43 | 3.62E-06 |
| BdPP2C62 | -3.33381 | 10.08±0.01 | 0.001298 | -2.23901 | 4.72±1.84 | 0.000599 |
| BdPP2C64 | -2.45451 | 5.48±0.77 | 0.003323 | 0.189482 | 0.88±0.08 | 0.497693 |
| BdPP2C65 | 0.164577 | 0.89±0.09 | 0.683912 | -0.71749 | 1.64±0.12 | 0.432989 |
| BdPP2C66 | 0.239601 | 0.85±0.06 | 0.621863 | -0.31279 | 1.24±0.15 | 0.526676 |
| BdPP2C67 | 0.734395 | 0.6±0.06 | 0.079492 | 1.29915 | 0.41±0.06 | 1.84E-06 |
| BdPP2C68 | -2.12923 | 4.37±0.34 | 0.003687 | 0.143456 | 0.91±0.08 | 0.107395 |
| BdPP2C69 | -3.35449 | 10.23±2.49 | 0.00405 | -1.39557 | 2.63±0.22 | 0.021867 |
| BdPP2C70 | -4.0986 | 17.13±0.43 | 0.000357 | -3.24226 | 9.46±1.56 | 2.13E-05 |
| BdPP2C71 | -2.41873 | 5.35±0.22 | 0.008672 | 0.66106 | 0.63±0.02 | 0.069472 |
| BdPP2C72 | -1.8034 | 3.49±0.58 | 0.001577 | 0.704154 | 0.61±0.06 | 0.306553 |
| BdPP2C73 | -0.18765 | 1.14±0.04 | 0.460063 | 1.59536 | 0.33±0.05 | 0.004943 |
| BdPP2C74 | -1.86664 | 3.65±1.53 | 0.013121 | 0.519976 | 0.7±0.02 | 0.2658 |
| BdPP2C75 | 0.213004 | 0.86±0.24 | 0.714976 | 0.411001 | 0.75±0.06 | 0.079274 |
| BdPP2C76 | -2.48437 | 5.6±2.83 | 0.001839 | -0.60144 | 1.52±0.28 | 0.027241 |
| BdPP2C77 | -2.6518 | 6.28±3.58 | 0.020126 | -0.16151 | 1.12±0.19 | 0.796463 |
| BdPP2C79 | -1.53451 | 2.9±0.52 | 0.007927 | -4.33197 | 20.14±1.13 | 0.234626 |
| BdPP2C80 | -3.30636 | 9.89±2.3 | 0.000474 | -1.02288 | 2.03±0.36 | 0.002745 |
| BdPP2C81 | -2.57862 | 5.97±1.06 | 0.000166 | 0.190282 | 0.88±0.13 | 0.482353 |
| BdPP2C82 | -3.69957 | 12.99±5.71 | 0.00029 | -1.4476 | 2.73±0.32 | 0.004134 |
| BdPP2C83 | -2.61842 | 6.14±0.1 | 0.000184 | -0.35401 | 1.28±0.08 | 0.5537 |
| BdPP2C84 | 0.034997 | 0.98±0.42 | 0.943913 | 2.069618 | 0.24±0.01 | 0.002244 |
| BdPP2C85 | -4.0039 | 16.04±3.88 | 1.18E-05 | -2.03243 | 4.09±0.55 | 0.00767 |
| BdPP2C86 | -2.96057 | 7.78±2.93 | 0.000112 | -0.42858 | 1.35±0.09 | 0.130276 |
|  | **CdCl_2_-3h** |  |  | **CdCl_2_-6h** |  |  |
| **BdPP2C** | **average** | **fold-change** | **p-value** | **average** | **fold-change** | **p-value** |
| BdPP2C1 | 0.442301 | 0.74±0.09 | 0.185897 | 0.35392 | 0.78±0.12 | 0.520797 |
| BdPP2C2 | -1.83322 | 3.56±0.63 | 0.0322 | -0.44615 | 1.36±0.53 | 0.331836 |
| BdPP2C3 | -0.39219 | 1.31±0.08 | 0.084333 | 0.331378 | 0.79±0.05 | 0.548893 |
| BdPP2C4 | -0.53524 | 1.45±0.25 | 0.334673 | 0.199935 | 0.87±0.37 | 0.6987 |
| BdPP2C5 | -0.51068 | 1.42±0.16 | 0.278676 | -0.76891 | 1.7±0.43 | 0.200887 |
| BdPP2C6 | 0.122931 | 0.92±0.19 | 0.646519 | -0.8856 | 1.85±0.13 | 0.207347 |
| BdPP2C7 | 0.253345 | 0.84±0.1 | 0.408971 | 0.085788 | 0.94±0.14 | 0.874675 |
| BdPP2C8 | -0.25243 | 1.19±0.07 | 0.538467 | 0.558323 | 0.68±0.06 | 0.198423 |
| BdPP2C9 | -0.70623 | 1.63±0.43 | 0.176726 | 0.260676 | 0.83±0.07 | 0.343138 |
| BdPP2C10 | 0.105504 | 0.93±0.09 | 0.751749 | 0.748112 | 0.6±0.03 | 0.150954 |
| BdPP2C11 | -0.62738 | 1.54±0.01 | 0.111196 | -0.16358 | 1.12±0.19 | 0.828081 |
| BdPP2C12 | 0.777653 | 0.58±0.09 | 6.18E-07 | 0.280641 | 0.82±0.01 | 0.542572 |
| BdPP2C13 | -2.66018 | 6.32±0.49 | 0.000768 | -1.4521 | 2.74±0.49 | 0.016622 |
| BdPP2C14 | -0.14348 | 1.1±0.15 | 0.720624 | 0.244126 | 0.84±0.17 | 0.104131 |
| BdPP2C16 | 0.046968 | 0.97±0.08 | 0.89547 | 0.543853 | 0.69±0.03 | 0.23072 |
| BdPP2C19 | 0.246454 | 0.84±0.07 | 0.290246 | 0.643339 | 0.64±0.02 | 0.185193 |
| BdPP2C20 | -0.7594 | 1.69±0.24 | 0.277565 | 0.547208 | 0.68±0.09 | 0.185475 |
| BdPP2C22 | 0.132506 | 0.91±0.09 | 0.71003 | 0.24148 | 0.85±0.08 | 0.502486 |
| BdPP2C23 | -0.98661 | 1.98±0.39 | 0.199398 | 0.493617 | 0.71±0.06 | 0.296191 |
| BdPP2C24 | 0.058271 | 0.96±0.07 | 0.820138 | 0.588865 | 0.66±0.15 | 0.736689 |
| BdPP2C25 | -1.77294 | 3.42±0.36 | 0.000318 | -2.003 | 4.01±1.59 | 0.005292 |
| BdPP2C26 | -0.52994 | 1.44±0.07 | 0.236198 | 2.080617 | 0.24±0.04 | 0.026986 |
| BdPP2C28 | 0.096173 | 0.94±0.13 | 0.893676 | 1.151776 | 0.45±0.01 | 0.000637 |
| BdPP2C29 | -2.65959 | 6.32±0.09 | 0.041654 | 1.047307 | 0.48±0.02 | 0.081119 |
| BdPP2C30 | -0.91903 | 1.89±0.09 | 0.058444 | -0.02024 | 1.01±0.9 | 0.964177 |
| BdPP2C31 | 0.999934 | 0.5±0.13 | 0.008706 | 0.586142 | 0.67±0.08 | 0.209616 |
| BdPP2C32 | -4.114608 | 16.93±2.08 | 5.43E-07 | -5.5261 | 46.08±0.22 | 0.019252 |
| BdPP2C33 | 0.898205 | 0.54±0.07 | 0.002209 | 0.550054 | 0.68±0.06 | 0.073481 |
| BdPP2C34 | -0.30342 | 1.23±0.13 | 0.094324 | 1.254467 | 0.42±0.04 | 1.62E-05 |
| BdPP2C35 | -0.3276 | 1.25±0.04 | 0.273414 | 0.810305 | 0.57±0.02 | 0.055346 |
| BdPP2C36 | -0.63624 | 1.55±0.17 | 0.516089 | 0.424367 | 0.75±0.08 | 0.60421 |
| BdPP2C37 | 0.329252 | 0.8±0.19 | 0.319968 | -1.11663 | 2.17±0.18 | 0.056088 |
| BdPP2C38 | -2.12331 | 4.36±0.12 | 0.02573 | -0.40921 | 1.33±0.03 | 0.835862 |
| BdPP2C39 | 4.489279 | 0.04±0.01 | 1.52E-07 | -2.75728 | 6.76±0.46 | 0.000156 |
| BdPP2C40 | 0.287523 | 0.82±0.07 | 0.317422 | -0.74782 | 1.68±0.06 | 0.107848 |
| BdPP2C41 | 0.690343 | 0.62±0.07 | 0.238437 | -0.24145 | 1.18±0.31 | 0.642598 |
| BdPP2C42 | 0.365742 | 0.78±0.07 | 0.323844 | -0.02704 | 1.02±0.05 | 0.909134 |
| BdPP2C43 | 0.880915 | 0.54±0.07 | 3.34E-07 | -0.03152 | 1.02±0.09 | 0.922014 |
| BdPP2C44 | -1.84916 | 3.6±0.08 | 0.028648 | -0.21606 | 1.16±4.55 | 0.567364 |
| BdPP2C45 | 1.075978 | 0.47±0.07 | 0.001683 | 0.264181 | 0.83±0.01 | 0.599501 |
| BdPP2C46 | -1.80896 | 3.5±0.82 | 0.00049 | -1.53525 | 2.9±0.11 | 0.002551 |
| BdPP2C47 | -4.94706 | 30.85±0.17 | 3.46E-05 | -1.37801 | 2.6±0.35 | 0.025485 |
| BdPP2C48 | 0.061999 | 0.96±0.08 | 0.811099 | 0.557109 | 0.68±0.02 | 0.164834 |
| BdPP2C49 | -0.55243 | 1.47±0.03 | 0.094817 | 0.912183 | 0.53±0.35 | 0.108532 |
| BdPP2C50 | -0.20613 | 1.15±0.08 | 0.270636 | 0.064408 | 0.96±0.04 | 0.871681 |
| BdPP2C51 | -0.06159 | 1.04±0.21 | 0.60223 | 0.326822 | 0.8±0.08 | 0.081476 |
| BdPP2C52 | 0.449874 | 0.73±0.17 | 0.418626 | 0.766556 | 0.59±1.19 | 0.134758 |
| BdPP2C53 | 0.808 | 0.57±0.07 | 0.047332 | -0.31073 | 1.24±0.06 | 0.423635 |
| BdPP2C54 | -0.5046 | 1.42±0.13 | 0.26264 | 1.107828 | 0.46±0.32 | 0.006688 |
| BdPP2C55 | 0.775222 | 0.58±0.1 | 0.110895 | -0.48406 | 1.4±0.2 | 0.211516 |
| BdPP2C57 | -0.45708 | 1.37±0.12 | 0.34369 | -0.22309 | 1.17±0.67 | 0.460248 |
| BdPP2C58 | 0.056205 | 0.96±0.03 | 0.835699 | 0.045858 | 0.97±0.19 | 0.878598 |
| BdPP2C59 | -0.70528 | 1.63±0.08 | 0.30455 | -0.21762 | 1.16±0.74 | 0.323706 |
| BdPP2C60 | 0.424578 | 0.75±0.03 | 0.272141 | -0.12644 | 1.09±0.34 | 0.615603 |
| BdPP2C61 | -0.02842 | 1.02±0.02 | 0.863772 | -0.11052 | 1.08±0.35 | 0.840353 |
| BdPP2C62 | 0.151789 | 0.9±0.06 | 0.724542 | -0.52119 | 1.44±0.18 | 0.331268 |
| BdPP2C64 | 0.602728 | 0.66±0.07 | 0.06304 | 0.158749 | 0.9±0.1 | 0.578536 |
| BdPP2C65 | -1.28491 | 2.44±0.79 | 0.000182 | 0.375129 | 0.77±0.15 | 0.471205 |
| BdPP2C66 | -0.27486 | 1.21±0.03 | 0.645246 | 0.680417 | 0.62±0.02 | 0.26559 |
| BdPP2C67 | -0.06325 | 1.04±0.48 | 0.834444 | 0.158741 | 0.9±0.02 | 0.614546 |
| BdPP2C68 | -0.09694 | 1.07±0.38 | 0.590596 | 0.489776 | 0.71±0.08 | 0.10245 |
| BdPP2C69 | 0.630559 | 0.65±0.17 | 0.121883 | 0.118971 | 0.92±0.18 | 0.782778 |
| BdPP2C70 | -1.04899 | 2.07±0.84 | 0.025065 | -2.00398 | 4.01±0.07 | 0.00024 |
| BdPP2C71 | 0.15376 | 0.9±0.02 | 0.728009 | -0.181 | 1.13±0.44 | 0.600039 |
| BdPP2C72 | 0.682184 | 0.62±0.04 | 0.127658 | -0.0276 | 1.02±0.37 | 0.883712 |
| BdPP2C73 | 0.37819 | 0.77±0.06 | 0.258367 | -0.04088 | 1.03±0.05 | 0.886449 |
| BdPP2C74 | 1.733989 | 0.3±0.05 | 0.019441 | 0.441699 | 0.74±0.14 | 0.303108 |
| BdPP2C75 | -0.8337 | 1.78±0.13 | 0.576577 | 0.719272 | 0.61±0.03 | 0.124473 |
| BdPP2C76 | 0.455563 | 0.73±0.06 | 0.082436 | 0.282977 | 0.82±0.19 | 0.425273 |
| BdPP2C77 | 1.096678 | 0.47±0.08 | 0.133058 | -0.35267 | 1.28±0.25 | 0.617052 |
| BdPP2C79 | -0.57225 | 1.49±0.16 | 0.629339 | -0.02809 | 1.02±0.06 | 0.929311 |
| BdPP2C80 | 0.197078 | 0.87±0.07 | 0.327019 | 0.707172 | 0.61±0.08 | 0.090096 |
| BdPP2C81 | 0.028591 | 0.98±0.05 | 0.928566 | 0.515215 | 0.7±0.01 | 0.051352 |
| BdPP2C82 | 0.432235 | 0.74±0.07 | 0.180198 | -0.07649 | 1.05±0.14 | 0.811649 |
| BdPP2C83 | -0.55711 | 1.47±0.06 | 0.386391 | 0.056483 | 0.96±0.06 | 0.828329 |
| BdPP2C84 | 2.679764 | 0.16±0.08 | 0.000586 | -0.85115 | 1.8±0.1 | 0.098725 |
| BdPP2C85 | -0.28045 | 1.21±0.2 | 0.530597 | -0.36781 | 1.29±0.07 | 0.130538 |
| BdPP2C86 | 0.15003 | 0.9±0.04 | 0.423694 | 0.012706 | 0.99±0.14 | 0.956711 |
|  | **ZnCl_2_-3h** |  |  | **ZnCl_2_-6h** |  |  |
| **BdPP2C** | **average** | **fold-change** | **p-value** | **average** | **fold-change** | **p-value** |
| BdPP2C1 | 0.240901 | 0.85±0.03 | 0.5295 | -0.11451 | 1.08±0.01 | 0.789458 |
| BdPP2C2 | -2.18898 | 4.56±0.06 | 0.02083 | 0.084376 | 0.94±0.06 | 0.778847 |
| BdPP2C3 | -0.0705 | 1.05±0.12 | 0.880071 | 0.263427 | 0.83±0.05 | 0.457027 |
| BdPP2C4 | -0.53051 | 1.44±0.03 | 0.420568 | -0.15111 | 1.11±0.01 | 0.561486 |
| BdPP2C5 | 0.353768 | 0.78±0.12 | 0.533648 | 0.018297 | 0.99±0.04 | 0.963129 |
| BdPP2C6 | 0.494411 | 0.71±0.06 | 0.15083 | -0.71668 | 1.64±0.06 | 0.13864 |
| BdPP2C7 | 0.042738 | 0.97±0.33 | 0.876145 | -0.3496 | 1.27±0.05 | 0.445814 |
| BdPP2C8 | 0.456439 | 0.73±0.03 | 0.308935 | 0.011173 | 0.99±0.03 | 0.965636 |
| BdPP2C9 | -0.5369 | 1.45±0.08 | 0.355278 | 0.112719 | 0.92±0.04 | 0.62171 |
| BdPP2C10 | 0.203812 | 0.87±0.04 | 0.085748 | 0.021448 | 0.99±0.08 | 0.959613 |
| BdPP2C11 | -0.21564 | 1.16±0.05 | 0.657957 | 0.342218 | 0.79±0.03 | 0.195678 |
| BdPP2C12 | 1.414372 | 0.38±0.22 | 0.005055 | 0.215504 | 0.86±0.02 | 0.58029 |
| BdPP2C13 | -2.11261 | 4.32±0.13 | 0.003592 | -0.85436 | 1.81±0.14 | 0.000832 |
| BdPP2C14 | 0.356853 | 0.78±0.13 | 0.304909 | 0.170849 | 0.89±0.06 | 0.611154 |
| BdPP2C16 | 0.243997 | 0.84±0.04 | 0.216003 | 0.421648 | 0.75±0.08 | 0.229557 |
| BdPP2C19 | 0.128542 | 0.91±0.29 | 0.778938 | 0.430366 | 0.74±0.05 | 0.275651 |
| BdPP2C20 | -0.57229 | 1.49±0.3 | 0.378966 | 0.561538 | 0.68±0.02 | 0.361403 |
| BdPP2C22 | -0.62648 | 1.54±0.13 | 0.130883 | 0.799013 | 0.57±0.01 | 0.037369 |
| BdPP2C23 | -0.46062 | 1.38±0.25 | 0.513178 | 1.043274 | 0.49±0.1 | 0.107739 |
| BdPP2C24 | -0.07778 | 1.06±0.13 | 0.809931 | 0.75147 | 0.59±0.1 | 0.66283 |
| BdPP2C25 | -0.19507 | 1.14±0.06 | 0.340829 | 0.574129 | 0.67±0.07 | 0.152254 |
| BdPP2C26 | -0.9226 | 1.9±0.12 | 0.037587 | 1.565952 | 0.34±0.02 | 0.008528 |
| BdPP2C28 | 0.347266 | 0.79±0.02 | 0.766711 | 2.973233 | 0.13±0.01 | 2.54E-06 |
| BdPP2C29 | -2.47548 | 5.56±0.11 | 0.050742 | 0.398146 | 0.76±0.16 | 0.182371 |
| BdPP2C30 | -0.70365 | 1.63±0.34 | 0.163997 | 0.072078 | 0.95±0.1 | 0.916003 |
| BdPP2C31 | 1.64661 | 0.32±0.05 | 0.001521 | 0.504154 | 0.71±0.11 | 0.298582 |
| BdPP2C32 | -8.38176 | 333.55±0.04 | 0.000351 | -3.75533 | 13.5±0.19 | 0.013661 |
| BdPP2C33 | 1.492142 | 0.36±0.03 | 0.000924 | -0.1863 | 1.14±0.09 | 0.520462 |
| BdPP2C34 | -0.44618 | 1.36±0.05 | 0.124458 | 1.344665 | 0.39±0.15 | 0.004388 |
| BdPP2C35 | -0.63059 | 1.55±0.27 | 0.121695 | 0.436699 | 0.74±0.02 | 0.208917 |
| BdPP2C36 | 0.093945 | 0.94±0.16 | 0.934267 | 0.2686 | 0.83±0.08 | 0.750555 |
| BdPP2C37 | 1.077044 | 0.47±0.06 | 0.012405 | -0.1083 | 1.08±0.1 | 0.825712 |
| BdPP2C38 | -3.23196 | 9.4±1.36 | 0.004441 | 1.531056 | 0.35±0.02 | 0.078426 |
| BdPP2C39 | 6.206648 | 0.01±0.002 | 6.62E-07 | -6.1805 | 72.53±0.31 | 4.42E-09 |
| BdPP2C40 | 0.636058 | 0.64±0.03 | 0.143106 | -0.02552 | 1.02±0.16 | 0.940601 |
| BdPP2C41 | 0.421491 | 0.75±0.01 | 0.325005 | 0.020417 | 0.99±0.14 | 0.984998 |
| BdPP2C42 | -0.22902 | 1.17±0.21 | 0.537887 | -0.16692 | 1.12±0.14 | 0.468469 |
| BdPP2C43 | 1.432765 | 0.37±0.2 | 0.002842 | 0.037145 | 0.97±0.06 | 0.888298 |
| BdPP2C44 | -1.53978 | 2.91±0.32 | 0.101838 | 0.790281 | 0.58±0.05 | 0.066693 |
| BdPP2C45 | 1.306901 | 0.4±0.04 | 0.022901 | 0.340477 | 0.79±0.08 | 0.463897 |
| BdPP2C46 | -0.2459 | 1.19±0.09 | 0.517461 | -0.45188 | 1.37±0.31 | 0.173697 |
| BdPP2C47 | -3.26795 | 9.63±0.17 | 0.00016 | -1.80279 | 3.49±0.11 | 0.124406 |
| BdPP2C48 | 0.425191 | 0.74±0.03 | 0.081512 | 0.239356 | 0.85±0.06 | 0.522335 |
| BdPP2C49 | -0.15893 | 1.12±0.23 | 0.659058 | 0.585883 | 0.67±0.02 | 0.231466 |
| BdPP2C50 | 0.383722 | 0.77±0.06 | 0.404137 | 0.1607 | 0.89±0.02 | 0.646896 |
| BdPP2C51 | 0.765361 | 0.59±0.02 | 0.092309 | 0.141925 | 0.91±0.01 | 0.531009 |
| BdPP2C52 | -0.42594 | 1.34±0.2 | 0.491048 | 0.156103 | 0.9±0.16 | 0.750677 |
| BdPP2C53 | 1.377477 | 0.38±0.02 | 0.02304 | -0.48675 | 1.4±0.11 | 0.320742 |
| BdPP2C54 | 0.133799 | 0.91±0.06 | 0.683387 | 1.132851 | 0.46±0.13 | 0.002119 |
| BdPP2C55 | 1.423989 | 0.37±0.05 | 0.024372 | -0.19017 | 1.14±0.18 | 0.659849 |
| BdPP2C57 | -0.05705 | 1.04±0.07 | 0.909367 | -0.08114 | 1.06±0.16 | 0.793076 |
| BdPP2C58 | 0.30943 | 0.81±0.18 | 0.334586 | -0.19711 | 1.15±0.09 | 0.568916 |
| BdPP2C59 | -0.47664 | 1.39±0.08 | 0.467073 | 0.182396 | 0.88±0.62 | 0.166243 |
| BdPP2C60 | 0.783717 | 0.58±0.02 | 0.077007 | -0.53298 | 1.45±0.2 | 0.058716 |
| BdPP2C61 | 0.205477 | 0.87±0.19 | 0.483171 | -0.39682 | 1.32±0.2 | 0.074956 |
| BdPP2C62 | -0.25891 | 1.2±0.05 | 0.280995 | -0.65432 | 1.57±0.22 | 0.136323 |
| BdPP2C64 | 1.142234 | 0.45±0.03 | 0.01766 | -0.04596 | 1.03±0.12 | 0.905053 |
| BdPP2C65 | 0.004298 | 1±0.03 | 0.960067 | -0.11548 | 1.08±0.09 | 0.75801 |
| BdPP2C66 | 0.573067 | 0.67±0.13 | 0.389216 | 0.213946 | 0.86±0.12 | 0.776998 |
| BdPP2C67 | -0.25608 | 1.19±0.18 | 0.443328 | -0.13183 | 1.1±0.03 | 0.51954 |
| BdPP2C68 | 0.227359 | 0.85±0.13 | 0.474386 | -0.04801 | 1.03±0.12 | 0.825761 |
| BdPP2C69 | 1.203 | 0.43±0.06 | 0.026498 | 0.333328 | 0.79±0.14 | 0.523787 |
| BdPP2C70 | -0.04301 | 1.03±0.23 | 0.796054 | -0.31982 | 1.25±0.08 | 0.201286 |
| BdPP2C71 | 0.270348 | 0.83±0.13 | 0.535933 | -0.78471 | 1.72±0.37 | 0.074306 |
| BdPP2C72 | 0.254584 | 0.84±0.03 | 0.532936 | -1.12916 | 2.19±0.26 | 0.001203 |
| BdPP2C73 | 0.838428 | 0.56±0.06 | 0.132882 | -0.26098 | 1.2±0.16 | 0.451631 |
| BdPP2C74 | 1.801357 | 0.29±0.08 | 0.01224 | 0.018266 | 0.99±0.18 | 0.968333 |
| BdPP2C75 | 0.340549 | 0.79±0.04 | 0.800551 | -0.18428 | 1.14±0.01 | 0.633839 |
| BdPP2C76 | 0.386472 | 0.76±0.08 | 0.103185 | 0.128447 | 0.91±0.16 | 0.759937 |
| BdPP2C77 | 1.130655 | 0.46±0.1 | 0.096644 | 0.071553 | 0.95±0.18 | 0.927743 |
| BdPP2C79 | -0.95462 | 1.94±0.3 | 0.438981 | 0.232434 | 0.85±0.18 | 0.519397 |
| BdPP2C80 | -0.01031 | 1.01±0.05 | 0.963391 | 0.232434 | 0.85±0.24 | 0.519397 |
| BdPP2C81 | 0.68182 | 0.62±0.06 | 0.005653 | 0.334419 | 0.79±0.11 | 0.152858 |
| BdPP2C82 | 0.306738 | 0.81±0.07 | 0.258505 | -0.27415 | 1.21±0.27 | 0.44277 |
| BdPP2C83 | -0.56214 | 1.48±0.42 | 0.393112 | -0.01432 | 1.01±0.18 | 0.824111 |
| BdPP2C84 | 2.924907 | 0.13±0.06 | 0.000359 | -0.59968 | 1.52±0.12 | 0.218145 |
| BdPP2C85 | -0.2736 | 1.21±0.05 | 0.482994 | -0.0006 | 1±0.31 | 0.998129 |
| BdPP2C86 | -0.02941 | 1.02±0.37 | 0.804481 | -0.22071 | 1.17±0.01 | 0.37886 |

**Supplemental Table 5The expression profile date of *BdPP2C* genes under biotic stress**

|  | **PH14-4h** |  |  | **PH14-12h** | |  |  |
| --- | --- | --- | --- | --- | --- | --- | --- |
| **BdPP2C** | **average** | **fold-change** | **p-value** | **average** | | **fold-change** | **p-value** |
| BdPP2C1 | -0.11558 | 1.08±0.05 | 0.052084 | 0.154018 | | 0.9±0.08 | 0.634086 |
| BdPP2C2 | 1.121653 | 0.46±0.22 | 0.00052 | 1.112528 | | 0.46±0.1 | 7.85E-05 |
| BdPP2C3 | 0.805849 | 0.57±0.29 | 0.000401 | 1.36539 | | 0.39±0.06 | 4.02E-05 |
| BdPP2C4 | 1.105681 | 0.46±0.28 | 6.84E-05 | 1.930018 | | 0.26±0.2 | 0.006418 |
| BdPP2C5 | -0.14948 | 1.11±0.06 | 0.636973 | 0.01321 | | 0.99±0.03 | 0.9096 |
| BdPP2C6 | -0.06072 | 1.04±0.37 | 0.829076 | 1.140471 | | 0.45±0.09 | 3.25E-05 |
| BdPP2C7 | -0.14261 | 1.1±0.44 | 0.615192 | 0.297897 | | 0.81±0.13 | 0.327497 |
| BdPP2C8 | 0.03046 | 0.98±0.04 | 0.893141 | 1.177329 | | 0.44±0.13 | 0.000214 |
| BdPP2C9 | 2.049026 | 0.24±0.14 | 6.29E-07 | 2.453489 | | 0.18±0.05 | 1.89E-06 |
| BdPP2C10 | 4.239271 | 0.05±0.03 | 1.77E-07 | 4.485647 | | 0.04±0.01 | 6.93E-09 |
| BdPP2C11 | 2.390167 | 0.19±0.09 | 2.74E-06 | 2.955861 | | 0.13±0.01 | 7.9E-07 |
| BdPP2C12 | -0.26698 | 1.2±0.4 | 0.356363 | 1.087811 | | 0.47±0.08 | 1.4E-09 |
| BdPP2C13 | -1.41182 | 2.66±0.49 | 0.000104 | -0.11022 | | 1.08±0.08 | 0.583957 |
| BdPP2C14 | 0.930601 | 0.52±0.28 | 0.345986 | 0.932531 | | 0.52±0.02 | 0.352342 |
| BdPP2C16 | -0.0495 | 1.03±0.38 | 0.870488 | 1.091553 | | 0.47±0.11 | 3.58E-05 |
| BdPP2C19 | 0.131085 | 0.91±0.33 | 0.60852 | 1.209746 | | 0.43±0.06 | 7.79E-05 |
| BdPP2C20 | 2.00545 | 0.25±0.12 | 3.66E-07 | 2.085613 | | 0.24±0.07 | 2.68E-05 |
| BdPP2C22 | 0.91518 | 0.53±0.09 | 0.139967 | 2.660139 | | 0.16±0.02 | 0.007846 |
| BdPP2C23 | 1.536974 | 0.34±0.03 | 0.005563 | 1.652754 | | 0.32±0.09 | 0.006973 |
| BdPP2C24 | -0.03239 | 1.02±0.04 | 0.74609 | 4.786867 | | 0.04±0.07 | 0.000131 |
| BdPP2C25 | -0.7126 | 1.64±0.06 | 0.08822 | -0.29882 | | 1.23±0.18 | 0.149465 |
| BdPP2C26 | -0.80885 | 1.75±0.4 | 0.054852 | 2.590715 | | 0.17±0.06 | 0.000234 |
| BdPP2C28 | 0.430926 | 0.74±0.06 | 0.328769 | 1.79328 | | 0.29±0.01 | 0.00841 |
| BdPP2C29 | -0.68812 | 1.61±0.06 | 0.075684 | 0.057321 | | 0.96±0.15 | 0.64981 |
| BdPP2C30 | 0.419751 | 0.75±0.03 | 0.219606 | 0.984401 | | 0.51±0.08 | 0.000328 |
| BdPP2C31 | 1.668144 | 0.31±0.15 | 1.38E-06 | 2.376277 | | 0.19±0.03 | 3.54E-07 |
| BdPP2C32 | 1.051236 | 0.48±0.29 | 0.002407 | 4.780649 | | 0.04±0.08 | 8.16E-06 |
| BdPP2C33 | 0.315045 | 0.8±0.18 | 0.091403 | 2.115126 | | 0.23±0.22 | 0.031992 |
| BdPP2C34 | 1.492457 | 0.36±0.04 | 0.053091 | 1.045218 | | 0.48±0.04 | 0.011928 |
| BdPP2C35 | 0.066788 | 0.95±0.3 | 0.814652 | 0.426581 | | 0.74±0.04 | 0.167446 |
| BdPP2C36 | 0.525047 | 0.69±0 | 0.078001 | 3.066653 | | 0.12±0.37 | 0.00063 |
| BdPP2C38 | 0.312579 | 0.81±0.28 | 0.302444 | 1.389422 | | 0.38±0.1 | 1.38E-05 |
| BdPP2C39 | 0.30759 | 0.81±0.01 | 0.358359 | -0.36622 | | 1.29±0.0002 | 0.339088 |
| BdPP2C40 | -0.01365 | 1.01±0.44 | 0.914191 | 0.223023 | | 0.86±0.06 | 0.44168 |
| BdPP2C41 | 5.544345 | 0.02±0.02 | 3.55E-05 | 5.98492 | | 0.02±0.01 | 1.59E-06 |
| BdPP2C42 | 0.475039 | 0.72±0.0002 | 0.096139 | 1.835818 | | 0.28±0.0001 | 0.000423 |
| BdPP2C43 | 1.761463 | 0.29±0.15 | 1.58E-09 | 2.134233 | | 0.23±0.06 | 1.07E-05 |
| BdPP2C44 | 2.278574 | 0.21±0.12 | 8.02E-07 | 1.348216 | | 0.39±0.06 | 0.008997 |
| BdPP2C45 | 0.160184 | 0.89±0.26 | 0.585985 | 0.230501 | | 0.85±0.06 | 0.475819 |
| BdPP2C46 | 2.853365 | 0.14±0.09 | 0.000598 | 3.515971 | | 0.09±0.01 | 0.002827 |
| BdPP2C47 | 9.529465 | 0.0013±0.0003 | 2.83E-07 | 9.181551 | | 0.0017±0.0015 | 1.45E-06 |
| BdPP2C48 | -0.02434 | 1.02±0.28 | 0.926299 | 0.805393 | | 0.57±0.05 | 0.000283 |
| BdPP2C49 | -0.31739 | 1.25±0.05 | 0.336552 | 0.234958 | | 0.85±0.1 | 0.415138 |
| BdPP2C50 | 0.111737 | 0.93±0.07 | 0.345781 | 0.642768 | | 0.64±0.04 | 0.019161 |
| BdPP2C51 | 2.167015 | 0.22±0.33 | 0.000482 | 2.16428 | | 0.22±0.06 | 0.001096 |
| BdPP2C52 | 0.111737 | 0.93±0.13 | 0.345781 | 0.142768 | | 0.91±0.37 | 0.401455 |
| BdPP2C53 | 3.284523 | 0.1±0.03 | 2.6E-05 | 2.477403 | | 0.18±0.05 | 1.37E-05 |
| BdPP2C54 | 1.765312 | 0.29±0.01 | 3.25E-06 | 1.973644 | | 0.25±0.07 | 3.28E-05 |
| BdPP2C55 | -0.19069 | 1.14±0.07 | 0.481062 | 1.109294 | | 0.46±0.09 | 5.27E-06 |
| BdPP2C57 | -0.03861 | 1.03±0.02 | 0.881302 | 0.518214 | | 0.7±0.09 | 0.143621 |
| BdPP2C58 | 0.130595 | 0.91±0.05 | 0.053352 | 0.509417 | | 0.7±0.13 | 0.000674 |
| BdPP2C59 | -0.14885 | 1.11±0.07 | 0.187301 | 0.192823 | | 0.87±0.27 | 0.112811 |
| BdPP2C60 | 0.135296 | 0.91±0.36 | 0.1907 | -0.02447 | | 1.02±0.18 | 0.668742 |
| BdPP2C61 | -0.35978 | 1.28±0.27 | 0.202031 | 1.105173 | | 0.46±0.3 | 0.002622 |
| BdPP2C62 | -1.80525 | 3.49±0.01 | 2.66E-05 | -0.15309 | | 1.11±0.03 | 0.588211 |
| BdPP2C64 | -0.19515 | 1.14±0.08 | 0.563236 | 0.253055 | | 0.84±0.23 | 0.092061 |
| BdPP2C65 | 4.008989 | 0.06±0.01 | 1.05E-05 | 4.358526 | | 0.05±0.03 | 6.08E-05 |
| BdPP2C66 | 1.595146 | 0.33±0.04 | 6.15E-06 | 2.08412 | | 0.24±0.04 | 0.001852 |
| BdPP2C67 | 1.779341 | 0.29±0.02 | 4.77E-05 | 2.082096 | | 0.24±0.05 | 7.8E-05 |
| BdPP2C68 | 0.204154 | 0.87±0.08 | 0.540165 | 0.249225 | | 0.84±0.23 | 0.058651 |
| BdPP2C69 | -0.33658 | 1.26±0.15 | 0.267035 | 0.247413 | | 0.84±0.08 | 0.453118 |
| BdPP2C70 | 0.642359 | 0.64±0.13 | 0.006667 | 0.983681 | | 0.51±0.01 | 0.000897 |
| BdPP2C71 | 0.165812 | 0.89±0.13 | 0.487814 | -0.0644 | | 1.05±0.27 | 0.287785 |
| BdPP2C72 | 0.331171 | 0.79±0.16 | 0.202578 | 0.306367 | | 0.81±0.02 | 0.374167 |
| BdPP2C73 | 2.461518 | 0.18±0.04 | 1.36E-05 | 2.627033 | | 0.16±0.11 | 5.7E-06 |
| BdPP2C74 | 0.290401 | 0.82±0.09 | 0.318361 | 1.305547 | | 0.4±0.06 | 1.07E-07 |
| BdPP2C75 | -0.89251 | 1.86±0.07 | 0.437434 | -0.09894 | | 1.07±0.47 | 0.498034 |
| BdPP2C76 | 0.222618 | 0.86±0.31 | 0.457376 | 1.007586 | | 0.5±0.09 | 1.76E-06 |
| BdPP2C77 | -0.15948 | 1.12±0.04 | 0.587231 | 0.068087 | | 0.95±0.13 | 0.825127 |
| BdPP2C79 | -0.63113 | 1.55±0.06 | 0.07858 | 0.149258 | | 0.9±0.3 | 0.186426 |
| BdPP2C80 | 1.142185 | 0.45±0.13 | 0.001449 | 0.971928 | | 0.51±0.03 | 0.000256 |
| BdPP2C81 | 0.161052 | 0.89±0.16 | 0.129312 | -0.07645 | | 1.05±0.15 | 0.792819 |
| BdPP2C82 | -0.39402 | 1.31±0.15 | 0.234012 | -0.22178 | | 1.17±0.2 | 0.411608 |
| BdPP2C83 | 0.97873 | 0.51±0.02 | 0.1224 | -0.61655 | | 1.53±0.08 | 0.075105 |
| BdPP2C84 | 1.597857 | 0.33±0.17 | 2.06E-07 | 1.609224 | | 0.33±0.09 | 1.91E-05 |
| BdPP2C85 | 2.015135 | 0.25±0.02 | 1.01E-05 | 2.090358 | | 0.23±0.04 | 6.88E-07 |
| BdPP2C86 | 1.112198 | 0.46±0.01 | 1.92E-06 | 1.84252 | | 0.28±0.06 | 9.85E-06 |
|  | **Guy11-4h** |  |  | **Guy11-12h** | | |  |
| **BdPP2C** | **average** | **fold-change** | **p-value** | **average** | **fold-change** | | **p-value** |
| BdPP2C1 | 1.016257 | 0.49±0.17 | 1.39E-05 | 0.185966 | 0.88±0.15 | | 0.505223 |
| BdPP2C2 | 1.775733 | 0.29±0.11 | 2.26E-05 | 1.448598 | 0.37±0.14 | | 2.06E-05 |
| BdPP2C3 | 1.91598 | 0.26±0.08 | 1.07E-05 | 1.700905 | 0.31±0.07 | | 8.79E-06 |
| BdPP2C4 | 2.006711 | 0.25±0.11 | 6.44E-06 | 0.151001 | 0.9±0.37 | | 0.544099 |
| BdPP2C5 | -0.03224 | 1.02±0.11 | 0.910698 | -0.17376 | 1.13±0.1 | | 0.274885 |
| BdPP2C6 | 1.593122 | 0.33±0.12 | 1.69E-05 | 1.017573 | 0.49±0.14 | | 4.8E-06 |
| BdPP2C7 | 1.592182 | 0.33±0.1 | 5.62E-06 | 0.855683 | 0.55±0.17 | | 2.06E-05 |
| BdPP2C8 | 1.456367 | 0.36±0.24 | 0.000832 | -0.02178 | 1.02±0.29 | | 0.939852 |
| BdPP2C9 | 3.118292 | 0.12±0.04 | 5.13E-08 | 2.73023 | 0.15±0.05 | | 4E-07 |
| BdPP2C10 | 5.234479 | 0.03±0.01 | 9.5E-09 | 4.914477 | 0.03±0.01 | | 4.05E-10 |
| BdPP2C11 | 3.59772 | 0.08±0.03 | 1.01E-08 | 2.755122 | 0.15±0.05 | | 5.81E-09 |
| BdPP2C12 | 1.620461 | 0.33±0.1 | 2.57E-07 | 1.182456 | 0.44±0.15 | | 6.71E-07 |
| BdPP2C13 | 0.177106 | 0.88±0.31 | 0.551083 | 0.079415 | 0.95±0.13 | | 0.59212 |
| BdPP2C14 | -0.06558 | 1.05±0.18 | 0.946131 | -0.28072 | 1.21±0.4 | | 0.781348 |
| BdPP2C16 | 1.658854 | 0.32±0.09 | 2.63E-06 | 0.972321 | 0.51±0.2 | | 4.42E-05 |
| BdPP2C19 | 1.622094 | 0.32±0.11 | 7.51E-05 | 0.280542 | 0.82±0.21 | | 0.244388 |
| BdPP2C20 | 2.447068 | 0.18±0.07 | 1.82E-07 | 1.791931 | 0.29±0.09 | | 7.16E-07 |
| BdPP2C22 | 3.394069 | 0.1±0.001 | 0.02206 | 0.450418 | 0.73±0.02 | | 0.409607 |
| BdPP2C23 | 2.511929 | 0.18±0.1 | 0.000855 | 2.129009 | 0.23±0.22 | | 0.002546 |
| BdPP2C24 | -0.3139 | 1.24±0.27 | 0.168843 | 0.189739 | 0.88±0.002 | | 0.176357 |
| BdPP2C25 | -0.20931 | 1.16±0.26 | 0.416017 | 0.463208 | 0.73±0.21 | | 0.008103 |
| BdPP2C26 | 0.275706 | 0.83±0.15 | 0.48099 | 7.524532 | 0.01±0.09 | | 3.42E-06 |
| BdPP2C28 | 0.162298 | 0.89±0.25 | 0.594868 | 1.87721 | 0.27±0.05 | | 0.013122 |
| BdPP2C29 | 0.146498 | 0.9±0.14 | 0.165979 | 0.388428 | 0.76±0.29 | | 0.051323 |
| BdPP2C30 | 1.727659 | 0.3±0.09 | 0.000116 | 1.065361 | 0.48±0.13 | | 0.001518 |
| BdPP2C31 | 3.011958 | 0.12±0.03 | 5.41E-07 | 2.272885 | 0.21±0.04 | | 4.25E-06 |
| BdPP2C32 | 0.00136 | 1±0.06 | 0.992203 | 0.915557 | 0.53±0.05 | | 4.74E-05 |
| BdPP2C33 | 1.61058 | 0.33±0.07 | 8.16E-05 | 0.153886 | 0.9±0.05 | | 0.498012 |
| BdPP2C34 | 0.086248 | 0.94±0.01 | 0.509566 | -0.04181 | 1.03±0.04 | | 0.891229 |
| BdPP2C35 | 1.457681 | 0.36±0.11 | 3.57E-06 | 1.127339 | 0.46±0.17 | | 0.000236 |
| BdPP2C36 | 0.249428 | 0.84±0.01 | 0.235913 | -0.29644 | 1.23±0.03 | | 0.213311 |
| BdPP2C38 | 2.251775 | 0.21±0.07 | 5.93E-08 | 1.732679 | 0.3±0.09 | | 8.81E-07 |
| BdPP2C39 | 2.104541 | 0.23±0.02 | 0.001138 | 1.637963 | 0.32±0.1 | | 0.00851 |
| BdPP2C40 | 1.301985 | 0.41±0.07 | 0.00033 | 0.146639 | 0.9±0.14 | | 0.636048 |
| BdPP2C41 | 1.494709 | 0.35±0.11 | 0.00039 | 0.493313 | 0.71±0.08 | | 0.064534 |
| BdPP2C42 | 0.416253 | 0.75±0.08 | 0.156272 | 1.323884 | 0.4±0.01 | | 0.001756 |
| BdPP2C43 | 3.142869 | 0.11±0.03 | 4.4E-07 | 2.056992 | 0.24±0.08 | | 9.41E-10 |
| BdPP2C44 | 2.832621 | 0.14±0.08 | 3.07E-05 | 2.014719 | 0.25±0.03 | | 0.000428 |
| BdPP2C45 | 1.504995 | 0.35±0.09 | 3.34E-05 | 1.144695 | 0.45±0.11 | | 0.000105 |
| BdPP2C46 | 3.841545 | 0.07±0.03 | 0.000299 | 2.943197 | 0.13±0.17 | | 0.002451 |
| BdPP2C47 | 2.270962 | 0.21±0.11 | 0.000111 | 1.766197 | 0.29±0.12 | | 2.96E-05 |
| BdPP2C48 | 1.822877 | 0.28±0.06 | 1.77E-05 | 1.350585 | 0.39±0.09 | | 3.85E-05 |
| BdPP2C49 | 1.830124 | 0.28±0.09 | 2.96E-06 | 0.403883 | 0.76±0.16 | | 0.193791 |
| BdPP2C50 | 1.423692 | 0.37±0.08 | 3.59E-05 | 0.637952 | 0.64±0.05 | | 0.001013 |
| BdPP2C51 | 1.898944 | 0.27±0.09 | 0.005215 | 1.908579 | 0.27±0.13 | | 4.63E-05 |
| BdPP2C52 | 1.423692 | 0.37±0.2 | 3.59E-05 | 0.637952 | 0.64±0.06 | | 0.001013 |
| BdPP2C53 | 2.07833 | 0.24±0.08 | 2.53E-05 | 2.726477 | 0.15±0.05 | | 1.91E-05 |
| BdPP2C54 | 0.118277 | 0.92±0.01 | 0.426498 | 1.249764 | 0.42±0.01 | | 0.000608 |
| BdPP2C55 | 0.038366 | 0.97±0.19 | 0.1429 | 0.74039 | 0.6±0.08 | | 7.02E-06 |
| BdPP2C57 | 0.2204 | 0.86±0.14 | 0.475783 | 0.188499 | 0.88±0.01 | | 0.618929 |
| BdPP2C58 | 1.649548 | 0.32±0.1 | 2.3E-06 | 0.588616 | 0.66±0.24 | | 0.001266 |
| BdPP2C59 | 1.750789 | 0.3±0.18 | 0.000269 | -0.29753 | 1.23±0.4 | | 0.086596 |
| BdPP2C60 | 1.623871 | 0.32±0.12 | 3.17E-05 | -0.21535 | 1.16±0.19 | | 0.442128 |
| BdPP2C61 | -0.4709 | 1.39±0.25 | 0.198828 | 0.465974 | 0.72±0.13 | | 0.089078 |
| BdPP2C62 | -1.28023 | 2.43±0.04 | 3.99E-05 | 0.023174 | 0.98±0.25 | | 0.156295 |
| BdPP2C64 | 1.499023 | 0.35±0.1 | 1.74E-06 | -0.32787 | 1.26±0.29 | | 0.264976 |
| BdPP2C65 | 4.08431 | 0.06±0.06 | 0.000109 | 0.051491 | 0.96±0.08 | | 0.841004 |
| BdPP2C66 | 2.205371 | 0.22±0.12 | 1.57E-06 | 1.414972 | 0.38±0.03 | | 1.24E-05 |
| BdPP2C67 | 0.457736 | 0.73±0.04 | 0.074339 | 1.90612 | 0.27±0.08 | | 7.38E-05 |
| BdPP2C68 | 1.372232 | 0.39±0.17 | 0.000129 | 0.262435 | 0.83±0.29 | | 0.381336 |
| BdPP2C69 | 2.286066 | 0.21±0.06 | 4.4E-07 | 0.112861 | 0.92±0.17 | | 0.728275 |
| BdPP2C70 | 2.911849 | 0.13±0.05 | 1.58E-05 | 1.172937 | 0.44±0.3 | | 0.025932 |
| BdPP2C71 | 2.041257 | 0.24±0.14 | 0.000178 | 0.140464 | 0.91±0.12 | | 0.43423 |
| BdPP2C72 | 2.09944 | 0.23±0.05 | 5.92E-05 | 1.346836 | 0.39±0.03 | | 0.005303 |
| BdPP2C73 | 1.082691 | 0.47±0.2 | 0.012726 | 2.032949 | 0.24±0.03 | | 0.000143 |
| BdPP2C74 | 2.445158 | 0.18±0.04 | 8.52E-06 | 1.394447 | 0.38±0.12 | | 2.28E-07 |
| BdPP2C75 | 0.046892 | 0.97±0.58 | 0.857229 | 1.451637 | 0.37±0.13 | | 0.000457 |
| BdPP2C76 | 2.404197 | 0.19±0.03 | 0.000183 | 1.481802 | 0.36±0.09 | | 7.62E-05 |
| BdPP2C77 | 1.757379 | 0.3±0.1 | 1.1E-07 | 1.235369 | 0.42±0.13 | | 9.3E-06 |
| BdPP2C79 | 1.905088 | 0.27±0.11 | 1.25E-06 | -0.15519 | 1.11±0.32 | | 0.583678 |
| BdPP2C80 | 2.603342 | 0.16±0.05 | 5.39E-06 | 1.371008 | 0.39±0.12 | | 0.000131 |
| BdPP2C81 | 1.779499 | 0.29±0.12 | 1.56E-06 | -0.07343 | 1.05±0.18 | | 0.711564 |
| BdPP2C82 | 2.015567 | 0.25±0.09 | 1.1E-09 | 0.290796 | 0.82±0.09 | | 0.255859 |
| BdPP2C83 | -0.1807 | 1.13±0.34 | 0.399814 | -0.14208 | 1.1±0.34 | | 0.638399 |
| BdPP2C84 | 3.319534 | 0.1±0.05 | 3.63E-06 | 2.153671 | 0.22±0.05 | | 1.29E-07 |
| BdPP2C85 | 1.273574 | 0.41±0.07 | 1.18E-05 | 1.921918 | 0.26±0.06 | | 4.65E-06 |
| BdPP2C86 | 0.094989 | 0.94±0.12 | 0.766424 | 0.272499 | 0.83±0.06 | | 0.405412 |
|  | **F0968-4h** |  |  | **F0968-12h** | | |  |
| **BdPP2C** | **average** | **fold-change** | **p-value** | **average** | **fold-change** | | **p-value** |
| BdPP2C1 | 0.036417 | 0.98±0.03 | 0.892653 | -0.12563 | 1.09±0.21 | | 0.573863 |
| BdPP2C2 | 1.499176 | 0.35±0.13 | 0.000107 | 1.376131 | 0.39±0.09 | | 4.22E-05 |
| BdPP2C3 | 1.148905 | 0.45±0.2 | 0.000113 | 1.026208 | 0.49±0.01 | | 0.001894 |
| BdPP2C4 | 1.609185 | 0.33±0.18 | 1.5E-05 | 1.146247 | 0.45±0.06 | | 0.001019 |
| BdPP2C5 | 0.129568 | 0.91±0.02 | 0.645364 | 0.287158 | 0.82±0.16 | | 0.125762 |
| BdPP2C6 | 1.480697 | 0.36±0.18 | 2.62E-05 | 1.031667 | 0.49±0.12 | | 9.61E-05 |
| BdPP2C7 | 1.022519 | 0.49±0.12 | 2.52E-06 | 0.160923 | 0.89±0.02 | | 0.558255 |
| BdPP2C8 | 1.473318 | 0.36±0.17 | 0.000662 | 0.760548 | 0.59±0.06 | | 0.055063 |
| BdPP2C9 | 2.594278 | 0.17±0.09 | 4.2E-07 | 2.330151 | 0.2±0.04 | | 3.36E-07 |
| BdPP2C10 | 4.552687 | 0.04±0.02 | 3.07E-10 | 4.617602 | 0.04±0.01 | | 8.55E-09 |
| BdPP2C11 | 3.038428 | 0.12±0.05 | 7.1E-11 | 2.654135 | 0.16±0.03 | | 9.63E-08 |
| BdPP2C12 | 0.43689 | 0.74±0.02 | 0.192676 | 0.293043 | 0.82±0.1 | | 0.329829 |
| BdPP2C13 | 0.205527 | 0.87±0.19 | 0.174814 | 0.084961 | 0.94±0.02 | | 0.401478 |
| BdPP2C14 | 0.008463 | 0.99±0.17 | 0.992739 | -0.90782 | 1.88±0.27 | | 0.361707 |
| BdPP2C16 | 0.436794 | 0.74±0.02 | 0.188739 | 0.237929 | 0.85±0.07 | | 0.345404 |
| BdPP2C19 | 0.307105 | 0.81±0.01 | 0.209113 | 1.114679 | 0.46±0.14 | | 0.001937 |
| BdPP2C20 | 1.943336 | 0.26±0.15 | 2.02E-05 | 1.94615 | 0.26±0.09 | | 3.27E-05 |
| BdPP2C22 | 4.340523 | 0.05±0.01 | 0.000873 | 1.825991 | 0.28±0.001 | | 0.027421 |
| BdPP2C23 | 1.660482 | 0.32±0.14 | 0.003629 | 1.484095 | 0.36±0.11 | | 0.005068 |
| BdPP2C24 | 2.659089 | 0.16±0.0002 | 1.57E-06 | 2.992326 | 0.13±0.09 | | 1.38E-06 |
| BdPP2C25 | -0.04752 | 1.03±0.19 | 0.784981 | 1.098876 | 0.47±0.1 | | 5.02E-05 |
| BdPP2C26 | 6.267614 | 0.01±0.07 | 1.88E-05 | 0.145191 | 0.9±0.18 | | 0.60643 |
| BdPP2C28 | 2.486611 | 0.18±0.08 | 0.001467 | 1.018214 | 0.49±0.01 | | 0.054889 |
| BdPP2C29 | 1.259118 | 0.42±0.21 | 0.000189 | 0.289982 | 0.82±0.15 | | 0.050039 |
| BdPP2C30 | 0.390062 | 0.76±0.02 | 0.106526 | 1.052955 | 0.48±0.09 | | 0.000102 |
| BdPP2C31 | 2.12539 | 0.23±0.09 | 9.06E-07 | 2.058285 | 0.24±0.04 | | 5.28E-07 |
| BdPP2C32 | 2.337649 | 0.2±0.13 | 0.000909 | 5.056554 | 0.03±0.02 | | 9.31E-06 |
| BdPP2C33 | 1.150747 | 0.45±0.03 | 0.008661 | 0.478973 | 0.72±0.01 | | 0.096078 |
| BdPP2C34 | 1.694325 | 0.31±0.04 | 0.015054 | 0.129049 | 0.91±0.002 | | 0.791147 |
| BdPP2C35 | 0.441008 | 0.74±0.17 | 0.144234 | 0.337403 | 0.79±0.03 | | 0.190647 |
| BdPP2C36 | 2.029311 | 0.24±0.02 | 0.000444 | 5.855909 | 0.02±0.01 | | 5.23E-06 |
| BdPP2C38 | 1.641342 | 0.32±0.15 | 6.47E-08 | 1.545073 | 0.34±0.07 | | 9.4E-08 |
| BdPP2C39 | 1.228386 | 0.43±0.12 | 0.008973 | 1.551619 | 0.34±0.11 | | 0.004534 |
| BdPP2C40 | 0.170704 | 0.89±0.02 | 0.577985 | -0.07288 | 1.05±0.06 | | 0.819029 |
| BdPP2C41 | 2.698913 | 0.15±0.14 | 0.024487 | 1.103036 | 0.47±0.02 | | 0.002224 |
| BdPP2C42 | 0.409602 | 0.75±0.09 | 0.169279 | -0.64184 | 1.56±0.17 | | 0.043432 |
| BdPP2C43 | 2.042643 | 0.24±0.11 | 2.79E-09 | 2.231312 | 0.21±0.05 | | 5.38E-07 |
| BdPP2C44 | 2.907275 | 0.13±0.1 | 0.000116 | 2.349762 | 0.2±0.03 | | 5.52E-06 |
| BdPP2C45 | 0.180294 | 0.88±0.02 | 0.563182 | 0.282877 | 0.82±0.1 | | 0.317332 |
| BdPP2C46 | 2.732414 | 0.15±0.03 | 0.006348 | 5.268886 | 0.03±0.01 | | 3.19E-05 |
| BdPP2C47 | 0.205774 | 0.87±0.01 | 0.468997 | 0.661654 | 0.63±0.17 | | 0.054958 |
| BdPP2C48 | 1.047349 | 0.48±0.18 | 0.000107 | 0.382343 | 0.77±0.08 | | 0.243441 |
| BdPP2C49 | 1.077445 | 0.47±0.02 | 1.37E-05 | 0.139828 | 0.91±0.15 | | 0.649396 |
| BdPP2C50 | 0.545944 | 0.68±0.04 | 0.02307 | 0.859177 | 0.55±0.03 | | 0.000796 |
| BdPP2C51 | 0.348974 | 0.79±0.26 | 0.273482 | 1.857916 | 0.28±0.03 | | 0.000167 |
| BdPP2C52 | 0.195815 | 0.87±0.06 | 0.475791 | 0.462541 | 0.73±0.08 | | 0.100189 |
| BdPP2C53 | 1.012532 | 0.5±0.02 | 0.002016 | 1.912691 | 0.27±0.04 | | 3.69E-05 |
| BdPP2C54 | 0.410236 | 0.75±0.0002 | 0.282772 | 0.037433 | 0.97±0.17 | | 0.789017 |
| BdPP2C55 | -0.74489 | 1.68±0.04 | 0.050839 | 0.201296 | 0.87±0.01 | | 0.493765 |
| BdPP2C57 | 0.113856 | 0.92±0.13 | 0.571584 | -0.25603 | 1.19±0.16 | | 0.345395 |
| BdPP2C58 | 0.885053 | 0.54±0.04 | 0.00036 | 1.261021 | 0.42±0.14 | | 2.24E-05 |
| BdPP2C59 | 0.274439 | 0.83±0.03 | 0.379737 | 0.214435 | 0.86±0.03 | | 0.087292 |
| BdPP2C60 | 0.103313 | 0.93±0.16 | 0.57586 | 0.131145 | 0.91±0.05 | | 0.589735 |
| BdPP2C61 | 0.503248 | 0.71±0.15 | 0.065746 | -0.56591 | 1.48±0.3 | | 0.038713 |
| BdPP2C62 | -1.35231 | 2.55±0.03 | 0.000116 | -0.37897 | 1.3±0.13 | | 0.09764 |
| BdPP2C64 | 0.12699 | 0.92±0.14 | 0.082701 | 0.112414 | 0.93±0.13 | | 0.034019 |
| BdPP2C65 | 2.913248 | 0.13±0.03 | 3.16E-05 | 2.389366 | 0.19±0.004 | | 0.00425 |
| BdPP2C66 | 2.337881 | 0.2±0.03 | 2.52E-05 | 2.948912 | 0.13±0.03 | | 1.43E-05 |
| BdPP2C67 | -0.1378 | 1.1±0.01 | 0.459265 | 0.014123 | 0.99±0.19 | | 0.949882 |
| BdPP2C68 | 0.09087 | 0.94±0.06 | 0.386765 | -0.1809 | 1.13±0.23 | | 0.55013 |
| BdPP2C69 | 1.077958 | 0.47±0.21 | 3.33E-05 | 1.941472 | 0.26±0.06 | | 1.69E-05 |
| BdPP2C70 | 0.412376 | 0.75±0.25 | 0.116254 | 2.364893 | 0.19±0.05 | | 8.75E-05 |
| BdPP2C71 | 0.465889 | 0.72±0.07 | 0.189161 | -0.03563 | 1.03±0.04 | | 0.819549 |
| BdPP2C72 | 0.276398 | 0.83±0.17 | 0.122377 | 1.220809 | 0.43±0.01 | | 0.000576 |
| BdPP2C73 | 0.594229 | 0.66±0.03 | 0.368839 | 1.029457 | 0.49±0.06 | | 0.045277 |
| BdPP2C74 | 0.143456 | 0.91±0.22 | 0.556717 | 1.503812 | 0.35±0.05 | | 3.46E-06 |
| BdPP2C75 | -2.68657 | 6.44±1.21 | 0.043307 | 1.854287 | 0.28±0.18 | | 0.000313 |
| BdPP2C76 | -0.1714 | 1.13±0.05 | 0.610801 | 2.018499 | 0.25±0.05 | | 3.37E-07 |
| BdPP2C77 | 0.15788 | 0.9±0.05 | 0.098547 | 1.224182 | 0.43±0.09 | | 6.67E-07 |
| BdPP2C79 | -0.89756 | 1.86±0.14 | 0.021181 | 0.047556 | 0.97±0.15 | | 0.867967 |
| BdPP2C80 | -0.06864 | 1.05±0.21 | 0.795675 | 1.240873 | 0.42±0.06 | | 9.93E-05 |
| BdPP2C81 | -0.07241 | 1.05±0.16 | 0.179719 | 0.315342 | 0.8±0.2 | | 0.212551 |
| BdPP2C82 | -0.18938 | 1.14±0.14 | 0.504205 | 1.004325 | 0.5±0.09 | | 2.71E-06 |
| BdPP2C83 | -0.04488 | 1.03±0.06 | 0.893972 | -0.03027 | 1.02±0.12 | | 0.912495 |
| BdPP2C84 | 0.382255 | 0.77±0.25 | 0.235021 | 2.340196 | 0.2±0.03 | | 9.62E-07 |
| BdPP2C85 | 0.391603 | 0.76±0.01 | 0.214088 | 0.103129 | 0.93±0.04 | | 0.736909 |
| BdPP2C86 | 0.150119 | 0.9±0.14 | 0.137025 | -0.6281 | 1.55±0.03 | | 0.109853 |
